# Genome-wide investigation and expression analyses of the pentatricopeptide repeat protein gene family in foxtail millet

**DOI:** 10.1186/s12864-016-3184-2

**Published:** 2016-10-28

**Authors:** Jia-Ming Liu, Zhao-Shi Xu, Pan-Pan Lu, Wei-Wei Li, Ming Chen, Chang-Hong Guo, You-Zhi Ma

**Affiliations:** 1 Key Laboratory of Molecular Cytogenetics and Genetic Breeding of Heilongjiang Province, College of Life Science and Technology, Harbin Normal University, Harbin, 150025 China; 2 Institute of Crop Science, Chinese Academy of Agricultural Sciences/National Key Facility for Crop Gene Resources and Genetic Improvement, Key Laboratory of Biology and Genetic Improvement of Triticeae Crops, Ministry of Agriculture, Beijing, 100081 China

**Keywords:** Foxtail millet, Pentatricopeptide repeat (PPR) proteins, Genome segmental duplication, Phylogenesis, Responsive mechanism, Subcellular localization

## Abstract

**Background:**

Pentatricopeptide repeat (PPR) proteins are encoded by a large gene family of approximately 450 members in *Arabidopsis* and 477 in rice, which characterized by tandem repetitions of a degenerate 35 amino acid characteristic sequence motifs. A large majority of the PPR genes in the higher plants are localized in organelles. Their functions remain as yet largely unknown. The majority of characterized PPR proteins have been found to function in modulating the expression plastid and mitochondrial genes in plants.

**Results:**

Here, a genome-wide identification and comparison of the PPR genes from 5 organisms was performed, including the moss *Physcomitrella patens*, the lycophyte *Selaginella moellendorffii*, the eudicot *Arabidopsis*, and the monocots rice and foxtail millet. It appears that the expansion of this gene family prior to the divergence of the euphyllophytes and the lycophytes in land plants. The duplication and divergence rates of the foxtail millet PPR genes (SiPPRs) showed that the expansion period of this gene family around 400 Mya, and indicated that genome segmental duplication was very likely the primary mechanism underlying the expansion of the PPR gene family in vascular plants. An analysis of a complete set of SiPPR genes/proteins that included classification, chromosomal location, orthologous relationships, duplication analysis, and auxiliary motifs is presented. Expression analysis of the SiPPR genes under stress conditions revealed that the expression of 24 SiPPR genes was responsive to abiotic stress. Subcellular localization analysis of 11 PPR proteins indicated that 5 proteins were localized to chloroplasts, that 4 were localized to mitochondria, and that 2 were localized to the cytoplasm.

**Conclusions:**

Our results contribute to a more comprehensive understanding the roles of PPR proteins and will be useful in the prioritization of particular PPR proteins for subsequent functional validation studies in foxtail millet.

**Electronic supplementary material:**

The online version of this article (doi:10.1186/s12864-016-3184-2) contains supplementary material, which is available to authorized users.

## Background

Foxtail millet (*Setaria italica*) was domesticated in neolithic China approximately 8,700 years ago. It has been regarded as an important dietary staple in China for many millennia. Although it is closely related to the major food and feed crops maize and sorghum, the required growth conditions and low productivity have limited the potential of foxtail millet [1]. Foxtail millet has a relatively small genome (515 Mb; 2n = 2x = 18); it is a C4 panicoid crop with relatively low amounts of repetitive DNA, a short life-cycle, and is self-pollinating [2, 3]. It has prolific seed production and has close phylogenetic relationships with several biofuel crops such as switch grass (*Panicum virgatum*), napier grass (*Pennisetum purpureum*), and pearl millet (*Pennisetum glaucum*) [1]. Unsurprisingly, considering that foxtail millet is a cereal crop with excellent drought tolerance, there is an extensive germplasm collection available for research and the plant has become a prominent model genetic system for use in the study of the evolution and physiology of C4 photosynthesis and abiotic stress tolerance mechanisms, particularly for salinity and dehydration stress [3–5]. These features accentuate the potential of using this crop as an experimental model system to investigate the stress resistance mechanisms and of using this crop as a source from which to mine abiotic stress responsive genes.

The pentatricopeptide repeat (PPR) proteins represent one of the largest protein families in land plants (450 members in *Arabidopsis* and 477 members in rice). This family was identified serendipitously over a decade ago as a result of bioinformatics analyses of the then incomplete *Arabidopsis* genome sequence [6]. PPR proteins are characterized by the tandem array of a PPR motif, a highly degenerate unit consisting of 35 canonical amino acids [7–9]. The vast majority of the PPR genes in each of the higher plants with sequenced genomes were found to be lacking introns, and retrotransposition has been proposed as the likely mechanism underlying the expansion of the higher plant PPR gene family [8, 10]. The majority of plant PPR proteins that have been characterized to date function in important roles in a broad range of developmental and physiological processes such as respiration, cytoplasmic male sterility (CMS), embryogenesis, and photosynthesis, and few of these proteins have been proved to play a role in post-transcriptional processes associated with RNA in plant organelles [11–14]. PPR proteins escaped identification until complete genomic sequences became available because their primary sequences are highly degenerate. While subsequent analysis revealed that the PPR proteins are found in a few animal and fungal proteins, but it is clear that the family has expanded greatly in land plants; there are with 450 PPR genes in *Arabidopsis* and 477 PPR genes in rice (*Oryza sativa*) [6–8, 15]. The vast majority of these proteins are predicted to be localized to mitochondria or chloroplasts, however, there are also a few PPR proteins that have been shown to be localized to the nucleus [16–18]. Most of the functional analyses of PPR proteins have been performed *Arabidopsis*, rice, or maize (*Zea mays*), and these studies revealed that they participate in various post-transcriptional processes related to gene expression in plant organelles [11–14, 19–22]. Functional studies of PPR proteins relating to biotic and abiotic stress response mechanisms in higher plants remain very sparse, and the characterization of these proteins remains one a major challenge in plant science.

Here, we took advantage of the existing research results [7, 8, 10] and the newly completed foxtail millet genome sequencing results to perform a genome-wide comparison of the PPR genes in 5 organisms distributed widely across plant lineages: the moss *Physcomitrella patens* [23, 24], the lycophyte Selaginella (*Selaginella moellendorffii*) [25], the dicot *Arabidopsis*, and the monocots rice and foxtail millet [1]. Our results allow us to draw definitive conclusions on the timing and causes of the expansion of the vascular plants PPR gene family. In addition, our complete analysis of all of the PPR proteins in foxtail millet enables a more comprehensive foundation from which to explore the functional and regulatory networks of PPR genes. Our quantitative real-time PCR (qRT-PCR)-based analysis of these gene expression patterns under specific stress treatment conditions, and our subcellular localization studies of a number of candidate SiPPRs provide empirical data that should greatly facilitate advances in understanding of the biological functions of these fascinating proteins in foxtail millet in particular, and in plants generally.

## Results and Discussion

### Identification and classification of PPR proteins in foxtail millet

All of the 486 genes encoding PPR proteins in the genome of foxtail millet were identified in this paper. The Phytozome locus, protein length, open reading frame (ORF) length, and each of these PPR genes chromosomal location are listed in Additional file 1: Table S1. Foxtail millet PPR gene/protein sequence informations, and gene annotations in this study were downloaded from the Phytozome (http://phytozome.Jgi.doe.gov/pz/portal.html).

To better understand the phylogenetic relationships of the PPR protein-endoding genes among land plants, we identified 1670 nonredundant putative PPR proteins in the Selaginella, which is a nonseed vascular plant with a dominant and complex sporophyte generation of an ancient lineage that diverged shortly after land plants evolved vascular tissues [25] (Additional file 2: Table S2). The numbers of PPR genes in foxtail millet, *Arabidopsis*, and rice were found to be strikingly similar. There were approximately three times as many PPR genes in Selaginella than in foxtail millet. The Selaginella plastome sequence has a large amount of RNA-edited sites, which coincides with the exceptionally large number of PPR genes in Selaginella [25].

**Fig. 1:**
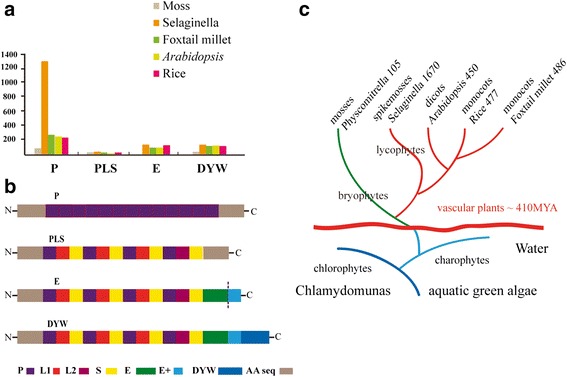
The numbers of PPR genes divided by species and subgroups and in the context of the phylogeny of plants. (**a**) Numbers and subclasses of PPR genes in moss, Selaginella, foxtail millet, *Arabidopsis*, rice. (**b**) Motif structures of PPR proteins. Schematic representation of typical plants PPR proteins from each subclass [7, 8, 10]. The number and even order of repeats can vary in individual proteins. The dashed line between the E and E+ motifs indicates that the E+ extension is not always present. (**c**) Phylogeny of plants. The species and the members of PPRs were labeled in end branch. Vascular plants appeared ~410 million years ago, then diverged into several lineages of which only two survive: the euphyllophytes (ferns and seed plants) and the lycophytes [25]

Given the structure of the repeated motif, PPR gene family can be split into two subfamilies; these are termed the P and PLS subfamilies. On the basis of the presence of the C-terminal conserved domains, the PLS subfamily can be further split into the following three subgroups: PLS, E/E+ and DYW subgroups [8]. Figure 1a details the numbers of PPR genes split by different species and subgroups and in the context of the phylogeny of plants. A large majority of the 1403 Selaginella PPRs, and 263 SiPPRs, belong to the P subfamily; 271 Selaginella PPRs, and 223 SiPPRs, belong to the PLS subfamily, including 39 and 32 PLS subgroups, 139 and 80 DYW subgroups, 93 and 111 E/E+ subgroups PPR genes were found in Selaginella and foxtail millet, respectively.

### Gene structure and chromosomal distribution of PPR genes in foxtail millet

**Fig. 2:**
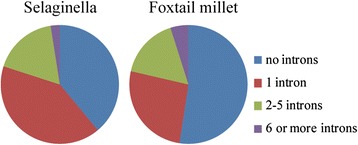
Relative proportions of intron-containing PPR genes in Selaginella and foxtail millet. Proportions were colored as: no introns (nattier blue), 1 intron (carnation), 2–5 introns (fluorescence green), and 6 or more introns (lavender)

**Fig. 3:**
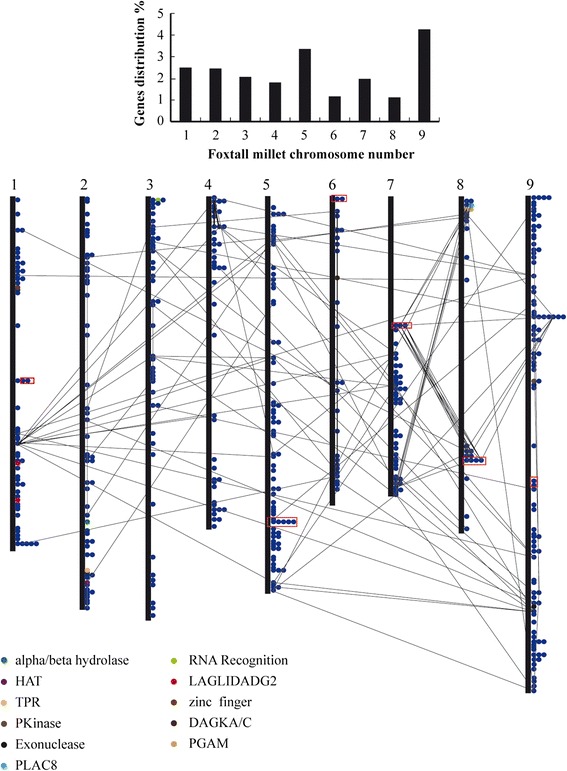
Percentage and genomic distribution of PPR genes on each the foxtail millet chromosome. PPR genes with different auxiliary domain/motif were shown in different colors. One circle represents one PPR gene. Chromosome numbers were indicated at the top of each bar. BLASTP search was performed against the complete peptide sequences of *Setaria italica* and the first 5 matches with E-value <1e-5 were identified as the PPR genes present on chromosomal segments duplication which were connected by black lines. The red boxes represent the PPR gene too close to connect by lines. The position (bp) each PPR protein gene on PHYTOZOME chromosome pseudomolecules was given in the Additional file 3: Table S3

The structures of PPR genes were determined by analyzing their exon-intron organization. The great majority of the foxtail millet PPR genes contained very few introns, as is the case with the vast majority of the Selaginella PPR genes. Figure 2 details the ratios of PPR genes in two species that contain no intron, 1 intron, 2–5 introns, and 6 or more introns. Approximately 80 % of the *Arabidopsis* and rice PPR genes lack introns [8, 10, 15]. Intron-rich PPR genes may represent“ancient” PPR genes previous to the occurrence of retrotransposition-mediated expansion of the PPR gene family in land plants [8, 10]. 79.94 % (1335 of 1671) and 78.60 % (382 of 486) of PPR genes of, respectively, Selaginella and foxtail millet, were predicted to lack introns. The 486 PPR genes are unevenly distributed on all 9 of the foxtail millet chromosomes. Chromosome 9 of foxtail millet possesses the highest number of SiPPRs [99 (20.49 %)], while the lowest number of SiPPRs were found on chromosome 8 [26 (5.34 %)]. For fine mapping, the exact position (in bp) of each SiPPR on the respective foxtail millet chromosome was obtained from Phytozome and is indicated diagrammatically in Fig. 3 (the exact position in bp is given in Additional file 1: Table S1). Substantial clustering of PPR genes was evident on all of the chromosomes. In total, 180 (~36.96 %) PPR genes were arranged in tandem repeats of two to seven genes, either in the same or the inverse direction (the distance between these genes being less than 100 kb), representing localized gene (tandem) duplications.

Comparing the numbers and structures of the PPR genes in all 5 species, including moss, Selaginella, *Arabidopsis*, rice, and foxtail millet, the numbers of Selaginella PPR genes were approximately three times as many as the other vascular plants. However, all homologous PPR genes in flowering plants also could be found in Selaginella, and the intron-rich PPR genes show similar structure in all 5 species, which suggested the PPR gene family expanded before the divergence of the euphyllophytes (ferns and seed plants) and the lycophytes (~400 Mya). The PPR genes may have been derived from a primitive and intron-rich “ancient” PPR gene by retrotransposition [7, 8, 10, 24]. Further, we speculate that some PPR genes may be non-functional, or may even have negatively affected the transition of non-flowering to flowering in vascular plants. The expansion of the PPR gene family may be coupled with organelle evolution.

**Fig. 4:**
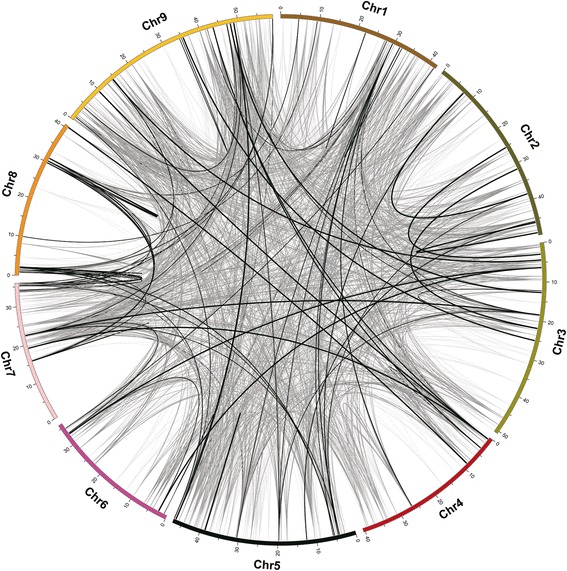
Distribution of segmentally duplicated PPR genes on foxtail millet chromosomes. Grey lines indicate collinear blocks in whole foxtail millet genome, and black lines indicate duplicated PPR gene pairs

6688 (~19 %) of the genes of the foxtail millet genome were segmentally duplicated [26]. Among the SiPPR genes, 190 (95 pairs; ~39.01 %) were segmentally duplicated (connected by black lines in Fig. 4); note that this proportion is far higher than that of genes generally. In particular, there were several genes that were duplicated a dramatic number of times, such as the gene Si020204m (located on chromosome 1), which was duplicated eleven times, forming 66 paralogs that can be found on all 9 of the foxtail millet chromosomes (Fig. 4). These results showed that genes segmental duplications were important in the expansion of the PPR gene family in vascular plants.

### Gene ontology annotation

**Fig. 5:**
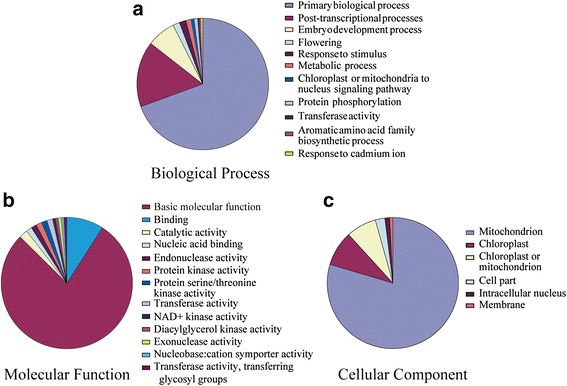
Gene Ontology (GO) distributions for the SiPPR protein. The Blast2Go program defined the gene ontology under three categories, (**a**) biological processes, (**b**) molecular functions and (**c**) cellular component

Blast2GO software performed the GO slim analysis, which suggested the putative participation of SiPPRs in various biological processes, molecular functions and cellular components (Fig. 5; Additional file 3: Table S3). Out of the 486 SiPPR proteins, GO annotation of 243 sequences could not be found, and the remaining 243 SiPPRs were divided into 9 separate categories of biological processes. The results indicated that a majority of SiPPRs were likely related to primary biological process [168 (~69.13 %)], followed by post-transcriptional processes within mitochondria and chloroplasts [39 (~16.04 %)] (including RNA editing, RNA splicing, RNA cleavage and translation), and embryo development processes [17 (~7 %)]. Of note, 4 SiPPRs were predicted to participate in responses to stress, a topic that we will address later in this text. The molecular functions of 112 (~46.09 %) SiPPRs were predicted to participate in molecular function, such as basic catalysis or binding function. The second most frequently annotated molecular function was nucleic acid binding function [10 (~4.11 %), which is in agreement with the molecular role of PPRs in binding RNA [17, 27–30]. Cellular localization prediction suggested that 203 (~83.53 %) SiPPR proteins were localized in the mitochondria, 22 (~9.05 %) were localized to chloroplasts, and 19 (7.1 %) SiPPRs were not sure localized in mitochondria or chloroplasts, 6 were localized in cytoplasm, 3 were localized in the cell nucleus, and 2 were localized in cell membranes (Fig. 5; Additional file 3: Table S3). The GO analysis results indicating that the SiPPR proteins participate in diverse biological processes, molecular functions and cellular components and will be useful for further verifications of gene functions in foxtail millet.

### Orthologous relationships

We explored the SiPPRs orthologous relationships by using a comparative mapping-based approach in which the physically mapped foxtail millet PPR genes were compared with those PPR genes in the chromosomes of other related grass genomes, including *B. distachyon*, rice, sorghum, and maize (Table 1; Additional file 4: Table S4 and Additional file 5: Figure S1). The specific orthologous relationships could be deduced on a mean value for ~ 65.46 % proteins, Maximal orthology of SiPPRs annotated on the *Setaria italica* chromosomes was found in *B. distachyon* (74.67 %), followed by rice (66.84 %), sorghum (66.36 %), and maize (54.01 %). The widespread synteny among *Setaria italica*, *B. distachyon*, rice, sorghum, and maize at gene level supports their close evolutionary relationships [1, 31–35]. Of note, most of the SiPPR genes had syntenic tend to special chromosomes of the four species, a finding consistent with what is known about the evolution of the *Setaria italica* genome [1, 33]. The two chromosome reshuffling events that occurred in foxtail millet, rice, and sorghum were reported [1]. Our results also suggested there may be several chromosome reshuffling events between foxtail millet and *B. distachyon* and maize, such as fusing *B. distachyon* chromosomes 1 and 4, 3 and 9 and 2 and 4 to foxtail millet chromosomes 2, 9 and 3, respectively, and maize chromosomes 4 and 5, 2 and 7 and 6 and 9 to foxtail millet chromosomes 1, 2 and 4, respectively. The comparative mapping information thus offers a helpful introduction for comprehending the evolution of PPR genes among those grasses, even the genome evolution of *Setaria italica*. In addition, this research would be helpful for the selection of candidate SiPPR genes and exploit them in genetic improvement of related grass family members. As for example, *Arabidopsis* PPR40 as a signaling link factor for the communication of mitochondrial electron transport and regulation of stress and hormonal responses. Insertion mutations inactivating *PPR40* affected plant growth and enhanced mutants sensitivity to salt, ABA, and oxidative stress [19]. Therefore, its orthologous *Setaria italica* gene (Phytozome ID: Si019461m) and rice gene (LOC_Os12g37100.1) may also have the similar function.

**Table 1:** A summary of comparative mapping of foxtail millet SiPPR genes on *Brachypodium sylvaticum*, sorghum, maize and rice

*Setaria italica*	*Brachypodium distachyon*	*Oryza sativa*	*Sorghum bicolor*	*Zea mays*
Chr1	Chr3(77.96 %)	Chr2(77.96 %)	Chr4(74.58 %)	Chr4(16.94 %),Chr5(52.54 %)
Chr2	Chr1(61.40 %),Chr4(24.56 %)	Chr7(57.89 %),Chr9(21.05 %)	Chr2(84.21 %)	Chr2(28.07 %),Chr7(49.12 %)
Chr3	Chr2(52.08 %),Chr4(25 %)	Chr5(47.91 %),Chr12(20.83 %)	Chr9(41.67 %)	Chr6(33.33 %)
Chr4	Chr1(76.19 %)	Chr6(59.52 %)	Chr10(71.42 %)	Chr6(26.19 %),Chr9(30.95 %)
Chr5	Chr2(78.2)	Chr1(73.07 %)	Chr3(70.51 %)	Chr3(39.74 %),Chr8(30.76 %)
Chr6	Chr3(85.18 %)	Chr8(74.07 %)	Chr7(70.37 %)	Chr1(25.92 %),Chr4(25.92 %)
Chr7	Chr5(47.82 %)	Chr4(50 %)	Chr6(50 %)	Chr2(36.95 %)
Chr8	Chr4(57.69)	Chr11(42.30 %)	Chr5(61.53 %)	Chr4(34.61 %)
Chr9	Chr9(60 %),Chr3(26 %)	Chr3(53 %),Chr10(24 %)	Chr1(73 %)	Chr1(55.01 %)

The percentages indicated among parentheses indicate the fraction of loci with conserved synthenic relationships

### Duplication and divergence rates of the SiPPR genes

**Fig. 6:**
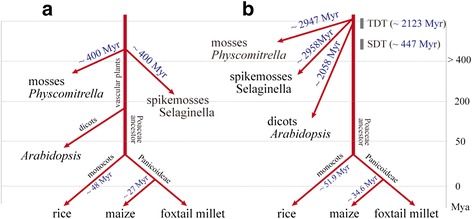
Diagram showing approximate times of duplication and divergence of the plants in this study. (**a**) Evolutionary relationships and approximate times of divergence of the six species in land plants [1, 75]. (**b**) The time of duplication and divergence of PPR genes in foxtail millet and the orthologous gene-pairs of SiPPR genes with those of other species: moss, Selaginella, *Arabidopsis*, rice and maize. Tandem duplication time (TDT) and segmental duplication time (SDT) of SiPPR genes were estimated to have occurred around 2123.51 Mya and 447.23 Mya, respectively

Gene duplications enable the evolution of genes with novel functions. Functional deficiency phenotypes occur less frequently when multiple copies of a gene are present then when there is only a single copy of a gene. Genes multiple copies may evolve new gene owing to evolutionary events such as the whole genome location (tandem) and segmental duplications. Such gene duplication has been described in previous reports [26, 36, 37]. Therefore, we searched that the effect of Darwinian positive selection in the duplication and divergence of the PPR genes. The synonymous (Ks) and non-synonymous (Ka) nucleotide substitution rates per site were determined for 52 location and 28 segmental duplication gene-pairs and between orthologous gene-pairs of SiPPR with those genes of moss (28 pairs), Selaginella (28 pairs), *Arabidopsis* (28 pairs), rice (28 pairs), and maize (28 pairs) (Fig. 6; Additional file 6: Table S5, Additional file 7: Table S6, Additional file 8: Table S7, Additional file 9: Table S8, Additional file 10: Table S9, Additional file 11: Table S10 and Additional file 12: Table S11). The non-synonymous/ synonymous ratio (Ka/Ks or ω) is a measure of natural selection acting on a protein, in which values of ω < 1 indicate negative purifying selection; ω = 1 indicates neutral evolution; and ω > 1 indicates positive diversifying selection. The mean number of synonymous substitutions (Ks) across all tandemly duplicated gene pairs was 21.69 and segmentally duplicated gene pairs were 5.81. The mean number of non-synonymous substitutions (Ka) across all tandemly duplicated gene pairs was 0.9662, and that for segmentally duplicated gene pairs was 0.29662. Even though the ω values extensive changes among gene pair groups, the average value was equal to 0.16631 and 0.37643 for tandemly duplicated gene pairs and segmentally duplicated gene pairs, respectively, suggesting that those duplicated SiPPRs were under very strong selection pressure, which had undergone substitution elimination and enormous selective constraint by natural selection during the course of evolution since ω < 1. Our analysis indicated that the duplication events leading to these location and segmental duplication SiPPRs may have happened approximately 2123.51 Mya and 447.23 Mya (Fig. 6), respectively. Presumably, the PPR family is a quite ancient gene family and may have existed as early as in the era of bacteria and algae (3500–1500 Mya), and PPRs were also found in ancient heterokont species (brown algae, diatoms) and bacterial species [7, 38]. However, there were no eukaryotes in the world at that time, the PPR genes family may have derived from *Bacteria* and/or *algae*, a supposition that is supported by the hypothesis of the endosymbiosis of a green algae into an ancestral host cell [39, 40]. Secondly, tandem duplication enabled the evolution of new functions among the PPR genes in evolutionarily diverse taxa (2157 Mya). The result of comparing the numbers and nature of the PPR genes in all 5 species indicated that the PPR gene family expanded before the divergence of the euphyllophytes (ferns and seed plants) and the lycophytes (~400 Mya), and segmental duplication of SiPPR genes occurred around 447.23 Mya, which suggests that genome segmental duplications may be responsible for the PPR gene expansion in vascular plant (Additional file 7: Tables S6).

Among the SiPPR orthologous gene-pairs with those of other four species, moss, Selaginella, *Arabidopsis*, rice, and maize, the value of ω was maximum between rice and foxtail millet (0.23), maize and foxtail millet (0.27), and least for moss and foxtail millet (0.032), Selaginella and foxtail millet (0.026), *Arabidopsis* and foxtail millet (0.046) (Additional file 8: Table S7, Additional file 9: Table S8, Additional file 10: Table S9, Additional file 11: Table S10 and Additional file 12: Table S11). The relatively higher ω between the SiPPR genes of maize and foxtail millet and rice and foxtail millet show their earlier divergence around 34.6 Mya and 51.9 Mya, the divergence time is in agreement with the recent time of divergence of maize-foxtail millet(~27 Mya) and rice-foxtail millet (~48 Mya, Fig. 6) [1]. However, the divergence time of moss-foxtail millet(~2947.9 Mya), Selaginella-foxtail millet (~2957.9 Mya) and *Arabidopsis*-foxtail millet (~2058.7 Mya) were far earlier than the time of divergence of monocots and dicots, which suggested the PPR gene family may share some common “ancient” PPR genes in land plants .

The estimation of tandem duplication time (~2123.51 Mya) of foxtail millet PPR genes is later than the divergence time of moss-foxtail millet (~2947.9 Mya), Selaginella-foxtail millet (~2957.9 Mya), and earlier than the divergence time of *Arabidopsis*-foxtail millet (~2058.7 Mya), maize-foxtail millet (~34.6 Mya) and rice-foxtail millet (~51.9 Mya). Segmental duplication time (~447.23 Mya) of foxtail millet PPR genes is later than the divergence time of moss-foxtail millet (~2947.9 Mya), Selaginella-foxtail millet (~2957.9 Mya), and *Arabidopsis*-foxtail millet (~2058.7 Mya), but earlier than maize-foxtail millet (~34.6 Mya) and rice-foxtail millet (~51.9 Mya). Remarkably, the orthologous PPR gene-pairs between moss-foxtail millet (0.032), Selaginella-foxtail millet (0.026), and *Arabidopsis*-foxtail millet (0.046) appear to have undergone extensive intense purifying selection, as compared to average ω values of the foxtail millet-rice (ω = 0.23) and foxtail millet-maize (ω = 0.27) PPR gene pairs. Consequently, we can draw a conclusion that the tandemly and segmentally duplicated events, including the divergence events of the PPRs from other species, have played a critical role in the evolution process of this gene family in foxtail millet, and genome segmental duplications may be responsible for the PPR gene expansion in vascular plant.

### Functional conservation of PPR proteins in foxtail millet

Most functional analyses of PPR proteins undertaken to date have only tested proteins from *Arabidopsis*, rice, or maize, and there is as yet very limited information regarding the evolutionary relationships between PPR proteins within or across species. The PPR gene family existed in the era of bacteria and green algae, and PPR proteins are mostly aimed at mitochondria or plastids in terrestrial plants [8]. It has been suggested that the origin and evolution of the PPR gene family may be inextricably coupled with the endosymbiosis of mitochondria and chloroplasts in the early evolutionary history of eukaryotes and plants [7, 8].

**Fig. 7:**
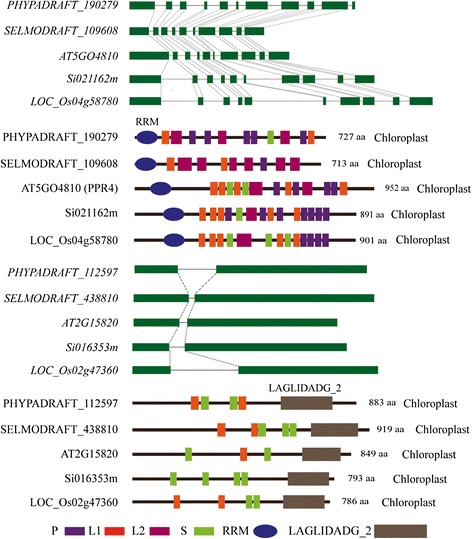
The PPRs contain RRM and LAGLIDADG domain were selected to example the intron conservation in the homologous PPR genes. In each figure, the fist panel shows the gene structure and the second panel shows the motif structure, protein size, and target location of the predicted PPR proteins

PPR proteins can be classified based on motif structure(s) and the presence of additional C-terminal domains. Subgroupings include the P and PLS subfamilies and the P-class PPR proteins presented the presence of one or more known/unknown functional motifs or domains, such as an RNA recognition motif (RRM) [41] and LAGLIDADG domain [42]. Those PPRs with the same domain always are intron rich (1 to 26) and tended to be located in the same organelles in moss, Selaginella, *Arabidopsis*, rice and foxtail millet (Additional file 13: Table S12), respectively. Comparison of the gene structures and amino acid sequences encoded by PPR proteins containing the same domains showed that these were well conserved among the five species (Fig. 7), indicating that these attributes had been preserved during evolution. This suggested that these homologous PPRs likely share the same or similar origins and functions, and further suggest that these the intron-rich PPR genes of moss may represent “ancient” PPR genes previous to the occurrence of retrotransposition-mediated [10, 24] or/and segmental duplication-mediated the PPR family expansion in terrestrial plants. Such conservation was likely very important for the survival and development of the chloroplast and mitochondria in these 5 species, perhaps even in all land plants. For instance, the PPR-SMR motif containing proteins of *Arabidopsis* (pTAC2, SVR7, and AT5G46580) and the corresponding maize orthologs (Zm-pTAC2, ATP4, and PPR53) were found to be localized to the chloroplast and to have the same or similar functions [43–46]. The *Arabidopsis* protein (OTP51) contains two LAGLIDADG motifs that are known to specifically promote the splicing of introns, and loss of AtOTP51 affected the assembly of the PSI complex [47]. The rice protein (OsOTP51) was found to have a similar function with AtOTP51, and loss-of-function OsOTP51 affected the intron splicing of a number of plastid genes, indirectly affected the structure and functions of PSI, and resulted in severe photoinhibition and eventual plant death, even when mutant plants were grown under very low light [13]. This example of conservation suggests that such homologous PPR proteins are important for the growth and development of plants and they preserved their ancient functions.

**Fig. 8:**
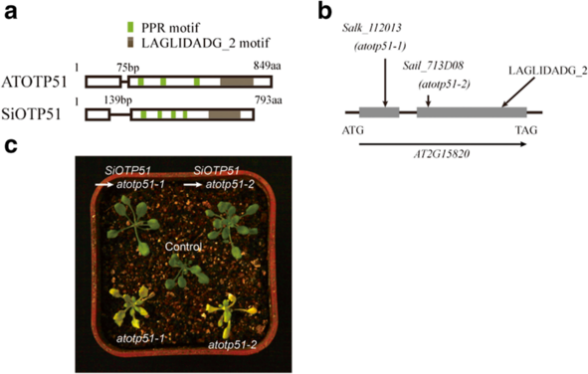
The phenotype of *Arabidopsis* seeding at two weeks old under low light conditions, which transformed *SiOTP51* into *atotp51-1* and *atotp51-2* mutants. (**a**) Schematic representation of ATOTP51 and SiOTP51 showed the gene or protein structure. Boxes and lines indicate exons and introns, respectively. (**b**) Positions of the two T-DNA insertions in the *atotp51* gene. *Salk_112013* was named *atotp51-1* and *Sail_713D08* was named *atotp51-2*. (**c**) Phenotype of two *atotp51* mutant seedling and reverse mutation of *atotp51* mutants with *SiOTP51* gene in low light conditions at two weeks old

In order to investigate the functional conservation of the PPR proteins in foxtail millet, we examined the presence of PPR-LAGLIDADG proteins in a broad range of species to explore their origins and diversification. The PPR-LAGLIDADG proteins were essentially only found in eukaryotic species and were largely limited to the Viridiplantae clade. We then investigated the function of SiOTP51. It contains a PPR motif and an LAGLIDADG_2 motif. We transformed *SiOTP51* into *Arabidopsis atotp51-1* and *atotp51-2* mutants using a plasmid containing *SiOTP51* under the control of the 35S cauliflower mosaic virus promoter*.* Homozygous *atotp51-1* and *atotp51-2* mutants exhibited identical phenotypes: seedlings were light straw yellow; rosettes were light yellow in standard light and pale green for young leaves in low light conditions; plants had retarded growth and greening [47] (Fig. 8). Complementation mutants of *atotp51* with the *Arabidopsis AtOTP51* gene ortholog *SiOTP51* showed that *SiOTP51* would to a certain extent reverse the mutant phenotypes. This would suggested that *SiOTP51* may also affect photosynthesis and plastid gene expression. The molecular functions of *OTP51s* in all land plants may be conserved.

### Expression of SiPPR genes under abiotic stress conditions and abscisic acid (ABA) treatment

**Fig. 9:**
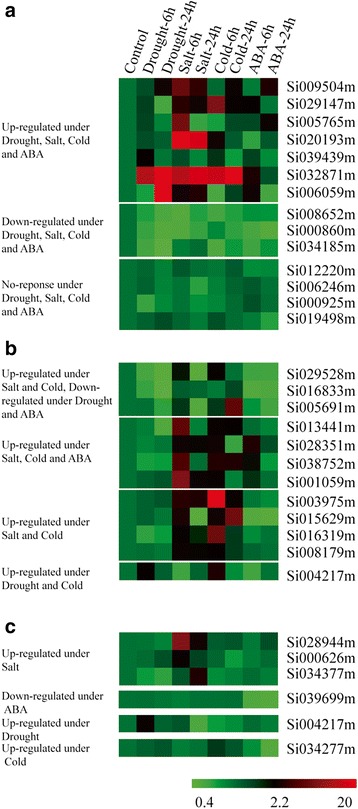
Expression profiles of foxtail millet PPR genes differentially expressed under abiotic stress conditions and abscisic acid (ABA) treatment. (**a**) Expression profiles of foxtail millet PPR genes differentially expressed under all four, (**b**) any three or two, (**c**) and specific stress condition(s) as compared to the control seedlings were presented. The values of SiPPR genes under control and various stress conditions (mentioned at the top of each lane) were presented by cluster display (values were given in Additional file 15: Table S13). The color scale (representing signal values) was shown at the bottom

The exposure of plants to changes in environment conditions and the treatment of plants with phytohormones is known to elicite various physiological, biochemical, and molecular responses, causing alterations in gene expression [36]. Some PPRs have been proved to function in post-transcriptional and post-translational processes and have been classified as sequence-specific RNA-binding proteins [7, 8, 48]. However, the function of the vast majority of PPR proteins remain to be characterized, and there is very limited information about this family in higher plants. This dearth of information is especially pronounced regarding the potential function of these proteins in plant responses to abiotic stresses and phytohormone treatments. To explore the potential functions of PPR proteins in stress and/or phytohormone responses in higher plants, the expression of the foxtail millet PPR genes was analyzed in plants that had been grown under drought treatment (20 % PEG 6000). We performed an RNA-seq experiment of total RNA isolated from 4-week-old foxtail millet seedlings, including leaves and roots (Beijing Genomics Institute, BGI). 51 PPR genes were identified which were differentially expressed (≥2-fold difference, p value less than 0.05) between plants grown under drought stress conditions and control plants. A heat map was got according to the RPKM values for the 51 genes. 41 genes were up-regulated; 10 genes were down-regulated genes (Additional file 14: Figure S2 and Additional file 15: Table S13). Then we examined the expression patterns of 31 PPR genes, including 21 PPR genes that were up-regulated expression, 6 PPR genes were down-regulated expression, and 4 selected from GO annotation (Additional file 16: Table S14), using qRT-PCR analysis of samples from plants, including leaves and roots, subjected to drought, salt, cold, and ABA treatment. A heat map illustration of expression patterns of 31 selected SiPPR genes is exhibited in Fig. 9. The qRT-PCR results of most candidate genes under abiotic stress condition (s) were agreement with the RNA-seq experiment under drought treated (Fig. 9). Seven of these genes were up-regulated and three were down-regulated; four showed no-response under any of the treatments (Fig. 9a). Three were up-regulated under salt and cold, but down-regulated under drought and ABA; Four were up-regulated under salt, cold, and ABA, but no response under drought stress; four PPR protein-encoding genes were up-regulated under salt and cold stresses, but no response under drought stress and ABA; one showed up-regulation under drought stress and cold, but no response under salt and ABA (Fig. 9b). Three were up-regulated under salt treatment, one was down-regulated under ABA, one was up-regulated under drought stress, and one was up-regulated under cold stress (Fig. 9c).

Of the 450 predicted PPR proteins in *Arabidopsis*, only five mitochondrion-localized proteins and one cytosol-nucleus dual-localized PPR protein were proved to function in response to abiotic stresses and/or ABA. *Arabidopsis* PPR40-mediated ubiqinol-cytochrome *c* oxidoreductase activity in mitochondrion complex III and it was related to oxidative respiration that also conducive to abiotic stress tolerance in *Arabidopsis* [19]. ABO5 was required for *cis*-splicing of the mitochondrial *nad2* intron 3 (*nad2* is one subunit in mitochondrion complex I) and altered the expression of several stress-responsive and nuclear-encoded genes to affect the ABA signaling pathway [11]. The PGN can regulate the ROS homeostasis in plants mitochondria during abiotic and biotic stress responses may occur through the regulation of mitochondria-nucleus retrograde signaling. AHG11 regulates the *nad4* (mitochondrion complex I) transcriptional level and thus led to changes in oxidative levels by controlling RNA editing events in plants mitochondria and affecting plant responses to ABA [21]. SLG1 regulated the *nad3* (mitochondrion complex I) transcript by regulating RNA editing events in mitochondria and affecting the expression of genes which were involved in the alternative respiratory pathway [22]. SOAR1 (cytosol-nucleus dual-localized) was identified recently as a crucial regulator in the CHLH/ABAR (Mg-chelatase H subunit/putative ABA receptor)-mediated signaling pathway that acts downstream of CHLH/ABAR and upstream of a nuclear ABA-responsive bZIP transcription factor, ABI5 [18]. qRT-PCR results showed that 11 SiPPRs were up-regulated under any three abiotic stress treatment (Fig. 9). These SiPPRs may have a similar stress response mechanism with *Arabisopsis* PPRs [11, 18–22]. In addition, the class of PPR proteins that can edit RNA are of the PLS subfamily, and the E+ motif is required for the conversion of C to U RNA editing [49]. Consistent with their functions, six stress-related PPR proteins, including PGN, AHG1, and SLG1, belonged to the E subgroup, and PPR40, ABO5, and SOAR1 belonged to the P subgroup. It has been suggested that the mitochondrial localization of P subgroup or E subgroup PPR proteins may participate in plant responses to biotic and abiotic stress.

### Subcellular localization of foxtail millet PPR proteins

**Fig. 10:**
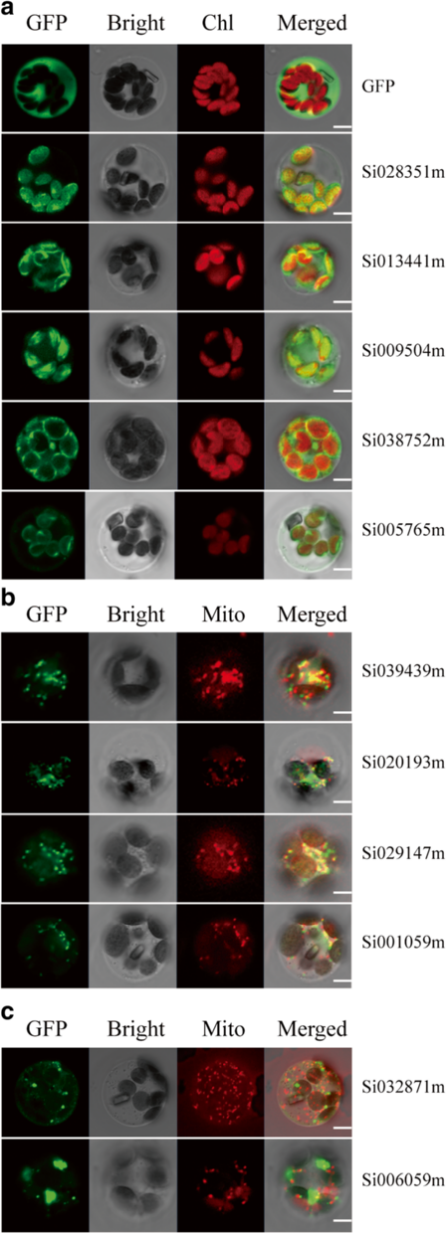
Subcellular localization of SiPPR protein. Fluorescence signals were visualized with 488 and 543 nm illumination using a Zeiss LSM700 confocal laser-scanning microscopy. Green fluorescence showed GFP, red fluorescence indicates chloroplast autofluorescence or stained protoplasts used mitotracker orange and yellow fluorescence indicated images with the two types of fluorescence merged. (**a**) Empty GFP vector without a specific targeting sequence and chloroplasts location of the SiPPR-GFP fusion protein. (**b**) Mitochondria location of the SiPPR-GFP fusion protein, red fluorescence indicated mitochondria. (**c**) Green fluorescence shows the proteins located to other organelles, red fluorescence indicated mitochondria. Bars = 5 mm. (**a**) to (**c**) were foxtail millet protoplasts

Based on the expression of SiPPR genes in plants grown under abiotic stress conditions, 11 candidates PPR proteins were selected for verification of the subcellular localization, including 7 up-regulated under any of the treatments, and 4 up-regulated under salt, cold, and ABA. Among these 11, 4 proteins were predicted by TargetP to be localized to chloroplasts, 6 proteins were predicted to be targeted to mitochondria, and 1 protein was predicted to be localized to cytoplasm [50]. Those foxtail millet PPR genes were cloned and inserted into a subcellular localization vector that included a GFP protein-encoding gene under the control of the 35S promoter; these vectors were transformed into foxtail millet protoplasts. To confirm that the putative mitochondrion-targeted PPR proteins were expressed in the mitochondria, we stained transformed foxtail millet protoplasts with a mitochondria-specific dye [51], and then observed the samples with 488 and 543 nm illumination. 5 of these proteins were localized to chloroplasts and 4 were localized to mitochondria and 2 located to the cytoplasm (Fig. 10). Among 11 SiPPR genes, 6 belong to the P subfamily and 5 belong to the PLS subfamily. Most of PPRs located to chloroplasts or mitochondria were consistent with the results predicted by TargetP. However, Si032871m and Si006059m, which were predicted to be localized to mitochondria, were localized to the cytoplasm. PPR proteins involved in abiotic stresses and ABA were localized to the cytoplasm and/or mitochondria [11, 18–22]. These results suggest that the 4 mitochondrion-localized genes and the 2 cytoplasm-localized SiPPR proteins may be viewed as candidates implicated in plant responses to adverse environmental conditions and exogenous phytohormones.

Chloroplasts and mitochondria are the two main plant cellular organelles with independent genomes. Under unfavorable conditions, the accumulation of ROS in both chloroplasts and mitochondria increases oxidative stress and this acts as a signal to help the plant respond to unfavorable environmental conditions. It is known that PPR proteins are mostly aimed at plastids and mitochondria in plants, and the mitochondrial/chloroplast PPR proteins play various and crucial roles in plant developmental processes and responses to environmental stresses [6, 8, 16, 52–61]. However, there is not much information about the chloroplast localization of PPR proteins in response to abiotic stresses. To date, the only reported chloroplast-localized PPR protein, GUN1, was found to be a central regulator of plastid to nucleus retrograde signaling, and its impairment led to ABI4-mediated repression of nuclear-encoded genes and enhanced sensitivity to ABA [57, 62]. The subcellular localization assays of 11 foxtail millet PPR proteins in our study revealed that 5 members were located to chloroplasts, and these candidate proteins seem to be responsive to diverse abiotic stresses (Figs. 9 and 10).

## Conclusions

Pentatricopeptide repeat (PPR) proteins are extensive in terrestrial plants, and the genomes of terrestrial plants typically include several hundred genes encoding these proteins. Most of PPRs are targeted to plastids and mitochondria, where they play crucial roles in mechanisms controlling post-transcriptional processes. However, functional studies of PPRs in terrestrial plants remain scarce. This study provides evidence that the period of the expansion of the PPR gene family occurred prior to the divergence of the euphyllophytes (ferns and seed plants) and the lycophytes (~400 Mya). Our results also suggest that genome segmental duplications were responsible for the expansion of the PPR gene family in vascular plants. In addition, we conducted a comprehensive analysis of the SiPPR genes/proteins that included classification, chromosomal location, orthologous relationships, duplication analysis, and auxiliary motifs. We conducted expression pattern analysis of 31 candidate SiPPR genes in plants grown under stress conditions, and performed subcellular localization analysis of 11 PPR proteins. Our study establishes an empirical foundation for further investigations seeking to elucidate the precise role of individual PPRs in foxtail millet.

## Methods

### Database searching

The Hidden Markov Model (HMM) profile of PPR motif (PF01535) was acquired from the Pfam v28.0 database (http://Pfam.sanger.ac.uk/), and was queried against the HMMER (http://www.ebi.ac.uk/Tools/hmmer/) of foxtail millet, with a threshold E-value ≤ 10. Ultimately, 486 and 1670 nonredundant PPR proteins in foxtail millet and lycophyte Selaginella, respectively, displayed the presence of PPR motif with confidence (E-value less than 1.0) in PFam searches, and SMART (http://smart.embl-heidelberg.de/). The sequence data of the PPR genes and the gene annotations used in this study were for moss, *Arabidopsis*, and rice as obtained according to the previous description [8, 10]. The hmmsearch program from the HMMER package (Eddy 1998) was applied to the translated sequence data to identify clusters of all of the PPR motifs (P, L, S, L2, E/E+, and DYW).

### Physical locations, gene structure, and genomic distribution

All the sequenced contigs of foxtail millet have been physically constructed as pseudo molecules at Phytozome v10 (http://phytozome.jgi.doe.gov/pz/portal.html). Subsequently, each of the PPRs were positioned on the nine foxtail millet chromosome pseudo molecules based on their ascending order of physical position (bp) according to BLASTN searching against the Phytozome database adopting default settings. The analytical tools available at the Plant Genome Duplication Database were used to identify segmentally duplicated PPR gene in foxtail millet [63], and detailed methods according to the previous description [64, 65]. Segmental duplications were drew using Circos 0.67 (http://circos.ca) [66]. Tandem duplications were characterized according to the previous description [65]. Manual curation and assessment of the numbers of introns and exon-intron positioning of the genes was based on comparison of the full-length cDNA or the predicted coding sequence (CDS) of SiPPR genes with their corresponding genomic sequence.

### Gene ontology (GO) annotation

Blast2GO (http://www.blast2go.com) software was used to perform the functional annotation of the SiPPR gene sequences and the subsequent analysis of annotation results, and detailed methods according to the previous description [67].

### Comparative physical mapping of PPRs between foxtail millet and other grass species

The comparative orthologous relationships of the PPR proteins between foxtail millet and other grass species were performed according to the previous description by Mishra et al. (2014) and Lata et al. (2014) [26, 37], and visualized by using Circos 0.67 [66].

### Estimation of duplication and divergence rates

The corresponding amino acids and their cDNA sequences of paralogous and orthologous gene-pairs of the SiPPR proteins in moss, Selaginella, *Arabidopsis*, rice, and maize were aligned using a ClustalW-based multiple sequence alignment tool and analyzed using the tool of PAL2NAL (http://www.bork.embl.de/pal2nal/) [68]. Time (million years ago, Mya) of duplication and divergence of each SiPPR gene was calculated according to the previous description [69, 70].

### Functional conservation of PPR proteins in foxtail millet

To create the complementation construct pSiOTP51, the full-length opening reading frames of *SiOTP51* (*Si016353m*) was obtained from Yugu 1 cDNA with the primers 5’-ATCCAAGGTTTACTCCCTCCTCAGC-3’ and 5’-GTGAACATACACTGTCCTTACCCT-3’, and the resulting fragments were inserted into the plant binary vector pCAMBIA1302, in which the *SiOTP51* gene was under the control of the CaMV 35S promoter. The constructs were introduced into the *atotp51-1* and *atotp51-2* mutant by *Agrobacterium tumefaciens*-mediated transformation as described previously [71]. The *Arabidopsis* Columbia-0 (Col) T-DNA mutants and wild-type plants were germinated on ½ MS medium, with 2 % sucrose, at 22 °C, with a 16-h photoperiod and a light intensity of 50 μmol photons/m^2^. Seedlings were transferred to Nutrition Soil (Pindstrup Mosebrug A/S) with vermiculite (nutrition soil:vermiculite, 2:1) and grown with the same conditions described above.

### RNA-seq experiment of drought-treated foxtail millet

Isolation of foxtail millet seedings total RNA using TRIzol reagent (Invitrogen) and treated with RNase free DNase I (Takara) according to the manufacturer’s recommendations. Subsequently, RNA-seq experiment of drought-treated foxtail millet was completed by Beijing Genomics Institute, and detailed description according to the previous description [72].

### Plant materials and stress treatments

Seeds of foxtail millet Yugu 1 were grown in a growth chamber (MGC-350HP-2; Blue Leopard) at 28 ± 1 °C day/23 ± 1 °C night with 70 ± 5 % relative humidity and a photoperiod of 14 h. For stress treatments, 21-day-old seedlings, including leaves and roots, were exposed to drought (20 % PEG 6000), salt (250 mM NaCl), cold (4 °C), or 100 μM ABA treatment for 0, 6 and 24 h. Unstressed plants were maintained as controls. After the treatments, the seedlings were immediately frozen in liquid nitrogen and stored at −80 °C until RNA isolation. These experiments were repeated thrice.

### RNA extraction and qRT-PCR analysis

Total RNA from Yugu 1 was extracted with an RNA extraction kit (Takara) according to the manufacturer's instructions. The cDNA synthesis was conducted as previous description [73]. qRT-PCR for examination were carried out with TransScript® II Probe One-Step qRT-PCR SuperMix (TransGen Biotech) followed by amplification on an ABI 7500 System. The qRT-PCR primers used in this study are listed in Additional file 17: Table S15.

### Subcellular localization of foxtail millet PPR proteins

11 candidate SiPPR genes were amplified from the cDNA of Yugu 1 with the primers listed in Additional file 18: Table S16. These genes were inserted into the subcellular localization vector p16318, which contains the CaMV 35S promoter and a C-terminal green fluorescent protein (GFP) domain [73]. The transient expression assays were performed as previously described [73, 74]. Transfected foxtail millet protoplasts were stained with a mitochondria-specific dye (MitoTracker Orange [Invitrogen catalogue no. M7510]) [51] and then observed with 488 and 543 nm illumination using a Zeiss LSM700 microscope.

## Additional files

Additional file 1: Table S1. PPR genes in foxtail millet. Detailed genomic information, including domain/class present, ORF length, predicted protein length, genomic locus (chromosomal location), number of introns within the ORF, and the subcellular localization of the PPR proteins for each PPR gene. (DOCX 62 kb)
Additional file 2: Table S2. PPR genes in Selaginella. The domain/class, open reading frame length, predicted protein length, chromosomal location, number of introns within the ORF, and the subcellular localization of the PPR proteins of each of these genes are listed. (DOCX 194 kb)
Additional file 3: Table S3. Blast2Go annotation details of SiPPR protein sequences. (XLSX 28 kb)
Additional file 4: Table S4. The physically mapped PPR genes of foxtail millet were compared with those in the chromosomes of other related grass genomes, including *B. distachyon*, rice, sorghum, and maize. (XLSX 133 kb)
Additional file 5: Figure S1. Orthologous relationships of the PPR genes between foxtail millet and those of other grass species. (TIF 5719 kb)
Additional file 6: Table S5. The Ka/Ks ratios and the estimated divergence times for putatively tandemly duplicated SiPPR genes. (DOCX 20 kb)
Additional file 7: Table S6. The Ks/Ka ratios and the estimated divergence times for putatively segmentally duplicated SiPPR genes. Detailed segmental duplication information including domains present, chromosomal location, E-value, and % homology are provided for each putatively segmentally duplicated SiPPR gene. (DOCX 30 kb)
Additional file 8: Table S7. The Ka/Ks ratios and the estimated divergence times for orthologous SiPPR proteins between foxtail millet and moss. (XLSX 29 kb)
Additional file 9: Table S8. The Ka/Ks ratios and the estimated divergence times for orthologous SiPPR proteins between foxtail millet and Selaginella. (XLSX 38 kb)
Additional file 10: Table S9. The Ka/Ks ratios and the estimated divergence times for orthologous SiPPR proteins between foxtail millet and *Arabidopsis*. (XLSX 36 kb)
Additional file 11: Table S10. The Ka/Ks ratios and the estimated divergence times for orthologous SiPPR proteins between foxtail millet and rice. (XLSX 38 kb)
Additional file 12: Table S11. The Ka/Ks ratios and the estimated divergence times for orthologous SiPPR proteins between foxtail millet and maize. (XLSX 34 kb)
Additional file 13: Table S12. The PPRs with putative auxiliary motifs or domains in moss, Selaginella, *Arabidopsis*, foxtail millet, and rice. The domain/class, number of introns within the ORF, and the subcellular localization of the PPR proteins are provided for each PPR gene. (DOCX 29 kb)
Additional file 14: Figure S2. The heat map of the expression of 51 PPR genes in foxtail millet under drought treatedment (20 % PEG 6000) based on RPKM values. (TIF 163 kb)
Additional file 15: Table S13. RNA-seq analysis of PPR gene expression under drought stress treatment (20 % PEG 6000) in foxtail millet. (XLSX 15 kb)
Additional file 16: Table S14. PPR genes differentially expressed under drought treatment (20 % PEG 6000), salt treatment (250 mM NaCl), cold treatment (4 °C), and treatment with 100 μM ABA, at the 0, 6, and 24 h time points. (DOCX 19 kb)
Additional file 17: Table S15. Primer sequences used for real-time PCR analysis. (DOCX 15 kb)
Additional file 18: Table S16. Primer sequences used for the subcellular localization analysis of the PPR proteins in foxtail millet. (DOCX 14 kb)

## Abbreviations

ABA: Abscisic acid
GFP: Green fluorescent protein
GO: Gene ontology
Ka: The non-synonymous nucleotide substitution rates per site
Ks: The synonymous nucleotide substitution rates per site
Mya: Millions of years ago
PEG6000: Polyethylene glycol 6000
PPR: Pentatricopeptide repeat proteins
PSI: Photosynthetic complex I
qRT-PCR: Quantitative real-time PCR
RPKM: Reads per kilobase of exon model per million mapped reads
RRM: RNA recognition motif.

## References

[1] Zhang G, Liu X, Quan Z, Cheng S, Xu X, Pan S, Xie M, Zeng P, Yue Z, Wang W (2012). Genome sequence of foxtail millet (*Setaria italica*) provides insights into grass evolution and biofuel potential. Nat Biotechnol.

[2] Li P, Brutnell TP (2011). *Setaria viridis* and *Setaria italica*, model genetic systems for the *Panicoid* grasses. J Exp Bot.

[3] Lata C, Gupta S, Prasad M (2013). Foxtail millet: a model crop for genetic and genomic studies in bioenergy grasses. Crit Rev Biotechnol.

[4] Lata C, Prasad M (2013). *Setaria* genome sequencing: an overview. J Plant Biochem Biotechnol.

[5] Muthamilarasan M, Theriappan P, Prasad M (2013). Recent advances in crop genomics for ensuring food security. Curr Sci.

[6] Small ID, Peeters N (2000). The PPR motif–a TPR-related motif prevalent in plant organellar proteins. Trends Biochem Sci.

[7] Schmitz-Linneweber C, Small I (2008). Pentatricopeptide repeat proteins: a socket set for organelle gene expression. Trends Plant Sci.

[8] Lurin C, Andres C, Aubourg S, Bellaoui M, Bitton F, Bruyere C, Caboche M, Debast C, Gualberto J, Hoffmann B (2004). Genome-wide analysis of Arabidopsis pentatricopeptide repeat proteins reveals their essential role in organelle biogenesis. Plant Cell.

[9] Andrés C, Lurin C, Small ID (2007). The multifarious roles of PPR proteins in plant mitochondrial gene expression. Physiol Plant.

[10] O'Toole N, Hattori M, Andres C, Iida K, Lurin C, Schmitz-Linneweber C, Sugita M, Small I (2008). On the expansion of the pentatricopeptide repeat gene family in plants. Mol Biol Evol.

[11] Liu Y, He J, Chen Z, Ren X, Hong X, Gong Z (2010). *ABA overly-sensitive 5* (*ABO5*), encoding a pentatricopeptide repeat protein required for *cis*-splicing of mitochondrial *nad2* intron 3, is involved in the abscisic acid response in *Arabidopsis*. Plant J.

[12] Sung TY, Tseng CC, Hsieh MH (2010). The SLO1 PPR protein is required for RNA editing at multiple sites with similar upstream sequences in *Arabidopsis* mitochondria. Plant J.

[13] Ye JW, Gong ZY, Chen CG, Mi HL, Chen GY (2012). A mutation of *OSOTP 51* leads to impairment of photosystem I complex assembly and serious photo-damage in rice. J Integr Plant Biol.

[14] des Francs‐Small CC, Kroeger T, Zmudjak M, Ostersetzer‐Biran O, Rahimi N, Small I, Barkan A (2012). A PORR domain protein required for *rpl2* and *ccmFC* intron splicing and for the biogenesis of *c*‐type cytochromes in *Arabidopsis* mitochondria. Plant J.

[15] Rivals E, Bruyere C, Toffano-Nioche C, Lecharny A (2006). Formation of the Arabidopsis pentatricopeptide repeat family. Plant Physiol.

[16] Ding YH, Liu NY, Tang ZS, Liu J, Yang WC (2006). Arabidopsis GLUTAMINE-RICH PROTEIN23 is essential for early embryogenesis and encodes a novel nuclear PPR motif protein that interacts with RNA polymerase II subunit III. Plant Cell.

[17] Hammani K, Gobert A, Hleibieh K, Choulier L, Small I, Giegé P (2011). An Arabidopsis dual-localized pentatricopeptide repeat protein interacts with nuclear proteins involved in gene expression regulation. Plant Cell.

[18] Mei C, Jiang SC, Lu YF, Wu FQ, Yu YT, Liang S, Feng XJ, Portoles Comeras S, Lu K, Wu Z (2014). Arabidopsis pentatricopeptide repeat protein *SOAR1* plays a critical role in abscisic acid signalling. J Exp Bot.

[19] Zsigmond L, Rigó G, Szarka A, Székely G, Ötvös K, Darula Z, Medzihradszky KF, Koncz C, Koncz Z, Szabados L (2008). Arabidopsis PPR40 connects abiotic stress responses to mitochondrial electron transport. Plant Physiol.

[20] Laluk K, AbuQamar S, Mengiste T (2011). The Arabidopsis mitochondria-localized pentatricopeptide repeat protein PGN functions in defense against necrotrophic fungi and abiotic stress tolerance. Plant Physiol.

[21] Murayama M, Hayashi S, Nishimura N, Ishide M, Kobayashi K, Yagi Y, Asami T, Nakamura T, Shinozaki K, Hirayama T (2012). Isolation of *Arabidopsis ahg11*, a weak ABA hypersensitive mutant defective in *nad4* RNA editing. J Exp Bot.

[22] Yuan H, Liu D (2012). Functional disruption of the pentatricopeptide protein SLG1 affects mitochondrial RNA editing, plant development, and responses to abiotic stresses in *Arabidopsis*. Plant J.

[23] Rensing SA, Lang D, Zimmer AD, Terry A, Salamov A, Shapiro H, Nishiyama T, Perroud P-F, Lindquist EA, Kamisugi Y (2008). The *Physcomitrella* genome reveals evolutionary insights into the conquest of land by plants. Science.

[24] Sugita M, Ichinose M, Ide M, Sugita C (2013). Architecture of the PPR gene family in the moss *Physcomitrella patens*. RNA Biol.

[25] Banks JA, Nishiyama T, Hasebe M, Bowman JL, Gribskov M, Albert VA, Aono N, Aoyama T, Ambrose BA, Ashton NW (2011). The Selaginella genome identifies genetic changes associated with the evolution of vascular plants. Science.

[26] Mishra AK, Muthamilarasan M, Khan Y, Parida SK, Prasad M (2014). Genome-wide investigation and expression analyses of WD40 protein family in the model plant foxtail millet (*Setaria italica* L.). PLoS One.

[27] Lahmy S, Barnèche F, Derancourt J, Filipowicz W, Delseny M, Echeverria M (2000). A chloroplastic RNA-binding protein is a new member of the PPR family. FEBS Lett.

[28] Meierhoff K, Felder S, Nakamura T, Bechtold N, Schuster G (2003). HCF152, an Arabidopsis RNA binding pentatricopeptide repeat protein involved in the processing of chloroplast *psbB-psbT-psbH-petB-petD* RNAs. Plant Cell.

[29] Mili S, Piñol-Roma S (2003). LRP130, a pentatricopeptide motif protein with a noncanonical RNA-binding domain, is bound *in vivo* to mitochondrial and nuclear RNAs. Mol Cell Biol.

[30] Nakamura T, Meierhoff K, Westhoff P, Schuster G (2003). RNA-binding properties of HCF152, an *Arabidopsis* PPR protein involved in the processing of chloroplast RNA. Eur J Biochem.

[31] Bennetzen JL, Schmutz J, Wang H, Percifield R, Hawkins J, Pontaroli AC, Estep M, Feng L, Vaughn JN, Grimwood J (2012). Reference genome sequence of the model plant *Setaria*. Nat Biotechnol.

[32] Puranik S, Sahu PP, Mandal SN, Parida SK, Prasad M (2013). Comprehensive genome-wide survey, genomic constitution and expression profiling of the NAC transcription factor family in foxtail millet (*Setaria italica* L.). PLoS One.

[33] Devos KM (2010). Grass genome organization and evolution. Curr Opin Plant Biol.

[34] Salse J, Bolot S, Throude M, Jouffe V, Piegu B, Quraishi UM, Calcagno T, Cooke R, Delseny M, Feuillet C (2008). Identification and characterization of shared duplications between rice and wheat provide new insight into grass genome evolution. Plant Cell.

[35] Huo N, Vogel JP, Lazo GR, You FM, Ma Y, McMahon S, Dvorak J, Anderson OD, Luo MC, Gu YQ (2009). Structural characterization of *Brachypodium* genome and its syntenic relationship with rice and wheat. Plant Mol Biol.

[36] Jain M, Nijhawan A, Arora R, Agarwal P, Ray S, Sharma P, Kapoor S, Tyagi AK, Khurana JP (2007). F-box proteins in rice. Genome-wide analysis, classification, temporal and spatial gene expression during panicle and seed development, and regulation by light and abiotic stress. Plant Physiol.

[37] Lata C, Mishra AK, Muthamilarasan M, Bonthala VS, Khan Y, Prasad M (2014). Genome-Wide Investigation and Expression Profiling of *AP2/ERF* Transcription Factor Superfamily in Foxtail Millet (*Setaria italica* L.). PLoS One.

[38] Liu S, Melonek J, Boykin LM, Small I, Howell KA (2013). PPR-SMRs: ancient proteins with enigmatic functions. RNA Biol.

[39] Moustafa A, Beszteri B, Maier UG, Bowler C, Valentin K, Bhattacharya D (2009). Genomic footprints of a cryptic plastid endosymbiosis in diatoms. Science.

[40] Dorrell RG, Smith AG (2011). Do red and green make brown?: perspectives on plastid acquisitions within chromalveolates. Eukaryotic Cell.

[41] Burd CG, Dreyfuss G. Conserved structures and diversity of functions of RNA-binding proteins. Science. 1994;265(5172):615–620. 10.1126/science.8036511 8036511

[42] Dalgaard JZ, Klar AJ, Moser MJ, Holley WR, Chatterjee A, Mian IS (1997). Statistical modeling and analysis of the LAGLIDADG family of site-specific endonucleases and identification of an intein that encodes a site-specific endonuclease of the HNH family. Nucleic Acids Res.

[43] Ferro M, Brugière S, Salvi D, Seigneurin-Berny D, Moyet L, Ramus C, Miras S, Mellal M, Le Gall S, Kieffer-Jaquinod S (2010). AT_CHLORO, a comprehensive chloroplast proteome database with subplastidial localization and curated information on envelope proteins. Mol Cell Proteomics.

[44] Olinares PDB, Ponnala L, van Wijk KJ (2010). Megadalton complexes in the chloroplast stroma of *Arabidopsis thaliana* characterized by size exclusion chromatography, mass spectrometry, and hierarchical clustering. Mol Cell Proteomics.

[45] Majeran W, Friso G, Asakura Y, Qu X, Huang M, Ponnala L, Watkins KP, Barkan A, Van Wijk KJ (2012). Nucleoid-enriched proteomes in developing plastids and chloroplasts from maize leaves: a new conceptual framework for nucleoid functions. Plant Physiol.

[46] Huang M, Friso G, Nishimura K, Qu X, Olinares PDB, Majeran W, Sun Q, van Wijk KJ (2012). Construction of plastid reference proteomes for maize and *Arabidopsis* and evaluation of their orthologous relationships; the concept of orthoproteomics. J Proteome Res.

[47] de Longevialle AF, Hendrickson L, Taylor NL, Delannoy E, Lurin C, Badger M, Millar AH, Small I (2008). The pentatricopeptide repeat gene *OTP51* with two LAGLIDADG motifs is required for the *cis*-splicing of plastid *ycf3* intron 2 in *Arabidopsis thaliana*. Plant J.

[48] Delannoy E, Stanley W, Bond C, Small I (2007). Pentatricopeptide repeat (PPR) proteins as sequence-specificity factors in post-transcriptional processes in organelles. Biochem Soc Trans.

[49] Okuda K, Myouga F, Motohashi R, Shinozaki K, Shikanai T (2007). Conserved domain structure of pentatricopeptide repeat proteins involved in chloroplast RNA editing. Proc Natl Acad Sci.

[50] Emanuelsson O, Nielsen H, Brunak S, von Heijne G (2000). Predicting subcellular localization of proteins based on their N-terminal amino acid sequence. J Mol Biol.

[51] Jiang K, Schwarzer C, Lally E, Zhang S, Ruzin S, Machen T, Remington SJ, Feldman L (2006). Expression and characterization of a redox-sensing green fluorescent protein (reduction-oxidation-sensitive green fluorescent protein) in Arabidopsis. Plant Physiol.

[52] Oguchi T, Sage-Ono K, Kamada H, Ono M (2004). Genomic structure of a novel *Arabidopsis* clock-controlled gene, *AtC401*, which encodes a pentatricopeptide repeat protein. Gene.

[53] Tzafrir I, Pena-Muralla R, Dickerman A, Berg M, Rogers R, Hutchens S, Sweeney TC, McElver J, Aux G, Patton D (2004). Identification of genes required for embryo development in Arabidopsis. Plant Physiol.

[54] Cushing DA, Forsthoefel NR, Gestaut DR, Vernon DM (2005). Arabidopsis *emb175* and other *ppr* knockout mutants reveal essential roles for pentatricopeptide repeat (PPR) proteins in plant embryogenesis. Planta.

[55] Wang Z, Zou Y, Li X, Zhang Q, Chen L, Wu H, Su D, Chen Y, Guo J, Luo D (2006). Cytoplasmic male sterility of rice with boro II cytoplasm is caused by a cytotoxic peptide and is restored by two related PPR motif genes via distinct modes of mRNA silencing. Plant Cell.

[56] Gutiérrez-Marcos JF, Dal Prà M, Giulini A, Costa LM, Gavazzi G, Cordelier S, Sellam O, Tatout C, Paul W, Perez P (2007). empty pericarp4 encodes a mitochondrion-targeted pentatricopeptide repeat protein necessary for seed development and plant growth in maize. Plant Cell.

[57] Koussevitzky S, Nott A, Mockler TC, Hong F, Sachetto-Martins G, Surpin M, Lim J, Mittler R, Chory J (2007). Signals from chloroplasts converge to regulate nuclear gene expression. Science.

[58] Chi W, Ma J, Zhang D, Guo J, Chen F, Lu C, Zhang L (2008). The pentratricopeptide repeat protein DELAYED GREENING1 is involved in the regulation of early chloroplast development and chloroplast gene expression in Arabidopsis. Plant Physiol.

[59] Fujii S, Small I (2011). The evolution of RNA editing and pentatricopeptide repeat genes. New Phytol.

[60] Hu J, Wang K, Huang W, Liu G, Gao Y, Wang J, Huang Q, Ji Y, Qin X, Wan L (2012). The rice pentatricopeptide repeat protein RF5 restores fertility in Hong-Lian cytoplasmic male-sterile lines via a complex with the glycine-rich protein GRP162. Plant Cell.

[61] Nakamura T, Yagi Y, Kobayashi K (2012). Mechanistic insight into pentatricopeptide repeat proteins as sequence-specific RNA-binding proteins for organellar RNAs in plants. Plant Cell Physiol.

[62] Cottage A, Mott EK, Kempster JA, Gray JC (2010). The *Arabidopsis* plastid-signalling mutant *gun1* (genomes uncoupled1) shows altered sensitivity to sucrose and abscisic acid and alterations in early seedling development. J Exp Bot.

[63] Tang H, Bowers JE, Wang X, Ming R, Alam M, Paterson AH (2008). Synteny and collinearity in plant genomes. Science.

[64] Du D, Zhang Q, Cheng T, Pan H, Yang W, Sun L (2013). Genome-wide identification and analysis of late embryogenesis abundant (*LEA*) genes in *Prunus mume*. Mol Biol Rep.

[65] Shiu S-H, Bleecker AB (2003). Expansion of the receptor-like kinase/Pelle gene family and receptor-like proteins in Arabidopsis. Plant Physiol.

[66] Krzywinski M, Schein J, Birol I, Connors J, Gascoyne R, Horsman D, Jones SJ, Marra MA (2009). Circos: an information aesthetic for comparative genomics. Genome Res.

[67] Conesa A, Götz S (2008). Blast2GO: A comprehensive suite for functional analysis in plant genomics. Int J Plant Genomics.

[68] Suyama M, Torrents D, Bork P (2006). PAL2NAL: robust conversion of protein sequence alignments into the corresponding codon alignments. Nucleic Acids Res.

[69] Yang Z, Gu S, Wang X, Li W, Tang Z, Xu C (2008). Molecular evolution of the CPP-like gene family in plants: insights from comparative genomics of *Arabidopsis* and rice. J Mol Evol.

[70] Lynch M, Conery JS (2000). The evolutionary fate and consequences of duplicate genes. Science.

[71] Jeon JS, Lee S, Jung KH, Jun SH, Jeong DH, Lee J, Kim C, Jang S, Lee S, Yang K (2000). T-DNA insertional mutagenesis for functional genomics in rice. Plant J.

[72] He GH, Xu JY, Wang YX, Liu JM, Li PS, Chen M, Ma YZ, Xu ZS (2016). Drought-responsive WRKY transcription factor genes *TaWRKY1* and *TaWRKY33* from wheat confer drought and/or heat resistance in *Arabidopsis*. BMC Plant Biol.

[73] Xu Z-S, Xia L-Q, Chen M, Cheng X-G, Zhang R-Y, Li L-C, Zhao Y-X, Lu Y, Ni Z-Y, Liu L (2007). Isolation and molecular characterization of the *Triticum aestivum* L. ethylene-responsive factor 1 (*TaERF1*) that increases multiple stress tolerance. Plant Mol Biol.

[74] Liu P, Xu ZS, Pan-Pan L, Hu D, Chen M, Li LC, Ma YZ (2013). A wheat *PI4K* gene whose product possesses threonine autophophorylation activity confers tolerance to drought and salt in *Arabidopsis*. J Exp Bot.

[75] Nishiyama T, Fujita T, Shin IT, Seki M, Nishide H, Uchiyama I, Kamiya A, Carninci P, Hayashizaki Y, Shinozaki K (2003). Comparative genomics of *Physcomitrella patens* gametophytic transcriptome and *Arabidopsis thaliana*: implication for land plant evolution. Proc Natl Acad Sci U S A.

