# Supplementary material for: Genome-wide investigation and expression analyses of the pentatricopeptide repeat protein gene family in foxtail millet
Source: BMC Genomics. 2016 Oct 28;17:840. doi: 10.1186/s12864-016-3184-2 (PMC5084403; doi:10.1186/s12864-016-3184-2)
Supplement: Additional file 1: Table S1. — PPR genes in foxtail millet. Detailed genomic information, including domain/class present, ORF length, predicted protein length, genomic locus (chromosomal location), number of introns within the ORF, and the subcellular localization of the PPR proteins for each PPR gene. (DOCX 62 kb) [file 12864_2016_3184_MOESM1_ESM.docx]

**Table S1.** PPR genes in foxtail millet. Detailed genomic information, including domain/class present, ORF length, predicted protein length, genomic locus (chromosomal location), number of introns within the ORF, and the subcellular localization of the PPR proteins for each PPR gene.

| **Phytozome**  **Locus Name** | **Protein ID** | **SMART/PfamA Domain/Class** | | **ORF length** | **Protein Length** | **Chromosome** | **Phytozome pseudomolecule position** | **Introns NO.** | **Location** | **Gene orientation** |
| --- | --- | --- | --- | --- | --- | --- | --- | --- | --- | --- |
| Si000105m.g | K3XDY8 | LysM Pkinase Pkinase_Tyr | | 3492 | 1164 | Chr5 | 24737314-24744857 | 10 | _ | Upward |
| Si000187m.g | K3XE70 | P | | 2901 | 967 | Chr5 | 32942506-32947419 | 8 | Chloroplast | Upward |
| Si000252m.g | K3XED5 | DYW | | 2658 | 886 | Chr5 | 30483839-30486638 | 0 | Mitochondria | Downward |
| Si000279m.g | K3XEG2 | P | | 2583 | 861 | Chr5 | 9083576-9086406 | 1 | Chloroplast | Downward |
| Si000316m.g | K3XEJ9 | DYW | | 2475 | 825 | Chr5 | 19714369-19716978 | 0 | Mitochondria | Downward |
| Si000325m.g | K3XEK8 | E | | 2466 | 822 | Chr5 | 47128378-47130905 | 0 | Chloroplast | Upward |
| Si000333m.g | K3XEL6 | P | | 2442 | 814 | Chr5 | 28357392-28362133 | 2 | Mitochondria | Downward |
| Si000377m.g | K3XER0 | DYW | | 2346 | 782 | Chr5 | 46754222-46756681 | 1 | Mitochondria | Upward |
| Si000391m.g | K3XES4 | DYW | | 2328 | 776 | Chr5 | 36238045-36240845 | 1 | Mitochondria | Upward |
| Si000425m.g | K3XEV8 | E | | 2229 | 743 | Chr5 | 17574173-17576404 | 0 | Mitochondria | Downward |
| Si000446m.g | K3XEX9 | PLS | | 2202 | 734 | Chr5 | 34055874-34058341 | 0 | Chloroplast | Upward |
| Si000565m.g | K3XF96 | P | | 2025 | 675 | Chr5 | 38713970-38716978 | 2 | Chloroplast | Downward |
| Si000581m.g | K3XFB2 | DYW | | 1998 | 666 | Chr5 | 19137454-19139594 | 1 | C/M | Downward |
| Si000595m.g | K3XFC6 | P | | 1980 | 660 | Chr5 | 40650922-40652922 | 0 | Chloroplast | Upward |
| Si000626m.g | K3XFF7 | P | | 1935 | 645 | Chr5 | 25905417-25911459 | 10 | Chloroplast | Upward |
| Si000643m.g | K3XFH4 | DYW | | 1920 | 640 | Chr5 | 39978779-39980701 | 0 | Chloroplast | Upward |
| Si000716m.g | K3XFP7 | P | | 1839 | 613 | Chr5 | 3561580-3565306 | 2 | Mitochondria | Downward |
| Si000780m.g | K3XFW1 | E | | 1773 | 591 | Chr5 | 44583923-44585698 | 0 | Mitochondria | Downward |
| Si000794m.g | K3XFX2 | E | | 1755 | 585 | Chr5 | 43728898-43733372 | 2 | Mitochondria | Upward |
| Si000819m.g | K3XG00 | P | | 1728 | 576 | Chr5 | 24879145-24881006 | 0 | Mitochondria | Upward |
| Si000827m.g | K3XG08 | P | | 1719 | 573 | Chr5 | 10138404-10141218 | 2 | Mitochondria | Upward |
| Si000860m.g | K3XG41 | P | | 1686 | 562 | Chr5 | 47061494-47063182 | 0 | Mitochondria | Downward |
| Si000904m.g | K3XG84 | P | | 1656 | 552 | Chr5 | 42988280-42989938 | 0 | Mitochondria | Downward |
| Si000922m.g | K3XGA2 | P | | 1632 | 544 | Chr5 | 8832064-8833843 | 1 | Mitochondria | Upward |
| Si000925m.g | K3XGA5 | P | | 1629 | 543 | Chr5 | 28012293-28014142 | 1 | Chloroplast | Upward |
| Si001037m.g | K3XGL6 | P | | 1560 | 520 | Chr5 | 2669297-2672659 | 1 | Mitochondria | Upward |
| Si001059m.g | K3XGN8 | P | | 1551 | 517 | Chr5 | 39819744-39821297 | 0 | Mitochondria | Upward |
| Si001102m.g | K3XGT1 | DYW | | 1530 | 510 | Chr5 | 41413041-41415292 | 0 | _ | Downward |
| Si001169m.g | K3XGZ8 | P | | 1485 | 495 | Chr5 | 44556824-44559371 | 1 | Mitochondria | Upward |
| Si001224m.g | K3XH52 | P | | 1461 | 487 | Chr5 | 44361793-44365194 | 2 | Mitochondria | Upward |
| Si001272m.g | K3XHA1 | P | | 1434 | 478 | Chr5 | 32768126-32769562 | 0 | _ | Downward |
| Si001526m.g | K3XI04 | P | | 1296 | 432 | Chr5 | 38848725-38850376 | 1 | Mitochondria | Upward |
| Si001716m.g | K3XIJ4 | P | | 1203 | 401 | Chr5 | 4405325-4408308 | 1 | _ | Upward |
| Si001783m.g | K3XIR2 | P | | 1173 | 391 | Chr5 | 39005239-39008614 | 1 | Mitochondria | Upward |
| Si002242m.g | K3XK19 | P | | 969 | 323 | Chr5 | 24875785-24876790 | 1 | Mitochondria | Downward |
| Si002336m.g | K3XKB3 | P | | 933 | 311 | Chr5 | 37346686-37350395 | 5 | Mitochondria | Downward |
| Si003738m.g | K3XPB5 | P | | 2688 | 896 | Chr5 | 45907627-45910317 | 0 | Mitochondria | Upward |
| Si003790m.g | K3XPG6 | E | | 2181 | 727 | Chr5 | 47242452-47244805 | 2 | Mitochondria | Upward |
| Si003824m.g | K3XPJ7 | PLS | | 1365 | 455 | Chr5 | 22437909-22439276 | 0 | Chloroplast | Upward |
| Si003835m.g | K3XPK8 | P | | 1785 | 595 | Chr5 | 37143099-37145075 | 2 | Mitochondria | Downward |
| Si003962m.g | K3XPY2 | E | | 1308 | 436 | Chr5 | 35645254-35646561 | 0 | _ | Upward |
| Si003975m.g | K3XPZ5 | P | | 2493 | 831 | Chr5 | 12116516-12119605 | 1 | Mitochondria | Upward |
| Si003992m.g | K3XQ12 | E | | 1668 | 556 | Chr5 | 39929121-39930791 | 0 | Mitochondria | Downward |
| Si004007m.g | K3XQ27 | P | | 2109 | 703 | Chr5 | 41679675-41681786 | 0 | _ | Upward |
| Si004067m.g | K3XQ87 | P | | 561 | 187 | Chr5 | 25593621-25594195 | 1 | _ | Upward |
| Si004100m.g | K3XQB7 | E | | 1812 | 604 | Chr5 | 35158180-35159991 | 0 | Mitochondria | Downward |
| Si004159m.g | K3XQH3 | P | | 2016 | 672 | Chr5 | 38949434-38951452 | 0 | Mitochondria | Downward |
| Si004167m.g | K3XQI1 | DYW | | 3069 | 1023 | Chr5 | 26942209-26945376 | 1 | Chloroplast | Downward |
| Si004217m.g | K3XQN0 | P | | 1854 | 618 | Chr5 | 26306566-26308422 | 0 | Mitochondria | Downward |
| Si004230m.g | K3XQP3 | DYW | | 2760 | 920 | Chr5 | 4094435-4097284 | 2 | Chloroplast | Downward |
| Si004235m.g | K3XQP8 | E | | 1377 | 459 | Chr5 | 41014830-41016209 | 0 | Mitochondria | Upward |
| Si004306m.g | K3XQW7 | PLS | | 3273 | 1091 | Chr5 | 4665814-4669089 | 0 | Chloroplast | Upward |
| Si004352m.g | K3XR13 | DYW | | 1551 | 517 | Chr5 | 43237326-43238879 | 0 | Mitochondria | Upward |
| Si004356m.g | K3XR17 | E | | 600 | 200 | Chr5 | 17539473-17540107 | 1 | _ | Upward |
| Si004364m.g | K3XR25 | DYW | | 2412 | 804 | Chr5 | 5702298-5704712 | 0 | Mitochondria | Downward |
| Si004376m.g | K3XR37 | P | | 2127 | 709 | Chr5 | 9879966-9882292 | 1 | Mitochondria | Downward |
| Si004380m.g | K3XR41 | E | | 1293 | 431 | Chr5 | 39248199-39249491 | 0 | Mitochondria | Upward |
| Si004475m.g | K3XRD3 | E | | 1803 | 601 | Chr5 | 3210135-3212027 | 1 | _ | Upward |
| Si004501m.g | K3XRF9 | DYW | | 1869 | 623 | Chr5 | 35154024-35155895 | 0 | _ | Upward |
| Si004548m.g | K3XRK5 | E | | 1647 | 549 | Chr5 | 39747805-39749751 | 1 | Mitochondria | Upward |
| Si004569m.g | K3XRM5 | P | | 1629 | 543 | Chr5 | 26919673-26921304 | 0 | Mitochondria | Downward |
| Si004577m.g | K3XRN3 | E | | 1839 | 613 | Chr5 | 30574831-30576849 | 1 | Mitochondria | Upward |
| Si004604m.g | K3XRR0 | DYW | | 1740 | 580 | Chr5 | 37557000-37558742 | 0 | Mitochondria | Downward |
| Si004621m.g | K3XRS5 | DYW | | 1803 | 601 | Chr5 | 30791311-30793116 | 0 | _ | Upward |
| Si004622m.g | K3XRS6 | P | | 1986 | 662 | Chr5 | 2130669-2132657 | 0 | Mitochondria | Upward |
| Si004681m.g | K3XRY5 | E | | 1515 | 505 | Chr5 | 4897207-4898724 | 0 | Chloroplast | Downward |
| Si004729m.g | K3XS32 | P | | 1137 | 379 | Chr5 | 41104502-41105638 | 0 | Mitochondria | Upward |
| Si004766m.g | K3XS69 | P | | 1425 | 475 | Chr5 | 38853949-38855376 | 0 | Chloroplast | Upward |
| Si004775m.g | K3XS77 | DYW | | 1869 | 623 | Chr5 | 10415396-10417267 | 0 | Mitochondria | Downward |
| Si004822m.g | K3XSC3 | P | | 1131 | 377 | Chr5 | 28847643-28849438 | 1 | C/M | Upward |
| Si005055m.g | K3XT00 | P | | 1449 | 483 | Chr5 | 37761676-37763127 | 0 | _ | Downward |
| Si005072m.g | K3XT17 | E | | 1290 | 430 | Chr5 | 38857578-38858975 | 1 | Chloroplast | Upward |
| Si005100m.g | K3XT44 | E | | 1581 | 527 | Chr5 | 2770171-2772370 | 2 | Mitochondria | Downward |
| Si005162m.g | K3XTA6 | DYW | | 1941 | 647 | Chr5 | 41724720-41726663 | 0 | _ | Upward |
| Si005164m.g | K3XTA8 | E | | 1602 | 534 | Chr5 | 44534811-44536415 | 0 | Mitochondria | Downward |
| Si005165m.g | K3XTA9 | P | | 1779 | 593 | Chr5 | 12535592-12537370 | 0 | Mitochondria | Downward |
| Si005166m.g | K3XTB0 | P | | 1521 | 507 | Chr5 | 5363770-5366064 | 2 | Chloroplast | Downward |
| Si005172m.g | K3XTB6 | P | | 1608 | 536 | Chr5 | 2657173-2660972 | 1 | Chloroplast | Upward |
| Si005691m.g | K3XUT4 | P | | 4344 | 1448 | Chr4 | 2608341-2616420 | 7 | Chloroplast | Upward |
| Si005765m.g | K3XV08 | P | | 3063 | 1021 | Chr4 | 622229-626448 | 7 | Chloroplast | Upward |
| Si005769m.g | K3XV12 | P | | 3015 | 1005 | Chr4 | 37058286-37061587 | 0 | Mitochondria | Upward |
| Si005778m.g | K3XV21 | P | | 2964 | 988 | Chr4 | 37333975-37336990 | 0 | Chloroplast | Downward |
| Si005890m.g | K3XVD3 | P | | 2364 | 788 | Chr4 | 30895976-30898612 | 0 | Chloroplast | Downward |
| Si005891m.g | K3XVD4 | DYW | | 2364 | 788 | Chr4 | 3467239-3469605 | 0 | Mitochondria | Upward |
| Si005922m.g | K3XVG5 | DYW | | 2244 | 748 | Chr4 | 26581248-26583865 | 0 | C/M | Downward |
| Si005929m.g | K3XVH2 | P | | 2229 | 743 | Chr4 | 1767033-1770731 | 2 | Chloroplast | Downward |
| Si005931m.g | K3XVH4 | P | | 2220 | 740 | Chr4 | 38825371-38831546 | 3 | _ | Downward |
| Si005933m.g | K3XVH6 | DYW | | 2211 | 737 | Chr4 | 537041-539254 | 0 | Chloroplast | Downward |
| Si005994m.g | K3XVN6 | E | | 2046 | 682 | Chr4 | 8627028-8629441 | 0 | Chloroplast | Upward |
| Si006042m.g | K3XVT4 | DYW | | 1917 | 639 | Chr4 | 26819037-26820956 | 0 | Mitochondria | Upward |
| Si006059m.g | K3XVV1 | P | | 1887 | 629 | Chr4 | 33521771-33524899 | 3 | _ | Upward |
| Si006072m.g | K3XVW4 | E | | 1872 | 624 | Chr4 | 33829748-33832076 | 0 | Chloroplast | Upward |
| Si006080m.g | K3XVX2 | E | | 1860 | 620 | Chr4 | 1817143-1819037 | 0 | Mitochondria | Downward |
| Si006109m.g | K3XW01 | DYW | | 1794 | 598 | Chr4 | 8132383-8134805 | 0 | Chloroplast | Downward |
| Si006121m.g | K3XW13 | DYW | | 1764 | 588 | Chr4 | 33487860-33491785 | 3 | Mitochondria | Upward |
| Si006180m.g | K3XW72 | P | | 1653 | 551 | Chr4 | 5443292-5445476 | 2 | Chloroplast | Upward |
| Si006203m.g | K3XW95 | P | | 1620 | 540 | Chr4 | 105871-111000 | 4 | Mitochondria | Downward |
| Si006246m.g | K3XWD8 | E | | 1554 | 518 | Chr4 | 706571-708559 | 0 | Chloroplast | Upward |
| Si006247m.g | K3XWD9 | P | | 1551 | 517 | Chr4 | 1232471-1236608 | 2 | Mitochondria | Upward |
| Si006249m.g | K3XWE1 | E | | 1551 | 517 | Chr4 | 1004054-1005891 | 1 | Mitochondria | Upward |
| Si006274m.g | K3XWG7 | P | | 1527 | 509 | Chr4 | 8807909-8810755 | 3 | Mitochondria | Downward |
| Si006322m.g | K3XWL4 | P | | 1455 | 485 | Chr4 | 37065004-37066721 | 0 | Mitochondria | Upward |
| Si006390m.g | K3XWT2 | P | | 1377 | 459 | Chr4 | 36688073-36689820 | 0 | Mitochondria | Downward |
| Si007926m.g | K3Y164 | DYW | | 1794 | 598 | Chr4 | 8791450-8793255 | 1 | Chloroplast | Downward |
| Si008069m.g | K3Y1K4 | E | | 1581 | 527 | Chr4 | 34623693-34625276 | 0 | Mitochondria | Upward |
| Si008084m.g | K3Y1L8 | E | | 1689 | 563 | Chr4 | 12536635-12538323 | 0 | Chloroplast | Upward |
| Si008099m.g | K3Y1N3 | DYW | | 2223 | 741 | Chr4 | 38833497-38835722 | 0 | Mitochondria | Downward |
| Si008175m.g | K3Y1V6 | E | | 1164 | 388 | Chr4 | 3503030-3504196 | 0 | Mitochondria | Upward |
| Si008179m.g | K3Y1W0 | E | | 1476 | 492 | Chr4 | 4270416-4272310 | 2 | Chloroplast | Downward |
| Si008514m.g | K3Y2T4 | E | | 1584 | 528 | Chr4 | 8782785-8784371 | 0 | Mitochondria | Downward |
| Si008540m.g | K3Y2V8 | P | | 2055 | 685 | Chr4 | 3470970-3473027 | 0 | Chloroplast | Downward |
| Si008546m.g | K3Y2W3 | P | | 1509 | 503 | Chr4 | 5952609-5954120 | 0 | Chloroplast | Upward |
| Si008548m.g | K3Y2W5 | P | | 1527 | 509 | Chr4 | 17104552-17106995 | 2 | _ | Upward |
| Si008589m.g | K3Y304 | P | | 1359 | 453 | Chr4 | 6035710-6037068 | 0 | Mitochondria | Upward |
| Si008652m.g | K3Y367 | P | | 1677 | 559 | Chr4 | 7488707-7490796 | 1 | Mitochondria | Downward |
| Si008679m.g | K3Y394 | P | | 1788 | 596 | Chr4 | 10342608-10344395 | 0 | Mitochondria | Downward |
| Si008699m.g | K3Y3B4 | P | | 1275 | 425 | Chr4 | 32748275-32749552 | 0 | _ | Upward |
| Si008727m.g | K3Y3E2 | P | | 1479 | 493 | Chr4 | 39245469-39248081 | 4 | Mitochondria | Upward |
| Si008760m.g | K3Y3H5 | DYW | | 1962 | 654 | Chr4 | 37074380-37076344 | 0 | Mitochondria | Downward |
| Si008825m.g | K3Y3N8 | P | | 2103 | 701 | Chr4 | 36197963-36200068 | 0 | Mitochondria | Downward |
| Si009274m.g | K3Y4Y6 | P | | 2901 | 967 | Chr7 | 23440136-23443946 | 1 | Mitochondria | Upward |
| Si009278m.g | K3Y4Z0 | P | | 2871 | 957 | Chr7 | 17840584-17843710 | 0 | Mitochondria | Upward |
| Si009326m.g | K3Y538 | DYW | | 2658 | 886 | Chr7 | 19118936-19121834 | 0 | _ | Upward |
| Si009336m.g | K3Y548 | DYW | | 2595 | 865 | Chr7 | 28344539-28347136 | 0 | Mitochondria | Upward |
| Si009380m.g | K3Y592 | P | | 2409 | 803 | Chr7 | 29097452-29100336 | 0 | Chloroplast | Downward |
| Si009423m.g | K3Y5D5 | P | | 2316 | 772 | Chr7 | 22131234-22133552 | 0 | Chloroplast | Downward |
| Si009450m.g | K3Y5G2 | P | | 2244 | 748 | Chr7 | 15690397-15692828 | 0 | Chloroplast | Downward |
| Si009504m.g | K3Y5L6 | PLS | | 2115 | 705 | Chr7 | 28250714-28253315 | 1 | C/M | Upward |
| Si009530m.g | K3Y5P1 | E | | 2067 | 689 | Chr7 | 16104895-16107185 | 1 | Mitochondria | Downward |
| Si009532m.g | K3Y5P3 | E | | 2061 | 687 | Chr7 | 20571277-20573388 | 0 | Mitochondria | Downward |
| Si009592m.g | K3Y5V3 | DYW | | 1905 | 635 | Chr7 | 35633431-35635386 | 1 | _ | Upward |
| Si009625m.g | K3Y5Y6 | DYW | | 1857 | 619 | Chr7 | 24782092-24785959 | 3 | Chloroplast | Downward |
| Si009635m.g | K3Y5Z6 | E | | 1821 | 607 | Chr7 | 19594208-19596031 | 0 | Mitochondria | Upward |
| Si009638m.g | K3Y5Z9 | P | | 1818 | 606 | Chr7 | 22103593-22105413 | 0 | Mitochondria | Upward |
| Si009676m.g | K3Y637 | E | | 1764 | 588 | Chr7 | 35415031-35416955 | 2 | Mitochondria | Upward |
| Si009770m.g | K3Y6C9 | P | | 1623 | 541 | Chr7 | 22781622-22783352 | 0 | _ | Upward |
| Si010036m.g | K3Y745 | P | | 1395 | 465 | Chr7 | 25185717-25190652 | 1 | _ | Upward |
| Si010112m.g | K3Y7C1 | P | | 1335 | 445 | Chr7 | 31348309-31349954 | 0 | Mitochondria | Upward |
| Si010241m.g | K3Y7Q0 | P | | 1224 | 408 | Chr7 | 21506266-21508419 | 1 | Mitochondria | Upward |
| Si010939m.g | K3Y9P7 | P | | 726 | 242 | Chr7 | 22759189-22761572 | 1 | Mitochondria | Downward |
| Si011681m.g | K3YBT7 | P | | 1926 | 642 | Chr7 | 31031429-31033357 | 0 | Mitochondria | Upward |
| Si011688m.g | K3YBU4 | DYW | | 2859 | 953 | Chr7 | 22462397-22465258 | 0 | Chloroplast | Downward |
| Si011726m.g | K3YBY2 | P | | 2160 | 720 | Chr7 | 35444897-35447059 | 0 | Chloroplast | Downward |
| Si011763m.g | K3YC19 | E | | 2283 | 761 | Chr7 | 25103594-25105879 | 0 | Chloroplast | Downward |
| Si011834m.g | K3YC90 | E | | 3060 | 1020 | Chr7 | 24749924-24752986 | 0 | Mitochondria | Downward |
| Si011942m.g | K3YCJ4 | P | | 1494 | 498 | Chr7 | 33821002-33823140 | 2 | Mitochondria | Upward |
| Si011969m.g | K3YCM0 | E | | 1260 | 420 | Chr7 | 27755772-27757240 | 2 | Mitochondria | Downward |
| Si012001m.g | K3YCQ2 | P | | 1143 | 381 | Chr7 | 21237261-21238406 | 0 | _ | Upward |
| Si012024m.g | K3YCS4 | P | | 2139 | 713 | Chr7 | 20579599-20581740 | 0 | Chloroplast | Downward |
| Si012075m.g | K3YCX4 | P | | 2067 | 689 | Chr7 | 32530440-32532509 | 0 | Chloroplast | Downward |
| Si012080m.g | K3YCX9 | DYW | | 1788 | 596 | Chr7 | 33628099-33629889 | 0 | _ | Downward |
| Si012084m.g | K3YCY3 | E | | 1500 | 500 | Chr7 | 31819173-31820675 | 0 | Chloroplast | Upward |
| Si012138m.g | K3YD35 | E | | 1512 | 504 | Chr7 | 33913465-33914976 | 0 | Mitochondria | Downward |
| Si012174m.g | K3YD71 | DYW | | 2085 | 695 | Chr7 | 23038887-23040974 | 0 | Chloroplast | Downward |
| Si012220m.g | K3YDB6 | P | | 1266 | 422 | Chr7 | 34175516-34178074 | 2 | Mitochondria | Downward |
| Si012233m.g | K3YDC9 | E | | 1452 | 484 | Chr7 | 32701368-32702957 | 1 | Mitochondria | Upward |
| Si012259m.g | K3YDF4 | P | | 1662 | 554 | Chr7 | 21216610-21218274 | 0 | Mitochondria | Downward |
| Si012267m.g | K3YDG2 | P | | 1083 | 361 | Chr7 | 30101631-30102713 | 0 | Mitochondria | Upward |
| Si012413m.g | K3YDV5 | E | | 2715 | 905 | Chr7 | 20907222-20909939 | 0 | Mitochondria | Upward |
| Si012426m.g | K3YDW7 | P | | 2097 | 699 | Chr7 | 35255292-35257908 | 3 | Mitochondria | Downward |
| Si012467m.g | K3YE08 | P | | 2667 | 889 | Chr7 | 29712047-29715179 | 2 | _ | Downward |
| Si012507m.g | K3YE47 | P | | 2274 | 758 | Chr7 | 15763106-15765434 | 1 | Chloroplast | Upward |
| Si012630m.g | K3YEG6 | P | | 1941 | 647 | Chr7 | 15683154-15685289 | 1 | Mitochondria | Downward |
| Si012711m.g | K3YEP5 | P | | 1260 | 420 | Chr7 | 34119609-34121333 | 3 | _ | Upward |
| Si012752m.g | K3YET5 | PLS | | 2208 | 736 | Chr7 | 35952992-35955372 | 2 | Chloroplast | Upward |
| Si012850m.g | K3YF33 | P | | 315 | 105 | Chr7 | 22856622-22857215 | 0 | _ | Downward |
| Si013161m.g | K3YFZ3 | DYW | | 3264 | 1088 | Chr6 | 33093915-33097446 | 0 | Chloroplast | Upward |
| Si013172m.g | K3YG04 | DYW DAGKc DAGKa | | 3132 | 1044 | Chr6 | 9929740-9938657 | 12 | Chloroplast | Upward |
| Si013244m.g | K3YG76 | DYW | | 2586 | 862 | Chr6 | 23402336-23404924 | 0 | Chloroplast | Upward |
| Si013265m.g | K3YG97 | P | | 2451 | 817 | Chr6 | 2399796-2402691 | 1 | Mitochondria | Upward |
| Si013359m.g | K3YGJ1 | E | | 2034 | 678 | Chr6 | 848981-852690 | 3 | Chloroplast | Downward |
| Si013431m.g | K3YGR3 | DYW | | 1830 | 610 | Chr6 | 4849951-4851783 | 0 | Mitochondria | Downward |
| Si013441m.g | K3YGS3 | P | | 1806 | 602 | Chr6 | 2709681-2714433 | 6 | Chloroplast | Downward |
| Si013474m.g | K3YGV6 | E | | 1743 | 581 | Chr6 | 34168375-34170120 | 0 | _ | Downward |
| Si013541m.g | K3YH23 | P | | 1590 | 530 | Chr6 | 12304073-12309785 | 3 | Mitochondria | Downward |
| Si013553m.g | K3YH35 | E | | 1584 | 528 | Chr6 | 3985814-3987846 | 0 | Mitochondria | Downward |
| Si015005m.g | K3YL83 | P | | 1086 | 362 | Chr6 | 803310-804796 | 1 | Mitochondria | Upward |
| Si015139m.g | K3YLL5 | PLS | | 1950 | 650 | Chr6 | 30484545-30486725 | 1 | Chloroplast | Upward |
| Si015208m.g | K3YLT3 | P | | 1377 | 459 | Chr6 | 33398995-33400374 | 0 | Mitochondria | Downward |
| Si015299m.g | K3YM22 | P | | 2328 | 776 | Chr6 | 15724126-15726489 | 1 | Mitochondria | Upward |
| Si015302m.g | K3YM25 | DYW | | 2064 | 688 | Chr6 | 5974442-5976508 | 0 | Chloroplast | Downward |
| Si015328m.g | K3YM50 | PLS | | 2637 | 879 | Chr6 | 34479834-34482470 | 0 | Chloroplast | Upward |
| Si015375m.g | K3YM96 | P | | 1491 | 497 | Chr6 | 32060339-32061829 | 0 | Mitochondria | Upward |
| Si015401m.g | K3YMC2 | P | | 1371 | 457 | Chr6 | 31996941-31998314 | 0 | Chloroplast | Upward |
| Si015406m.g | K3YMC7 | P | | 2295 | 765 | Chr6 | 32806976-32809363 | 2 | Mitochondria | Upward |
| Si015407m.g | K3YMC8 | PLS | | 2046 | 682 | Chr6 | 6579781-6581829 | 0 | Mitochondria | Downward |
| Si015417m.g | K3YMD8 | P | | 1575 | 525 | Chr6 | 3981843-3983921 | 1 | Mitochondria | Downward |
| Si015438m.g | K3YMF9 | P | | 2100 | 700 | Chr6 | 25023931-25026030 | 0 | Mitochondria | Upward |
| Si015461m.g | K3YMI2 | DYW | | 1179 | 393 | Chr6 | 28228178-28229403 | 1 | Mitochondria | Downward |
| Si015592m.g | K3YMV9 | E | | 1641 | 547 | Chr6 | 31184769-31187709 | 1 | Mitochondria | Upward |
| Si015619m.g | K3YMY5 | DYW | | 1923 | 641 | Chr6 | 32615828-32617753 | 0 | Mitochondria | Downward |
| Si015629m.g | K3YMZ5 | DYW | | 2232 | 744 | Chr6 | 22717570-22720475 | 1 | Chloroplast | Downward |
| Si015792m.g | K3YNF1 | E | | 2091 | 697 | Chr6 | 22638233-22640326 | 0 | Mitochondria | Downward |
| Si016257m.g | K3YPR5 | P | | 2742 | 914 | Chr1 | 8807072-8810950 | 1 | Mitochondria | Downward |
| Si016264m.g | K3YPS2 | P | | 2712 | 904 | Chr1 | 8462321-8469863 | 14 | Chloroplast | Upward |
| Si016291m.g | K3YPU9 | P | | 2598 | 866 | Chr1 | 8503775-8507587 | 2 | Chloroplast | Upward |
| Si016319m.g | K3YPX7 | P | | 2460 | 820 | Chr1 | 29045861-29048439 | 0 | Chloroplast | Downward |
| Si016353m.g | K3YQ11 | P LAGLIDADG_2 | | 2379 | 793 | Chr1 | 35708015-35710754 | 1 | Chloroplast | Upward |
| Si016367m.g | K3YQ25 | P | | 2343 | 781 | Chr1 | 40806413-40808758 | 0 | _ | Upward |
| Si016506m.g | K3YQG3 | E | | 2094 | 698 | Chr1 | 35930255-35932351 | 0 | _ | Upward |
| Si016535m.g | K3YQJ2 | E | | 2055 | 685 | Chr1 | 34546348-34548708 | 1 | Chloroplast | Upward |
| Si016553m.g | K3YQL2 | P | | 2028 | 676 | Chr1 | 30606684-30610683 | 2 | Mitochondria | Upward |
| Si016590m.g | K3YQP6 | P | | 1968 | 656 | Chr1 | 22107709-22110780 | 2 | Chloroplast | Downward |
| Si016610m.g | K3YQR6 | P | | 1935 | 645 | Chr1 | 8836564-8840659 | 4 | Chloroplast | Downward |
| Si016662m.g | K3YQW8 | PLS | | 1860 | 620 | Chr1 | 9386650-9388512 | 0 | _ | Downward |
| Si016685m.g | K3YQZ1 | P | | 1818 | 606 | Chr1 | 4555519-4557339 | 0 | Mitochondria | Downward |
| Si016689m.g | K3YQZ5 | P | | 1812 | 604 | Chr1 | 22016906-22018938 | 1 | _ | Upward |
| Si016698m.g | K3YR04 | P | | 1797 | 599 | Chr1 | 681050-684097 | 1 | _ | Downward |
| Si016732m.g | K3YR38 | DYW | | 1755 | 585 | Chr1 | 27364323-27366630 | 1 | Mitochondria | Upward |
| Si016815m.g | K3YRC1 | P | | 1656 | 552 | Chr1 | 28496897-28498756 | 0 | Mitochondria | Upward |
| Si016821m.g | K3YRC7 | P | | 1650 | 550 | Chr1 | 38766840-38768492 | 0 | Mitochondria | Upward |
| Si016833m.g | K3YRD9 | P | | 1638 | 546 | Chr1 | 6646767-6650737 | 6 | C/M | Downward |
| Si016865m.g | K3YRH1 | P | | 1605 | 535 | Chr1 | 28124411-28127698 | 2 | Chloroplast | Upward |
| Si016875m.g | K3YRI1 | E | | 1590 | 530 | Chr1 | 31520924-31522573 | 0 | Mitochondria | Downward |
| Si016962m.g | K3YRR8 | P | | 1515 | 505 | Chr1 | 30054985-30056539 | 0 | Mitochondria | Upward |
| Si016997m.g | K3YRV3 | PLS | | 1497 | 499 | Chr1 | 41930223-41931729 | 0 | Chloroplast | Upward |
| Si017009m.g | K3YRW5 | E | | 1488 | 496 | Chr1 | 39222961-39224597 | 0 | Chloroplast | Downward |
| Si017099m.g | K3YS55 | P | | 1419 | 473 | Chr1 | 7015902-7017323 | 0 | Mitochondria | Downward |
| Si017139m.g | K3YS95 | P | | 1386 | 462 | Chr1 | 32196599-32198299 | 0 | Mitochondria | Upward |
| Si017469m.g | K3YT74 | P | | 1167 | 389 | Chr1 | 9695188-9696829 | 0 | Mitochondria | Upward |
| Si019062m.g | K3YXR5 | P | | 1725 | 575 | Chr1 | 41454088-41455815 | 0 | Mitochondria | Upward |
| Si019135m.g | K3YXY5 | P | | 1986 | 662 | Chr1 | 22082060-22084350 | 1 | Chloroplast | Upward |
| Si019387m.g | K3YYN2 | P | | 1386 | 462 | Chr1 | 27884244-27886144 | 2 | Mitochondria | Downward |
| Si019461m.g | K3YYV5 | P | | 1896 | 632 | Chr1 | 33217666-33219564 | 0 | _ | Downward |
| Si019494m.g | K3YYY8 | DYW | | 2073 | 691 | Chr1 | 4637358-4639542 | 1 | _ | Upward |
| Si019498m.g | K3YYZ2 | PLS | | 1794 | 598 | Chr1 | 41852883-41854827 | 1 | _ | Downward |
| Si019532m.g | K3YZ25 | P | | 264 | 88 | Chr1 | 10045020-10045371 | 1 | _ | Downward |
| Si019566m.g | K3YZ58 | P | | 2394 | 798 | Chr1 | 25809585-25811981 | 0 | Chloroplast | Downward |
| Si019580m.g | K3YZ72 | P | | 1926 | 642 | Chr1 | 37327909-37329837 | 0 | Chloroplast | Upward |
| Si019614m.g | K3YZA6 | E | | 1983 | 661 | Chr1 | 33725719-33727704 | 0 | Mitochondria | Downward |
| Si019685m.g | K3YZH6 | E | | 2421 | 807 | Chr1 | 6876310-6878733 | 0 | Chloroplast | Upward |
| Si019714m.g | K3YZK4 | P | | 1515 | 505 | Chr1 | 34500124-34501641 | 0 | Chloroplast | Downward |
| Si019777m.g | K3YZR7 | E | | 1482 | 494 | Chr1 | 31699769-31701253 | 0 | Chloroplast | Upward |
| Si019779m.g | K3YZR9 | P | | 1470 | 490 | Chr1 | 36164125-36165597 | 0 | Mitochondria | Downward |
| Si019792m.g | K3YZT2 | P | | 1431 | 477 | Chr1 | 9683421-9685441 | 1 | Chloroplast | Upward |
| Si019849m.g | K3YZY8 | DYW | | 1371 | 457 | Chr1 | 36726211-36727584 | 0 | Mitochondria | Downward |
| Si019887m.g | K3Z026 | P | | 894 | 298 | Chr1 | 15780955-15781848 | 0 | Chloroplast | Downward |
| Si019908m.g | K3Z047 | PLS | | 1899 | 633 | Chr1 | 2850276-2853437 | 1 | Chloroplast | Upward |
| Si019943m.g | K3Z082 | E | | 1791 | 597 | Chr1 | 27487351-27489144 | 0 | Mitochondria | Upward |
| Si020047m.g | K3Z0I1 | P | | 1368 | 456 | Chr1 | 31585192-31586562 | 0 | Mitochondria | Downward |
| Si020179m.g | K3Z0W2 | E | | 1968 | 656 | Chr1 | 8678925-8680895 | 0 | Mitochondria | Upward |
| Si020192m.g | K3Z0X5 | P | | 1272 | 424 | Chr1 | 41755814-41757189 | 2 | Chloroplast | Downward |
| Si020193m.g | K3Z0X6 | PLS ZnF_C2H2 E | | 2595 | 865 | Chr1 | 10254126-10259518 | 6 | Mitochondria | Downward |
| Si020204m.g | K3Z0Y7 | DYW | | 2649 | 883 | Chr1 | 29526724-29529375 | 0 | Mitochondria | Upward |
| Si020206m.g | K3Z0Y9 | P | | 2166 | 722 | Chr1 | 10389056-10391435 | 1 | Mitochondria | Upward |
| Si020207m.g | K3Z0Z0 | P | | 1902 | 634 | Chr1 | 10542719-10545246 | 2 | Chloroplast | Downward |
| Si020221m.g | K3Z104 | P | | 1242 | 414 | Chr1 | 41924869-41926130 | 1 | Mitochondria | Downward |
| Si020237m.g | K3Z119 | E | | 1446 | 482 | Chr1 | 37099718-37101166 | 0 | Chloroplast | Downward |
| Si020238m.g | K3Z120 | P | | 1485 | 495 | Chr1 | 38224571-38226557 | 1 | Mitochondria | Upward |
| Si020239m.g | K3Z121 | P | | 1557 | 519 | Chr1 | 40441789-40443886 | 1 | Chloroplast | Downward |
| Si020380m.g | K3Z1G2 | P | | 255 | 85 | Chr1 | 41755437-41755752 | 0 | _ | Downward |
| Si020686m.g | K3Z2B8 | P | | 1977 | 659 | _ | scaffold_11:198410-200389 | 0 | _ | Downward |
| Si021162m.g | K3Z3P4 | PLS RRM | | 2673 | 891 | Chr3 | 490063-495987 | 9 | Chloroplast | Downward |
| Si021190m.g | K3Z3S2 | PLS | | 2601 | 867 | Chr3 | 42833745-42837613 | 3 | Chloroplast | Downward |
| Si021191m.g | K3Z3S3 | DYW | | 2595 | 865 | Chr3 | 9539489-9542086 | 0 | Chloroplast | Downward |
| Si021194m.g | K3Z3S6 | P | | 2580 | 860 | Chr3 | 50499113-50504543 | 2 | Mitochondria | Downward |
| Si021196m.g | K3Z3S8 | DYW | | 2571 | 857 | Chr3 | 8595792-8599263 | 1 | Chloroplast | Downward |
| Si021258m.g | K3Z3Z0 | P | | 2388 | 796 | Chr3 | 6334285-6336675 | 0 | Mitochondria | Upward |
| Si021325m.g | K3Z457 | P | | 2193 | 731 | Chr3 | 12205263-12207948 | 1 | _ | Downward |
| Si021338m.g | K3Z470 | P | | 2166 | 722 | Chr3 | 49352629-49355076 | 0 | Mitochondria | Downward |
| Si021396m.g | K3Z5N9 | P | | 1482 | 494 | Chr3 | 36791277-36793533 | 1 | _ | Upward |
| Si021396m.g | K3Z4C8 | P | | 2013 | 671 | Chr3 | 36791277-36793533 | 0 | Chloroplast | Upward |
| Si021399m.g | K3Z4D1 | DYW | | 2007 | 669 | Chr3 | 23814083-23816092 | 0 | Mitochondria | Upward |
| Si021453m.g | K3Z4I5 | P | | 1908 | 636 | Chr3 | 12273424-12275334 | 0 | Mitochondria | Upward |
| Si021471m.g | K3Z4K3 | E | | 1875 | 625 | Chr3 | 9221740-9223821 | 1 | Mitochondria | Downward |
| Si021489m.g | K3Z4M1 | E | | 1851 | 617 | Chr3 | 46766429-46768282 | 0 | Mitochondria | Downward |
| Si021496m.g | K3Z4M8 | E | | 1842 | 614 | Chr3 | 2382076-2383920 | 0 | _ | Downward |
| Si021546m.g | K3Z4S8 | E | | 1791 | 596 | Chr3 | 911009..912799 | 0 | Mitochondria | Upward |
| Si021705m.g | K3Z587 | E | | 1611 | 537 | Chr3 | 2897230-2899489 | 1 | C/M | Downward |
| Si021749m.g | K3Z5D1 | P | | 1578 | 526 | Chr3 | 583153-585316 | 1 | Mitochondria | Upward |
| Si021777m.g | K3Z5F9 | DYW | | 1548 | 516 | Chr3 | 20220626-20222436 | 0 | Mitochondria | Upward |
| Si021872m.g | K3Z5Q4 | DYW | | 1473 | 491 | Chr3 | 4833326-4835864 | 1 | Chloroplast | Upward |
| Si022021m.g | K3Z653 | P | | 1353 | 451 | Chr3 | 3822294-3827538 | 12 | Mitochondria | Downward |
| Si023345m.g | K3Z9X4 | P | | 570 | 190 | Chr3 | 29228883-29231519 | 3 | Mitochondria | Downward |
| Si024073m.g | K3ZC00 | E | | 1914 | 638 | Chr3 | 3230980-3233127 | 1 | Chloroplast | Upward |
| Si024194m.g | K3ZCC0 | P | | 1323 | 441 | Chr3 | 49396633-49398068 | 1 | Mitochondria | Upward |
| Si024301m.g | K3ZCM5 | P | | 1941 | 647 | Chr3 | 22067758-22069746 | 1 | Mitochondria | Downward |
| Si024323m.g | K3ZCP6 | E | | 2154 | 718 | Chr3 | 48672826-48675051 | 1 | Chloroplast | Upward |
| Si024327m.g | K3ZCQ0 | E | | 2358 | 786 | Chr3 | 5442516-5444876 | 0 | _ | Upward |
| Si024607m.g | K3ZDH1 | DYW | | 1659 | 553 | Chr3 | 12724055-12726694 | 1 | _ | Downward |
| Si024660m.g | K3ZDM2 | P | | 2184 | 728 | Chr3 | 31366675-31369407 | 1 | Mitochondria | Upward |
| Si024706m.g | K3ZDR5 | E | | 2010 | 670 | Chr3 | 386980-388989 | 0 | Mitochondria | Upward |
| Si024715m.g | K3ZDS4 | P | | 1380 | 460 | Chr3 | 4710288-4711670 | 0 | Chloroplast | Upward |
| Si024927m.g | K3ZED3 | E | | 1203 | 401 | Chr3 | 20914957-20916162 | 0 | Chloroplast | Downward |
| Si024948m.g | K3ZEF3 | E | | 1650 | 550 | Chr3 | 46450604-46452293 | 1 | Mitochondria | Downward |
| Si024990m.g | K3ZEJ5 | E | | 1743 | 581 | Chr3 | 18766173-18768054 | 1 | _ | Upward |
| Si025038m.g | K3ZEP2 | P | | 1533 | 511 | Chr3 | 21120106-21121641 | 0 | Mitochondria | Upward |
| Si025040m.g | K3ZEP4 | E | | 2157 | 719 | Chr3 | 1099943-1102102 | 0 | Chloroplast | Upward |
| Si025063m.g | K3ZER6 | E | | 1956 | 652 | Chr3 | 5905082-5907040 | 0 | Chloroplast | Upward |
| Si025085m.g | K3ZET7 | P | | 747 | 249 | Chr3 | 4704645-4706131 | 2 | _ | Upward |
| Si025098m.g | K3ZEV0 | PLS | | 1647 | 549 | Chr3 | 920458-922107 | 0 | Mitochondria | Upward |
| Si025222m.g | K3ZF73 | DYW | | 2616 | 872 | Chr3 | 18483214-18485832 | 0 | Mitochondria | Upward |
| Si025228m.g | K3ZF79 | P | | 1284 | 428 | Chr3 | 21967236-21968773 | 1 | _ | Upward |
| Si025244m.g | K3ZF95 | PLS | | 2343 | 781 | Chr3 | 28021221-28023563 | 0 | Mitochondria | Upward |
| Si025318m.g | K3ZFG7 | DYW | | 1971 | 657 | Chr3 | 4381945-4384001 | 2 | Mitochondria | Upward |
| Si025343m.g | K3ZFJ1 | P | | 1950 | 650 | Chr3 | 25178790-25180742 | 0 | C/M | Downward |
| Si025355m.g | K3ZFK3 | P | | 1818 | 606 | Chr3 | 25153908-25155785 | 1 | Mitochondria | Downward |
| Si025357m.g | K3ZFK5 | P | | 2592 | 864 | Chr3 | 22487327-22490011 | 2 | Chloroplast | Upward |
| Si025358m.g | K3ZFK6 | P | | 1530 | 510 | Chr3 | 44903376-44905056 | 1 | Mitochondria | Downward |
| Si025361m.g | K3ZFK9 | P | | 1137 | 379 | Chr3 | 15382440-15383933 | 1 | Mitochondria | Downward |
| Si025368m.g | K3ZFL6 | DYW | | 1083 | 361 | Chr3 | 8365600-8367250 | 0 | _ | Downward |
| Si025982m.g | K3ZHD0 | PLS | | 2466 | 822 | Chr8 | 750605-753279 | 1 | Mitochondria | Upward |
| Si026059m.g | K3ZHK7 | E | | 2058 | 686 | Chr8 | 31127615-31129675 | 0 | Mitochondria | Downward |
| Si026106m.g | K3ZHQ4 | PLS | | 1908 | 636 | Chr8 | 2040430-2042765 | 2 | Mitochondria | Upward |
| Si026218m.g | K3ZI16 | E | | 1566 | 522 | Chr8 | 38178303-38179871 | 0 | Chloroplast | Downward |
| Si026902m.g | K3ZJZ7 | P | | 558 | 186 | Chr8 | 2940927-2942132 | 1 | Mitochondria | Upward |
| Si026947m.g | K3ZK42 | P | | 465 | 154 | Chr8 | 964531-965199 | 0 | Mitochondria | Upward |
| Si027294m.g | K3ZL38 | P | | 1986 | 662 | Chr8 | 33160800-33162788 | 0 | _ | Upward |
| Si027424m.g | K3ZLG6 | P | | 1848 | 616 | Chr8 | 1897936-1899849 | 2 | _ | Downward |
| Si027584m.g | K3ZLX2 | P | | 882 | 294 | Chr8 | 30263039-30263975 | 1 | Mitochondria | Downward |
| Si027650m.g | K3ZM37 | P | | 1386 | 462 | Chr8 | 4705377-4706765 | 0 | Mitochondria | Downward |
| Si027698m.g | K3ZM84 | P | | 906 | 301 | Chr8 | 36249712-36250753 | 4 | Mitochondria | Upward |
| Si027712m.g | K3ZM98 | P | | 1743 | 581 | Chr8 | 30551858-30554294 | 1 | Chloroplast | Downward |
| Si027756m.g | K3ZME1 | P | | 2079 | 693 | Chr8 | 31079172-31081289 | 1 | Mitochondria | Downward |
| Si027760m.g | K3ZME5 | P | | 1869 | 623 | Chr8 | 29882484-29884928 | 2 | Chloroplast | Upward |
| Si027770m.g | K3ZMF5 | PLAC8 DUF2985 DYW | | 3492 | 1164 | Chr8 | 980489-984211 | 1 | _ | Upward |
| Si027872m.g | K3ZMQ2 | P | | 1638 | 546 | Chr8 | 811515-813155 | 0 | Chloroplast | Downward |
| Si027888m.g | K3ZMR8 | P | | 1446 | 482 | Chr8 | 22986024-22987472 | 0 | Mitochondria | Downward |
| Si027905m.g | K3ZMT5 | PLS | | 2628 | 876 | Chr8 | 31107278-31110804 | 2 | _ | Downward |
| Si027910m.g | K3ZMU0 | E | | 1692 | 564 | Chr8 | 8172314-8174008 | 0 | Mitochondria | Upward |
| Si028006m.g | K3ZN32 | P | | 2307 | 769 | Chr8 | 30333402-30335919 | 2 | Mitochondria | Upward |
| Si028041m.g | K3ZN66 | PLS | | 2085 | 695 | Chr8 | 30521648-30523845 | 1 | _ | Downward |
| Si028047m.g | K3ZN72 | DYW | | 1920 | 640 | Chr8 | 9975499-9977913 | 2 | _ | Upward |
| Si028089m.g | K3ZNB4 | PLS | | 1074 | 358 | Chr8 | 972755-973831 | 0 | Mitochondria | Upward |
| Si028249m.g | K3ZNR9 | P PGAM | | 1572 | 524 | Chr8 | 1486491-1491921 | 7 | Chloroplast | Downward |
| Si028348m.g | K3ZP18 | P | | 2364 | 788 | Chr8 | 31201831-31204264 | 1 | Mitochondria | Upward |
| Si028351m.g | K3ZP21 | E | | 1551 | 517 | Chr8 | 40415776-40417329 | 0 | Chloroplast | Upward |
| Si028360m.g | K3ZP30 | PLS PGAM | | 2976 | 992 | Chr8 | 1398082-1401749 | 9 | Chloroplast | Downward |
| Si028380m.g | K3ZP50 | P | | 2151 | 717 | Chr8 | 2230148-2232298 | 0 | C/M | Downward |
| Si028837m.g | K3ZQF7 | E Abhydrolase_5 | | 2865 | 955 | Chr2 | 37615978-37622524 | 7 | Chloroplast | Upward |
| Si028853m.g | K3ZQH3 | PLS HAT | | 2784 | 928 | Chr2 | 46961056-46965870 | 6 | C/M | Downward |
| Si028890m.g | K3ZQL0 | P | | 2631 | 877 | Chr2 | 40185660-40188449 | 1 | Chloroplast | Downward |
| Si028899m.g | K3ZQL9 | DYW | | 2601 | 867 | Chr2 | 3822379-3824982 | 0 | Chloroplast | Downward |
| Si028921m.g | K3ZQP1 | TPR | | 2529 | 843 | Chr2 | 45886670-45889398 | 2 | Mitochondria | Upward |
| Si028944m.g | K3ZQR4 | P | | 2463 | 821 | Chr2 | 7220361-7227657 | 10 | Chloroplast | Downward |
| Si028964m.g | K3ZQT4 | PLS | | 2397 | 799 | Chr2 | 16991241-16993871 | 0 | _ | Downward |
| Si028988m.g | K3ZQV8 | PLS | | 2358 | 786 | Chr2 | 34559287-34566759 | 15 | Mitochondria | Downward |
| Si029027m.g | K3ZQZ7 | E | | 2262 | 754 | Chr2 | 48077265-48079529 | 0 | _ | Downward |
| Si029034m.g | K3ZR04 | E | | 2250 | 750 | Chr2 | 47895184-47897436 | 0 | Mitochondria | Upward |
| Si029086m.g | K3ZR56 | PLS | | 2118 | 706 | Chr2 | 4303761-4305962 | 1 | Chloroplast | Upward |
| Si029143m.g | K3ZRB3 | DYW | | 2004 | 668 | Chr2 | 10527551-10531014 | 1 | Chloroplast | Downward |
| Si029147m.g | K3ZRB7 | P | | 2001 | 667 | Chr2 | 42679413-42682817 | 4 | Mitochondria | Downward |
| Si029198m.g | K3ZRG8 | DYW | | 1899 | 633 | Chr2 | 99620-101638 | 0 | Chloroplast | Downward |
| Si029244m.g | K3ZRL4 | P | | 1842 | 614 | Chr2 | 31459963-31468187 | 11 | Chloroplast | Upward |
| Si029246m.g | K3ZRL6 | P | | 1839 | 613 | Chr2 | 48552124-48554650 | 2 | Chloroplast | Downward |
| Si029254m.g | K3ZRM4 | P | | 1830 | 610 | Chr2 | 44978561-44982340 | 5 | Mitochondria | Upward |
| Si029326m.g | K3ZRU6 | P | | 1743 | 581 | Chr2 | 41037454-41040218 | 0 | Mitochondria | Upward |
| Si029354m.g | K3ZRX4 | P | | 1713 | 571 | Chr2 | 570457-575171 | 3 | Chloroplast | Downward |
| Si029422m.g | K3ZS42 | P | | 1632 | 544 | Chr2 | 28727671-28731867 | 4 | Mitochondria | Downward |
| Si029528m.g | K3ZSE8 | P | | 1539 | 513 | Chr2 | 22832099-22833977 | 1 | Chloroplast | Upward |
| Si029658m.g | K3ZSS8 | P | | 1455 | 485 | Chr2 | 45203376-45207120 | 1 | Mitochondria | Upward |
| Si029682m.g | K3ZSV0 | P | | 1443 | 481 | Chr2 | 4725395-4729534 | 1 | C/M | Downward |
| Si029770m.g | K3ZT40 | P | | 1386 | 462 | Chr2 | 41094423-41096791 | 1 | _ | Downward |
| Si029844m.g | K3ZTB4 | P | | 1332 | 444 | Chr2 | 8624636-8626057 | 0 | Chloroplast | Downward |
| Si029918m.g | K3ZTI8 | P | | 1284 | 428 | Chr2 | 7307732-7311044 | 1 | Mitochondria | Upward |
| Si031905m.g | K3ZZ73 | E | | 1617 | 539 | Chr2 | 30276043-30277662 | 0 | Mitochondria | Downward |
| Si031916m.g | K3ZZ84 | P | | 1653 | 551 | Chr2 | 15286590-15288263 | 1 | Chloroplast | Upward |
| Si031917m.g | K3ZZ85 | P | | 1158 | 386 | Chr2 | 38261317-38262474 | 0 | _ | Upward |
| Si031929m.g | K3ZZ97 | P | | 1143 | 381 | Chr2 | 7550538-7551683 | 0 | _ | Downward |
| Si031975m.g | K3ZZE3 | E | | 1692 | 564 | Chr2 | 46324466-46327097 | 1 | Mitochondria | Upward |
| Si032069m.g | K3ZZN4 | E | | 2091 | 697 | Chr2 | 10421943-10424036 | 0 | Chloroplast | Upward |
| Si032081m.g | K3ZZP6 | E | | 1818 | 606 | Chr2 | 26224034-26226213 | 2 | Chloroplast | Upward |
| Si032086m.g | K3ZZQ1 | P | | 1185 | 395 | Chr2 | 47567539-47569127 | 1 | _ | Downward |
| Si032224m.g | K4A038 | DYW | | 1884 | 628 | Chr2 | 35112430-35114316 | 0 | Mitochondria | Upward |
| Si032252m.g | K4A066 | E | | 2007 | 669 | Chr2 | 26826210-26829263 | 3 | Mitochondria | Downward |
| Si032264m.g | K4A077 | P | | 2271 | 757 | Chr2 | 41727014-41729287 | 0 | Mitochondria | Upward |
| Si032304m.g | K4A0B5 | E | | 2094 | 698 | Chr2 | 46585189-46587285 | 0 | Mitochondria | Upward |
| Si032333m.g | K4A0E4 | P | | 1545 | 515 | Chr2 | 11978177-11979724 | 0 | _ | Upward |
| Si032394m.g | K4A0K5 | P | | 1533 | 511 | Chr2 | 44994928-44996463 | 0 | Mitochondria | Downward |
| Si032405m.g | K4A0L5 | P | | 1560 | 520 | Chr2 | 38843964-38845564 | 1 | Mitochondria | Downward |
| Si032416m.g | K4A0M6 | P | | 2466 | 822 | Chr2 | 30435241-30437709 | 0 | Chloroplast | Downward |
| Si032423m.g | K4A0N3 | P | | 1755 | 585 | Chr2 | 9326902-9328999 | 2 | Mitochondria | Downward |
| Si032483m.g | K4A0U2 | DYW | | 1632 | 544 | Chr2 | 34582299-34583964 | 1 | Chloroplast | Upward |
| Si032568m.g | K4A126 | E | | 1929 | 643 | Chr2 | 39554108-39556039 | 0 | Chloroplast | Downward |
| Si032642m.g | K4A199 | DYW | | 1842 | 614 | Chr2 | 46644678-46646522 | 0 | _ | Downward |
| Si032737m.g | K4A1J4 | P | | 1821 | 607 | Chr2 | 45558201-45560021 | 0 | Mitochondria | Upward |
| Si032758m.g | K4A1L4 | DYW | | 2406 | 802 | Chr2 | 44620458-44622866 | 0 | Chloroplast | Downward |
| Si032802m.g | K4A1Q8 | E | | 1938 | 646 | Chr2 | 25213462-25215791 | 1 | C/M | Downward |
| Si032861m.g | K4A1W5 | DYW | | 1857 | 619 | Chr2 | 3425348-3427204 | 0 | _ | Downward |
| Si032871m.g | K4A1X5 | DYW | | 1782 | 594 | Chr2 | 2541148-2542932 | 0 | Mitochondria | Downward |
| Si033103m.g | K4A2K4 | E | | 1518 | 506 | Chr2 | 30398782-30400302 | 0 | Chloroplast | Upward |
| Si033236m.g | K4A2Y4 | P | | 945 | 315 | Chr2 | 31404170-31406058 | 2 | _ | Upward |
| Si033371m.g | K4A3B8 | P | | 2397 | 799 | Chr2 | 44742495-44744894 | 0 | Chloroplast | Upward |
| Si033390m.g | K4A3D7 | P | | 780 | 260 | Chr2 | 42722768-42724669 | 1 | Mitochondria | Downward |
| Si033420m.g | K4A3G7 | DYW | | 2181 | 727 | Chr2 | 8357393-8359576 | 0 | Chloroplast | Downward |
| Si033422m.g | K4A3G9 | P | | 2598 | 866 | Chr2 | 8775061-8777658 | 0 | Mitochondria | Downward |
| Si034006m.g | K4A552 | P | | 3282 | 1094 | Chr9 | 42052172-42055711 | 0 | Chloroplast | Upward |
| Si034130m.g | K4A5H6 | DYW | | 2760 | 920 | Chr9 | 48928469-48932088 | 2 | _ | Downward |
| Si034164m.g | K4A5L0 | P | | 2676 | 892 | Chr9 | 1631163-1635974 | 4 | Chloroplast | Upward |
| Si034185m.g | K4A5N1 | P | | 2631 | 877 | Chr9 | 41361353-41368334 | 9 | Chloroplast | Downward |
| Si034189m.g | K4A5N5 | P | | 2625 | 875 | Chr9 | 14623370-14626112 | 2 | Chloroplast | Downward |
| Si034232m.g | K4A5S8 | P | | 2556 | 852 | Chr9 | 19109544-19112645 | 1 | Mitochondria | Downward |
| Si034277m.g | K4A5X3 | PLS | | 2454 | 818 | Chr9 | 49481994-49487646 | 11 | Chloroplast | Upward |
| Si034307m.g | K4A603 | E | | 2406 | 802 | Chr9 | 42004849-42007257 | 0 | Mitochondria | Upward |
| Si034313m.g | K4A609 | DYW | | 2391 | 797 | Chr9 | 9844384-9846834 | 1 | Mitochondria | Upward |
| Si034314m.g | K4A610 | PLS | | 2385 | 795 | Chr9 | 53611652-53615945 | 1 | Chloroplast | Downward |
| Si034315m.g | K4A611 | E | | 2385 | 795 | Chr9 | 7919628-7922071 | 1 | Mitochondria | Downward |
| Si034333m.g | K4A629 | DYW | | 2322 | 774 | Chr9 | 43519077-43521436 | 0 | _ | Downward |
| Si034336m.g | K4A632 | P | | 2319 | 773 | Chr9 | 22198287-22200936 | 0 | Mitochondria | Upward |
| Si034342m.g | K4A638 | P | | 2310 | 770 | Chr9 | 7321431-7323863 | 0 | Mitochondria | Downward |
| Si034377m.g | K4A673 | E | | 2265 | 755 | Chr9 | 14517301-14520028 | 3 | Chloroplast | Downward |
| Si034391m.g | K4A687 | P | | 2250 | 750 | Chr9 | 12934803-12937128 | 0 | Chloroplast | Upward |
| Si034392m.g | K4A688 | DYW | | 2247 | 749 | Chr9 | 47137893-47140142 | 0 | Chloroplast | Downward |
| Si034454m.g | K4A6F0 | P | | 2151 | 717 | Chr9 | 3293048-3295887 | 1 | Mitochondria | Downward |
| Si034467m.g | K4A6G3 | P | | 2130 | 710 | Chr9 | 301489-304113 | 0 | Chloroplast | Downward |
| Si034471m.g | K4A6G7 | DYW | | 2118 | 706 | Chr9 | 48021358-48023478 | 0 | Mitochondria | Downward |
| Si034478m.g | K4A6H4 | DYW | | 2106 | 702 | Chr9 | 16645234-16647348 | 0 | _ | Upward |
| Si034510m.g | K4A6K5 | P | | 2070 | 690 | Chr9 | 29958008-29962979 | 12 | Chloroplast | Downward |
| Si034513m.g | K4A6K8 | P | | 2067 | 689 | Chr9 | 52863566-52865857 | 0 | Chloroplast | Upward |
| Si034522m.g | K4A6L7 | E | | 2055 | 685 | Chr9 | 41564524-41566821 | 1 | Mitochondria | Upward |
| Si034526m.g | K4A6M1 | E | | 2049 | 683 | Chr9 | 794060-796424 | 0 | Chloroplast | Downward |
| Si034527m.g | K4A6M2 | DYW | | 2046 | 682 | Chr9 | 14456423-14458942 | 0 | Mitochondria | Downward |
| Si034560m.g | K4A6Q5 | Pkinase_Tyr Kdo RIO1 | | 2010 | 670 | Chr9 | 22225351-22229036 | 6 | Mitochondria | Downward |
| Si034581m.g | K4A6S6 | E EXOIII | | 1971 | 657 | Chr9 | 48717259-48721512 | 3 | _ | Downward |
| Si034598m.g | K4A6U3 | E | | 1950 | 650 | Chr9 | 48120003-48122296 | 0 | Mitochondria | Downward |
| Si034599m.g | K4A6U4 | P | | 1950 | 650 | Chr9 | 55475121-55479027 | 3 | Mitochondria | Upward |
| Si034723m.g | K4A767 | P | | 1860 | 620 | Chr9 | 45729581-45731912 | 1 | Mitochondria | Upward |
| Si034731m.g | K4A775 | P | | 1854 | 618 | Chr9 | 12755603-12757522 | 1 | Chloroplast | Downward |
| Si034734m.g | K4A778 | P | | 1848 | 616 | Chr9 | 18276142-18278263 | 1 | Chloroplast | Downward |
| Si034740m.g | K4A784 | DYW | | 1845 | 615 | Chr9 | 1785007-1788666 | 1 | Chloroplast | Upward |
| Si034766m.g | K4A7B0 | DYW | | 1815 | 605 | Chr9 | 3310413-3312373 | 0 | Chloroplast | Downward |
| Si034813m.g | K4A7F7 | P | | 1776 | 592 | Chr9 | 6588734-6590512 | 0 | Mitochondria | Downward |
| Si034848m.g | K4A7J2 | P | | 1749 | 583 | Chr9 | 53180086-53182739 | 1 | Chloroplast | Downward |
| Si034870m.g | K4A7L4 | P | | 1737 | 579 | Chr9 | 15807404-15810491 | 1 | Mitochondria | Upward |
| Si034956m.g | K4A7V0 | P | | 1686 | 562 | Chr9 | 16672382-16674105 | 0 | Chloroplast | Upward |
| Si035003m.g | K4A7Z7 | PLS | | 1656 | 552 | Chr9 | 47071665-47073354 | 0 | Chloroplast | Downward |
| Si035028m.g | K4A822 | P | | 1641 | 547 | Chr9 | 23775889-23778794 | 1 | Mitochondria | Downward |
| Si035086m.g | K4A880 | E | | 1608 | 536 | Chr9 | 55300467-55302077 | 0 | Mitochondria | Upward |
| Si035125m.g | K4A8B9 | E | | 1440 | 480 | Chr9 | 10871326-10872969 | 0 | Mitochondria | Upward |
| Si035127m.g | K4A8C1 | E | | 1587 | 529 | Chr9 | 2903639-2905552 | 0 | C/M | Downward |
| Si035290m.g | K4A8T4 | P | | 1518 | 506 | Chr9 | 23421262-23422782 | 0 | _ | Downward |
| Si035349m.g | K4A8Z3 | P | | 1485 | 495 | Chr9 | 14521841-14523450 | 1 | Mitochondria | Downward |
| Si035359m.g | K4A903 | P | | 1482 | 494 | Chr9 | 14523638-14525837 | 1 | Chloroplast | Upward |
| Si035534m.g | K4A9H8 | P | | 1401 | 467 | Chr9 | 39268193-39269806 | 1 | Mitochondria | Upward |
| Si035805m.g | K4AA98 | E | | 1287 | 429 | Chr9 | 45502449-45504106 | 0 | Mitochondria | Downward |
| Si035856m.g | K4AAE9 | P | | 1257 | 419 | Chr9 | 6153592-6155197 | 0 | Chloroplast | Downward |
| Si035860m.g | K4AAF3 | P | | 1257 | 419 | Chr9 | 57785066-57786760 | 0 | Mitochondria | Downward |
| Si035918m.g | K4AAL1 | P | | 1227 | 409 | Chr9 | 55767105-55768886 | 1 | Mitochondria | Downward |
| Si037184m.g | K4AE77 | P | | 744 | 248 | Chr9 | 47554522-47555395 | 1 | Mitochondria | Upward |
| Si038542m.g | K4AI35 | DYW | | 2091 | 697 | Chr9 | 15458660-15460753 | 0 | Mitochondria | Downward |
| Si038545m.g | K4AI38 | P | | 1203 | 401 | Chr9 | 52836486-52837691 | 0 | _ | Upward |
| Si038646m.g | K4AID5 | E | | 2964 | 988 | Chr9 | 54637294-54640260 | 0 | Chloroplast | Downward |
| Si038662m.g | K4AIF0 | E | | 1935 | 645 | Chr9 | 20681866-20683803 | 0 | Mitochondria | Downward |
| Si038671m.g | K4AIF9 | E | | 1308 | 436 | Chr9 | 423481-424791 | 0 | Mitochondria | Upward |
| Si038752m.g | K4AIP0 | P | | 1761 | 587 | Chr9 | 34987589-34989349 | 0 | Chloroplast | Upward |
| Si038777m.g | K4AIR5 | P | | 1416 | 472 | Chr9 | 58115244-58116758 | 1 | Chloroplast | Upward |
| Si038790m.g | K4AIS8 | E | | 2613 | 871 | Chr9 | 3962505-3965213 | 1 | Mitochondria | Downward |
| Si038792m.g | K4AIT0 | DYW | | 2040 | 680 | Chr9 | 40100133-40102175 | 0 | Mitochondria | Upward |
| Si038813m.g | K4AIV1 | P | | 1539 | 513 | Chr9 | 53323441-53324982 | 0 | Mitochondria | Downward |
| Si038916m.g | K4AJ52 | DYW | | 1605 | 535 | Chr9 | 56571298-56572953 | 1 | Chloroplast | Upward |
| Si038942m.g | K4AJ77 | P | | 1581 | 527 | Chr9 | 35458481-35460391 | 3 | Mitochondria | Upward |
| Si039048m.g | K4AJH8 | P | | 1500 | 500 | Chr9 | 14204529-14206031 | 0 | Mitochondria | Downward |
| Si039087m.g | K4AJL6 | P | | 1485 | 495 | Chr9 | 6219173-6220660 | 0 | Chloroplast | Upward |
| Si039115m.g | K4AJP4 | DYW | | 2409 | 803 | Chr9 | 4239931-4242342 | 0 | C/M | Upward |
| Si039136m.g | K4AJR4 | E | | 1497 | 499 | Chr9 | 18642619-18644118 | 0 | Chloroplast | Downward |
| Si039179m.g | K4AJV7 | PLS | | 1461 | 487 | Chr9 | 4880921-4882384 | 0 | Mitochondria | Downward |
| Si039288m.g | K4AK65 | P | | 1587 | 529 | Chr9 | 36437193-36438782 | 0 | _ | Downward |
| Si039323m.g | K4AKA0 | E | | 2583 | 861 | Chr9 | 40030598-40033183 | 0 | Mitochondria | Upward |
| Si039389m.g | K4AKG6 | P | | 1314 | 438 | Chr9 | 34488758-34490074 | 0 | _ | Upward |
| Si039392m.g | K4AKG9 | P | | 2055 | 685 | Chr9 | 58899438-58901785 | 2 | Mitochondria | Upward |
| Si039399m.g | K4AKH6 | P | | 1812 | 604 | Chr9 | 14049632-14052284 | 5 | _ | Upward |
| Si039439m.g | K4AKL6 | DYW | | 1908 | 636 | Chr9 | 51812774-51814684 | 0 | Mitochondria | Downward |
| Si039489m.g | K4AKR4 | P | | 1629 | 543 | Chr9 | 18250263-18252548 | 8 | Mitochondria | Downward |
| Si039571m.g | K4AKZ2 | P | | 750 | 250 | Chr9 | 57012365-57013927 | 1 | Mitochondria | Downward |
| Si039582m.g | K4AL03 | P | | 2427 | 809 | Chr9 | 16374705-16377654 | 1 | Mitochondria | Downward |
| Si039649m.g | K4AL69 | E | | 1788 | 596 | Chr9 | 49543178-49544968 | 0 | _ | Upward |
| Si039650m.g | K4AL70 | E | | 2163 | 721 | Chr9 | 448850-451015 | 0 | Chloroplast | Downward |
| Si039680m.g | K4ALA0 | E | | 1791 | 597 | Chr9 | 1296780-1298714 | 2 | Mitochondria | Upward |
| Si039699m.g | K4ALB9 | E | | 1518 | 506 | Chr9 | 55266815-55268335 | 0 | _ | Downward |
| Si039712m.g | K4ALD2 | E | | 1560 | 520 | Chr9 | 36945095-36946654 | 0 | Mitochondria | Upward |
| Si039802m.g | K4ALM1 | E | | 1425 | 475 | Chr9 | 54588341-54589768 | 0 | _ | Upward |
| Si039892m.g | K4ALV7 | P | | 1563 | 521 | Chr9 | 14652507-14654795 | 2 | Mitochondria | Downward |
| Si039898m.g | K4ALW3 | E | | 1962 | 654 | Chr9 | 55560427-55562391 | 0 | Mitochondria | Downward |
| Si039925m.g | K4ALZ0 | P | | 3057 | 1019 | Chr9 | 11769677-11773916 | 3 | Mitochondria | Downward |
| Si039943m.g | K4AM08 | DYW | | 1980 | 660 | Chr9 | 51516579-51518558 | 0 | _ | Upward |
| Si040060m.g | K4AMC1 | P | | 1545 | 515 | Chr9 | 52839527-52841623 | 2 | _ | Downward |
| Si040151m.g | K4AMK7 | E | | 2646 | 882 | Chr9 | 39975363-39978008 | 0 | _ | Upward |
| Si040217m.g | K4AMS1 | P | | 1671 | 557 | Chr9 | 5713600-5715273 | 0 | Mitochondria | Upward |
| Si040248m.g | K4AMV2 | P | | 3777 | 1259 | Chr9 | 55307840-55311706 | 1 | Mitochondria | Downward |
| Si040273m.g | K4AMX7 | E | | 1374 | 458 | Chr9 | 50686139-50687515 | 0 | Mitochondria | Upward |
| Si040278m.g | K4AMY2 | P | | 1959 | 653 | Chr9 | 123793-125823 | 2 | Mitochondria | Downward |
| Si040279m.g | K4AMY3 | P | | 2280 | 760 | Chr9 | 952102-955147 | 1 | Mitochondria | Upward |
| Si040284m.g | K4AMY8 | E | | 1809 | 603 | Chr9 | 5839844-5841655 | 0 | Mitochondria | Downward |
| Si040465m.g | K4ANG8 | P | | 183 | 61 | Chr9 | 14722969-14723350 | 0 | _ | Upward |
| Si040503m.g | K4ANK6 | P | | 657 | 219 | Chr9 | 15739697-15740879 | 0 | _ | Upward |
| Si040868m.g | K4APM0 | P | | 816 | 272 | _ | scaffold_43:12987-13805 | 0 | Mitochondria | Upward |
|  |  |  |  |  | |  |  |  |  |  |
