# Supplementary material for: Genome-wide investigation and expression analyses of the pentatricopeptide repeat protein gene family in foxtail millet
Source: BMC Genomics. 2016 Oct 28;17:840. doi: 10.1186/s12864-016-3184-2 (PMC5084403; doi:10.1186/s12864-016-3184-2)
Supplement: Additional file 2: Table S2. — PPR genes in Selaginella. The domain/class, open reading frame length, predicted protein length, chromosomal location, number of introns within the ORF, and the subcellular localization of the PPR proteins of each of these genes are listed. (DOCX 194 kb) [file 12864_2016_3184_MOESM2_ESM.docx]

**Table S2.** PPR protein encoding genes in Selaginella. The domain/class, open reading frame length, protein length, chromosomal location, number of introns within ORF and the subcellular localization of the PPR proteins of each of these genes were listed.

| **Gene names** | | **Protein ID** | | **SMART/PfamA Domain/Class** | | **ORF length** | | **Protein Length** | | | **Genomic location (strand)** | | **Introns NO.** | | **Location** | |  |
| --- | --- | --- | --- | --- | --- | --- | --- | --- | --- | --- | --- | --- | --- | --- | --- | --- | --- |
| SELMODRAFT_10002 | | D8RW64 | | P | | 2190 | | 730 | | | GL377592:1176260-1178668 | | 1 | | _ | |  |
| SELMODRAFT_100028 | | D8RSQ0 | | P | | 1659 | | 553 | | | GL377588:1719002-1720767 | | 1 | | _ | |  |
| SELMODRAFT_100071 | | D8RS31 | | P | | 1026 | | 342 | | | GL377588:510215-511240 | | 0 | | _ | |  |
| SELMODRAFT_100125 | | D8RRS0 | | P | | 1563 | | 521 | | | GL377588:3974-5740 | | 1 | | _ | |  |
| SELMODRAFT_100166 | | D8RSS0 | | P | | 1152 | | 384 | | | GL377588:1784604-1785875 | | 1 | | _ | |  |
| SELMODRAFT_100216 | | D8RSR5 | | DYW | | 2103 | | 701 | | | GL377588:1773528-1776197 | | 3 | | Mitochondria | |  |
| SELMODRAFT_100264 | | D8RSC5 | | P | | 1197 | | 399 | | | GL377588:1154939-1156138 | | 0 | | _ | |  |
| SELMODRAFT_100308 | | D8RS66 | | P | | 1212 | | 404 | | | GL377588:800877-803120 | | 1 | | _ | |  |
| SELMODRAFT_100342 | | D8RSM6 | | P | | 696 | | 232 | | | GL377588:1594233-1594931 | | 0 | | _ | |  |
| SELMODRAFT_100350 | | D8RS25 | | P | | 1479 | | 493 | | | GL377588:486607-488262 | | 1 | | Mitochondria | |  |
| SELMODRAFT_100388 | | D8RS44 | | P | | 969 | | 323 | | | GL377588:563213-564184 | | 0 | | _ | |  |
| SELMODRAFT_100394 | | D8RRW3 | | P | | 1683 | | 561 | | | GL377588:144252-145937 | | 0 | | Mitochondria | |  |
| SELMODRAFT_100448 | | D8RSR8 | | P | | 525 | | 175 | | | GL377588:1781828-1782355 | | 0 | | _ | |  |
| SELMODRAFT_100511 | | D8RSJ6 | | DYW | | 1395 | | 465 | | | GL377588:1492763-1495870 | | 1 | | _ | |  |
| SELMODRAFT_100620 | | D8RRW5 | | P | | 1692 | | 564 | | | GL377588:149981-151954 | | 2 | | _ | |  |
| SELMODRAFT_100621 | | D8RS73 | | E | | 1848 | | 616 | | | GL377588:825289-827643 | | 1 | | Mitochondria | |  |
| SELMODRAFT_100651 | | D8RSD3 | | P | | 576 | | 192 | | | GL377588:1202488-1204395 | | 1 | | _ | |  |
| SELMODRAFT_10066 | | D8RW09 | | P | | 882 | | 294 | | | GL377592:310633-311514 | | 0 | | _ | |  |
| SELMODRAFT_100681 | | D8RSJ0 | | P | | 708 | | 236 | | | GL377588:1478543-1479361 | | 1 | | _ | |  |
| SELMODRAFT_100719 | | D8RS60 | | P | | 489 | | 163 | | | GL377588:679823-684676 | | 2 | | _ | |  |
| SELMODRAFT_100758 | | D8RSN1 | | P | | 2469 | | 823 | | | GL377588:1637049-1639520 | | 0 | | Chloroplast | |  |
| SELMODRAFT_100773 | | D8RSJ9 | | P | | 1614 | | 538 | | | GL377588:1499005-1500621 | | 0 | | _ | |  |
| SELMODRAFT_100779 | | D8RSW1 | | P | | 795 | | 265 | | | GL377588:1940904-1941698 | | 0 | | _ | |  |
| SELMODRAFT_100785 | | D8RSQ6 | | P | | 660 | | 220 | | | GL377588:1744724-1745851 | | 1 | | _ | |  |
| SELMODRAFT_100795 | | D8RRU0 | | P | | 723 | | 241 | | | GL377588:65573-66298 | | 0 | | _ | |  |
| SELMODRAFT_100825 | | D8RSS1 | | P | | 1941 | | 647 | | | GL377588:1787326-1790978 | | 1 | | _ | |  |
| SELMODRAFT_100938 | | D8RTL6 | | P | | 1146 | | 382 | | | GL377589:1561443-1562909 | | 1 | | Mitochondria | |  |
| SELMODRAFT_101 | | D8RUC7 | | P | | 2763 | | 921 | | | GL377590:1670813-1673575 | | 0 | | _ | |  |
| SELMODRAFT_101075 | | D8RTS6 | | P | | 1701 | | 567 | | | GL377589:1878327-1880105 | | 0 | | _ | |  |
| SELMODRAFT_101093 | | D8RTR3 | | DYW | | 1845 | | 615 | | | GL377589:1801058-1803820 | | 2 | | _ | |  |
| SELMODRAFT_101239 | | D8RT43 | | DYW | | 2037 | | 679 | | | GL377589:519315-521747 | | 2 | | Mitochondria | |  |
| SELMODRAFT_101245 | | D8RTN7 | | P | | 1143 | | 381 | | | GL377589:1690348-1692006 | | 1 | | _ | |  |
| SELMODRAFT_101316 | | D8RTN2 | | E | | 837 | | 279 | | | GL377589:1673352-1674191 | | 0 | | _ | |  |
| SELMODRAFT_101336 | | D8RTE9 | | P | | 990 | | 330 | | | GL377589:1215782-1217500 | | 1 | | _ | |  |
| SELMODRAFT_101455 | | D8RTR2 | | DYW | | 1905 | | 635 | | | GL377589:1798613-1800811 | | 1 | | Mitochondria | |  |
| SELMODRAFT_101474 | | D8RT97 | | P | | 1092 | | 364 | | | GL377589:860547-863246 | | 1 | | Mitochondria | |  |
| SELMODRAFT_101547 | | D8RT39 | | P | | 822 | | 274 | | | GL377589:507700-509073 | | 1 | | _ | |  |
| SELMODRAFT_101549 | | D8RT56 | | P | | 1359 | | 453 | | | GL377589:673168-674916 | | 2 | | _ | |  |
| SELMODRAFT_101588 | | D8RUD6 | | E | | 759 | | 253 | | | GL377590:1701149-1702093 | | 0 | | _ | |  |
| SELMODRAFT_101593 | | D8RUK0 | | P | | 324 | | 108 | | | GL377590:109699-110022 | | 0 | | Chloroplast | |  |
| SELMODRAFT_101726 | | D8RUD3 | | P | | 1035 | | 345 | | | GL377590:1693700-1696491 | | 1 | | _ | |  |
| SELMODRAFT_101730 | | D8RUC5 | | P | | 582 | | 194 | | | GL377590:1667801-1668385 | | 0 | | _ | |  |
| SELMODRAFT_101849 | | D8RU36 | | P | | 579 | | 193 | | | GL377590:1285283-1285948 | | 1 | | SP | |  |
| SELMODRAFT_101881 | | D8RUS2 | | P | | 1485 | | 495 | | | GL377590:664327-665814 | | 0 | | _ | |  |
| SELMODRAFT_101941 | | D8QWM3 | | P | | 477 | | 159 | | | GL377590:1812679-1813158 | | 0 | | _ | |  |
| SELMODRAFT_102001 | | D8RUS1 | | PLS | | 1740 | | 580 | | | GL377590:651712-661542 | | 2 | | _ | |  |
| SELMODRAFT_102049 | | D8RU11 | | P | | 942 | | 314 | | | GL377590:1122920-1123864 | | 0 | | _ | |  |
| SELMODRAFT_102226 | | D8RUL1 | | P | | 1233 | | 411 | | | GL377590:152707-154308 | | 2 | | _ | |  |
| SELMODRAFT_102230 | | D8RU34 | | P | | 642 | | 214 | | | GL377590:1280911-1281555 | | 0 | | SP | |  |
| SELMODRAFT_102231 | | D8RU82 | | P | | 708 | | 236 | | | GL377590:1510122-1513213 | | 1 | | _ | |  |
| SELMODRAFT_102386 | | D8RVL0 | | P | | 1149 | | 383 | | | GL377591:1378284-1380062 | | 1 | | _ | |  |
| SELMODRAFT_102413 | | D8RVN5 | | P | | 741 | | 247 | | | GL377591:1452073-1452813 | | 0 | | Mitochondria | |  |
| SELMODRAFT_102435 | | D8RVP8 | | DYW | | 2415 | | 805 | | | GL377591:1509300-1511717 | | 0 | | Mitochondria | |  |
| SELMODRAFT_102503 | | D8RVG2 | | P | | 642 | | 214 | | | GL377591:1134132-1134776 | | 0 | | Mitochondria | |  |
| SELMODRAFT_102603 | | D8RV63 | | DYW | | 1455 | | 485 | | | GL377591:582516-583973 | | 0 | | _ | |  |
| SELMODRAFT_102729 | | D8RV90 | | E | | 1212 | | 404 | | | GL377591:766369-768126 | | 1 | | _ | |  |
| SELMODRAFT_102968 | | D8RVR6 | | P | | 627 | | 209 | | | GL377591:1568284-1568913 | | 0 | | _ | |  |
| SELMODRAFT_103065 | | D8RWJ2 | | P | | 1983 | | 661 | | | GL377592:1893988-1897632 | | 4 | | Mitochondria | |  |
| SELMODRAFT_103099 | | D8RWG4 | | P | | 1368 | | 456 | | | GL377592:1762130-1763713 | | 1 | | _ | |  |
| SELMODRAFT_10313 | | D8SDV7 | | P | | 888 | | 296 | | | GL377614:646858-670937 | | 1 | | _ | |  |
| SELMODRAFT_103221 | | D8RWK6 | | P | | 486 | | 162 | | | GL377592:1950899-1951387 | | 0 | | _ | |  |
| SELMODRAFT_103241 | | D8RW63 | | P | | 927 | | 309 | | | GL377592:1173026-1175764 | | 1 | | _ | |  |
| SELMODRAFT_103302 | | D8RW08 | | P | | 783 | | 261 | | | GL377592:309118-309900 | | 0 | | SP | |  |
| SELMODRAFT_103455 | | D8RWZ4 | | E | | 2034 | | 678 | | | GL377593:780607-782643 | | 0 | | _ | |  |
| SELMODRAFT_103498 | | D8RX33 | | P | | 1641 | | 547 | | | GL377593:970672-972924 | | 2 | | _ | |  |
| SELMODRAFT_103577 | | D8RXK6 | | P | | 1386 | | 462 | | | GL377593:1956698-1958752 | | 1 | | _ | |  |
| SELMODRAFT_103597 | | D8RXD2 | | P | | 321 | | 107 | | | GL377593:1596300-1596620 | | 0 | | _ | |  |
| SELMODRAFT_103638 | | D8RXA1 | | P | | 1413 | | 471 | | | GL377593:1418311-1420786 | | 5 | | Mitochondria | |  |
| SELMODRAFT_103748 | | D8RXG1 | | P | | 1317 | | 439 | | | GL377593:1698849-1700168 | | 0 | | Mitochondria | |  |
| SELMODRAFT_103939 | | D8RWN6 | | P | | 1125 | | 375 | | | GL377593:141564-142895 | | 1 | | _ | |  |
| SELMODRAFT_103955 | | D8RX91 | | P | | 1527 | | 509 | | | GL377593:1375486-1377585 | | 1 | | Mitochondria | |  |
| SELMODRAFT_104027 | | D8RWZ6 | | P | | 582 | | 194 | | | GL377593:784314-785345 | | 1 | | Mitochondria | |  |
| SELMODRAFT_104103 | | D8RXS5 | | DYW | | 2037 | | 679 | | | GL377594:701120-703449 | | 1 | | Mitochondria | |  |
| SELMODRAFT_104134 | | D8RYL0 | | P | | 459 | | 153 | | | GL377594:123883-124344 | | 0 | | _ | |  |
| SELMODRAFT_104228 | | D8RYJ1 | | P | | 1485 | | 495 | | | GL377594:49591-51975 | | 1 | | _ | |  |
| SELMODRAFT_10445 | | D8STS5 | | P | | 1539 | | 513 | | | GL377640:934633-937587 | | 2 | | _ | |  |
| SELMODRAFT_104474 | | D8RXS1 | | DYW | | 1995 | | 665 | | | GL377594:680528-682717 | | 1 | | _ | |  |
| SELMODRAFT_10449 | | D8SVD5 | | P | | 1773 | | 591 | | | GL377645:543964-546021 | | 1 | | _ | |  |
| SELMODRAFT_104499 | | D8RXV6 | | P | | 969 | | 323 | | | GL377594:820555-821526 | | 0 | | _ | |  |
| SELMODRAFT_104710 | | D8RXS6 | | P | | 2097 | | 699 | | | GL377594:703558-707178 | | 2 | | SP | |  |
| SELMODRAFT_104790 | | D8RZ37 | | P | | 561 | | 187 | | | GL377595:877597-878160 | | 0 | | _ | |  |
| SELMODRAFT_104848 | | D8RZB7 | | P | | 1113 | | 371 | | | GL377595:1435733-1438270 | | 2 | | SP | |  |
| SELMODRAFT_105033 | | D8RZD4 | | P | | 2859 | | 953 | | | GL377595:1729891-1732752 | | 0 | | _ | |  |
| SELMODRAFT_105057 | | D8RYY0 | | P | | 684 | | 228 | | | GL377595:550652-552913 | | 2 | | Mitochondria | |  |
| SELMODRAFT_105138 | | D8RZC0 | | E | | 1326 | | 442 | | | GL377595:1463990-1465872 | | 1 | | _ | |  |
| SELMODRAFT_105197 | | D8RYP4 | | P | | 1626 | | 542 | | | GL377595:70700-72922 | | 2 | | _ | |  |
| SELMODRAFT_105248 | | D8S084 | | P | | 2007 | | 669 | | | GL377596:1627503-1629512 | | 0 | | Chloroplast | |  |
| SELMODRAFT_105296 | | D8S026 | | P | | 678 | | 226 | | | GL377596:1332906-1334261 | | 2 | | _ | |  |
| SELMODRAFT_105356 | | D8RZG7 | | P | | 2202 | | 734 | | | GL377596:233615-238468 | | 2 | | _ | |  |
| SELMODRAFT_105472 | | D8S073 | | P | | 1842 | | 614 | | | GL377596:1552646-1554490 | | 0 | | _ | |  |
| SELMODRAFT_105614 | | D8RZL8 | | P | | 1023 | | 341 | | | GL377596:483511-485439 | | 1 | | SP | |  |
| SELMODRAFT_10568 | | D8RQT4 | | P | | 1872 | | 624 | | | GL377586:583228-585387 | | 1 | | Mitochondria | |  |
| SELMODRAFT_105728 | | D8RZK2 | | E | | 552 | | 184 | | | GL377596:367867-368421 | | 0 | | _ | |  |
| SELMODRAFT_105729 | | D8S0D6 | | P | | 1185 | | 395 | | | GL377597:73906-75396 | | 1 | | _ | |  |
| SELMODRAFT_105776 | | D8S0S3 | | P | | 852 | | 284 | | | GL377597:1349930-1351786 | | 1 | | _ | |  |
| SELMODRAFT_105818 | | D8S0I2 | | P | | 924 | | 308 | | | GL377597:371886-373437 | | 1 | | _ | |  |
| SELMODRAFT_105853 | | D8S0G9 | | P | | 1908 | | 636 | | | GL377597:301784-303808 | | 1 | | _ | |  |
| SELMODRAFT_106081 | | D8S0M1 | | DYW | | 1896 | | 632 | | | GL377597:688998-691181 | | 1 | | Mitochondria | |  |
| SELMODRAFT_106096 | | D8S0L1 | | DYW | | 3324 | | 1108 | | | GL377597:637926-641252 | | 0 | | Mitochondria | |  |
| SELMODRAFT_106129 | | D8S0Z5 | | P | | 2112 | | 704 | | | GL377597:1700566-1703034 | | 1 | | Mitochondria | |  |
| SELMODRAFT_106227 | | D8S1H7 | | P | | 681 | | 227 | | | GL377598:854702-856975 | | 1 | | _ | |  |
| SELMODRAFT_106265 | | D8S1K7 | | P | | 1542 | | 514 | | | GL377598:1008017-1010401 | | 2 | | Mitochondria | |  |
| SELMODRAFT_10629 | | D8RKK8 | | E | | 1647 | | 549 | | | GL377582:1510659-1512395 | | 1 | | _ | |  |
| SELMODRAFT_106415 | | D8S1P8 | | P | | 1494 | | 498 | | | GL377598:1265109-1266671 | | 1 | | _ | |  |
| SELMODRAFT_106523 | | D8R0U3 | | P | | 576 | | 192 | | | GL377598:60067-60645 | | 0 | | _ | |  |
| SELMODRAFT_106533 | | D8S160 | | P | | 987 | | 329 | | | GL377598:226307-227833 | | 3 | | _ | |  |
| SELMODRAFT_106617 | | D8S1F7 | | P | | 558 | | 186 | | | GL377598:772500-774998 | | 1 | | _ | |  |
| SELMODRAFT_106624 | | D8S164 | | P | | 2649 | | 883 | | | GL377598:239501-243067 | | 2 | | Mitochondria | |  |
| SELMODRAFT_106630 | | D8S155 | | E | | 2418 | | 806 | | | GL377598:211276-213696 | | 0 | | Mitochondria | |  |
| SELMODRAFT_106687 | | D8S1T7 | | P | | 1017 | | 339 | | | GL377599:592571-593590 | | 0 | | _ | |  |
| SELMODRAFT_106750 | | D8S1Z1 | | E | | 1635 | | 545 | | | GL377599:898379-900442 | | 2 | | SP | |  |
| SELMODRAFT_106765 | | D8S2B0 | | P | | 1206 | | 402 | | | GL377599:1550405-1551838 | | 1 | | SP | |  |
| SELMODRAFT_106776 | | D8S2F3 | | DYW | | 2415 | | 805 | | | GL377599:1680206-1682623 | | 0 | | Mitochondria | |  |
| SELMODRAFT_106830 | | D8S2E0 | | P | | 741 | | 247 | | | GL377599:1624388-1625128 | | 0 | | Mitochondria | |  |
| SELMODRAFT_106857 | | D8S2J0 | | P | | 429 | | 143 | | | GL377599:18768-20222 | | 1 | | Mitochondria | |  |
| SELMODRAFT_106861 | | D8S258 | | P | | 1032 | | 344 | | | GL377599:1254391-1255989 | | 1 | | Mitochondria | |  |
| SELMODRAFT_106883 | | D8S2L3 | | E | | 1095 | | 365 | | | GL377599:93977-95353 | | 1 | | _ | |  |
| SELMODRAFT_106991 | | D8S2Q8 | | P | | 615 | | 205 | | | GL377599:486254-487147 | | 1 | | Mitochondria | |  |
| SELMODRAFT_107109 | | D8S1X7 | | P | | 1272 | | 424 | | | GL377599:821682-823511 | | 1 | | SP | |  |
| SELMODRAFT_107186 | | D8S1S5 | | P | | 1908 | | 636 | | | GL377599:517690-519600 | | 0 | | _ | |  |
| SELMODRAFT_10719 | | D8SIL3 | | P | | 1677 | | 559 | | | GL377622:59820-61700 | | 1 | | Mitochondria | |  |
| SELMODRAFT_107192 | | D8S1W0 | | DYW | | 1956 | | 652 | | | GL377599:702671-724899 | | 1 | | _ | |  |
| SELMODRAFT_107240 | | D8S2Q9 | | P | | 1344 | | 448 | | | GL377599:487109-488455 | | 0 | | _ | |  |
| SELMODRAFT_107260 | | D8S210 | | P | | 1104 | | 368 | | | GL377599:1024193-1025299 | | 0 | | SP | |  |
| SELMODRAFT_107264 | | D8S264 | | P | | 642 | | 214 | | | GL377599:1295538-1296182 | | 0 | | _ | |  |
| SELMODRAFT_107283 | | D8S276 | | P | | 1665 | | 555 | | | GL377599:1325578-1327245 | | 0 | | _ | |  |
| SELMODRAFT_107304 | | D8S1V5 | | DYW | | 2019 | | 673 | | | GL377599:646085-648106 | | 0 | | _ | |  |
| SELMODRAFT_107359 | | D8S380 | | DYW | | 1392 | | 464 | | | GL377600:939441-940835 | | 0 | | _ | |  |
| SELMODRAFT_107423 | | D8S3B8 | | P | | 603 | | 201 | | | GL377600:1132822-1133424 | | 0 | | _ | |  |
| SELMODRAFT_107453 | | D8S369 | | P | | 1455 | | 485 | | | GL377600:865631-867454 | | 2 | | _ | |  |
| SELMODRAFT_107480 | | D8S3B7 | | P | | 702 | | 234 | | | GL377600:1131157-1131861 | | 0 | | _ | |  |
| SELMODRAFT_107490 | | D8S3G5 | | P | | 1002 | | 334 | | | GL377600:1332981-1333985 | | 0 | | _ | |  |
| SELMODRAFT_107592 | | D8S390 | | P | | 1635 | | 545 | | | GL377600:1000578-1002416 | | 1 | | Mitochondria | |  |
| SELMODRAFT_107700 | | D8S2S8 | | DYW | | 2637 | | 879 | | | GL377600:40447-43086 | | 0 | | _ | |  |
| SELMODRAFT_107734 | | D8S3A8 | | P | | 1500 | | 500 | | | GL377600:1100586-1102088 | | 0 | | SP | |  |
| SELMODRAFT_107819 | | D8S3A5 | | P | | 1155 | | 385 | | | GL377600:1052471-1053628 | | 0 | | Chloroplast | |  |
| SELMODRAFT_107843 | | D8S2Z2 | | P | | 1164 | | 388 | | | GL377600:452613-453950 | | 1 | | _ | |  |
| SELMODRAFT_107844 | | D8S3J7 | | P | | 1029 | | 343 | | | GL377600:1566923-1567954 | | 0 | | Mitochondria | |  |
| SELMODRAFT_107947 | | D8S3R1 | | P | | 588 | | 196 | | | GL377601:90183-90773 | | 0 | | _ | |  |
| SELMODRAFT_108052 | | D8S4G2 | | E | | 1011 | | 337 | | | GL377601:1370485-1372101 | | 1 | | _ | |  |
| SELMODRAFT_108163 | | D8S3P6 | | P | | 576 | | 192 | | | GL377601:38477-39973 | | 1 | | _ | |  |
| SELMODRAFT_108199 | | D8S3R4 | | P | | 2085 | | 695 | | | GL377601:98459-100969 | | 2 | | SP | |  |
| SELMODRAFT_10825 | | D8RVH5 | | P | | 1665 | | 555 | | | GL377591:1163125-1164789 | | 0 | | Mitochondria | |  |
| SELMODRAFT_108285 | | D8S493 | | P | | 996 | | 332 | | | GL377601:1070463-1071461 | | 0 | | Mitochondria | |  |
| SELMODRAFT_108325 | | D8S3V4 | | DYW | | 1839 | | 613 | | | GL377601:292396-294441 | | 1 | | _ | |  |
| SELMODRAFT_108400 | | D8S4P5 | | E | | 1644 | | 548 | | | GL377601:1701272-1702918 | | 0 | | _ | |  |
| SELMODRAFT_108408 | | D8S470 | | DYW | | 1680 | | 560 | | | GL377601:967700-969382 | | 0 | | Mitochondria | |  |
| SELMODRAFT_108616 | | D8S5F3 | | DYW | | 2697 | | 899 | | | GL377602:1479040-1486868 | | 4 | | _ | |  |
| SELMODRAFT_108652 | | D8S526 | | DYW | | 2061 | | 687 | | | GL377602:788311-790671 | | 1 | | SP | |  |
| SELMODRAFT_108697 | | D8S4U6 | | P | | 1290 | | 430 | | | GL377602:351595-353673 | | 2 | | Mitochondria | |  |
| SELMODRAFT_108734 | | D8S5A9 | | P | | 1425 | | 475 | | | GL377602:1302658-1304178 | | 1 | | _ | |  |
| SELMODRAFT_108836 | | D8S5C3 | | P | | 681 | | 227 | | | GL377602:1391867-1392817 | | 1 | | _ | |  |
| SELMODRAFT_108854 | | D8S4Z6 | | E | | 1767 | | 589 | | | GL377602:572801-579664 | | 1 | | Mitochondria | |  |
| SELMODRAFT_108969 | | D8S4S0 | | P | | 1932 | | 644 | | | GL377602:154478-156529 | | 1 | | Mitochondria | |  |
| SELMODRAFT_109016 | | D8S5A5 | | P | | 2355 | | 785 | | | GL377602:1260226-1262688 | | 1 | | Mitochondria | |  |
| SELMODRAFT_109195 | | D8S661 | | P | | 1044 | | 348 | | | GL377603:1478998-1480044 | | 0 | | _ | |  |
| SELMODRAFT_109214 | | D8S5M0 | | DYW | | 1287 | | 429 | | | GL377603:37707-39248 | | 1 | | _ | |  |
| SELMODRAFT_109283 | | D8S5M6 | | DYW | | 1314 | | 438 | | | GL377603:55779-57095 | | 0 | | Mitochondria | |  |
| SELMODRAFT_109285 | | D8S5L7 | | E | | 1077 | | 359 | | | GL377603:24018-25913 | | 1 | | _ | |  |
| SELMODRAFT_109332 | | D8S5W7 | | P | | 1014 | | 338 | | | GL377603:842119-843885 | | 2 | | _ | |  |
| SELMODRAFT_109426 | | D8S5R0 | | P | | 810 | | 270 | | | GL377603:595785-597869 | | 2 | | _ | |  |
| SELMODRAFT_109608 | | D8S5Q5 | | P | | 2139 | | 713 | | | GL377603:574885-577500 | | 9 | | _ | |  |
| SELMODRAFT_109632 | | D8S5Q4 | | P | | 1563 | | 521 | | | GL377603:569523-571386 | | 5 | | _ | |  |
| SELMODRAFT_109709 | | D8S6M1 | | P | | 657 | | 219 | | | GL377604:171335-172927 | | 1 | | _ | |  |
| SELMODRAFT_109721 | | D8S6E8 | | DYW | | 2844 | | 948 | | | GL377604:1443722-1450014 | | 3 | | Mitochondria | |  |
| SELMODRAFT_109727 | | D8S6H0 | | P | | 2010 | | 670 | | | GL377604:1550750-1552762 | | 0 | | _ | |  |
| SELMODRAFT_109758 | | D8S6D2 | | DYW | | 954 | | 318 | | | GL377604:1374489-1375445 | | 0 | | SP | |  |
| SELMODRAFT_109820 | | D8S6P4 | | P | | 426 | | 142 | | | GL377604:317443-317871 | | 0 | | SP | |  |
| SELMODRAFT_109908 | | D8S6R9 | | PLS | | 1500 | | 500 | | | GL377604:418613-420115 | | 0 | | _ | |  |
| SELMODRAFT_109921 | | D8S6W5 | | P | | 465 | | 155 | | | GL377604:735631-737202 | | 1 | | _ | |  |
| SELMODRAFT_109988 | | D8S6P7 | | P | | 1389 | | 463 | | | GL377604:321734-332742 | | 1 | | SP | |  |
| SELMODRAFT_110016 | | D8S6N3 | | P | | 534 | | 178 | | | GL377604:274507-275043 | | 0 | | _ | |  |
| SELMODRAFT_110121 | | D8S6S8 | | P | | 900 | | 300 | | | GL377604:527478-528380 | | 0 | | _ | |  |
| SELMODRAFT_11024 | | D8R6Y3 | | P | | 1452 | | 484 | | | GL377573:269983-271554 | | 2 | | _ | |  |
| SELMODRAFT_110342 | | D8S7H4 | | P | | 834 | | 278 | | | GL377605:1054961-1055797 | | 0 | | _ | |  |
| SELMODRAFT_110409 | | D8S7H7 | | P | | 1287 | | 429 | | | GL377605:1065753-1067042 | | 0 | | SP | |  |
| SELMODRAFT_110457 | | D8S7T3 | | P | | 2106 | | 702 | | | GL377605:1690209-1692422 | | 1 | | Mitochondria | |  |
| SELMODRAFT_110462 | | D8S7B3 | | P | | 1098 | | 366 | | | GL377605:658226-659524 | | 1 | | _ | |  |
| SELMODRAFT_110531 | | D8S7C5 | | P | | 561 | | 187 | | | GL377605:714561-716331 | | 1 | | _ | |  |
| SELMODRAFT_110549 | | D8S7R1 | | P | | 1377 | | 459 | | | GL377605:1575287-1577299 | | 1 | | _ | |  |
| SELMODRAFT_110559 | | D8S7C0 | | P | | 513 | | 171 | | | GL377605:687017-687532 | | 0 | | Mitochondria | |  |
| SELMODRAFT_11063 | | D8SQC2 | | P | | 897 | | 299 | | | GL377633:604776-606302 | | 1 | | _ | |  |
| SELMODRAFT_110655 | | D8S7K9 | | P | | 3429 | | 1143 | | | GL377605:1291797-1295663 | | 3 | | Chloroplast | |  |
| SELMODRAFT_110780 | | D8S808 | | DYW | | 1911 | | 637 | | | GL377606:343726-345996 | | 1 | | _ | |  |
| SELMODRAFT_110838 | | D8S8F5 | | DYW | | 2637 | | 879 | | | GL377606:1571259-1573898 | | 0 | | _ | |  |
| SELMODRAFT_110868 | | D8S899 | | P | | 930 | | 310 | | | GL377606:1065827-1066759 | | 0 | | _ | |  |
| SELMODRAFT_110876 | | D8S804 | | P | | 711 | | 237 | | | GL377606:325318-326610 | | 1 | | _ | |  |
| SELMODRAFT_11088 | | D8S4A9 | | P | | 1497 | | 499 | | | GL377601:1167006-1168802 | | 1 | | _ | |  |
| SELMODRAFT_110980 | | D8S7X1 | | P | | 1026 | | 342 | | | GL377606:136096-138642 | | 1 | | _ | |  |
| SELMODRAFT_111075 | | D8S823 | | E | | 2067 | | 689 | | | GL377606:427133-438985 | | 4 | | SP | |  |
| SELMODRAFT_111096 | | D8S7Z9 | | P | | 1494 | | 498 | | | GL377606:299873-301627 | | 1 | | _ | |  |
| SELMODRAFT_111143 | | D8S872 | | P | | 768 | | 256 | | | GL377606:825362-826405 | | 1 | | _ | |  |
| SELMODRAFT_111350 | | D8S969 | | P | | 1119 | | 373 | | | GL377607:1609453-1610574 | | 0 | | _ | |  |
| SELMODRAFT_111390 | | D8S954 | | P | | 570 | | 190 | | | GL377607:1474643-1475575 | | 1 | | _ | |  |
| SELMODRAFT_111428 | | D8S971 | | P | | 1164 | | 388 | | | GL377607:1613983-1615464 | | 1 | | SP | |  |
| SELMODRAFT_111471 | | D8S8H1 | | E | | 705 | | 235 | | | GL377607:370-1077 | | 0 | | _ | |  |
| SELMODRAFT_11159 | | D8SGQ8 | | P | | 1431 | | 477 | | | GL377619:397736-400108 | | 1 | | _ | |  |
| SELMODRAFT_111724 | | D8S9M9 | | P | | 597 | | 199 | | | GL377608:885790-886389 | | 0 | | _ | |  |
| SELMODRAFT_111767 | | D8S9K4 | | P | | 2088 | | 696 | | | GL377608:778717-780807 | | 0 | | _ | |  |
| SELMODRAFT_111830 | | D8S997 | | P | | 1458 | | 486 | | | GL377608:175224-176870 | | 1 | | _ | |  |
| SELMODRAFT_111891 | | D8S9C3 | | P | | 2157 | | 719 | | | GL377608:320484-322826 | | 0 | | Mitochondria | |  |
| SELMODRAFT_111910 | | D8S9K2 | | P | | 1869 | | 623 | | | GL377608:772924-774795 | | 0 | | _ | |  |
| SELMODRAFT_111918 | | D8S9G8 | | P | | 225 | | 75 | | | GL377608:581993-584798 | | 1 | | _ | |  |
| SELMODRAFT_111951 | | D8S9N1 | | P | | 585 | | 195 | | | GL377608:889539-890126 | | 0 | | _ | |  |
| SELMODRAFT_11198 | | D8R6J0 | | P | | 1191 | | 397 | | | GL377572:2306205-2307395 | | 0 | | _ | |  |
| SELMODRAFT_111982 | | D8S9K6 | | P | | 1041 | | 347 | | | GL377608:786286-787545 | | 1 | | SP | |  |
| SELMODRAFT_111998 | | D8S9S4 | | P | | 1329 | | 443 | | | GL377608:1171861-1173621 | | 1 | | Mitochondria | |  |
| SELMODRAFT_112001 | | D8S998 | | P | | 576 | | 192 | | | GL377608:177819-178676 | | 1 | | _ | |  |
| SELMODRAFT_112013 | | D8S9U7 | | PLS | | 1377 | | 459 | | | GL377608:1355247-1356716 | | 1 | | _ | |  |
| SELMODRAFT_112044 | | D8S9U2 | | P | | 1302 | | 434 | | | GL377608:1334806-1336110 | | 0 | | SP | |  |
| SELMODRAFT_112100 | | D8S9A9 | | P | | 1482 | | 494 | | | GL377608:210455-213375 | | 1 | | _ | |  |
| SELMODRAFT_112121 | | D8S9C1 | | P | | 567 | | 189 | | | GL377608:317631-318200 | | 0 | | _ | |  |
| SELMODRAFT_112158 | | D8S9J1 | | P | | 576 | | 192 | | | GL377608:702641-703219 | | 0 | | _ | |  |
| SELMODRAFT_112174 | | D8SAJ4 | | DYW | | 2403 | | 801 | | | GL377609:77598-80003 | | 0 | | SP | |  |
| SELMODRAFT_112220 | | D8SAN9 | | P | | 1575 | | 525 | | | GL377609:244633-252941 | | 1 | | _ | |  |
| SELMODRAFT_112410 | | D8SAQ6 | | P | | 672 | | 224 | | | GL377609:334345-339699 | | 1 | | _ | |  |
| SELMODRAFT_112424 | | D8SAU7 | | P | | 669 | | 223 | | | GL377609:546603-548951 | | 1 | | _ | |  |
| SELMODRAFT_112433 | | D8SAT0 | | DYW | | 2217 | | 739 | | | GL377609:459393-461612 | | 0 | | Mitochondria | |  |
| SELMODRAFT_112505 | | D8SAC4 | | P | | 1032 | | 344 | | | GL377609:1361072-1373836 | | 1 | | _ | |  |
| SELMODRAFT_112582 | | D8QVV6 | | P | | 540 | | 180 | | | GL377609:938950-939492 | | 0 | | _ | |  |
| SELMODRAFT_112694 | | D8SAV3 | | P | | 543 | | 181 | | | GL377609:601990-602819 | | 1 | | _ | |  |
| SELMODRAFT_113055 | | D8SB61 | | P | | 1908 | | 636 | | | GL377610:409659-411683 | | 1 | | _ | |  |
| SELMODRAFT_113147 | | D8SB24 | | P | | 1206 | | 402 | | | GL377610:183417-184925 | | 1 | | _ | |  |
| SELMODRAFT_113156 | | D8SBA8 | | DYW | | 1896 | | 632 | | | GL377610:683282-685465 | | 1 | | Mitochondria | |  |
| SELMODRAFT_113181 | | D8SB97 | | DYW | | 3324 | | 1108 | | | GL377610:635702-639028 | | 0 | | Mitochondria | |  |
| SELMODRAFT_113274 | | D8SC63 | | P | | 1401 | | 467 | | | GL377611:1171770-1173173 | | 0 | | Mitochondria | |  |
| SELMODRAFT_113516 | | D8SBW0 | | P | | 687 | | 229 | | | GL377611:431612-432298 | | 0 | | _ | |  |
| SELMODRAFT_113536 | | D8SC94 | | PLS | | 1338 | | 446 | | | GL377611:1472610-1474136 | | 1 | | _ | |  |
| SELMODRAFT_113567 | | D8SC00 | | DYW | | 2427 | | 809 | | | GL377611:707820-711200 | | 1 | | Mitochondria | |  |
| SELMODRAFT_113585 | | D8SC03 | | P | | 585 | | 195 | | | GL377611:719984-720571 | | 0 | | _ | |  |
| SELMODRAFT_113923 | | D8SCJ9 | | P | | 1134 | | 378 | | | GL377612:502355-503695 | | 1 | | _ | |  |
| SELMODRAFT_113948 | | D8SD20 | | P | | 1203 | | 401 | | | GL377612:1368331-1370259 | | 1 | | _ | |  |
| SELMODRAFT_113974 | | D8SD02 | | P | | 2202 | | 734 | | | GL377612:1277219-1280323 | | 1 | | Mitochondria | |  |
| SELMODRAFT_113996 | | D8SCH5 | | E | | 1257 | | 419 | | | GL377612:415843-417654 | | 1 | | _ | |  |
| SELMODRAFT_114007 | | D8SCJ3 | | P | | 1344 | | 448 | | | GL377612:491318-492664 | | 0 | | _ | |  |
| SELMODRAFT_114085 | | D8SCX6 | | P | | 720 | | 240 | | | GL377612:1175571-1176293 | | 0 | | _ | |  |
| SELMODRAFT_114102 | | D8SCI3 | | P | | 1506 | | 502 | | | GL377612:455065-456909 | | 1 | | _ | |  |
| SELMODRAFT_114142 | | D8SCK6 | | P | | 1389 | | 463 | | | GL377612:519670-522075 | | 2 | | _ | |  |
| SELMODRAFT_114156 | | D8SDN1 | | P | | 921 | | 307 | | | GL377613:1238692-1240209 | | 1 | | SP | |  |
| SELMODRAFT_114322 | | D8SDE1 | | E | | 2046 | | 682 | | | GL377613:747319-749661 | | 1 | | _ | |  |
| SELMODRAFT_114363 | | D8SDE5 | | DYW | | 2367 | | 789 | | | GL377613:764366-766735 | | 0 | | _ | |  |
| SELMODRAFT_114398 | | D8SDG6 | | P | | 1371 | | 457 | | | GL377613:910107-911480 | | 0 | | _ | |  |
| SELMODRAFT_114405 | | D8SDJ0 | | P | | 1533 | | 511 | | | GL377613:990078-992114 | | 1 | | _ | |  |
| SELMODRAFT_114611 | | D8SDV0 | | P | | 963 | | 321 | | | GL377614:610584-616550 | | 2 | | _ | |  |
| SELMODRAFT_114722 | | D8SDX4 | | P | | 1827 | | 609 | | | GL377614:803721-805550 | | 0 | | _ | |  |
| SELMODRAFT_114725 | | D8SDY6 | | P | | 690 | | 230 | | | GL377614:868885-869922 | | 1 | | _ | |  |
| SELMODRAFT_114819 | | D8SDX1 | | P | | 1884 | | 628 | | | GL377614:714506-716696 | | 1 | | Mitochondria | |  |
| SELMODRAFT_11486 | | D8RQ59 | | P | | 579 | | 193 | | | GL377586:1487057-1487635 | | 0 | | _ | |  |
| SELMODRAFT_114861 | | D8SDU8 | | P | | 678 | | 226 | | | GL377614:587223-587903 | | 0 | | Mitochondria | |  |
| SELMODRAFT_114873 | | D8SDU7 | | PLS | | 1815 | | 605 | | | GL377614:541894-586998 | | 2 | | Mitochondria | |  |
| SELMODRAFT_114884 | | D8SDY4 | | P | | 1923 | | 641 | | | GL377614:865045-866970 | | 0 | | _ | |  |
| SELMODRAFT_114915 | | D8SEN9 | | P | | 567 | | 189 | | | GL377615:505954-506805 | | 1 | | _ | |  |
| SELMODRAFT_114918 | | D8SEI7 | | DYW | | 1911 | | 637 | | | GL377615:1456138-1463146 | | 1 | | _ | |  |
| SELMODRAFT_115031 | | D8SEG0 | | DYW | | 1449 | | 483 | | | GL377615:1278498-1279949 | | 0 | | _ | |  |
| SELMODRAFT_115078 | | D8SEM7 | | P | | 1755 | | 585 | | | GL377615:432456-434952 | | 11 | | _ | |  |
| SELMODRAFT_115083 | | D8SE77 | | DYW | | 2019 | | 673 | | | GL377615:545727-547748 | | 0 | | _ | |  |
| SELMODRAFT_115145 | | D8SEI0 | | P | | 2148 | | 716 | | | GL377615:1370198-1389016 | | 1 | | _ | |  |
| SELMODRAFT_115229 | | D8SEA5 | | P | | 867 | | 289 | | | GL377615:735024-735962 | | 1 | | _ | |  |
| SELMODRAFT_115235 | | D8SEC8 | | P | | 1122 | | 374 | | | GL377615:1062586-1068661 | | 2 | | Mitochondria | |  |
| SELMODRAFT_115254 | | D8SEU8 | | DYW | | 2046 | | 682 | | | GL377616:298943-300991 | | 0 | | _ | |  |
| SELMODRAFT_115259 | | D8SF71 | | DYW | | 1812 | | 604 | | | GL377616:1152050-1153864 | | 0 | | SP | |  |
| SELMODRAFT_115372 | | D8SEV2 | | P | | 1350 | | 450 | | | GL377616:340470-341915 | | 1 | | _ | |  |
| SELMODRAFT_115449 | | D8SF67 | | P | | 834 | | 278 | | | GL377616:1136942-1141204 | | 1 | | _ | |  |
| SELMODRAFT_115547 | | D8SEV0 | | P | | 957 | | 319 | | | GL377616:316527-317852 | | 1 | | _ | |  |
| SELMODRAFT_115590 | | D8SEQ7 | | P | | 741 | | 247 | | | GL377616:94926-95669 | | 0 | | _ | |  |
| SELMODRAFT_115591 | | D8SEZ4 | | P | | 675 | | 225 | | | GL377616:578928-580733 | | 1 | | Mitochondria | |  |
| SELMODRAFT_116224 | | D8SG48 | | DYW | | 2760 | | 920 | | | GL377618:598849-601611 | | 0 | | _ | |  |
| SELMODRAFT_116290 | | D8SG74 | | P | | 2337 | | 779 | | | GL377618:792107-794446 | | 0 | | _ | |  |
| SELMODRAFT_116308 | | D8SGF9 | | P | | 1074 | | 358 | | | GL377618:1100871-1101947 | | 0 | | _ | |  |
| SELMODRAFT_116314 | | D8SG26 | | P | | 531 | | 177 | | | GL377618:510709-511242 | | 0 | | _ | |  |
| SELMODRAFT_116350 | | D8SG68 | | P | | 1545 | | 515 | | | GL377618:764389-766029 | | 1 | | SP | |  |
| SELMODRAFT_116430 | | D8SH05 | | P | | 609 | | 203 | | | GL377619:876463-877074 | | 0 | | Mitochondria | |  |
| SELMODRAFT_116482 | | D8SH91 | | P | | 1464 | | 488 | | | GL377619:1284521-1285987 | | 0 | | _ | |  |
| SELMODRAFT_116484 | | D8SH79 | | P | | 1467 | | 489 | | | GL377619:1238443-1240215 | | 1 | | _ | |  |
| SELMODRAFT_116494 | | D8SH33 | | P | | 780 | | 260 | | | GL377619:974846-975628 | | 0 | | _ | |  |
| SELMODRAFT_116520 | | D8SGS2 | | P | | 1038 | | 346 | | | GL377619:431733-432773 | | 0 | | _ | |  |
| SELMODRAFT_116559 | | D8SH38 | | P | | 1395 | | 465 | | | GL377619:992127-993620 | | 1 | | _ | |  |
| SELMODRAFT_116576 | | D8SH14 | | P | | 558 | | 186 | | | GL377619:904046-904606 | | 0 | | _ | |  |
| SELMODRAFT_11658 | | D8RIK8 | | P | | 1551 | | 517 | | | GL377580:2376054-2378636 | | 1 | | Mitochondria | |  |
| SELMODRAFT_116601 | | D8SGR5 | | P | | 1344 | | 448 | | | GL377619:420670-422016 | | 0 | | _ | |  |
| SELMODRAFT_116683 | | D8SH62 | | P | | 2202 | | 734 | | | GL377619:1107198-1110302 | | 1 | | Mitochondria | |  |
| SELMODRAFT_116709 | | D8SGT3 | | E | | 579 | | 193 | | | GL377619:463086-463667 | | 0 | | _ | |  |
| SELMODRAFT_116724 | | D8SH99 | | P | | 984 | | 328 | | | GL377619:1302798-1303844 | | 1 | | _ | |  |
| SELMODRAFT_116755 | | D8SGW2 | | P | | 2082 | | 694 | | | GL377619:581607-583907 | | 1 | | _ | |  |
| SELMODRAFT_116842 | | D8SGQ6 | | E | | 1701 | | 567 | | | GL377619:385425-387830 | | 1 | | Mitochondria | |  |
| SELMODRAFT_116892 | | D8SGV4 | | P | | 1215 | | 405 | | | GL377619:556841-558442 | | 2 | | _ | |  |
| SELMODRAFT_117009 | | D8SHS0 | | P | | 1227 | | 409 | | | GL377620:838904-840133 | | 0 | | _ | |  |
| SELMODRAFT_117025 | | D8SHK7 | | P | | 888 | | 296 | | | GL377620:439337-440475 | | 4 | | _ | |  |
| SELMODRAFT_117107 | | D8SHC5 | | P | | 1107 | | 369 | | | GL377620:40356-41870 | | 1 | | SP | |  |
| SELMODRAFT_117111 | | D8SHE3 | | P | | 417 | | 139 | | | GL377620:182229-182645 | | 0 | | _ | |  |
| SELMODRAFT_117142 | | D8SHD1 | | P | | 612 | | 204 | | | GL377620:105642-111583 | | 1 | | _ | |  |
| SELMODRAFT_117185 | | D8SHK6 | | P | | 567 | | 189 | | | GL377620:437064-438842 | | 1 | | _ | |  |
| SELMODRAFT_117264 | | D8SHT5 | | P | | 1350 | | 450 | | | GL377620:912721-914166 | | 1 | | _ | |  |
| SELMODRAFT_117311 | | D8SHJ3 | | DYW | | 2769 | | 923 | | | GL377620:403705-406476 | | 0 | | Mitochondria | |  |
| SELMODRAFT_117326 | | D8SHT3 | | P | | 957 | | 319 | | | GL377620:889808-891133 | | 1 | | _ | |  |
| SELMODRAFT_117346 | | D8SHT1 | | DYW | | 2046 | | 682 | | | GL377620:869316-871364 | | 0 | | _ | |  |
| SELMODRAFT_117412 | | D8SI75 | | P | | 1149 | | 383 | | | GL377621:547923-550157 | | 2 | | SP | |  |
| SELMODRAFT_117419 | | D8SIG2 | | DYW | | 2112 | | 704 | | | GL377621:1176457-1179126 | | 1 | | _ | |  |
| SELMODRAFT_117440 | | D8SIC6 | | P | | 1089 | | 363 | | | GL377621:918900-919991 | | 0 | | _ | |  |
| SELMODRAFT_117460 | | D8SI28 | | P | | 1110 | | 370 | | | GL377621:351184-352851 | | 2 | | _ | |  |
| SELMODRAFT_117546 | | D8SID4 | | P | | 1632 | | 544 | | | GL377621:949003-950637 | | 0 | | _ | |  |
| SELMODRAFT_117602 | | D8SI62 | | P | | 1602 | | 534 | | | GL377621:447519-449555 | | 6 | | _ | |  |
| SELMODRAFT_117641 | | D8SIB0 | | P | | 453 | | 151 | | | GL377621:816787-817242 | | 0 | | _ | |  |
| SELMODRAFT_117748 | | D8SI63 | | P | | 438 | | 146 | | | GL377621:450770-451207 | | 0 | | SP | |  |
| SELMODRAFT_117798 | | D8SJ85 | | DYW | | 1455 | | 485 | | | GL377622:1196496-1198877 | | 0 | | _ | |  |
| SELMODRAFT_117846 | | D8SJ20 | | P | | 936 | | 312 | | | GL377622:833617-834555 | | 0 | | _ | |  |
| SELMODRAFT_117923 | | D8SIV8 | | P | | 1152 | | 384 | | | GL377622:505583-512286 | | 1 | | _ | |  |
| SELMODRAFT_117954 | | D8SIP6 | | P | | 1485 | | 495 | | | GL377622:192255-193847 | | 1 | | Mitochondria | |  |
| SELMODRAFT_117959 | | D8SIU2 | | P | | 1263 | | 421 | | | GL377622:429717-431027 | | 1 | | _ | |  |
| SELMODRAFT_117998 | | D8SJ58 | | E | | 552 | | 184 | | | GL377622:1051313-1051867 | | 0 | | SP | |  |
| SELMODRAFT_118039 | | D8SJ42 | | DYW | | 1572 | | 524 | | | GL377622:973028-975919 | | 1 | | _ | |  |
| SELMODRAFT_118071 | | D8SJ04 | | P | | 2172 | | 724 | | | GL377622:770635-784604 | | 3 | | _ | |  |
| SELMODRAFT_118230 | | D8SJB8 | | E | | 1518 | | 506 | | | GL377623:156087-157811 | | 1 | | _ | |  |
| SELMODRAFT_118241 | | D8SJC7 | | P | | 1104 | | 368 | | | GL377623:196166-198106 | | 1 | | _ | |  |
| SELMODRAFT_118251 | | D8SJS7 | | P | | 762 | | 254 | | | GL377623:1052354-1053118 | | 0 | | _ | |  |
| SELMODRAFT_118252 | | D8SJN9 | | P | | 351 | | 117 | | | GL377623:936249-936599 | | 0 | | Mitochondria | |  |
| SELMODRAFT_118272 | | D8SJP1 | | P | | 1683 | | 561 | | | GL377623:941526-943211 | | 0 | | Mitochondria | |  |
| SELMODRAFT_11844 | | D8SAK8 | | P | | 1110 | | 370 | | | GL377609:114818-115927 | | 0 | | _ | |  |
| SELMODRAFT_118492 | | D8SJJ1 | | P | | 1434 | | 478 | | | GL377623:607722-609821 | | 2 | | _ | |  |
| SELMODRAFT_11856 | | D8SP33 | | P | | 1329 | | 443 | | | GL377630:990749-992077 | | 0 | | SP | |  |
| SELMODRAFT_118613 | | D8SJD9 | | P | | 1077 | | 359 | | | GL377623:326416-327492 | | 0 | | _ | |  |
| SELMODRAFT_118626 | | D8SJV1 | | P | | 771 | | 257 | | | GL377624:89858-90739 | | 1 | | _ | |  |
| SELMODRAFT_118751 | | D8SK43 | | P | | 1089 | | 363 | | | GL377624:614389-615945 | | 1 | | _ | |  |
| SELMODRAFT_118756 | | D8SJU5 | | PLS | | 936 | | 312 | | | GL377624:73661-74914 | | 1 | | _ | |  |
| SELMODRAFT_118817 | | D8SK79 | | P | | 627 | | 209 | | | GL377624:797637-798266 | | 0 | | Mitochondria | |  |
| SELMODRAFT_118828 | | D8SKC0 | | P | | 2889 | | 963 | | | GL377624:1082984-1085875 | | 0 | | _ | |  |
| SELMODRAFT_118946 | | D8SJZ2 | | P | | 909 | | 303 | | | GL377624:334253-336376 | | 1 | | Mitochondria | |  |
| SELMODRAFT_119022 | | D8SJZ5 | | P | | 1188 | | 396 | | | GL377624:343572-345254 | | 1 | | _ | |  |
| SELMODRAFT_119183 | | D8SKG7 | | P | | 2628 | | 876 | | | GL377625:18430-21060 | | 0 | | Mitochondria | |  |
| SELMODRAFT_119202 | | D8SL11 | | P | | 696 | | 232 | | | GL377625:902280-902975 | | 0 | | _ | |  |
| SELMODRAFT_119240 | | D8SKW7 | | P | | 738 | | 246 | | | GL377625:735116-735853 | | 0 | | _ | |  |
| SELMODRAFT_119331 | | D8SL74 | | P | | 594 | | 198 | | | GL377625:1188802-1189398 | | 0 | | _ | |  |
| SELMODRAFT_119347 | | D8SL71 | | E | | 1221 | | 407 | | | GL377625:1177064-1179352 | | 2 | | _ | |  |
| SELMODRAFT_119445 | | D8SKP7 | | DYW | | 1443 | | 481 | | | GL377625:406034-407479 | | 0 | | _ | |  |
| SELMODRAFT_119487 | | D8SL28 | | P | | 1014 | | 338 | | | GL377625:1015356-1016660 | | 2 | | _ | |  |
| SELMODRAFT_119493 | | D8SKU7 | | P | | 945 | | 315 | | | GL377625:608022-608969 | | 0 | | _ | |  |
| SELMODRAFT_119502 | | D8SKZ8 | | P | | 759 | | 253 | | | GL377625:866256-867017 | | 0 | | _ | |  |
| SELMODRAFT_119518 | | D8SL19 | | P | | 657 | | 219 | | | GL377625:930788-931444 | | 0 | | _ | |  |
| SELMODRAFT_119862 | | D8SLM0 | | P | | 1758 | | 586 | | | GL377626:815054-817111 | | 2 | | _ | |  |
| SELMODRAFT_119895 | | D8SLF6 | | DYW | | 1461 | | 487 | | | GL377626:550406-552163 | | 1 | | SP | |  |
| SELMODRAFT_119978 | | D8SLM8 | | DYW | | 1344 | | 448 | | | GL377626:850487-851833 | | 0 | | _ | |  |
| SELMODRAFT_120057 | | D8SLF4 | | E | | 1023 | | 341 | | | GL377626:542058-543083 | | 0 | | SP | |  |
| SELMODRAFT_120073 | | D8SM93 | | P | | 1980 | | 660 | | | GL377627:626541-632177 | | 1 | | Mitochondria | |  |
| SELMODRAFT_120178 | | D8SM23 | | P | | 579 | | 193 | | | GL377627:212831-214204 | | 1 | | _ | |  |
| SELMODRAFT_120182 | | D8SMI3 | | P | | 873 | | 291 | | | GL377627:1064223-1065095 | | 0 | | _ | |  |
| SELMODRAFT_120395 | | D8SLY0 | | P | | 1437 | | 479 | | | GL377627:79109-80548 | | 0 | | _ | |  |
| SELMODRAFT_12041 | | D8SZP9 | | P | | 978 | | 326 | | | GL377656:369654-371066 | | 2 | | Mitochondria | |  |
| SELMODRAFT_120439 | | D8SM24 | | P | | 1563 | | 521 | | | GL377627:215252-217150 | | 2 | | SP | |  |
| SELMODRAFT_12050 | | D8SUN3 | | P | | 855 | | 285 | | | GL377643:398349-399203 | | 0 | | _ | |  |
| SELMODRAFT_120613 | | D8SMN5 | | E | | 966 | | 322 | | | GL377628:265050-266318 | | 1 | | SP | |  |
| SELMODRAFT_120666 | | D8SMU5 | | P | | 474 | | 158 | | | GL377628:647550-648761 | | 1 | | _ | |  |
| SELMODRAFT_120669 | | D8SMP5 | | DYW | | 1107 | | 369 | | | GL377628:306380-307489 | | 0 | | _ | |  |
| SELMODRAFT_120991 | | D8SNJ7 | | P | | 933 | | 311 | | | GL377629:901628-902560 | | 0 | | _ | |  |
| SELMODRAFT_121006 | | D8SNM5 | | P | | 900 | | 300 | | | GL377629:1070534-1071436 | | 0 | | _ | |  |
| SELMODRAFT_121207 | | D8SN48 | | P | | 1692 | | 564 | | | GL377629:163532-165301 | | 1 | | Mitochondria | |  |
| SELMODRAFT_121294 | | D8SNL6 | | PLS | | 1404 | | 468 | | | GL377629:970541-972370 | | 1 | | SP | |  |
| SELMODRAFT_121321 | | D8SNT5 | | DYW | | 1137 | | 379 | | | GL377630:378440-405367 | | 3 | | _ | |  |
| SELMODRAFT_121322 | | D8SP30 | | P | | 864 | | 288 | | | GL377630:970955-983983 | | 1 | | _ | |  |
| SELMODRAFT_121341 | | D8SNU3 | | P | | 669 | | 223 | | | GL377630:454016-454867 | | 1 | | Mitochondria | |  |
| SELMODRAFT_121358 | | D8SNT6 | | P | | 912 | | 304 | | | GL377630:414756-423907 | | 3 | | _ | |  |
| SELMODRAFT_121365 | | D8SNN7 | | P | | 2289 | | 763 | | | GL377630:153961-164650 | | 4 | | SP | |  |
| SELMODRAFT_121399 | | D8SP14 | | P | | 1977 | | 659 | | | GL377630:896905-898884 | | 0 | | _ | |  |
| SELMODRAFT_121414 | | D8RIP0 | | P | | 1629 | | 543 | | | GL377630:926100-927731 | | 0 | | _ | |  |
| SELMODRAFT_121499 | | D8SP27 | | P | | 1710 | | 570 | | | GL377630:960107-961921 | | 1 | | Chloroplast | |  |
| SELMODRAFT_121537 | | D8SNR0 | | P | | 906 | | 302 | | | GL377630:234620-237165 | | 1 | | _ | |  |
| SELMODRAFT_121614 | | D8SPP3 | | P | | 1293 | | 431 | | | GL377631:953573-955291 | | 0 | | _ | |  |
| SELMODRAFT_121697 | | D8SPD5 | | DYW | | 2232 | | 744 | | | GL377631:369116-371386 | | 1 | | Mitochondria | |  |
| SELMODRAFT_121741 | | D8SPJ0 | | P | | 318 | | 106 | | | GL377631:698954-699271 | | 0 | | _ | |  |
| SELMODRAFT_121750 | | D8SPE3 | | P | | 840 | | 280 | | | GL377631:390284-391429 | | 1 | | _ | |  |
| SELMODRAFT_122009 | | D8SP95 | | P | | 741 | | 247 | | | GL377631:224557-225300 | | 0 | | Mitochondria | |  |
| SELMODRAFT_122264 | | D8SPT7 | | E | | 1212 | | 404 | | | GL377632:123306-124658 | | 1 | | _ | |  |
| SELMODRAFT_122334 | | D8SQG4 | | P | | 903 | | 301 | | | GL377633:987939-989351 | | 1 | | _ | |  |
| SELMODRAFT_122399 | | D8SQA7 | | P | | 1527 | | 509 | | | GL377633:427300-429606 | | 1 | | Mitochondria | |  |
| SELMODRAFT_122489 | | D8SQB9 | | P | | 1767 | | 589 | | | GL377633:474994-477372 | | 1 | | _ | |  |
| SELMODRAFT_122544 | | D8SQU6 | | P | | 1233 | | 411 | | | GL377634:778941-780176 | | 0 | | _ | |  |
| SELMODRAFT_122934 | | D8SQI8 | | DYW | | 2118 | | 706 | | | GL377634:94556-96988 | | 1 | | Mitochondria | |  |
| SELMODRAFT_122941 | | D8SQJ6 | | P | | 525 | | 175 | | | GL377634:142245-143072 | | 1 | | Chloroplast | |  |
| SELMODRAFT_123027 | | D8SRK2 | | P | | 933 | | 311 | | | GL377635:958948-959880 | | 0 | | _ | |  |
| SELMODRAFT_12303 | | D8RXJ4 | | P | | 1047 | | 349 | | | GL377593:1881763-1883358 | | 2 | | SP | |  |
| SELMODRAFT_12304 | | D8SZT2 | | P | | 1296 | | 432 | | | GL377656:563281-564669 | | 0 | | _ | |  |
| SELMODRAFT_123132 | | D8SR66 | | PLS | | 1722 | | 574 | | | GL377635:326135-327919 | | 1 | | Mitochondria | |  |
| SELMODRAFT_123298 | | D8SRY0 | | DYW | | 2067 | | 689 | | | GL377636:744706-746775 | | 0 | | _ | |  |
| SELMODRAFT_123361 | | D8SRM0 | | P | | 570 | | 190 | | | GL377636:10955-11698 | | 1 | | SP | |  |
| SELMODRAFT_123379 | | D8SRZ3 | | P | | 711 | | 237 | | | GL377636:786560-793134 | | 1 | | _ | |  |
| SELMODRAFT_123384 | | D8SRP2 | | P | | 474 | | 158 | | | GL377636:176639-177115 | | 0 | | Mitochondria | |  |
| SELMODRAFT_123450 | | D8SRX8 | | P | | 804 | | 268 | | | GL377636:741377-742183 | | 0 | | _ | |  |
| SELMODRAFT_123482 | | D8SRS8 | | P | | 777 | | 259 | | | GL377636:516285-518348 | | 1 | | Mitochondria | |  |
| SELMODRAFT_123584 | | D8SS79 | | DYW | | 1134 | | 378 | | | GL377637:540619-584451 | | 0 | | _ | |  |
| SELMODRAFT_123646 | | D8SSA1 | | P | | 657 | | 219 | | | GL377637:755092-755748 | | 0 | | _ | |  |
| SELMODRAFT_123655 | | D8SS77 | | P | | 738 | | 246 | | | GL377637:533331-535145 | | 1 | | _ | |  |
| SELMODRAFT_123660 | | D8SS78 | | P | | 2175 | | 725 | | | GL377637:535585-539785 | | 1 | | Mitochondria | |  |
| SELMODRAFT_123701 | | D8SS40 | | P | | 480 | | 160 | | | GL377637:257097-257858 | | 1 | | _ | |  |
| SELMODRAFT_123731 | | D8SS27 | | P | | 1317 | | 439 | | | GL377637:203143-204462 | | 0 | | SP | |  |
| SELMODRAFT_123789 | | D8SSX3 | | P | | 1926 | | 642 | | | GL377638:901192-904038 | | 3 | | _ | |  |
| SELMODRAFT_123901 | | D8SSN7 | | P | | 954 | | 318 | | | GL377638:552526-553482 | | 0 | | Mitochondria | |  |
| SELMODRAFT_123904 | | D8SSH5 | | P | | 1383 | | 461 | | | GL377638:210491-212989 | | 2 | | _ | |  |
| SELMODRAFT_123910 | | D8SSG9 | | PLS | | 1548 | | 516 | | | GL377638:186187-188439 | | 2 | | _ | |  |
| SELMODRAFT_124032 | | D8SSL5 | | P | | 633 | | 211 | | | GL377638:467814-468743 | | 1 | | _ | |  |
| SELMODRAFT_124046 | | D8SSV6 | | P | | 1035 | | 345 | | | GL377638:849534-850568 | | 0 | | Mitochondria | |  |
| SELMODRAFT_124083 | | D8SSJ4 | | P | | 1335 | | 445 | | | GL377638:404110-405447 | | 0 | | Mitochondria | |  |
| SELMODRAFT_124101 | | D8STD5 | | P | | 1113 | | 371 | | | GL377639:906862-908310 | | 2 | | _ | |  |
| SELMODRAFT_124189 | | D8STB0 | | P | | 1335 | | 445 | | | GL377639:722992-724329 | | 0 | | _ | |  |
| SELMODRAFT_124337 | | D8STC2 | | DYW | | 1461 | | 487 | | | GL377639:818251-819714 | | 0 | | _ | |  |
| SELMODRAFT_124407 | | D8STD3 | | P | | 1875 | | 625 | | | GL377639:880745-886268 | | 2 | | _ | |  |
| SELMODRAFT_124408 | | D8STF1 | | P | | 741 | | 247 | | | GL377639:970768-971557 | | 1 | | SP | |  |
| SELMODRAFT_124564 | | D8STL5 | | P | | 2073 | | 691 | | | GL377640:500796-503436 | | 3 | | SP | |  |
| SELMODRAFT_124576 | | D8STL0 | | P | | 1986 | | 662 | | | GL377640:483301-485289 | | 0 | | Chloroplast | |  |
| SELMODRAFT_124816 | | D8SU13 | | P | | 2535 | | 845 | | | GL377641:473617-476154 | | 0 | | Mitochondria | |  |
| SELMODRAFT_124927 | | D8SUB0 | | P | | 1476 | | 492 | | | GL377642:562436-564706 | | 4 | | Mitochondria | |  |
| SELMODRAFT_125114 | | D8SUC7 | | P | | 1131 | | 377 | | | GL377642:689290-690627 | | 1 | | Chloroplast | |  |
| SELMODRAFT_125134 | | D8SU54 | | P | | 1374 | | 458 | | | GL377642:180710-182086 | | 0 | | _ | |  |
| SELMODRAFT_125156 | | D8SU86 | | P | | 1005 | | 335 | | | GL377642:417082-418965 | | 1 | | _ | |  |
| SELMODRAFT_125160 | | D8SU94 | | P | | 657 | | 219 | | | GL377642:455605-462527 | | 1 | | _ | |  |
| SELMODRAFT_125193 | | D8SUK6 | | P | | 774 | | 258 | | | GL377643:284835-286340 | | 1 | | _ | |  |
| SELMODRAFT_125221 | | D8SUM6 | | P | | 843 | | 281 | | | GL377643:349664-350506 | | 0 | | _ | |  |
| SELMODRAFT_125319 | | D8SUL3 | | P | | 1767 | | 589 | | | GL377643:306769-309147 | | 1 | | _ | |  |
| SELMODRAFT_125425 | | D8SUX3 | | P | | 1614 | | 538 | | | GL377644:225708-244546 | | 2 | | _ | |  |
| SELMODRAFT_125444 | | D8SUX9 | | E | | 1197 | | 399 | | | GL377644:355770-357155 | | 2 | | _ | |  |
| SELMODRAFT_125581 | | D8SV84 | | P | | 1047 | | 349 | | | GL377644:844173-845285 | | 1 | | Mitochondria | |  |
| SELMODRAFT_125582 | | D8SUT9 | | P | | 1059 | | 353 | | | GL377644:53774-56284 | | 1 | | _ | |  |
| SELMODRAFT_125731 | | D8SVA1 | | E | | 660 | | 220 | | | GL377645:229847-230797 | | 1 | | _ | |  |
| SELMODRAFT_125795 | | D8SVF7 | | P | | 468 | | 156 | | | GL377645:622805-623275 | | 0 | | Mitochondria | |  |
| SELMODRAFT_125812 | | D8SVH2 | | P | | 519 | | 173 | | | GL377645:759682-760203 | | 0 | | _ | |  |
| SELMODRAFT_125836 | | D8SVH3 | | P | | 1533 | | 511 | | | GL377645:763016-764968 | | 2 | | _ | |  |
| SELMODRAFT_125838 | | D8SVA2 | | P | | 309 | | 103 | | | GL377645:232680-232991 | | 0 | | Mitochondria | |  |
| SELMODRAFT_125978 | | D8SVP4 | | P | | 888 | | 296 | | | GL377646:399002-400627 | | 1 | | _ | |  |
| SELMODRAFT_125980 | | D8SVJ8 | | P | | 669 | | 223 | | | GL377646:2342-4042 | | 1 | | SP | |  |
| SELMODRAFT_125997 | | D8SVW7 | | P | | 720 | | 240 | | | GL377646:826239-827243 | | 1 | | _ | |  |
| SELMODRAFT_126073 | | D8SVJ9 | | P | | 783 | | 261 | | | GL377646:4229-5011 | | 0 | | SP | |  |
| SELMODRAFT_126075 | | D8SVS4 | | P | | 825 | | 275 | | | GL377646:575088-575915 | | 0 | | _ | |  |
| SELMODRAFT_126135 | | D8SVP5 | | P | | 915 | | 305 | | | GL377646:401081-402454 | | 1 | | _ | |  |
| SELMODRAFT_126148 | | D8SVS2 | | P | | 567 | | 189 | | | GL377646:570720-571289 | | 0 | | _ | |  |
| SELMODRAFT_126152 | | D8SVK9 | | P | | 945 | | 315 | | | GL377646:195552-196775 | | 1 | | _ | |  |
| SELMODRAFT_126194 | | D8SW10 | | P | | 1623 | | 541 | | | GL377647:352823-355072 | | 2 | | SP | |  |
| SELMODRAFT_126210 | | D8SW75 | | P | | 567 | | 189 | | | GL377647:784318-784887 | | 0 | | _ | |  |
| SELMODRAFT_126354 | | D8SW50 | | P | | 927 | | 309 | | | GL377647:651819-653021 | | 1 | | _ | |  |
| SELMODRAFT_126411 | | D8SWD3 | | P | | 993 | | 331 | | | GL377648:341375-342967 | | 1 | | Mitochondria | |  |
| SELMODRAFT_126599 | | D8SWF7 | | P | | 1620 | | 540 | | | GL377648:464274-466100 | | 1 | | Mitochondria | |  |
| SELMODRAFT_126675 | | D8SWT4 | | P | | 288 | | 96 | | | GL377649:308302-308589 | | 0 | | _ | |  |
| SELMODRAFT_126729 | | D8SWP9 | | P | | 501 | | 167 | | | GL377649:119549-120052 | | 0 | | _ | |  |
| SELMODRAFT_126772 | | D8SWV5 | | E | | 1140 | | 380 | | | GL377649:399623-400945 | | 1 | | _ | |  |
| SELMODRAFT_126782 | | D8SWN5 | | P | | 1797 | | 599 | | | GL377649:30921-33083 | | 1 | | _ | |  |
| SELMODRAFT_126797 | | D8SWX2 | | P | | 1554 | | 518 | | | GL377649:554294-556048 | | 1 | | _ | |  |
| SELMODRAFT_126801 | | D8SWW7 | | P | | 699 | | 233 | | | GL377649:509212-509913 | | 0 | | _ | |  |
| SELMODRAFT_126864 | | D8SWN9 | | P | | 1767 | | 589 | | | GL377649:37590-39653 | | 1 | | _ | |  |
| SELMODRAFT_126882 | | D8SWQ8 | | DYW | | 1539 | | 513 | | | GL377649:141069-142610 | | 0 | | _ | |  |
| SELMODRAFT_126928 | | D8SX22 | | E | | 1311 | | 437 | | | GL377650:156795-158108 | | 0 | | _ | |  |
| SELMODRAFT_126951 | | D8SX73 | | P | | 900 | | 300 | | | GL377650:523764-524951 | | 1 | | _ | |  |
| SELMODRAFT_127009 | | D8SX16 | | P | | 738 | | 246 | | | GL377650:137270-138007 | | 0 | | _ | |  |
| SELMODRAFT_127010 | | D8SX39 | | P | | 1209 | | 403 | | | GL377650:393305-394966 | | 2 | | _ | |  |
| SELMODRAFT_127119 | | D8SXI3 | | P | | 1188 | | 396 | | | GL377651:392594-394525 | | 1 | | _ | |  |
| SELMODRAFT_127120 | | D8SXT2 | | E | | 1299 | | 433 | | | GL377651:773249-775009 | | 1 | | _ | |  |
| SELMODRAFT_127153 | | D8SXF5 | | P | | 1035 | | 345 | | | GL377651:282270-284225 | | 1 | | Chloroplast | |  |
| SELMODRAFT_127233 | | D8SXC7 | | P | | 729 | | 243 | | | GL377651:124093-124821 | | 0 | | _ | |  |
| SELMODRAFT_127332 | | D8SXN7 | | E | | 579 | | 193 | | | GL377651:588738-589319 | | 0 | | _ | |  |
| SELMODRAFT_127380 | | D8SXD6 | | P | | 780 | | 260 | | | GL377651:142488-143576 | | 0 | | _ | |  |
| SELMODRAFT_127389 | | D8SXI0 | | P | | 867 | | 289 | | | GL377651:383733-385019 | | 1 | | Chloroplast | |  |
| SELMODRAFT_127538 | | D8SY04 | | P | | 459 | | 153 | | | GL377652:248780-249241 | | 0 | | _ | |  |
| SELMODRAFT_127559 | | D8SY05 | | P | | 687 | | 229 | | | GL377652:249569-250255 | | 0 | | _ | |  |
| SELMODRAFT_127579 | | D8SXW5 | | DYW | | 2226 | | 742 | | | GL377652:117564-119792 | | 0 | | _ | |  |
| SELMODRAFT_127658 | | D8SY21 | | DYW | | 1437 | | 479 | | | GL377652:356601-358040 | | 0 | | SP | |  |
| SELMODRAFT_127664 | | D8SY58 | | P | | 1131 | | 377 | | | GL377652:586730-588265 | | 1 | | _ | |  |
| SELMODRAFT_127725 | | D8SYA4 | | P | | 990 | | 330 | | | GL377652:721960-722952 | | 0 | | _ | |  |
| SELMODRAFT_127736 | | D8SY89 | | E | | 1965 | | 655 | | | GL377652:683106-685652 | | 1 | | _ | |  |
| SELMODRAFT_127772 | | D8SXU6 | | P | | 672 | | 224 | | | GL377652:23716-24390 | | 0 | | _ | |  |
| SELMODRAFT_127854 | | D8SYF4 | | P | | 564 | | 188 | | | GL377653:113907-115157 | | 1 | | _ | |  |
| SELMODRAFT_127877 | | D8SYN8 | | P | | 1770 | | 590 | | | GL377653:552190-556854 | | 3 | | Mitochondria | |  |
| SELMODRAFT_127914 | | D8SYR0 | | P | | 471 | | 157 | | | GL377653:673553-674026 | | 0 | | _ | |  |
| SELMODRAFT_128001 | | D8SYM8 | | P | | 1164 | | 388 | | | GL377653:516207-517688 | | 1 | | SP | |  |
| SELMODRAFT_128024 | | D8SYM6 | | P | | 900 | | 300 | | | GL377653:511870-512772 | | 0 | | _ | |  |
| SELMODRAFT_128086 | | D8SYK9 | | P | | 570 | | 190 | | | GL377653:390025-390957 | | 1 | | _ | |  |
| SELMODRAFT_128138 | | D8SZ40 | | P | | 1836 | | 612 | | | GL377654:616870-619506 | | 2 | | _ | |  |
| SELMODRAFT_12814 | | D8S9J5 | | P | | 660 | | 220 | | | GL377608:723956-726519 | | 3 | | _ | |  |
| SELMODRAFT_128148 | | D8SZ51 | | P | | 513 | | 171 | | | GL377654:672539-673054 | | 0 | | Mitochondria | |  |
| SELMODRAFT_128246 | | D8SZ46 | | P | | 426 | | 142 | | | GL377654:653064-653492 | | 0 | | _ | |  |
| SELMODRAFT_128481 | | D8SZF8 | | P | | 1182 | | 394 | | | GL377655:331585-332769 | | 1 | | _ | |  |
| SELMODRAFT_128498 | | D8SZI2 | | PLS | | 1719 | | 573 | | | GL377655:492043-493950 | | 1 | | _ | |  |
| SELMODRAFT_128569 | | D8SZD4 | | P | | 633 | | 211 | | | GL377655:268315-269244 | | 1 | | _ | |  |
| SELMODRAFT_128578 | | D8SZH6 | | P | | 684 | | 228 | | | GL377655:468189-468872 | | 0 | | _ | |  |
| SELMODRAFT_128601 | | D8SZB5 | | P | | 954 | | 318 | | | GL377655:219303-220259 | | 0 | | Mitochondria | |  |
| SELMODRAFT_128660 | | D8SZR2 | | P | | 1317 | | 439 | | | GL377656:425416-427200 | | 1 | | _ | |  |
| SELMODRAFT_128769 | | D8SZQ6 | | P | | 1641 | | 547 | | | GL377656:400186-401916 | | 1 | | _ | |  |
| SELMODRAFT_128798 | | D8SZL1 | | P | | 1743 | | 581 | | | GL377656:4490-6760 | | 2 | | _ | |  |
| SELMODRAFT_128847 | | D8SZL0 | | DYW | | 2388 | | 796 | | | GL377656:2057-4447 | | 0 | | Mitochondria | |  |
| SELMODRAFT_128850 | | D8SZN2 | | P | | 2943 | | 981 | | | GL377656:174344-177753 | | 2 | | Mitochondria | |  |
| SELMODRAFT_129138 | | D8T0D5 | | E | | 2418 | | 806 | | | GL377658:292644-295355 | | 1 | | Mitochondria | |  |
| SELMODRAFT_129183 | | D8T0J3 | | P | | 642 | | 214 | | | GL377658:583962-584606 | | 0 | | Mitochondria | |  |
| SELMODRAFT_129209 | | D8T0D3 | | P | | 2916 | | 972 | | | GL377658:286478-289396 | | 0 | | Mitochondria | |  |
| SELMODRAFT_129287 | | D8T0Q3 | | P | | 468 | | 156 | | | GL377659:557502-557972 | | 0 | | Mitochondria | |  |
| SELMODRAFT_129330 | | D8T0N8 | | P | | 522 | | 174 | | | GL377659:352795-353319 | | 0 | | _ | |  |
| SELMODRAFT_129336 | | D8T0S6 | | P | | 2022 | | 674 | | | GL377659:627137-629236 | | 1 | | _ | |  |
| SELMODRAFT_129364 | | D8T0T5 | | P | | 690 | | 230 | | | GL377659:664822-665511 | | 0 | | _ | |  |
| SELMODRAFT_129427 | | D8T0N7 | | P | | 1842 | | 614 | | | GL377659:348038-349990 | | 1 | | _ | |  |
| SELMODRAFT_129545 | | D8T113 | | P | | 870 | | 290 | | | GL377660:695316-696188 | | 0 | | _ | |  |
| SELMODRAFT_129552 | | D8T111 | | P | | 744 | | 248 | | | GL377660:691382-692524 | | 1 | | Mitochondria | |  |
| SELMODRAFT_129614 | | D8T162 | | P | | 549 | | 183 | | | GL377661:335413-336327 | | 1 | | SP | |  |
| SELMODRAFT_129617 | | D8T119 | | P | | 1338 | | 446 | | | GL377661:47252-48778 | | 1 | | _ | |  |
| SELMODRAFT_129646 | | D8T157 | | P | | 372 | | 124 | | | GL377661:313552-313926 | | 0 | | SP | |  |
| SELMODRAFT_129708 | | D8T158 | | P | | 705 | | 235 | | | GL377661:314510-315214 | | 0 | | _ | |  |
| SELMODRAFT_129764 | | D8T1C3 | | P | | 693 | | 231 | | | GL377662:148571-153013 | | 1 | | SP | |  |
| SELMODRAFT_129900 | | D8T1V1 | | P | | 444 | | 148 | | | GL377663:520487-520933 | | 0 | | _ | |  |
| SELMODRAFT_129917 | | D8T1R7 | | P | | 2232 | | 744 | | | GL377663:360294-362840 | | 1 | | Mitochondria | |  |
| SELMODRAFT_129973 | | D8T1W7 | | P | | 1455 | | 485 | | | GL377663:593025-595682 | | 1 | | _ | |  |
| SELMODRAFT_129982 | | D8T1P0 | | P | | 1146 | | 382 | | | GL377663:275587-276735 | | 0 | | SP | |  |
| SELMODRAFT_130008 | | D8T1L1 | | P | | 1308 | | 436 | | | GL377663:159010-160320 | | 0 | | _ | |  |
| SELMODRAFT_130050 | | D8T1W2 | | DYW | | 2208 | | 736 | | | GL377663:553309-556116 | | 1 | | SP | |  |
| SELMODRAFT_130235 | | D8T229 | | DYW | | 2067 | | 689 | | | GL377664:517290-519359 | | 0 | | _ | |  |
| SELMODRAFT_130253 | | D8T2A0 | | P | | 864 | | 288 | | | GL377665:249975-251141 | | 1 | | SP | |  |
| SELMODRAFT_130420 | | D8T2B2 | | P | | 828 | | 276 | | | GL377665:324560-325390 | | 0 | | _ | |  |
| SELMODRAFT_130421 | | D8T296 | | P | | 699 | | 233 | | | GL377665:232391-234392 | | 1 | | _ | |  |
| SELMODRAFT_130453 | | D8T2P8 | | DYW | | 2823 | | 941 | | | GL377666:499437-502262 | | 0 | | SP | |  |
| SELMODRAFT_130516 | | D8T2P0 | | P | | 1461 | | 487 | | | GL377666:446180-447946 | | 2 | | _ | |  |
| SELMODRAFT_130525 | | D8T2M7 | | P | | 2268 | | 756 | | | GL377666:310983-342019 | | 4 | | _ | |  |
| SELMODRAFT_130540 | | D8T2P4 | | E | | 783 | | 261 | | | GL377666:464812-466197 | | 1 | | Mitochondria | |  |
| SELMODRAFT_130617 | | D8T2V1 | | E | | 1185 | | 395 | | | GL377667:138675-140576 | | 1 | | _ | |  |
| SELMODRAFT_130658 | | D8T300 | | DYW | | 2454 | | 818 | | | GL377667:352475-355123 | | 1 | | _ | |  |
| SELMODRAFT_130917 | | D8T351 | | P | | 1341 | | 447 | | | GL377667:558974-560761 | | 2 | | SP | |  |
| SELMODRAFT_130980 | | D8T388 | | P | | 1374 | | 458 | | | GL377668:466779-468155 | | 0 | | _ | |  |
| SELMODRAFT_131092 | | D8T3P2 | | P | | 1896 | | 632 | | | GL377670:259085-261496 | | 1 | | _ | |  |
| SELMODRAFT_131102 | | D8T3L0 | | P | | 2280 | | 760 | | | GL377670:143231-145765 | | 3 | | Chloroplast | |  |
| SELMODRAFT_131128 | | D8T3M0 | | P | | 663 | | 221 | | | GL377670:177635-179932 | | 1 | | Mitochondria | |  |
| SELMODRAFT_131170 | | D8T3L3 | | DYW | | 2547 | | 849 | | | GL377670:148595-151144 | | 0 | | Mitochondria | |  |
| SELMODRAFT_131203 | | D8T3P0 | | P | | 693 | | 231 | | | GL377670:224721-225692 | | 1 | | SP | |  |
| SELMODRAFT_131439 | | D8T3Y3 | | P | | 3417 | | 1139 | | | GL377672:114817-118659 | | 7 | | Chloroplast | |  |
| SELMODRAFT_131477 | | D8T454 | | P | | 846 | | 282 | | | GL377672:535327-536175 | | 0 | | _ | |  |
| SELMODRAFT_131485 | | D8T456 | | P | | 780 | | 260 | | | GL377672:546148-547287 | | 1 | | _ | |  |
| SELMODRAFT_131509 | | D8T455 | | P | | 1668 | | 556 | | | GL377672:536382-538415 | | 2 | | _ | |  |
| SELMODRAFT_131534 | | D8T428 | | P | | 1377 | | 459 | | | GL377672:385697-387709 | | 1 | | _ | |  |
| SELMODRAFT_131583 | | D8T472 | | P | | 699 | | 233 | | | GL377673:168391-169092 | | 0 | | _ | |  |
| SELMODRAFT_131638 | | D8T470 | | P | | 561 | | 187 | | | GL377673:133785-135086 | | 1 | | _ | |  |
| SELMODRAFT_131641 | | D8T489 | | P | | 1035 | | 345 | | | GL377673:237551-238588 | | 0 | | Mitochondria | |  |
| SELMODRAFT_131642 | | D8T4D6 | | P | | 2490 | | 830 | | | GL377673:489508-492000 | | 0 | | _ | |  |
| SELMODRAFT_131643 | | D8T4B7 | | P | | 1476 | | 492 | | | GL377673:415978-417456 | | 0 | | _ | |  |
| SELMODRAFT_131657 | | D8T4D5 | | P | | 1065 | | 355 | | | GL377673:488044-489348 | | 3 | | _ | |  |
| SELMODRAFT_131784 | | D8T4F1 | | P | | 573 | | 191 | | | GL377674:10588-11163 | | 0 | | _ | |  |
| SELMODRAFT_131810 | | D8T4M9 | | P | | 1692 | | 564 | | | GL377674:369954-371942 | | 1 | | _ | |  |
| SELMODRAFT_131822 | | D8T4L6 | | P | | 1065 | | 355 | | | GL377674:310596-311663 | | 0 | | _ | |  |
| SELMODRAFT_131869 | | D8T4L9 | | DYW | | 1467 | | 489 | | | GL377674:318685-320844 | | 1 | | _ | |  |
| SELMODRAFT_131937 | | D8T4H5 | | P | | 843 | | 281 | | | GL377674:126021-126866 | | 0 | | _ | |  |
| SELMODRAFT_132095 | | D8T529 | | P | | 681 | | 227 | | | GL377675:527239-528189 | | 1 | | _ | |  |
| SELMODRAFT_132101 | | D8T4V4 | | P | | 696 | | 232 | | | GL377675:235659-236813 | | 1 | | _ | |  |
| SELMODRAFT_132132 | | D8T4W8 | | P | | 597 | | 199 | | | GL377675:286251-286850 | | 0 | | Chloroplast | |  |
| SELMODRAFT_132169 | | D8T538 | | DYW | | 1338 | | 446 | | | GL377675:561305-562645 | | 0 | | _ | |  |
| SELMODRAFT_132252 | | D8T559 | | P | | 1872 | | 624 | | | GL377676:137294-139168 | | 0 | | _ | |  |
| SELMODRAFT_132289 | | D8T578 | | P | | 1983 | | 661 | | | GL377676:224090-227422 | | 2 | | Mitochondria | |  |
| SELMODRAFT_132312 | | D8T5J2 | | P | | 648 | | 216 | | | GL377677:438685-440247 | | 1 | | _ | |  |
| SELMODRAFT_132317 | | D8T5J6 | | P | | 852 | | 284 | | | GL377677:460904-462370 | | 1 | | Mitochondria | |  |
| SELMODRAFT_132337 | | D8T5D2 | | P | | 1872 | | 624 | | | GL377677:99751-103552 | | 2 | | _ | |  |
| SELMODRAFT_132374 | | D8T5L2 | | P | | 744 | | 248 | | | GL377677:538168-538911 | | 0 | | _ | |  |
| SELMODRAFT_132472 | | D8T5H5 | | P | | 1452 | | 484 | | | GL377677:310307-312706 | | 1 | | _ | |  |
| SELMODRAFT_132504 | | D8T5H3 | | PLS | | 1662 | | 554 | | | GL377677:301831-303717 | | 1 | | _ | |  |
| SELMODRAFT_132593 | | D8T5N3 | | P | | 1071 | | 357 | | | GL377678:108869-109942 | | 0 | | _ | |  |
| SELMODRAFT_132764 | | D8T5L9 | | P | | 612 | | 204 | | | GL377678:12206-18238 | | 1 | | _ | |  |
| SELMODRAFT_132819 | | D8T621 | | P | | 462 | | 154 | | | GL377679:186945-187409 | | 0 | | Mitochondria | |  |
| SELMODRAFT_132894 | | D8T661 | | P | | 522 | | 174 | | | GL377679:317084-317608 | | 0 | | Mitochondria | |  |
| SELMODRAFT_132923 | | D8T660 | | P | | 714 | | 238 | | | GL377679:316169-316882 | | 0 | | SP | |  |
| SELMODRAFT_132950 | | D8T6D2 | | DYW | | 1746 | | 582 | | | GL377680:426104-428155 | | 1 | | Mitochondria | |  |
| SELMODRAFT_133020 | | D8T6C9 | | DYW | | 1896 | | 632 | | | GL377680:387363-389501 | | 1 | | _ | |  |
| SELMODRAFT_133094 | | D8T6F8 | | P | | 855 | | 285 | | | GL377681:146690-147547 | | 0 | | Mitochondria | |  |
| SELMODRAFT_133216 | | D8T6L9 | | P | | 855 | | 285 | | | GL377682:162629-163486 | | 0 | | Mitochondria | |  |
| SELMODRAFT_133320 | | D8T6S2 | | P | | 540 | | 180 | | | GL377683:45798-46340 | | 0 | | Mitochondria | |  |
| SELMODRAFT_133407 | | D8T741 | | P | | 597 | | 199 | | | GL377684:199813-200412 | | 0 | | _ | |  |
| SELMODRAFT_133508 | | D8T726 | | E | | 1956 | | 652 | | | GL377684:159120-161666 | | 1 | | _ | |  |
| SELMODRAFT_133535 | | D8T6Z0 | | P | | 1128 | | 376 | | | GL377684:51828-53903 | | 1 | | _ | |  |
| SELMODRAFT_133644 | | D8T7E0 | | P | | 2427 | | 809 | | | GL377685:224228-227446 | | 3 | | _ | |  |
| SELMODRAFT_133742 | | D8T7K8 | | P | | 558 | | 186 | | | GL377686:159526-160362 | | 1 | | _ | |  |
| SELMODRAFT_133790 | | D8T7L2 | | P | | 534 | | 178 | | | GL377686:166135-166671 | | 0 | | Mitochondria | |  |
| SELMODRAFT_133813 | | D8T7L6 | | P | | 1068 | | 356 | | | GL377686:182693-183763 | | 0 | | _ | |  |
| SELMODRAFT_133817 | | D8T7Q4 | | P | | 1791 | | 597 | | | GL377686:315945-317837 | | 1 | | _ | |  |
| SELMODRAFT_133828 | | D8T7J1 | | P | | 1242 | | 414 | | | GL377686:59658-61529 | | 1 | | _ | |  |
| SELMODRAFT_133872 | | D8T7M2 | | P | | 669 | | 223 | | | GL377686:196213-198282 | | 1 | | Mitochondria | |  |
| SELMODRAFT_133908 | | D8T7U2 | | P | | 1767 | | 589 | | | GL377687:132850-134895 | | 1 | | _ | |  |
| SELMODRAFT_134101 | | D8T832 | | P | | 621 | | 207 | | | GL377688:172468-173091 | | 1 | | _ | |  |
| SELMODRAFT_134108 | | D8T851 | | P | | 930 | | 310 | | | GL377688:273918-274847 | | 0 | | _ | |  |
| SELMODRAFT_134168 | | D8T830 | | P | | 450 | | 150 | | | GL377688:145314-145766 | | 0 | | _ | |  |
| SELMODRAFT_134189 | | D8T871 | | P | | 1695 | | 565 | | | GL377688:341752-343626 | | 1 | | Mitochondria | |  |
| SELMODRAFT_134202 | | D8T881 | | P | | 831 | | 277 | | | GL377688:369277-370110 | | 0 | | _ | |  |
| SELMODRAFT_134253 | | D8T8E4 | | P | | 909 | | 303 | | | GL377689:325326-327073 | | 2 | | _ | |  |
| SELMODRAFT_134282 | | D8T8B8 | | P | | 1275 | | 425 | | | GL377689:188988-190571 | | 1 | | Mitochondria | |  |
| SELMODRAFT_134380 | | D8T8K2 | | P | | 669 | | 223 | | | GL377690:292250-293764 | | 1 | | _ | |  |
| SELMODRAFT_134394 | | D8T8L4 | | P | | 585 | | 195 | | | GL377690:358568-359155 | | 0 | | _ | |  |
| SELMODRAFT_134407 | | D8T8L6 | | P | | 1149 | | 383 | | | GL377690:361758-362909 | | 0 | | _ | |  |
| SELMODRAFT_134446 | | D8T8G8 | | P | | 2070 | | 690 | | | GL377690:113230-115530 | | 1 | | _ | |  |
| SELMODRAFT_134463 | | D8T8R6 | | DYW | | 1860 | | 620 | | | GL377691:214635-217316 | | 1 | | _ | |  |
| SELMODRAFT_134528 | | D8T8Q5 | | P | | 912 | | 304 | | | GL377691:173160-174071 | | 0 | | _ | |  |
| SELMODRAFT_134538 | | D8T8R8 | | E | | 684 | | 228 | | | GL377691:232342-233028 | | 0 | | _ | |  |
| SELMODRAFT_134546 | | D8T8Q2 | | P | | 219 | | 73 | | | GL377691:100784-101005 | | 0 | | Mitochondria | |  |
| SELMODRAFT_134551 | | D8T8S3 | | P | | 2841 | | 947 | | | GL377691:250642-288784 | | 3 | | _ | |  |
| SELMODRAFT_134559 | | D8T8P3 | | P | | 288 | | 96 | | | GL377691:18543-18830 | | 0 | | _ | |  |
| SELMODRAFT_134581 | | D8T8Y5 | | DYW | | 2313 | | 771 | | | GL377692:230142-242434 | | 1 | | Mitochondria | |  |
| SELMODRAFT_13472 | | D8RGE2 | | E | | 996 | | 332 | | | GL377579:676293-677813 | | 1 | | _ | |  |
| SELMODRAFT_134779 | | D8T946 | | P | | 1818 | | 606 | | | GL377694:67548-70166 | | 1 | | Mitochondria | |  |
| SELMODRAFT_134788 | | D8T990 | | P | | 1392 | | 464 | | | GL377694:290491-292236 | | 0 | | _ | |  |
| SELMODRAFT_134893 | | D8T9F2 | | P | | 501 | | 167 | | | GL377695:198951-199454 | | 1 | | _ | |  |
| SELMODRAFT_134993 | | D8T9J7 | | P | | 1761 | | 587 | | | GL377696:238505-240568 | | 1 | | _ | |  |
| SELMODRAFT_135000 | | D8T9J3 | | P | | 1635 | | 545 | | | GL377696:231820-233982 | | 1 | | _ | |  |
| SELMODRAFT_135030 | | D8T9L3 | | DYW | | 1644 | | 548 | | | GL377696:328190-330118 | | 1 | | _ | |  |
| SELMODRAFT_135087 | | D8T9R4 | | P | | 1287 | | 429 | | | GL377698:240572-241861 | | 0 | | SP | |  |
| SELMODRAFT_13509 | | D8S9J4 | | P | | 1032 | | 344 | | | GL377608:721364-722710 | | 1 | | _ | |  |
| SELMODRAFT_135096 | | D8T9R1 | | P | | 834 | | 278 | | | GL377698:229913-230749 | | 0 | | _ | |  |
| SELMODRAFT_135281 | | D8T9U1 | | P | | 2094 | | 698 | | | GL377699:42640-44736 | | 0 | | Mitochondria | |  |
| SELMODRAFT_135367 | | D8TAA8 | | P | | 2277 | | 759 | | | GL377701:273350-275870 | | 4 | | Chloroplast | |  |
| SELMODRAFT_135370 | | D8TAA5 | | DYW | | 2547 | | 849 | | | GL377701:267928-270477 | | 0 | | Mitochondria | |  |
| SELMODRAFT_135409 | | D8TA73 | | P | | 1896 | | 632 | | | GL377701:186232-188643 | | 2 | | _ | |  |
| SELMODRAFT_135469 | | D8TA98 | | P | | 663 | | 221 | | | GL377701:239135-241432 | | 1 | | Mitochondria | |  |
| SELMODRAFT_135637 | | D8TAL3 | | P | | 2235 | | 745 | | | GL377703:258497-261214 | | 2 | | _ | |  |
| SELMODRAFT_135687 | | D8R9D9 | | P | | 819 | | 273 | | | GL377703:130893-131879 | | 1 | | Mitochondria | |  |
| SELMODRAFT_135749 | | D8TAR3 | | P | | 615 | | 205 | | | GL377704:204766-206031 | | 1 | | _ | |  |
| SELMODRAFT_135756 | | D8TAQ2 | | P | | 651 | | 217 | | | GL377704:162169-162822 | | 0 | | _ | |  |
| SELMODRAFT_135764 | | D8TAN6 | | P | | 507 | | 169 | | | GL377704:93799-94308 | | 0 | | Mitochondria | |  |
| SELMODRAFT_135788 | | D8TAT0 | | P | | 2823 | | 941 | | | GL377704:304975-313018 | | 3 | | _ | |  |
| SELMODRAFT_135793 | | D8TAU1 | | P | | 237 | | 79 | | | GL377705:58589-58825 | | 0 | | Mitochondria | |  |
| SELMODRAFT_135816 | | D8TAU6 | | P | | 228 | | 76 | | | GL377705:78940-79167 | | 0 | | Mitochondria | |  |
| SELMODRAFT_135822 | | D8TAT7 | | P | | 1320 | | 440 | | | GL377705:51781-53103 | | 0 | | _ | |  |
| SELMODRAFT_135826 | | D8TAX6 | | P | | 1347 | | 449 | | | GL377705:246605-248009 | | 1 | | Mitochondria | |  |
| SELMODRAFT_135827 | | D8TAV2 | | P | | 543 | | 181 | | | GL377705:93895-94620 | | 1 | | _ | |  |
| SELMODRAFT_135839 | | D8TAT8 | | P | | 747 | | 249 | | | GL377705:53894-54640 | | 0 | | _ | |  |
| SELMODRAFT_135850 | | D8TAX7 | | P | | 615 | | 205 | | | GL377705:252026-252643 | | 0 | | _ | |  |
| SELMODRAFT_135885 | | D8TAW0 | | P | | 738 | | 246 | | | GL377705:129703-130443 | | 0 | | _ | |  |
| SELMODRAFT_136037 | | D8TB70 | | P | | 1623 | | 541 | | | GL377707:292191-294440 | | 2 | | SP | |  |
| SELMODRAFT_136091 | | D8TB92 | | P | | 2274 | | 758 | | | GL377708:94756-97032 | | 0 | | _ | |  |
| SELMODRAFT_136113 | | D8TBC6 | | DYW | | 1890 | | 630 | | | GL377708:299642-302748 | | 2 | | _ | |  |
| SELMODRAFT_136118 | | D8TBB4 | | P | | 1251 | | 417 | | | GL377708:212979-214316 | | 1 | | _ | |  |
| SELMODRAFT_136134 | | D8TBB9 | | DYW | | 2148 | | 716 | | | GL377708:238307-240457 | | 0 | | _ | |  |
| SELMODRAFT_136199 | | D8TBE4 | | PLS | | 1278 | | 426 | | | GL377709:209314-210798 | | 1 | | _ | |  |
| SELMODRAFT_136241 | | D8TBE9 | | P | | 1530 | | 510 | | | GL377709:234622-236364 | | 1 | | _ | |  |
| SELMODRAFT_136281 | | D8TBM5 | | P | | 1293 | | 431 | | | GL377711:180385-181986 | | 1 | | Mitochondria | |  |
| SELMODRAFT_136285 | | D8TBQ3 | | P | | 753 | | 251 | | | GL377711:302706-303461 | | 0 | | _ | |  |
| SELMODRAFT_136306 | | D8TBM3 | | PLS | | 1737 | | 579 | | | GL377711:170791-174766 | | 2 | | _ | |  |
| SELMODRAFT_136371 | | D8TBR8 | | P | | 1008 | | 336 | | | GL377712:160803-162185 | | 1 | | _ | |  |
| SELMODRAFT_136422 | | D8TBT3 | | P | | 570 | | 190 | | | GL377712:216185-216943 | | 1 | | Mitochondria | |  |
| SELMODRAFT_136484 | | D8TBV9 | | P | | 711 | | 237 | | | GL377713:109259-109969 | | 0 | | _ | |  |
| SELMODRAFT_136503 | | D8TBW0 | | P | | 828 | | 276 | | | GL377713:111107-111937 | | 0 | | _ | |  |
| SELMODRAFT_136535 | | D8TBV8 | | P | | 1749 | | 583 | | | GL377713:106218-108473 | | 2 | | _ | |  |
| SELMODRAFT_136545 | | D8TC22 | | P | | 621 | | 207 | | | GL377714:107771-108394 | | 0 | | Chloroplast | |  |
| SELMODRAFT_136558 | | D8TC23 | | P | | 720 | | 240 | | | GL377714:108608-109327 | | 0 | | _ | |  |
| SELMODRAFT_136561 | | D8TC14 | | P | | 1707 | | 569 | | | GL377714:71972-73861 | | 1 | | _ | |  |
| SELMODRAFT_136851 | | D8TCG9 | | P | | 762 | | 254 | | | GL377717:69568-70332 | | 0 | | Mitochondria | |  |
| SELMODRAFT_136907 | | D8TCJ0 | | P | | 1512 | | 504 | | | GL377717:125290-127104 | | 1 | | _ | |  |
| SELMODRAFT_136919 | | D8TCM4 | | P | | 984 | | 328 | | | GL377718:280519-281565 | | 1 | | _ | |  |
| SELMODRAFT_136939 | | D8TCL5 | | DYW | | 2181 | | 727 | | | GL377718:259497-261887 | | 1 | | Mitochondria | |  |
| SELMODRAFT_137002 | | D8TCS3 | | P | | 561 | | 187 | | | GL377719:170223-170786 | | 0 | | SP | |  |
| SELMODRAFT_137008 | | D8TCQ1 | | P | | 579 | | 193 | | | GL377719:93916-94497 | | 0 | | _ | |  |
| SELMODRAFT_137057 | | D8TCN7 | | DYW | | 1965 | | 655 | | | GL377719:38940-40907 | | 0 | | _ | |  |
| SELMODRAFT_137234 | | D8TD76 | | P | | 1308 | | 436 | | | GL377727:19271-22553 | | 3 | | Mitochondria | |  |
| SELMODRAFT_137248 | | D8TD77 | | P | | 597 | | 199 | | | GL377727:24211-26019 | | 1 | | _ | |  |
| SELMODRAFT_137251 | | D8TD81 | | P | | 558 | | 186 | | | GL377727:84478-85038 | | 0 | | _ | |  |
| SELMODRAFT_137265 | | D8TDB3 | | P | | 1353 | | 451 | | | GL377728:176914-178324 | | 1 | | Mitochondria | |  |
| SELMODRAFT_137330 | | D8TDH5 | | P | | 1437 | | 479 | | | GL377729:170041-172185 | | 1 | | _ | |  |
| SELMODRAFT_137399 | | D8TDJ3 | | P | | 744 | | 248 | | | GL377731:14472-15866 | | 1 | | _ | |  |
| SELMODRAFT_137451 | | D8TDR4 | | P | | 858 | | 286 | | | GL377733:124345-125205 | | 0 | | _ | |  |
| SELMODRAFT_137481 | | D8TDR0 | | P | | 990 | | 330 | | | GL377733:118141-120534 | | 1 | | _ | |  |
| SELMODRAFT_137492 | | D8TDR3 | | P | | 291 | | 97 | | | GL377733:122980-123270 | | 0 | | Mitochondria | |  |
| SELMODRAFT_137493 | | D8TDR5 | | P | | 1017 | | 339 | | | GL377733:125270-126289 | | 1 | | _ | |  |
| SELMODRAFT_137550 | | D8TDV7 | | E | | 1164 | | 388 | | | GL377734:168585-170279 | | 2 | | _ | |  |
| SELMODRAFT_137607 | | D8TDW2 | | P | | 1071 | | 357 | | | GL377735:92958-94514 | | 2 | | _ | |  |
| SELMODRAFT_137632 | | D8TE01 | | P | | 1218 | | 406 | | | GL377737:46933-48678 | | 2 | | _ | |  |
| SELMODRAFT_137997 | | D8TEI7 | | DYW | | 1302 | | 434 | | | GL377743:93178-94482 | | 0 | | Mitochondria | |  |
| SELMODRAFT_138033 | | D8TEG8 | | DYW | | 2844 | | 948 | | | GL377743:23060-29316 | | 3 | | Mitochondria | |  |
| SELMODRAFT_138036 | | D8TEJ0 | | P | | 972 | | 324 | | | GL377743:99055-100743 | | 1 | | _ | |  |
| SELMODRAFT_138059 | | D8TEK9 | | E | | 1572 | | 524 | | | GL377744:95498-97072 | | 0 | | _ | |  |
| SELMODRAFT_138070 | | D8TEL4 | | P | | 900 | | 300 | | | GL377744:111974-113176 | | 1 | | _ | |  |
| SELMODRAFT_138083 | | D8TEL6 | | P | | 600 | | 200 | | | GL377744:115864-116925 | | 1 | | _ | |  |
| SELMODRAFT_138108 | | D8TEP0 | | P | | 2859 | | 953 | | | GL377745:73348-76209 | | 0 | | _ | |  |
| SELMODRAFT_138235 | | D8TEX1 | | P | | 669 | | 223 | | | GL377750:95007-98745 | | 1 | | _ | |  |
| SELMODRAFT_138241 | | D8TEX2 | | P | | 1317 | | 439 | | | GL377750:100414-102036 | | 1 | | _ | |  |
| SELMODRAFT_138440 | | D8TFC8 | | P | | 1731 | | 577 | | | GL377778:38703-40580 | | 1 | | _ | |  |
| SELMODRAFT_138844 | | D8TG72 | | P | | 1797 | | 599 | | | GL377945:18-2294 | | 9 | | _ | |  |
| SELMODRAFT_138846 | | D8TG74 | | P | | 1563 | | 521 | | | GL377945:5772-7636 | | 5 | | _ | |  |
| SELMODRAFT_138941 | | D8TGC6 | | P | | 1791 | | 597 | | | GL378009:2733-5087 | | 1 | | Mitochondria | |  |
| SELMODRAFT_138948 | | D8TGD6 | | P | | 543 | | 181 | | | GL378022:2029-2574 | | 3 | | _ | |  |
| SELMODRAFT_138963 | | D8TGE0 | | P | | 864 | | 288 | | | GL378028:1503-4800 | | 1 | | _ | |  |
| SELMODRAFT_139039 | | D8TGI6 | | P | | 975 | | 325 | | | GL378098:145-1758 | | 0 | | _ | |  |
| SELMODRAFT_139075 | | D8TGM1 | | P | | 579 | | 193 | | | GL378164:3206-3787 | | 1 | | _ | |  |
| SELMODRAFT_139540 | | D8QQI8 | | P | | 1545 | | 515 | | | GL377565:2732483-2734359 | | 0 | | _ | |  |
| SELMODRAFT_142586 | | D8R0C8 | | P | | 1509 | | 503 | | | GL377569:3353532-3355625 | | 0 | | _ | |  |
| SELMODRAFT_142860 | | D8R1X9 | | P | | 1437 | | 479 | | | GL377570:1315740-1317227 | | 1 | | _ | |  |
| SELMODRAFT_144844 | | D8R8T5 | | P | | 1953 | | 651 | | | GL377573:3434882-3437406 | | 1 | | SP | |  |
| SELMODRAFT_145138 | | D8R8X9 | | PLS | | 4062 | | 1354 | | | GL377574:2219327-2223583 | | 3 | | Mitochondria | |  |
| SELMODRAFT_150113 | | D8RUE7 | | P | | 1146 | | 382 | | | GL377590:1725782-1727025 | | 0 | | _ | |  |
| SELMODRAFT_150456 | | D8RW75 | | P | | 840 | | 280 | | | GL377592:1291262-1293292 | | 8 | | _ | |  |
| SELMODRAFT_150748 | | D8RXC4 | | P | | 1770 | | 590 | | | GL377593:1570187-1572734 | | 1 | | Mitochondria | |  |
| SELMODRAFT_152973 | | D8S6W7 | | P | | 2613 | | 871 | | | GL377604:744343-747518 | | 2 | | Mitochondria | |  |
| SELMODRAFT_155469 | | D8SHW4 | | P | | 1911 | | 637 | | | GL377620:1080390-1082824 | | 0 | | _ | |  |
| SELMODRAFT_157270 | | D8SPT3 | | P | | 1467 | | 489 | | | GL377632:93834-95303 | | 1 | | _ | |  |
| SELMODRAFT_157280 | | D8S8I5 | | P | | 1485 | | 495 | | | GL377632:435342-437726 | | 13 | | Mitochondria | |  |
| SELMODRAFT_161227 | | D8T5I0 | | P | | 1110 | | 370 | | | GL377677:328073-329774 | | 0 | | _ | |  |
| SELMODRAFT_16172 | | D8SUT5 | | P | | 447 | | 149 | | | GL377644:28196-29718 | | 1 | | _ | |  |
| SELMODRAFT_16190 | | D8SMZ7 | | P | | 453 | | 151 | | | GL377628:965266-965718 | | 0 | | Mitochondria | |  |
| SELMODRAFT_161978 | | D8T8B6 | | P | | 1302 | | 434 | | | GL377689:179374-183502 | | 3 | | _ | |  |
| SELMODRAFT_162081 | | D8T8T4 | | P | | 1527 | | 509 | | | GL377691:327342-329475 | | 0 | | Mitochondria | |  |
| SELMODRAFT_16313 | | D8SR14 | | P | | 546 | | 182 | | | GL377635:103067-103612 | | 0 | | _ | |  |
| SELMODRAFT_16355 | | D8S834 | | P | | 570 | | 190 | | | GL377606:497384-498544 | | 1 | | Mitochondria | |  |
| SELMODRAFT_16501 | | D8SJB0 | | P | | 576 | | 192 | | | GL377623:127549-129021 | | 2 | | _ | |  |
| SELMODRAFT_16502 | | D8RS82 | | P | | 576 | | 192 | | | GL377588:853297-854769 | | 2 | | _ | |  |
| SELMODRAFT_165305 | | D8QU24 | | P | | 2121 | | 707 | | | GL377567:249010-252141 | | 3 | | _ | |  |
| SELMODRAFT_16599 | | D8RJB5 | | P | | 633 | | 211 | | | GL377581:1445271-1445903 | | 0 | | _ | |  |
| SELMODRAFT_16609 | | D8R9J9 | | P | | 549 | | 183 | | | GL377574:277031-277579 | | 0 | | Mitochondria | |  |
| SELMODRAFT_16621 | | D8RTM1 | | P | | 429 | | 143 | | | GL377589:1575034-1575462 | | 0 | | Mitochondria | |  |
| SELMODRAFT_166434 | | D8QYQ9 | | PLS | | 4095 | | 1365 | | | GL377569:275038-279317 | | 0 | | _ | |  |
| SELMODRAFT_16644 | | D8SM51 | | P | | 543 | | 181 | | | GL377627:388151-388693 | | 3 | | _ | |  |
| SELMODRAFT_16651 | | D8R8I4 | | P | | 552 | | 184 | | | GL377573:2916774-2920186 | | 1 | | _ | |  |
| SELMODRAFT_16653 | | D8T5G4 | | P | | 543 | | 181 | | | GL377677:258817-260286 | | 1 | | _ | |  |
| SELMODRAFT_16655 | | D8RPE3 | | P | | 543 | | 181 | | | GL377585:1550061-1550603 | | 0 | | _ | |  |
| SELMODRAFT_16697 | | D8SMY0 | | P | | 585 | | 195 | | | GL377628:851042-851626 | | 0 | | _ | |  |
| SELMODRAFT_16715 | | D8SS80 | | P | | 348 | | 116 | | | GL377637:623611-624054 | | 1 | | _ | |  |
| SELMODRAFT_16728 | | D8RIS2 | | P | | 588 | | 196 | | | GL377581:285397-286269 | | 1 | | _ | |  |
| SELMODRAFT_167316 | | D8R2K6 | | P | | 2319 | | 773 | | | GL377570:2507056-2509737 | | 3 | | _ | |  |
| SELMODRAFT_167728 | | D8R3S2 | | DYW | | 2736 | | 912 | | | GL377571:1935088-1938197 | | 1 | | Mitochondria | |  |
| SELMODRAFT_16781 | | D8RIZ4 | | P | | 585 | | 195 | | | GL377581:590534-591208 | | 1 | | _ | |  |
| SELMODRAFT_16803 | | D8S180 | | P | | 330 | | 110 | | | GL377598:343127-343696 | | 1 | | Chloroplast | |  |
| SELMODRAFT_16804 | | D8S818 | | P | | 567 | | 189 | | | GL377606:408696-409262 | | 0 | | _ | |  |
| SELMODRAFT_16806 | | D8RF93 | | P | | 513 | | 171 | | | GL377578:709116-709628 | | 0 | | _ | |  |
| SELMODRAFT_168641 | | D8R743 | | P | | 1353 | | 451 | | | GL377573:524904-526848 | | 0 | | _ | |  |
| SELMODRAFT_16872 | | D8SCY2 | | P | | 450 | | 150 | | | GL377612:1193101-1193550 | | 0 | | _ | |  |
| SELMODRAFT_169254 | | D8R9S8 | | P | | 2193 | | 731 | | | GL377574:891495-895075 | | 15 | | _ | |  |
| SELMODRAFT_169299 | | D8R9Y7 | | DYW | | 2385 | | 795 | | | GL377574:1288282-1296074 | | 1 | | SP | |  |
| SELMODRAFT_16948 | | D8S2H1 | | P | | 573 | | 191 | | | GL377599:1738758-1739330 | | 0 | | _ | |  |
| SELMODRAFT_169661 | | D8RAK7 | | P | | 1596 | | 532 | | | GL377575:383794-385716 | | 6 | | Chloroplast | |  |
| SELMODRAFT_16979 | | D8SLJ0 | | P | | 585 | | 195 | | | GL377626:676846-678465 | | 2 | | _ | |  |
| SELMODRAFT_17005 | | D8SF36 | | P | | 366 | | 122 | | | GL377616:964337-965824 | | 1 | | _ | |  |
| SELMODRAFT_17031 | | D8S817 | | P | | 510 | | 170 | | | GL377606:407445-408527 | | 1 | | Mitochondria | |  |
| SELMODRAFT_170452 | | D8RD94 | | P | | 1479 | | 493 | | | GL377576:2552166-2554104 | | 1 | | _ | |  |
| SELMODRAFT_171135 | | D8RFY1 | | DYW | | 2487 | | 829 | | | GL377578:2010222-2013721 | | 1 | | _ | |  |
| SELMODRAFT_17157 | | D8RLG1 | | P | | 504 | | 168 | | | GL377583:2317060-2317780 | | 1 | | _ | |  |
| SELMODRAFT_172234 | | D8RKL4 | | P | | 1071 | | 357 | | | GL377582:1535790-1537367 | | 1 | | _ | |  |
| SELMODRAFT_172645 | | D8RMC4 | | E | | 864 | | 288 | | | GL377584:246865-248281 | | 1 | | _ | |  |
| SELMODRAFT_173093 | | D8RP72 | | P | | 1605 | | 535 | | | GL377585:1226161-1228055 | | 0 | | _ | |  |
| SELMODRAFT_173113 | | D8RPB7 | | P | | 1803 | | 601 | | | GL377585:1414419-1418934 | | 6 | | _ | |  |
| SELMODRAFT_173139 | | D8RPI7 | | P | | 1872 | | 624 | | | GL377585:1867827-1869701 | | 0 | | SP | |  |
| SELMODRAFT_174049 | | D8RT85 | | P | | 1341 | | 447 | | | GL377589:814753-818030 | | 5 | | Mitochondria | |  |
| SELMODRAFT_174459 | | D8RUC2 | | P | | 570 | | 190 | | | GL377590:1658758-1659581 | | 0 | | _ | |  |
| SELMODRAFT_177686 | | D8S7Y4 | | P | | 1500 | | 500 | | | GL377606:198240-200144 | | 0 | | SP | |  |
| SELMODRAFT_178081 | | D8S9Q3 | | P | | 1665 | | 555 | | | GL377608:1009769-1011788 | | 0 | | _ | |  |
| SELMODRAFT_179010 | | D8SE50 | | P | | 1176 | | 392 | | | GL377614:1226128-1227790 | | 1 | | SP | |  |
| SELMODRAFT_179704 | | D8SHA9 | | DYW | | 1956 | | 652 | | | GL377619:1322911-1325436 | | 0 | | _ | |  |
| SELMODRAFT_180422 | | D8SK38 | | P | | 1476 | | 492 | | | GL377624:603073-604702 | | 5 | | _ | |  |
| SELMODRAFT_180944 | | D8SM22 | | DYW | | 1776 | | 592 | | | GL377627:202033-212393 | | 1 | | Mitochondria | |  |
| SELMODRAFT_181046 | | D8SMC9 | | E | | 2376 | | 792 | | | GL377627:734767-737624 | | 0 | | SP | |  |
| SELMODRAFT_181879 | | D8SQM8 | | P | | 1341 | | 447 | | | GL377634:349395-352667 | | 0 | | Mitochondria | |  |
| SELMODRAFT_182148 | | D8SRT2 | | DYW | | 1812 | | 604 | | | GL377636:527676-529636 | | 0 | | Chloroplast | |  |
| SELMODRAFT_182249 | | D8SSA7 | | P | | 1827 | | 609 | | | GL377637:775068-777701 | | 2 | | _ | |  |
| SELMODRAFT_18228 | | D8RFD6 | | P | | 249 | | 83 | | | GL377578:904038-904286 | | 1 | | _ | |  |
| SELMODRAFT_182414 | | D8ST55 | | P | | 2319 | | 773 | | | GL377639:413069-415730 | | 3 | | _ | |  |
| SELMODRAFT_182822 | | D8SUL0 | | P | | 2082 | | 694 | | | GL377643:295060-297178 | | 0 | | SP | |  |
| SELMODRAFT_185992 | | D8T6V4 | | P | | 900 | | 300 | | | GL377683:196808-199232 | | 1 | | _ | |  |
| SELMODRAFT_186379 | | D8T8A7 | | P | | 1215 | | 405 | | | GL377689:131625-133497 | | 1 | | _ | |  |
| SELMODRAFT_186416 | | D8T8E7 | | P | | 1065 | | 355 | | | GL377689:383247-385177 | | 2 | | Mitochondria | |  |
| SELMODRAFT_187379 | | D8TCJ4 | | P | | 1149 | | 383 | | | GL377717:175199-176695 | | 1 | | Mitochondria | |  |
| SELMODRAFT_187482 | | D8TD84 | | P | | 1992 | | 664 | | | GL377727:89747-93089 | | 14 | | Chloroplast | |  |
| SELMODRAFT_19623 | | D8RPD9 | | P | | 219 | | 73 | | | GL377585:1540549-1540767 | | 0 | | _ | |  |
| SELMODRAFT_19624 | | D8SM47 | | P | | 225 | | 75 | | | GL377627:378518-378742 | | 0 | | Mitochondria | |  |
| SELMODRAFT_19658 | | D8S496 | | P | | 258 | | 86 | | | GL377601:1081141-1081398 | | 0 | | _ | |  |
| SELMODRAFT_19675 | | D8RNS4 | | P | | 261 | | 87 | | | GL377585:609402-609662 | | 0 | | _ | |  |
| SELMODRAFT_20225 | | D8TBY7 | | P | | 684 | | 228 | | | GL377713:232030-247509 | | 1 | | Mitochondria | |  |
| SELMODRAFT_20442 | | D8T2C3 | | E | | 2247 | | 749 | | | GL377665:361286-365274 | | 3 | | _ | |  |
| SELMODRAFT_20534 | | D8TFD8 | | P | | 663 | | 221 | | | GL377782:30697-41546 | | 2 | | Mitochondria | |  |
| SELMODRAFT_20613 | | D8SM36 | | P | | 1650 | | 550 | | | GL377627:326311-327960 | | 0 | | _ | |  |
| SELMODRAFT_20633 | | D8R2T1 | | P | | 1812 | | 604 | | | GL377570:2836466-2838745 | | 1 | | _ | |  |
| SELMODRAFT_20977 | | D8SNV9 | | P | | 1413 | | 471 | | | GL377630:563349-564761 | | 0 | | _ | |  |
| SELMODRAFT_21174 | | D8SPS1 | | P | | 1377 | | 459 | | | GL377632:45161-46537 | | 0 | | _ | |  |
| SELMODRAFT_21211 | | D8QN94 | | P | | 1491 | | 497 | | | GL377565:5074951-5076516 | | 1 | | _ | |  |
| SELMODRAFT_21339 | | D8SUU7 | | P | | 1425 | | 475 | | | GL377644:80449-81873 | | 0 | | _ | |  |
| SELMODRAFT_21427 | | D8QT01 | | P | | 984 | | 328 | | | GL377566:5527941-5535698 | | 2 | | _ | |  |
| SELMODRAFT_21446 | | D8T227 | | P | | 1392 | | 464 | | | GL377664:512738-514804 | | 1 | | Mitochondria | |  |
| SELMODRAFT_21751 | | D8T8J5 | | P | | 675 | | 225 | | | GL377690:250327-251370 | | 1 | | _ | |  |
| SELMODRAFT_21801 | | D8QRM9 | | P | | 1560 | | 520 | | | GL377566:2898899-2900458 | | 0 | | _ | |  |
| SELMODRAFT_21888 | | D8R0B5 | | P | | 1236 | | 412 | | | GL377569:3175827-3177062 | | 0 | | SP | |  |
| SELMODRAFT_21981 | | D8T1W8 | | P | | 1104 | | 368 | | | GL377663:660409-661512 | | 0 | | _ | |  |
| SELMODRAFT_22115 | | D8QQI9 | | E | | 1194 | | 398 | | | GL377565:2739670-2741304 | | 2 | | Mitochondria | |  |
| SELMODRAFT_22375 | | D8SX00 | | P | | 1188 | | 396 | | | GL377649:762445-763824 | | 1 | | Mitochondria | |  |
| SELMODRAFT_22638 | | D8SRW3 | | P | | 1128 | | 376 | | | GL377636:683179-684675 | | 1 | | _ | |  |
| SELMODRAFT_22845 | | D8T241 | | P | | 1011 | | 337 | | | GL377664:559717-560943 | | 1 | | _ | |  |
| SELMODRAFT_228785 | | D8SEY0 | | P | | 1911 | | 637 | | | GL377616:502214-504603 | | 0 | | _ | |  |
| SELMODRAFT_22884 | | D8STX8 | | P | | 1161 | | 387 | | | GL377641:272511-274244 | | 1 | | _ | |  |
| SELMODRAFT_22978 | | D8SQ98 | | P | | 1044 | | 348 | | | GL377633:373528-374571 | | 0 | | _ | |  |
| SELMODRAFT_231012 | | D8R893 | | PLS | | 1638 | | 546 | | | GL377573:2514900-2516540 | | 0 | | _ | |  |
| SELMODRAFT_23607 | | D8T847 | | P | | 897 | | 299 | | | GL377688:245042-246547 | | 1 | | _ | |  |
| SELMODRAFT_236212 | | D8T6C6 | | P | | 1842 | | 614 | | | GL377680:367933-369855 | | 1 | | _ | |  |
| SELMODRAFT_23653 | | D8T2Q7 | | P | | 1059 | | 353 | | | GL377666:529089-530147 | | 0 | | _ | |  |
| SELMODRAFT_23654 | | D8T8Q6 | | P | | 1059 | | 353 | | | GL377691:174072-175130 | | 0 | | _ | |  |
| SELMODRAFT_24095 | | D8SWP3 | | P | | 666 | | 222 | | | GL377649:95289-95954 | | 0 | | _ | |  |
| SELMODRAFT_24174 | | D8SLX9 | | P | | 756 | | 252 | | | GL377627:76547-77761 | | 1 | | Mitochondria | |  |
| SELMODRAFT_24361 | | D8QTY0 | | P | | 846 | | 282 | | | GL377566:1973123-1973968 | | 0 | | _ | |  |
| SELMODRAFT_24454 | | D8R2T2 | | P | | 858 | | 286 | | | GL377570:2839411-2840268 | | 0 | | _ | |  |
| SELMODRAFT_24718 | | D8QR24 | | P | | 888 | | 296 | | | GL377565:3561141-3563330 | | 1 | | Mitochondria | |  |
| SELMODRAFT_25760 | | D8SMX9 | | P | | 666 | | 222 | | | GL377628:849293-849958 | | 0 | | _ | |  |
| SELMODRAFT_25908 | | D8SNV3 | | P | | 657 | | 219 | | | GL377630:539727-540383 | | 0 | | _ | |  |
| SELMODRAFT_25915 | | D8SQP1 | | P | | 705 | | 235 | | | GL377634:398332-399036 | | 0 | | _ | |  |
| SELMODRAFT_25944 | | D8T8I4 | | P | | 675 | | 225 | | | GL377690:207188-207862 | | 0 | | Mitochondria | |  |
| SELMODRAFT_25995 | | D8SX33 | | P | | 642 | | 214 | | | GL377650:177724-178365 | | 0 | | _ | |  |
| SELMODRAFT_264 | | D8QSE3 | | P | | 2802 | | 934 | | | GL377566:4460785-4463844 | | 0 | | _ | |  |
| SELMODRAFT_267 | | D8SKR2 | | P | | 2670 | | 890 | | | GL377625:452980-455925 | | 1 | | _ | |  |
| SELMODRAFT_26721 | | D8QYM3 | | P | | 465 | | 155 | | | GL377569:134961-135425 | | 0 | | _ | |  |
| SELMODRAFT_26754 | | D8R4G2 | | P | | 540 | | 180 | | | GL377571:3180198-3181364 | | 1 | | Mitochondria | |  |
| SELMODRAFT_26786 | | D8QRJ3 | | P | | 633 | | 211 | | | GL377566:2696414-2697046 | | 0 | | _ | |  |
| SELMODRAFT_268 | | D8QPR5 | | P | | 2670 | | 890 | | | GL377565:1084059-1087004 | | 1 | | _ | |  |
| SELMODRAFT_26900 | | D8QS24 | | P | | 588 | | 196 | | | GL377566:3740516-3741388 | | 1 | | _ | |  |
| SELMODRAFT_26969 | | D8QYD5 | | P | | 615 | | 205 | | | GL377568:2496444-2497359 | | 1 | | _ | |  |
| SELMODRAFT_26992 | | D8R111 | | P | | 321 | | 107 | | | GL377570:3488420-3488980 | | 1 | | Chloroplast | |  |
| SELMODRAFT_26993 | | D8QNL9 | | P | | 606 | | 202 | | | GL377565:5735708-5736625 | | 1 | | Mitochondria | |  |
| SELMODRAFT_27025 | | D8R4P6 | | P | | 573 | | 191 | | | GL377571:3718706-3719278 | | 0 | | _ | |  |
| SELMODRAFT_27032 | | D8R3D9 | | P | | 603 | | 201 | | | GL377571:1225740-1226342 | | 0 | | _ | |  |
| SELMODRAFT_27061 | | D8QYA7 | | P | | 579 | | 193 | | | GL377568:2391161-2391739 | | 0 | | _ | |  |
| SELMODRAFT_28702 | | D8TGF8 | | P | | 657 | | 219 | | | GL378052:3247-3903 | | 0 | | Mitochondria | |  |
| SELMODRAFT_29419 | | D8TDR6 | | P | | 273 | | 91 | | | GL377733:126401-126673 | | 0 | | Mitochondria | |  |
| SELMODRAFT_2948 | | D8TAT2 | | P | | 1023 | | 341 | | | GL377704:318507-319721 | | 1 | | _ | |  |
| SELMODRAFT_299 | | D8SKB8 | | P | | 2733 | | 911 | | | GL377624:1077100-1079832 | | 0 | | _ | |  |
| SELMODRAFT_30076 | | D8RPV9 | | P | | 984 | | 328 | | | GL377586:1053130-1054456 | | 3 | | Chloroplast | |  |
| SELMODRAFT_30095 | | D8SKU1 | | DYW | | 2235 | | 745 | | | GL377625:569938-572237 | | 1 | | _ | |  |
| SELMODRAFT_30550 | | D8R0Z9 | | P | | 2109 | | 703 | | | GL377570:3413199-3416519 | | 2 | | _ | |  |
| SELMODRAFT_30579 | | D8R7S1 | | P | | 1707 | | 569 | | | GL377573:1682320-1687931 | | 2 | | _ | |  |
| SELMODRAFT_30598 | | D8SMS2 | | P | | 1953 | | 651 | | | GL377628:537997-542452 | | 7 | | _ | |  |
| SELMODRAFT_30615 | | D8S5P9 | | P | | 696 | | 232 | | | GL377603:462332-485794 | | 2 | | _ | |  |
| SELMODRAFT_30641 | | D8R7J3 | | P | | 1518 | | 506 | | | GL377573:1377798-1390664 | | 3 | | Mitochondria | |  |
| SELMODRAFT_30677 | | D8S7T4 | | P | | 1938 | | 646 | | | GL377605:1692647-1694755 | | 1 | | _ | |  |
| SELMODRAFT_30946 | | D8S3I2 | | P | | 1947 | | 649 | | | GL377600:1457522-1459669 | | 2 | | Mitochondria | |  |
| SELMODRAFT_30949 | | D8RTN9 | | P | | 1566 | | 522 | | | GL377589:1699145-1701124 | | 2 | | _ | |  |
| SELMODRAFT_30966 | | D8SJP7 | | P | | 1887 | | 629 | | | GL377623:960244-962775 | | 1 | | Mitochondria | |  |
| SELMODRAFT_31034 | | D8R830 | | P | | 1680 | | 560 | | | GL377573:2253782-2256403 | | 2 | | _ | |  |
| SELMODRAFT_31105 | | D8RIP7 | | P | | 1167 | | 389 | | | GL377581:82543-91243 | | 1 | | _ | |  |
| SELMODRAFT_31233 | | D8QTY4 | | P | | 1365 | | 455 | | | GL377566:2061868-2063232 | | 0 | | _ | |  |
| SELMODRAFT_31242 | | D8R865 | | P | | 1254 | | 418 | | | GL377573:2389907-2391160 | | 0 | | Mitochondria | |  |
| SELMODRAFT_31382 | | D8T6T2 | | P | | 1743 | | 581 | | | GL377683:86354-88198 | | 1 | | _ | |  |
| SELMODRAFT_31384 | | D8SX46 | | P | | 1743 | | 581 | | | GL377650:410573-412417 | | 1 | | _ | |  |
| SELMODRAFT_31438 | | D8RVZ9 | | P | | 1683 | | 561 | | | GL377592:124728-126803 | | 3 | | _ | |  |
| SELMODRAFT_31516 | | D8RIV9 | | P | | 1278 | | 426 | | | GL377581:445339-447138 | | 1 | | _ | |  |
| SELMODRAFT_31610 | | D8RQ79 | | P | | 1527 | | 509 | | | GL377586:1711375-1714074 | | 1 | | Mitochondria | |  |
| SELMODRAFT_31619 | | D8R3G3 | | P | | 1488 | | 496 | | | GL377571:1287783-1289999 | | 1 | | _ | |  |
| SELMODRAFT_31620 | | D8SI51 | | P | | 1488 | | 496 | | | GL377621:414488-416704 | | 1 | | _ | |  |
| SELMODRAFT_31715 | | D8RUR7 | | P | | 1476 | | 492 | | | GL377590:470080-471846 | | 1 | | _ | |  |
| SELMODRAFT_31733 | | D8QZN6 | | P | | 1284 | | 428 | | | GL377569:1807995-1809362 | | 1 | | _ | |  |
| SELMODRAFT_31776 | | D8QP18 | | P | | 1452 | | 484 | | | GL377565:6626232-6627683 | | 0 | | _ | |  |
| SELMODRAFT_31855 | | D8SA00 | | P | | 1422 | | 474 | | | GL377608:1648206-1649990 | | 1 | | _ | |  |
| SELMODRAFT_31893 | | D8R070 | | P | | 1314 | | 438 | | | GL377569:2860248-2862413 | | 2 | | Mitochondria | |  |
| SELMODRAFT_31963 | | D8R8L9 | | P | | 1446 | | 482 | | | GL377573:3053995-3055950 | | 2 | | Mitochondria | |  |
| SELMODRAFT_32021 | | D8SB73 | | P | | 1224 | | 408 | | | GL377610:468357-469781 | | 1 | | _ | |  |
| SELMODRAFT_32257 | | D8SAW5 | | P | | 1371 | | 457 | | | GL377609:646455-648137 | | 2 | | Mitochondria | |  |
| SELMODRAFT_32263 | | D8SD38 | | P | | 1401 | | 467 | | | GL377612:1435919-1437319 | | 0 | | _ | |  |
| SELMODRAFT_32269 | | D8R8E0 | | E | | 1185 | | 395 | | | GL377573:2746480-2748120 | | 1 | | Mitochondria | |  |
| SELMODRAFT_32333 | | D8QX66 | | P | | 1122 | | 374 | | | GL377568:4077746-4079206 | | 2 | | _ | |  |
| SELMODRAFT_32366 | | D8RSF9 | | P | | 1167 | | 389 | | | GL377588:1315479-1316762 | | 1 | | Mitochondria | |  |
| SELMODRAFT_32460 | | D8S1S7 | | P | | 1008 | | 336 | | | GL377599:522082-526947 | | 2 | | _ | |  |
| SELMODRAFT_32476 | | D8QUL5 | | P | | 714 | | 238 | | | GL377567:1184738-1185451 | | 0 | | _ | |  |
| SELMODRAFT_32515 | | D8RN30 | | P | | 1023 | | 341 | | | GL377584:1552155-1553177 | | 0 | | _ | |  |
| SELMODRAFT_327 | | D8T4J0 | | P | | 2751 | | 917 | | | GL377674:187851-190907 | | 1 | | _ | |  |
| SELMODRAFT_328 | | D8SK28 | | P | | 2751 | | 917 | | | GL377624:523519-526575 | | 2 | | _ | |  |
| SELMODRAFT_32917 | | D8RA97 | | P | | 891 | | 297 | | | GL377574:1930082-1930972 | | 0 | | _ | |  |
| SELMODRAFT_32924 | | D8RP71 | | E | | 1098 | | 366 | | | GL377585:1224042-1225220 | | 1 | | _ | |  |
| SELMODRAFT_33065 | | D8SJF4 | | P | | 939 | | 313 | | | GL377623:431248-432372 | | 1 | | SP | |  |
| SELMODRAFT_33143 | | D8R3M1 | | P | | 981 | | 327 | | | GL377571:1631223-1632833 | | 2 | | _ | |  |
| SELMODRAFT_33144 | | D8QQR6 | | P | | 1116 | | 372 | | | GL377565:3143410-3144597 | | 1 | | _ | |  |
| SELMODRAFT_33173 | | D8R3H6 | | P | | 1116 | | 372 | | | GL377571:1316791-1318185 | | 2 | | _ | |  |
| SELMODRAFT_33257 | | D8RPK4 | | P | | 966 | | 322 | | | GL377585:1914691-1916196 | | 2 | | _ | |  |
| SELMODRAFT_334 | | D8SCP4 | | P | | 2499 | | 833 | | | GL377612:695200-697698 | | 0 | | Mitochondria | |  |
| SELMODRAFT_33898 | | D8QXA5 | | P | | 948 | | 316 | | | GL377568:4239677-4241452 | | 2 | | SP | |  |
| SELMODRAFT_33936 | | D8R9K3 | | P | | 870 | | 290 | | | GL377574:349119-350825 | | 2 | | _ | |  |
| SELMODRAFT_34012 | | D8SK67 | | P | | 903 | | 301 | | | GL377624:748139-749308 | | 6 | | _ | |  |
| SELMODRAFT_34152 | | D8RJR2 | | PLS | | 888 | | 296 | | | GL377581:2219041-2220579 | | 1 | | _ | |  |
| SELMODRAFT_34237 | | D8SCN5 | | P | | 870 | | 290 | | | GL377612:665676-667448 | | 1 | | _ | |  |
| SELMODRAFT_34426 | | D8S348 | | E | | 969 | | 323 | | | GL377600:743416-744960 | | 1 | | SP | |  |
| SELMODRAFT_3444 | | D8TBB3 | | P | | 882 | | 294 | | | GL377708:210930-211811 | | 0 | | Mitochondria | |  |
| SELMODRAFT_34484 | | D8RQ24 | | P | | 876 | | 292 | | | GL377586:1393072-1394814 | | 1 | | _ | |  |
| SELMODRAFT_34488 | | D8RS03 | | P | | 882 | | 294 | | | GL377588:375537-377477 | | 2 | | _ | |  |
| SELMODRAFT_34491 | | D8RPB6 | | P | | 894 | | 298 | | | GL377585:1411811-1413277 | | 1 | | _ | |  |
| SELMODRAFT_34582 | | D8QXP5 | | P | | 804 | | 268 | | | GL377568:1165462-1167000 | | 1 | | _ | |  |
| SELMODRAFT_34609 | | D8SEC4 | | P | | 882 | | 294 | | | GL377615:1005575-1007293 | | 1 | | _ | |  |
| SELMODRAFT_34638 | | D8SCM1 | | P | | 753 | | 251 | | | GL377612:574296-575654 | | 1 | | _ | |  |
| SELMODRAFT_34765 | | D8S1D6 | | P | | 885 | | 295 | | | GL377598:578820-580301 | | 1 | | _ | |  |
| SELMODRAFT_34767 | | D8R164 | | P | | 885 | | 295 | | | GL377570:3724549-3726030 | | 1 | | _ | |  |
| SELMODRAFT_34785 | | D8RNY0 | | P | | 858 | | 286 | | | GL377585:795181-797427 | | 2 | | _ | |  |
| SELMODRAFT_34928 | | D8QQM3 | | P | | 723 | | 241 | | | GL377565:2954957-2956297 | | 1 | | _ | |  |
| SELMODRAFT_35138 | | D8SD24 | | P | | 816 | | 272 | | | GL377612:1381232-1382829 | | 2 | | _ | |  |
| SELMODRAFT_35195 | | D8RDN7 | | P | | 870 | | 290 | | | GL377577:71586-72947 | | 1 | | _ | |  |
| SELMODRAFT_35949 | | D8SLI6 | | P | | 681 | | 227 | | | GL377626:666622-667302 | | 0 | | _ | |  |
| SELMODRAFT_35950 | | D8RMF3 | | P | | 693 | | 231 | | | GL377584:435288-435980 | | 0 | | _ | |  |
| SELMODRAFT_36105 | | D8S083 | | P | | 696 | | 232 | | | GL377596:1614344-1618291 | | 1 | | Mitochondria | |  |
| SELMODRAFT_36171 | | D8RDG7 | | E | | 789 | | 263 | | | GL377576:2828593-2829513 | | 1 | | _ | |  |
| SELMODRAFT_36212 | | D8RQS1 | | P | | 597 | | 199 | | | GL377586:555287-556816 | | 1 | | Mitochondria | |  |
| SELMODRAFT_36220 | | D8RRN7 | | P | | 633 | | 211 | | | GL377587:1595463-1596371 | | 1 | | _ | |  |
| SELMODRAFT_36319 | | D8S515 | | P | | 657 | | 219 | | | GL377602:698626-699282 | | 0 | | _ | |  |
| SELMODRAFT_36321 | | D8QZ97 | | P | | 651 | | 217 | | | GL377569:1216007-1218058 | | 1 | | Mitochondria | |  |
| SELMODRAFT_36327 | | D8RKV4 | | P | | 549 | | 183 | | | GL377582:1989500-1990048 | | 0 | | _ | |  |
| SELMODRAFT_36412 | | D8RGE8 | | P | | 657 | | 219 | | | GL377579:763087-763743 | | 0 | | _ | |  |
| SELMODRAFT_3827 | | D8TDY9 | | P | | 750 | | 250 | | | GL377736:159417-160166 | | 0 | | _ | |  |
| SELMODRAFT_3910 | | D8TCC0 | | P | | 864 | | 288 | | | GL377716:80363-81226 | | 0 | | _ | |  |
| SELMODRAFT_399 | | D8R605 | | P | | 2196 | | 732 | | | GL377572:1449599-1451794 | | 0 | | _ | |  |
| SELMODRAFT_402120 | | D8QPN2 | | P | | 2031 | | 677 | | | GL377565:871951-874527 | | 4 | | Mitochondria | |  |
| SELMODRAFT_402147 | | D8QPR3 | | P | | 981 | | 327 | | | GL377565:1079187-1080597 | | 1 | | _ | |  |
| SELMODRAFT_402152 | | D8QPS2 | | P | | 1200 | | 400 | | | GL377565:1099915-1101301 | | 1 | | Mitochondria | |  |
| SELMODRAFT_402234 | | D8QQ06 | | P | | 1755 | | 585 | | | GL377565:1599941-1601796 | | 1 | | Mitochondria | |  |
| SELMODRAFT_402968 | | D8QNM4 | | P | | 579 | | 193 | | | GL377565:5745976-5747022 | | 1 | | _ | |  |
| SELMODRAFT_403158 | | D8QP86 | | P | | 747 | | 249 | | | GL377565:6920054-6921166 | | 1 | | _ | |  |
| SELMODRAFT_403170 | | D8QTB5 | | P | | 903 | | 301 | | | GL377566:88750-90603 | | 3 | | _ | |  |
| SELMODRAFT_403193 | | D8QTD5 | | P | | 1071 | | 357 | | | GL377566:299153-300293 | | 1 | | _ | |  |
| SELMODRAFT_40326 | | D8SV43 | | P | | 1047 | | 349 | | | GL377644:607427-631845 | | 3 | | _ | |  |
| SELMODRAFT_403786 | | D8QSJ0 | | P | | 1635 | | 545 | | | GL377566:4760904-4762541 | | 0 | | Mitochondria | |  |
| SELMODRAFT_404011 | | D8QTA6 | | P | | 708 | | 236 | | | GL377566:6157750-6159478 | | 3 | | _ | |  |
| SELMODRAFT_404389 | | D8QV64 | | P | | 3093 | | 1031 | | | GL377567:2197080-2200683 | | 3 | | Chloroplast | |  |
| SELMODRAFT_404554 | | D8QVQ0 | | P | | 4194 | | 1398 | | | GL377567:3128885-3134404 | | 17 | | Mitochondria | |  |
| SELMODRAFT_404881 | | D8QXN4 | | P | | 1332 | | 444 | | | GL377568:999823-1001546 | | 2 | | Mitochondria | |  |
| SELMODRAFT_404884 | | D8QXN8 | | P | | 594 | | 198 | | | GL377568:1076418-1077315 | | 2 | | _ | |  |
| SELMODRAFT_405034 | | D8QY63 | | P | | 615 | | 205 | | | GL377568:2197148-2198405 | | 1 | | _ | |  |
| SELMODRAFT_405530 | | D8QYW1 | | P | | 1368 | | 456 | | | GL377569:478780-480276 | | 1 | | Mitochondria | |  |
| SELMODRAFT_406060 | | D8R0J4 | | P | | 3522 | | 1174 | | | GL377569:3695097-3699490 | | 5 | | _ | |  |
| SELMODRAFT_406577 | | D8R0S9 | | PLS | | 1392 | | 464 | | | GL377570:2921327-2922925 | | 2 | | _ | |  |
| SELMODRAFT_406783 | | D8R2X4 | | P | | 1752 | | 584 | | | GL377571:173996-175976 | | 3 | | Chloroplast | |  |
| SELMODRAFT_407276 | | D8R4H8 | | P | | 2847 | | 949 | | | GL377571:3347565-3351462 | | 7 | | _ | |  |
| SELMODRAFT_407278 | | D8R4I0 | | P | | 3045 | | 1015 | | | GL377571:3365420-3368687 | | 4 | | Mitochondria | |  |
| SELMODRAFT_407543 | | D8R5Y6 | | P | | 1191 | | 397 | | | GL377572:1319530-1320989 | | 1 | | Mitochondria | |  |
| SELMODRAFT_407654 | | D8R6B0 | | E | | 3438 | | 1146 | | | GL377572:1988364-1992274 | | 7 | | _ | |  |
| SELMODRAFT_407679 | | D8R6E8 | | E | | 480 | | 160 | | | GL377572:2131889-2132371 | | 0 | | _ | |  |
| SELMODRAFT_408043 | | D8R709 | | P | | 819 | | 273 | | | GL377573:409710-410730 | | 1 | | _ | |  |
| SELMODRAFT_409004 | | D8R951 | | P | | 2112 | | 704 | | | GL377574:2596453-2599296 | | 14 | | Chloroplast | |  |
| SELMODRAFT_40989 | | D8T826 | | E | | 1767 | | 589 | | | GL377688:131926-135710 | | 2 | | SP | |  |
| SELMODRAFT_410174 | | D8RDV9 | | P | | 585 | | 195 | | | GL377577:576261-577296 | | 1 | | _ | |  |
| SELMODRAFT_410602 | | D8RF94 | | P | | 1614 | | 538 | | | GL377578:709986-712337 | | 2 | | _ | |  |
| SELMODRAFT_410940 | | D8RGC8 | | P | | 531 | | 177 | | | GL377578:2812448-2813181 | | 3 | | _ | |  |
| SELMODRAFT_411 | | D8R099 | | P | | 2181 | | 727 | | | GL377569:3126083-3128263 | | 3 | | Mitochondria | |  |
| SELMODRAFT_411080 | | D8RGI7 | | P | | 1317 | | 439 | | | GL377579:979952-982013 | | 4 | | _ | |  |
| SELMODRAFT_411148 | | D8RGQ5 | | P | | 2493 | | 831 | | | GL377579:1355365-1357931 | | 1 | | _ | |  |
| SELMODRAFT_411297 | | D8RH71 | | P | | 540 | | 180 | | | GL377579:2133203-2133994 | | 2 | | _ | |  |
| SELMODRAFT_411983 | | D8RJN5 | | P | | 1752 | | 584 | | | GL377581:2050747-2052501 | | 0 | | Mitochondria | |  |
| SELMODRAFT_412349 | | D8RKV5 | | P | | 1851 | | 617 | | | GL377582:1990439-1992389 | | 1 | | _ | |  |
| SELMODRAFT_412417 | | D8RL32 | | P | | 615 | | 205 | | | GL377582:2425224-2426343 | | 1 | | _ | |  |
| SELMODRAFT_412471 | | D8RLK7 | | P | | 6156 | | 2052 | | | GL377583:198341-205942 | | 20 | | Mitochondria | |  |
| SELMODRAFT_412514 | | D8RLQ4 | | P | | 1731 | | 577 | | | GL377583:525389-527647 | | 2 | | _ | |  |
| SELMODRAFT_412519 | | D8RLR1 | | P | | 1266 | | 422 | | | GL377583:557119-558387 | | 0 | | Mitochondria | |  |
| SELMODRAFT_412643 | | D8RM57 | | P | | 1710 | | 570 | | | GL377583:1290806-1292566 | | 0 | | Mitochondria | |  |
| SELMODRAFT_412752 | | D8RLC9 | | P | | 1587 | | 529 | | | GL377583:2070436-2074928 | | 6 | | SP | |  |
| SELMODRAFT_412800 | | D8RMC1 | | P | | 825 | | 275 | | | GL377584:240007-240913 | | 1 | | Mitochondria | |  |
| SELMODRAFT_41291 | | D8STU8 | | P | | 1761 | | 587 | | | GL377641:159922-161916 | | 1 | | _ | |  |
| SELMODRAFT_413076 | | D8RN93 | | P | | 2253 | | 751 | | | GL377584:1965882-1968685 | | 3 | | Mitochondria | |  |
| SELMODRAFT_413277 | | D8RNX9 | | P | | 1212 | | 404 | | | GL377585:791296-792732 | | 1 | | _ | |  |
| SELMODRAFT_413483 | | D8RPM1 | | P | | 1641 | | 547 | | | GL377585:1969920-1971622 | | 1 | | Mitochondria | |  |
| SELMODRAFT_414000 | | D8RRB1 | | P | | 501 | | 167 | | | GL377587:999470-999973 | | 0 | | _ | |  |
| SELMODRAFT_414470 | | D8RSW2 | | P | | 3336 | | 1112 | | | GL377588:1942293-1947176 | | 13 | | SP | |  |
| SELMODRAFT_414514 | | D8RT07 | | P | | 1917 | | 639 | | | GL377589:276621-280074 | | 3 | | Mitochondria | |  |
| SELMODRAFT_41458 | | D8STI8 | | P | | 1869 | | 623 | | | GL377640:390387-393656 | | 1 | | Mitochondria | |  |
| SELMODRAFT_414874 | | D8RUX2 | | P | | 669 | | 223 | | | GL377590:877771-879063 | | 2 | | _ | |  |
| SELMODRAFT_41495 | | D8T1X0 | | PLS | | 1569 | | 523 | | | GL377663:665720-667588 | | 1 | | _ | |  |
| SELMODRAFT_415084 | | D8RUY9 | | P | | 1002 | | 334 | | | GL377591:83272-84413 | | 1 | | _ | |  |
| SELMODRAFT_415618 | | D8RWQ0 | | P | | 867 | | 289 | | | GL377593:213159-215287 | | 2 | | _ | |  |
| SELMODRAFT_415658 | | D8RWU3 | | P | | 987 | | 329 | | | GL377593:431141-432130 | | 0 | | _ | |  |
| SELMODRAFT_415777 | | D8RX76 | | P | | 498 | | 166 | | | GL377593:1300769-1301269 | | 0 | | _ | |  |
| SELMODRAFT_41586 | | D8T553 | | P | | 1728 | | 576 | | | GL377676:117212-119699 | | 3 | | _ | |  |
| SELMODRAFT_415910 | | D8RYK5 | | P | | 1809 | | 603 | | | GL377594:94209-96139 | | 2 | | Chloroplast | |  |
| SELMODRAFT_41599 | | D8T276 | | P | | 1449 | | 483 | | | GL377665:164882-166783 | | 2 | | _ | |  |
| SELMODRAFT_416001 | | D8RXS0 | | P | | 630 | | 210 | | | GL377594:679554-680186 | | 0 | | _ | |  |
| SELMODRAFT_416406 | | D8RZ68 | | P | | 804 | | 268 | | | GL377595:999520-1001007 | | 1 | | _ | |  |
| SELMODRAFT_416459 | | D8RZC5 | | P | | 1527 | | 509 | | | GL377595:1473092-1474672 | | 1 | | _ | |  |
| SELMODRAFT_417058 | | D8S183 | | P | | 1377 | | 459 | | | GL377598:354866-356670 | | 6 | | _ | |  |
| SELMODRAFT_417186 | | D8S1N3 | | P | | 960 | | 320 | | | GL377598:1171071-1172205 | | 3 | | _ | |  |
| SELMODRAFT_417360 | | D8S1Z2 | | P | | 1965 | | 655 | | | GL377599:901015-903679 | | 12 | | Mitochondria | |  |
| SELMODRAFT_417488 | | D8S2D9 | | P | | 537 | | 179 | | | GL377599:1623232-1624228 | | 1 | | _ | |  |
| SELMODRAFT_417661 | | D8S361 | | P | | 540 | | 180 | | | GL377600:831387-832534 | | 2 | | _ | |  |
| SELMODRAFT_417683 | | D8S388 | | P | | 777 | | 259 | | | GL377600:988625-989531 | | 2 | | SP | |  |
| SELMODRAFT_417741 | | D8S3G4 | | P | | 1296 | | 432 | | | GL377600:1328545-1331634 | | 6 | | _ | |  |
| SELMODRAFT_41818 | | D8SUW8 | | P | | 1605 | | 535 | | | GL377644:213590-215488 | | 13 | | _ | |  |
| SELMODRAFT_418238 | | D8S539 | | P | | 2436 | | 812 | | | GL377602:828727-834333 | | 1 | | Mitochondria | |  |
| SELMODRAFT_418275 | | D8S575 | | P | | 597 | | 199 | | | GL377602:1094010-1094744 | | 1 | | _ | |  |
| SELMODRAFT_418596 | | D8S676 | | P | | 1089 | | 363 | | | GL377603:1550827-1553167 | | 2 | | _ | |  |
| SELMODRAFT_418647 | | D8S6P9 | | P | | 420 | | 140 | | | GL377604:344909-345331 | | 0 | | _ | |  |
| SELMODRAFT_418649 | | D8S6Q1 | | P | | 333 | | 111 | | | GL377604:349962-350483 | | 2 | | SP | |  |
| SELMODRAFT_418651 | | D8S6Q3 | | P | | 525 | | 175 | | | GL377604:354816-355582 | | 2 | | Mitochondria | |  |
| SELMODRAFT_418683 | | D8S6T9 | | P | | 1452 | | 484 | | | GL377604:633860-636924 | | 8 | | SP | |  |
| SELMODRAFT_418707 | | D8S6W6 | | P | | 1674 | | 558 | | | GL377604:737912-741595 | | 10 | | Chloroplast | |  |
| SELMODRAFT_418780 | | D8S6D0 | | P | | 1161 | | 387 | | | GL377604:1366935-1368912 | | 3 | | _ | |  |
| SELMODRAFT_41900 | | D8SZL7 | | P | | 1269 | | 423 | | | GL377656:126254-127522 | | 0 | | _ | |  |
| SELMODRAFT_419677 | | D8S9P4 | | P | | 1206 | | 402 | | | GL377608:982608-984470 | | 5 | | SP | |  |
| SELMODRAFT_419901 | | D8SAW7 | | P | | 387 | | 129 | | | GL377609:667742-668238 | | 1 | | Mitochondria | |  |
| SELMODRAFT_420032 | | D8SAC1 | | P | | 4581 | | 1527 | | | GL377609:1348395-1354122 | | 16 | | SP | |  |
| SELMODRAFT_420164 | | D8SB60 | | P | | 1866 | | 622 | | | GL377610:394952-396905 | | 1 | | Mitochondria | |  |
| SELMODRAFT_420323 | | D8SBM4 | | P | | 915 | | 305 | | | GL377610:1283981-1285553 | | 1 | | Mitochondria | |  |
| SELMODRAFT_420401 | | D8SBW2 | | P | | 924 | | 308 | | | GL377611:460872-462290 | | 4 | | _ | |  |
| SELMODRAFT_420644 | | D8SCN4 | | P | | 1356 | | 452 | | | GL377612:661454-664106 | | 5 | | Mitochondria | |  |
| SELMODRAFT_420654 | | D8SCP5 | | PLS | | 2388 | | 796 | | | GL377612:707475-709865 | | 0 | | Mitochondria | |  |
| SELMODRAFT_420695 | | D8SCU0 | | P | | 1242 | | 414 | | | GL377612:1023804-1025384 | | 1 | | _ | |  |
| SELMODRAFT_420704 | | D8SCU9 | | P | | 1623 | | 541 | | | GL377612:1079922-1082073 | | 1 | | Mitochondria | |  |
| SELMODRAFT_42073 | | D8T5F7 | | P | | 1551 | | 517 | | | GL377677:218378-221062 | | 2 | | _ | |  |
| SELMODRAFT_420919 | | D8SDJ3 | | P | | 1968 | | 656 | | | GL377613:1042774-1045180 | | 2 | | _ | |  |
| SELMODRAFT_420997 | | D8SDT5 | | P | | 567 | | 189 | | | GL377614:497370-498116 | | 1 | | SP | |  |
| SELMODRAFT_421012 | | D8SDV1 | | P | | 936 | | 312 | | | GL377614:616812-618128 | | 3 | | _ | |  |
| SELMODRAFT_421019 | | D8SDV8 | | P | | 885 | | 295 | | | GL377614:672521-673674 | | 1 | | _ | |  |
| SELMODRAFT_421718 | | D8SG56 | | P | | 1125 | | 375 | | | GL377618:673990-675434 | | 2 | | Mitochondria | |  |
| SELMODRAFT_421954 | | D8SGW4 | | PLS | | 2388 | | 796 | | | GL377619:593528-595918 | | 0 | | Mitochondria | |  |
| SELMODRAFT_421991 | | D8SH00 | | P | | 1242 | | 414 | | | GL377619:838321-839926 | | 1 | | _ | |  |
| SELMODRAFT_421992 | | D8SH01 | | P | | 1461 | | 487 | | | GL377619:840505-842076 | | 2 | | _ | |  |
| SELMODRAFT_421999 | | D8SH09 | | P | | 3414 | | 1138 | | | GL377619:885528-889355 | | 3 | | Mitochondria | |  |
| SELMODRAFT_422000 | | D8SH10 | | P | | 1623 | | 541 | | | GL377619:889613-891811 | | 1 | | Mitochondria | |  |
| SELMODRAFT_422089 | | D8SHB2 | | P | | 654 | | 218 | | | GL377619:1366370-1367248 | | 2 | | SP | |  |
| SELMODRAFT_422560 | | D8SIU1 | | P | | 867 | | 289 | | | GL377622:427565-428677 | | 1 | | _ | |  |
| SELMODRAFT_423071 | | D8SKG9 | | P | | 1794 | | 598 | | | GL377625:25587-27481 | | 1 | | Mitochondria | |  |
| SELMODRAFT_423148 | | D8SKQ3 | | P | | 1242 | | 414 | | | GL377625:434080-435375 | | 1 | | Chloroplast | |  |
| SELMODRAFT_423155 | | D8SKR4 | | P | | 1020 | | 340 | | | GL377625:459395-460844 | | 1 | | _ | |  |
| SELMODRAFT_423223 | | D8SKZ3 | | P | | 1386 | | 462 | | | GL377625:844292-845843 | | 1 | | _ | |  |
| SELMODRAFT_423324 | | D8SLA7 | | P | | 531 | | 177 | | | GL377626:213540-214270 | | 3 | | _ | |  |
| SELMODRAFT_423566 | | D8SM35 | | P | | 1425 | | 475 | | | GL377627:320723-323058 | | 3 | | _ | |  |
| SELMODRAFT_423640 | | D8SMC6 | | P | | 1641 | | 547 | | | GL377627:727742-729444 | | 1 | | Mitochondria | |  |
| SELMODRAFT_42377 | | D8TBX0 | | P | | 1440 | | 480 | | | GL377713:153982-155733 | | 1 | | _ | |  |
| SELMODRAFT_42378 | | D8SYW7 | | P | | 1434 | | 478 | | | GL377654:211375-212808 | | 0 | | _ | |  |
| SELMODRAFT_424099 | | D8SNT4 | | P | | 4908 | | 1636 | | | GL377630:371164-378165 | | 3 | | Chloroplast | |  |
| SELMODRAFT_42424 | | D8T8P7 | | P | | 993 | | 331 | | | GL377691:66920-67912 | | 0 | | Mitochondria | |  |
| SELMODRAFT_42426 | | D8T2R4 | | P | | 993 | | 331 | | | GL377666:627879-628871 | | 0 | | Mitochondria | |  |
| SELMODRAFT_425025 | | D8SRS6 | | P | | 762 | | 254 | | | GL377636:512115-513729 | | 1 | | Mitochondria | |  |
| SELMODRAFT_425365 | | D8SSV8 | | P | | 2676 | | 892 | | | GL377638:853825-858957 | | 15 | | Chloroplast | |  |
| SELMODRAFT_425515 | | D8STC5 | | P | | 567 | | 189 | | | GL377639:825862-826743 | | 1 | | _ | |  |
| SELMODRAFT_425548 | | D8STG6 | | P | | 492 | | 164 | | | GL377640:160966-161917 | | 4 | | _ | |  |
| SELMODRAFT_425944 | | D8SUU0 | | P | | 396 | | 132 | | | GL377644:60037-61151 | | 2 | | Mitochondria | |  |
| SELMODRAFT_425968 | | D8SUX2 | | P | | 492 | | 164 | | | GL377644:224659-225413 | | 1 | | _ | |  |
| SELMODRAFT_426357 | | D8SW45 | | P | | 2199 | | 733 | | | GL377647:601379-604720 | | 5 | | Mitochondria | |  |
| SELMODRAFT_42645 | | D8T9V0 | | P | | 1344 | | 448 | | | GL377699:67227-68570 | | 0 | | _ | |  |
| SELMODRAFT_426493 | | D8SWJ3 | | P | | 2196 | | 732 | | | GL377648:656155-659490 | | 0 | | Mitochondria | |  |
| SELMODRAFT_426864 | | D8SXR5 | | P | | 654 | | 218 | | | GL377651:714342-715849 | | 1 | | _ | |  |
| SELMODRAFT_427147 | | D8SYN3 | | P | | 504 | | 168 | | | GL377653:539896-540647 | | 3 | | _ | |  |
| SELMODRAFT_427387 | | D8SZF4 | | P | | 1416 | | 472 | | | GL377655:319315-320733 | | 0 | | Chloroplast | |  |
| SELMODRAFT_427987 | | D8T1C2 | | P | | 606 | | 202 | | | GL377662:147317-148510 | | 1 | | _ | |  |
| SELMODRAFT_428038 | | D8T1I0 | | P | | 2235 | | 745 | | | GL377662:460355-463030 | | 1 | | Mitochondria | |  |
| SELMODRAFT_428276 | | D8T2B3 | | P | | 1485 | | 495 | | | GL377665:327482-329034 | | 1 | | Chloroplast | |  |
| SELMODRAFT_428411 | | D8T2R0 | | P | | 327 | | 109 | | | GL377666:583832-584434 | | 1 | | Mitochondria | |  |
| SELMODRAFT_428635 | | D8T3H4 | | P | | 603 | | 201 | | | GL377669:607325-608278 | | 3 | | _ | |  |
| SELMODRAFT_428941 | | D8T4I5 | | P | | 360 | | 120 | | | GL377674:165278-165699 | | 1 | | _ | |  |
| SELMODRAFT_429174 | | D8T5A2 | | P | | 603 | | 201 | | | GL377676:597453-598406 | | 4 | | _ | |  |
| SELMODRAFT_429180 | | D8T5A8 | | P | | 555 | | 185 | | | GL377677:4133-5671 | | 1 | | _ | |  |
| SELMODRAFT_429215 | | D8T5E9 | | DYW | | 936 | | 312 | | | GL377677:201270-203159 | | 1 | | _ | |  |
| SELMODRAFT_429783 | | D8T7A4 | | P | | 1452 | | 484 | | | GL377685:55948-59016 | | 8 | | SP | |  |
| SELMODRAFT_430025 | | D8T831 | | P | | 924 | | 308 | | | GL377688:150183-151214 | | 1 | | _ | |  |
| SELMODRAFT_430370 | | D8T973 | | P | | 1284 | | 428 | | | GL377694:202861-204275 | | 2 | | Chloroplast | |  |
| SELMODRAFT_430583 | | D8T9V4 | | P | | 2112 | | 704 | | | GL377699:78684-81528 | | 14 | | Chloroplast | |  |
| SELMODRAFT_430638 | | D8TA13 | | P | | 603 | | 201 | | | GL377700:5657-6610 | | 4 | | _ | |  |
| SELMODRAFT_431017 | | D8TB94 | | P | | 1752 | | 584 | | | GL377708:131418-133229 | | 1 | | Mitochondria | |  |
| SELMODRAFT_431021 | | D8TB98 | | P | | 1803 | | 601 | | | GL377708:152158-154482 | | 5 | | Mitochondria | |  |
| SELMODRAFT_431043 | | D8TBC4 | | DYW | | 1851 | | 617 | | | GL377708:286409-289394 | | 4 | | Mitochondria | |  |
| SELMODRAFT_431170 | | D8TBR7 | | P | | 942 | | 314 | | | GL377712:156836-158158 | | 1 | | _ | |  |
| SELMODRAFT_431180 | | D8TBS7 | | P | | 1710 | | 570 | | | GL377712:197877-199637 | | 1 | | Mitochondria | |  |
| SELMODRAFT_431539 | | D8TCZ9 | | P | | 531 | | 177 | | | GL377723:122147-122877 | | 3 | | _ | |  |
| SELMODRAFT_43212 | | D8SKW1 | | P | | 1131 | | 377 | | | GL377625:707030-708250 | | 1 | | _ | |  |
| SELMODRAFT_432224 | | D8TFC7 | | P | | 2460 | | 820 | | | GL377778:34055-38174 | | 1 | | _ | |  |
| SELMODRAFT_432504 | | D8TG73 | | P | | 360 | | 120 | | | GL377945:4781-5194 | | 1 | | _ | |  |
| SELMODRAFT_432564 | | D8TGE1 | | P | | 537 | | 179 | | | GL378028:4978-5514 | | 0 | | _ | |  |
| SELMODRAFT_432572 | | D8TGE9 | | P | | 1509 | | 503 | | | GL378042:11-3244 | | 0 | | _ | |  |
| SELMODRAFT_432576 | | D8TGF3 | | PLS | | 2448 | | 816 | | | GL378047:2460-5198 | | 1 | | SP | |  |
| SELMODRAFT_43484 | | D8TAF8 | | P | | 1281 | | 427 | | | GL377702:179159-180439 | | 0 | | _ | |  |
| SELMODRAFT_43584 | | D8SM95 | | P | | 780 | | 260 | | | GL377627:642911-643964 | | 1 | | SP | |  |
| SELMODRAFT_437572 | | D8QN31 | | P | | 1134 | | 378 | | | GL377565:4798604-4799807 | | 1 | | _ | |  |
| SELMODRAFT_438275 | | D8QVL3 | | P | | 6222 | | 2074 | | | GL377567:2961009-2968440 | | 2 | | Mitochondria | |  |
| SELMODRAFT_43839 | | D8TG13 | | P | | 1206 | | 402 | | | GL377891:1353-3448 | | 1 | | _ | |  |
| SELMODRAFT_438810 | | D8QZL2 | | P | | 2757 | | 919 | | | GL377569:1639233-1642071 | | 1 | | Chloroplast | |  |
| SELMODRAFT_439165 | | D8R0V9 | | P | | 1038 | | 346 | | | GL377570:3058804-3060152 | | 0 | | _ | |  |
| SELMODRAFT_439521 | | D8R5M8 | | P | | 1359 | | 453 | | | GL377572:646517-648069 | | 0 | | _ | |  |
| SELMODRAFT_44042 | | D8SS05 | | P | | 951 | | 317 | | | GL377636:927863-932775 | | 2 | | Mitochondria | |  |
| SELMODRAFT_440917 | | D8RFF1 | | P | | 1488 | | 496 | | | GL377578:981351-983658 | | 2 | | _ | |  |
| SELMODRAFT_441099 | | D8RGG6 | | P | | 3594 | | 1198 | | | GL377579:889257-893844 | | 9 | | _ | |  |
| SELMODRAFT_441328 | | D8RIM7 | | P | | 2094 | | 698 | | | GL377580:2624913-2627248 | | 1 | | Mitochondria | |  |
| SELMODRAFT_441683 | | D8RM11 | | P | | 1065 | | 355 | | | GL377583:1051328-1052453 | | 0 | | _ | |  |
| SELMODRAFT_441948 | | D8RP20 | | P | | 2244 | | 748 | | | GL377585:981042-983850 | | 5 | | _ | |  |
| SELMODRAFT_442013 | | D8RQL7 | | P | | 2022 | | 674 | | | GL377586:220955-222979 | | 0 | | Chloroplast | |  |
| SELMODRAFT_442088 | | D8RQ60 | | P | | 846 | | 282 | | | GL377586:1487982-1489282 | | 1 | | _ | |  |
| SELMODRAFT_442782 | | D8RW16 | | P | | 1293 | | 431 | | | GL377592:444700-447611 | | 7 | | _ | |  |
| SELMODRAFT_442794 | | D8RW85 | | E | | 915 | | 305 | | | GL377592:1322332-1323561 | | 1 | | Chloroplast | |  |
| SELMODRAFT_442798 | | D8RW96 | | P | | 2742 | | 914 | | | GL377592:1510593-1513386 | | 1 | | Chloroplast | |  |
| SELMODRAFT_442983 | | D8RYL4 | | P | | 1068 | | 356 | | | GL377594:136258-138342 | | 5 | | _ | |  |
| SELMODRAFT_44307 | | D8TC85 | | P | | 1023 | | 341 | | | GL377715:105208-106320 | | 0 | | Mitochondria | |  |
| SELMODRAFT_443115 | | D8RYQ1 | | P | | 1218 | | 406 | | | GL377595:94373-95698 | | 1 | | _ | |  |
| SELMODRAFT_443202 | | D8RZG2 | | P | | 744 | | 248 | | | GL377596:108594-110090 | | 5 | | _ | |  |
| SELMODRAFT_443322 | | D8S0G8 | | P | | 1923 | | 641 | | | GL377597:288414-290657 | | 1 | | Mitochondria | |  |
| SELMODRAFT_443679 | | D8S3G3 | | P | | 957 | | 319 | | | GL377600:1323437-1326209 | | 6 | | _ | |  |
| SELMODRAFT_444519 | | D8SAB9 | | P | | 6090 | | 2030 | | | GL377609:1337758-1345163 | | 18 | | Mitochondria | |  |
| SELMODRAFT_444678 | | D8SC06 | | P | | 903 | | 301 | | | GL377611:768327-770190 | | 1 | | _ | |  |
| SELMODRAFT_444770 | | D8SCU8 | | P | | 3414 | | 1138 | | | GL377612:1075837-1079867 | | 3 | | Mitochondria | |  |
| SELMODRAFT_444771 | | D8SCV0 | | P | | 1410 | | 470 | | | GL377612:1082344-1084088 | | 2 | | _ | |  |
| SELMODRAFT_444852 | | D8SDE8 | | P | | 1614 | | 538 | | | GL377613:776880-779231 | | 2 | | _ | |  |
| SELMODRAFT_444869 | | D8SDK8 | | P | | 1167 | | 389 | | | GL377613:1135386-1136886 | | 1 | | _ | |  |
| SELMODRAFT_444974 | | D8SED3 | | DYW | | 1833 | | 611 | | | GL377615:1092921-1095186 | | 1 | | _ | |  |
| SELMODRAFT_445062 | | D8SFB9 | | P | | 1359 | | 453 | | | GL377617:48171-49640 | | 0 | | _ | |  |
| SELMODRAFT_445481 | | D8SIZ1 | | PLS | | 2274 | | 758 | | | GL377622:627812-630713 | | 11 | | Mitochondria | |  |
| SELMODRAFT_445726 | | D8SKR7 | | P | | 930 | | 310 | | | GL377625:480725-482227 | | 2 | | _ | |  |
| SELMODRAFT_445736 | | D8SKU6 | | P | | 1641 | | 547 | | | GL377625:605241-607143 | | 2 | | Mitochondria | |  |
| SELMODRAFT_445856 | | D8SLV0 | | P | | 429 | | 143 | | | GL377626:1199998-1200657 | | 2 | | _ | |  |
| SELMODRAFT_446519 | | D8SS49 | | P | | 1206 | | 402 | | | GL377637:282401-283658 | | 0 | | Mitochondria | |  |
| SELMODRAFT_446579 | | D8SSJ8 | | P | | 1419 | | 473 | | | GL377638:415449-416932 | | 0 | | Chloroplast | |  |
| SELMODRAFT_446777 | | D8SU93 | | P | | 864 | | 288 | | | GL377642:452439-454692 | | 6 | | Mitochondria | |  |
| SELMODRAFT_447 | | D8SG57 | | P | | 2190 | | 730 | | | GL377618:675831-678119 | | 6 | | Mitochondria | |  |
| SELMODRAFT_448320 | | D8T6H1 | | P | | 1683 | | 561 | | | GL377681:177512-179756 | | 6 | | Chloroplast | |  |
| SELMODRAFT_448344 | | D8T6N2 | | P | | 1683 | | 561 | | | GL377682:193581-195806 | | 6 | | _ | |  |
| SELMODRAFT_448545 | | D8T837 | | P | | 1485 | | 495 | | | GL377688:181333-182931 | | 1 | | Chloroplast | |  |
| SELMODRAFT_449117 | | D8TCP3 | | P | | 2244 | | 748 | | | GL377719:68155-70796 | | 5 | | _ | |  |
| SELMODRAFT_449147 | | D8TD41 | | P | | 987 | | 329 | | | GL377726:36148-37596 | | 0 | | _ | |  |
| SELMODRAFT_45004 | | D8SXC6 | | P | | 1032 | | 344 | | | GL377651:120067-122313 | | 2 | | _ | |  |
| SELMODRAFT_450632 | | D8QV22 | | P | | 2421 | | 807 | | | GL377567:2045577-2048479 | | 2 | | Mitochondria | |  |
| SELMODRAFT_450859 | | D8RM74 | | P | | 1446 | | 482 | | | GL377583:1366995-1368538 | | 0 | | SP | |  |
| SELMODRAFT_451 | | D8QQB9 | | P | | 2154 | | 718 | | | GL377565:2411965-2414118 | | 0 | | Mitochondria | |  |
| SELMODRAFT_451140 | | D8RJS8 | | P | | 2235 | | 745 | | | GL377581:2272688-2275338 | | 0 | | Mitochondria | |  |
| SELMODRAFT_451234 | | D8T7P7 | | P | | 1377 | | 459 | | | GL377686:288688-290458 | | 1 | | _ | |  |
| SELMODRAFT_45144 | | D8T2L8 | | P | | 1182 | | 394 | | | GL377666:275786-276967 | | 0 | | _ | |  |
| SELMODRAFT_45153 | | D8SMA8 | | P | | 885 | | 295 | | | GL377627:674440-675972 | | 0 | | _ | |  |
| SELMODRAFT_45637 | | D8SKC4 | | P | | 969 | | 323 | | | GL377624:1101724-1102785 | | 0 | | _ | |  |
| SELMODRAFT_4583 | | D8TGH1 | | PLS | | 1524 | | 508 | | | GL378069:1101-2624 | | 0 | | _ | |  |
| SELMODRAFT_459 | | D8SQN2 | | P | | 1977 | | 659 | | | GL377634:370758-372734 | | 0 | | _ | |  |
| SELMODRAFT_462 | | D8RT88 | | P | | 1977 | | 659 | | | GL377589:832504-834480 | | 0 | | _ | |  |
| SELMODRAFT_465 | | D8S4E1 | | P | | 2040 | | 680 | | | GL377601:1290257-1292296 | | 0 | | _ | |  |
| SELMODRAFT_46757 | | D8SMG5 | | P | | 747 | | 249 | | | GL377627:935365-936111 | | 0 | | _ | |  |
| SELMODRAFT_478 | | D8T7N8 | | P | | 1680 | | 560 | | | GL377686:255427-261982 | | 2 | | _ | |  |
| SELMODRAFT_47850 | | D8T5V6 | | P | | 585 | | 195 | | | GL377678:456605-457353 | | 3 | | SP | |  |
| SELMODRAFT_47998 | | D8TG30 | | P | | 873 | | 291 | | | GL377901:6963-7928 | | 1 | | _ | |  |
| SELMODRAFT_48533 | | D8T1D7 | | P | | 438 | | 146 | | | GL377662:282895-283332 | | 0 | | _ | |  |
| SELMODRAFT_50243 | | D8RND6 | | P | | 978 | | 326 | | | GL377584:2137652-2138824 | | 1 | | _ | |  |
| SELMODRAFT_50342 | | D8S0I4 | | P | | 840 | | 280 | | | GL377597:391332-392798 | | 1 | | _ | |  |
| SELMODRAFT_50634 | | D8S6N2 | | P | | 996 | | 332 | | | GL377604:248460-274236 | | 1 | | _ | |  |
| SELMODRAFT_50691 | | D8RLI2 | | E | | 1539 | | 513 | | | GL377583:81849-92887 | | 2 | | _ | |  |
| SELMODRAFT_50800 | | D8RPI2 | | P | | 819 | | 273 | | | GL377585:1848240-1849673 | | 1 | | _ | |  |
| SELMODRAFT_50864 | | D8RTQ4 | | P | | 2160 | | 720 | | | GL377589:1783690-1785849 | | 0 | | _ | |  |
| SELMODRAFT_50901 | | D8S1S9 | | P | | 699 | | 233 | | | GL377599:534517-535648 | | 2 | | _ | |  |
| SELMODRAFT_50936 | | D8S304 | | P | | 2082 | | 694 | | | GL377600:490049-492742 | | 1 | | _ | |  |
| SELMODRAFT_51146 | | D8S887 | | P | | 1785 | | 595 | | | GL377606:1027348-1030068 | | 2 | | Mitochondria | |  |
| SELMODRAFT_51380 | | D8RMI7 | | P | | 1698 | | 566 | | | GL377584:639203-641176 | | 1 | | _ | |  |
| SELMODRAFT_51482 | | D8S890 | | P | | 1713 | | 571 | | | GL377606:1035528-1037240 | | 0 | | _ | |  |
| SELMODRAFT_51483 | | D8S301 | | P | | 1713 | | 571 | | | GL377600:482807-484519 | | 0 | | _ | |  |
| SELMODRAFT_51561 | | D8S741 | | P | | 1710 | | 570 | | | GL377605:265844-267553 | | 0 | | _ | |  |
| SELMODRAFT_51574 | | D8SBM3 | | P | | 1497 | | 499 | | | GL377610:1282535-1284031 | | 0 | | _ | |  |
| SELMODRAFT_51593 | | D8RY47 | | P | | 1509 | | 503 | | | GL377594:1289660-1291636 | | 3 | | Mitochondria | |  |
| SELMODRAFT_51647 | | D8RNH4 | | P | | 1791 | | 597 | | | GL377585:40700-42490 | | 0 | | _ | |  |
| SELMODRAFT_51678 | | D8RY42 | | P | | 1710 | | 570 | | | GL377594:1273209-1275215 | | 1 | | Mitochondria | |  |
| SELMODRAFT_51693 | | D8S1Z3 | | P | | 1623 | | 541 | | | GL377599:904316-905938 | | 0 | | Mitochondria | |  |
| SELMODRAFT_51832 | | D8SGQ2 | | E | | 1578 | | 526 | | | GL377619:369157-371334 | | 1 | | _ | |  |
| SELMODRAFT_51938 | | D8SGS9 | | P | | 1443 | | 481 | | | GL377619:448658-451381 | | 2 | | Mitochondria | |  |
| SELMODRAFT_52147 | | D8RWK0 | | P | | 1626 | | 542 | | | GL377592:1936894-1939524 | | 3 | | _ | |  |
| SELMODRAFT_52385 | | D8RMW7 | | PLS | | 1350 | | 450 | | | GL377584:1216258-1217607 | | 0 | | _ | |  |
| SELMODRAFT_52548 | | D8RV91 | | P | | 1485 | | 495 | | | GL377591:772434-773918 | | 0 | | _ | |  |
| SELMODRAFT_52561 | | D8S2T6 | | P | | 1257 | | 419 | | | GL377600:89158-90414 | | 0 | | _ | |  |
| SELMODRAFT_52562 | | D8S8E7 | | P | | 1257 | | 419 | | | GL377606:1540595-1541851 | | 0 | | _ | |  |
| SELMODRAFT_52692 | | D8RM18 | | P | | 1461 | | 487 | | | GL377583:1115258-1117269 | | 10 | | _ | |  |
| SELMODRAFT_52932 | | D8RM67 | | P | | 1215 | | 405 | | | GL377583:1329951-1331165 | | 0 | | _ | |  |
| SELMODRAFT_52965 | | D8SCI1 | | P | | 882 | | 294 | | | GL377612:431961-433820 | | 1 | | _ | |  |
| SELMODRAFT_53130 | | D8RXT7 | | P | | 1320 | | 440 | | | GL377594:729842-731455 | | 2 | | Mitochondria | |  |
| SELMODRAFT_53460 | | D8SCU6 | | P | | 1389 | | 463 | | | GL377612:1068451-1069957 | | 1 | | _ | |  |
| SELMODRAFT_53462 | | D8SH06 | | P | | 1389 | | 463 | | | GL377619:878209-879705 | | 1 | | _ | |  |
| SELMODRAFT_53859 | | D8S9P2 | | P | | 819 | | 273 | | | GL377608:978021-978839 | | 0 | | _ | |  |
| SELMODRAFT_541 | | D8SVQ9 | | P | | 1917 | | 639 | | | GL377646:447403-450111 | | 2 | | _ | |  |
| SELMODRAFT_55007 | | D8S3F3 | | P | | 969 | | 323 | | | GL377600:1244696-1245892 | | 1 | | _ | |  |
| SELMODRAFT_55016 | | D8SC68 | | P | | 927 | | 309 | | | GL377611:1184141-1185709 | | 2 | | _ | |  |
| SELMODRAFT_5521 | | D8TCG4 | | P | | 582 | | 194 | | | GL377717:49844-50425 | | 0 | | _ | |  |
| SELMODRAFT_55271 | | D8RL48 | | P | | 933 | | 311 | | | GL377583:1495499-1496431 | | 0 | | _ | |  |
| SELMODRAFT_55272 | | D8S2K9 | | P | | 933 | | 311 | | | GL377599:86349-87281 | | 0 | | _ | |  |
| SELMODRAFT_55637 | | D8SCU1 | | P | | 717 | | 239 | | | GL377612:1026450-1027166 | | 0 | | _ | |  |
| SELMODRAFT_55723 | | D8SET4 | | P | | 948 | | 316 | | | GL377616:259825-260772 | | 0 | | _ | |  |
| SELMODRAFT_55883 | | D8RQV4 | | P | | 801 | | 267 | | | GL377586:684435-685427 | | 1 | | _ | |  |
| SELMODRAFT_55884 | | D8RZX4 | | P | | 801 | | 267 | | | GL377596:1076826-1077818 | | 1 | | _ | |  |
| SELMODRAFT_55945 | | D8RM10 | | P | | 945 | | 315 | | | GL377583:1047790-1049013 | | 1 | | Chloroplast | |  |
| SELMODRAFT_56766 | | D8RYP8 | | P | | 1008 | | 336 | | | GL377595:82651-83907 | | 2 | | _ | |  |
| SELMODRAFT_56840 | | D8RQ10 | | P | | 891 | | 297 | | | GL377586:1313637-1315124 | | 2 | | _ | |  |
| SELMODRAFT_5698 | | D8T264 | | P | | 588 | | 196 | | | GL377665:137850-138437 | | 0 | | _ | |  |
| SELMODRAFT_583 | | D8R1K9 | | P | | 1932 | | 644 | | | GL377570:628231-631260 | | 1 | | Mitochondria | |  |
| SELMODRAFT_58380 | | D8SHB1 | | P | | 348 | | 116 | | | GL377619:1364671-1365018 | | 0 | | _ | |  |
| SELMODRAFT_59387 | | D8RLD0 | | P | | 408 | | 136 | | | GL377583:2081058-2081465 | | 0 | | _ | |  |
| SELMODRAFT_599 | | D8RBV4 | | P | | 1998 | | 666 | | | GL377575:2737661-2741254 | | 2 | | SP | |  |
| SELMODRAFT_60139 | | D8RKX5 | | E | | 1548 | | 516 | | | GL377582:2178238-2183580 | | 3 | | _ | |  |
| SELMODRAFT_60915 | | D8QSJ8 | | PLS | | 1605 | | 535 | | | GL377566:4822319-4823992 | | 1 | | Mitochondria | |  |
| SELMODRAFT_61000 | | D8R6K1 | | P | | 1932 | | 644 | | | GL377572:2368376-2370613 | | 1 | | _ | |  |
| SELMODRAFT_61142 | | D8R794 | | P | | 2016 | | 672 | | | GL377573:748494-750509 | | 0 | | _ | |  |
| SELMODRAFT_61155 | | D8RDW3 | | P | | 1467 | | 489 | | | GL377577:585519-586985 | | 0 | | Mitochondria | |  |
| SELMODRAFT_61162 | | D8RD28 | | P | | 1938 | | 646 | | | GL377576:2217827-2219764 | | 0 | | _ | |  |
| SELMODRAFT_61257 | | D8R5Y7 | | P | | 1890 | | 630 | | | GL377572:1321353-1323674 | | 1 | | Mitochondria | |  |
| SELMODRAFT_61378 | | D8RE82 | | P | | 1761 | | 587 | | | GL377577:1077801-1079795 | | 1 | | _ | |  |
| SELMODRAFT_61490 | | D8R0C5 | | PLS | | 1569 | | 523 | | | GL377569:3330583-3332451 | | 1 | | _ | |  |
| SELMODRAFT_61513 | | D8QWR8 | | P | | 1500 | | 500 | | | GL377568:3418883-3420382 | | 0 | | Mitochondria | |  |
| SELMODRAFT_61534 | | D8RKV3 | | P | | 1515 | | 505 | | | GL377582:1987781-1989295 | | 0 | | Mitochondria | |  |
| SELMODRAFT_61643 | | D8QRZ0 | | P | | 1584 | | 528 | | | GL377566:3538139-3540217 | | 2 | | _ | |  |
| SELMODRAFT_61689 | | D8RJI0 | | P | | 1812 | | 604 | | | GL377581:1766649-1769039 | | 1 | | _ | |  |
| SELMODRAFT_61701 | | D8QZA4 | | P | | 1809 | | 603 | | | GL377569:1228751-1231477 | | 1 | | Mitochondria | |  |
| SELMODRAFT_61782 | | D8QRC2 | | P | | 1710 | | 570 | | | GL377566:2403741-2406029 | | 1 | | _ | |  |
| SELMODRAFT_61795 | | D8QP21 | | P | | 1338 | | 446 | | | GL377565:6637726-6639636 | | 2 | | _ | |  |
| SELMODRAFT_61973 | | D8RCA1 | | P | | 1632 | | 544 | | | GL377576:765580-767211 | | 0 | | Mitochondria | |  |
| SELMODRAFT_62209 | | D8QWA3 | | P | | 1200 | | 400 | | | GL377567:4260515-4261714 | | 0 | | _ | |  |
| SELMODRAFT_62267 | | D8QW16 | | P | | 1485 | | 495 | | | GL377567:3798151-3799635 | | 0 | | _ | |  |
| SELMODRAFT_62313 | | D8QNI2 | | P | | 1419 | | 473 | | | GL377565:5508860-5510461 | | 1 | | _ | |  |
| SELMODRAFT_62477 | | D8R592 | | P | | 1440 | | 480 | | | GL377572:3630245-3632536 | | 2 | | _ | |  |
| SELMODRAFT_626 | | D8QRL3 | | P | | 2025 | | 675 | | | GL377566:2850507-2853007 | | 4 | | _ | |  |
| SELMODRAFT_62636 | | D8RJ86 | | P | | 1305 | | 435 | | | GL377581:1181431-1183302 | | 1 | | _ | |  |
| SELMODRAFT_62785 | | D8RI81 | | P | | 819 | | 273 | | | GL377580:1374196-1375014 | | 0 | | _ | |  |
| SELMODRAFT_63047 | | D8RIY0 | | P | | 1239 | | 413 | | | GL377581:546932-548585 | | 1 | | Mitochondria | |  |
| SELMODRAFT_63315 | | D8RKJ5 | | P | | 948 | | 316 | | | GL377582:1424310-1427179 | | 2 | | _ | |  |
| SELMODRAFT_63558 | | D8R8Y8 | | P | | 1152 | | 384 | | | GL377574:2252708-2254007 | | 2 | | _ | |  |
| SELMODRAFT_63559 | | D8QYP8 | | P | | 1152 | | 384 | | | GL377569:243575-244873 | | 2 | | _ | |  |
| SELMODRAFT_63596 | | D8QRW8 | | P | | 1239 | | 413 | | | GL377566:3429342-3430998 | | 1 | | Mitochondria | |  |
| SELMODRAFT_65645 | | D8QUK9 | | P | | 975 | | 325 | | | GL377567:1166923-1168182 | | 1 | | SP | |  |
| SELMODRAFT_66768 | | D8RIA9 | | P | | 903 | | 301 | | | GL377580:1590268-1591170 | | 0 | | _ | |  |
| SELMODRAFT_67614 | | D8RAF9 | | P | | 483 | | 161 | | | GL377575:95129-95650 | | 1 | | _ | |  |
| SELMODRAFT_67616 | | D8R3V4 | | P | | 483 | | 161 | | | GL377571:2159992-2160512 | | 1 | | _ | |  |
| SELMODRAFT_6905 | | D8TG69 | | P | | 582 | | 194 | | | GL377942:2458-3039 | | 0 | | _ | |  |
| SELMODRAFT_70094 | | D8S1M5 | | P | | 423 | | 141 | | | GL377598:1085840-1087096 | | 1 | | _ | |  |
| SELMODRAFT_70138 | | D8QMY9 | | P | | 2016 | | 672 | | | GL377565:4656467-4658482 | | 0 | | _ | |  |
| SELMODRAFT_70140 | | D8QNM8 | | P | | 1467 | | 489 | | | GL377565:5755266-5756732 | | 0 | | Mitochondria | |  |
| SELMODRAFT_70183 | | D8QPN5 | | DYW | | 1959 | | 653 | | | GL377565:911188-913427 | | 0 | | SP | |  |
| SELMODRAFT_70262 | | D8QQQ9 | | P | | 1437 | | 479 | | | GL377565:3120677-3122113 | | 2 | | Mitochondria | |  |
| SELMODRAFT_70269 | | D8R871 | | P | | 1362 | | 454 | | | GL377573:2427285-2428646 | | 0 | | _ | |  |
| SELMODRAFT_70280 | | D8QU19 | | P | | 1317 | | 439 | | | GL377567:110605-111921 | | 0 | | _ | |  |
| SELMODRAFT_70281 | | D8QV71 | | P | | 1317 | | 439 | | | GL377567:2265759-2267075 | | 0 | | _ | |  |
| SELMODRAFT_70373 | | D8QMK3 | | P | | 1239 | | 413 | | | GL377565:3993542-3995281 | | 1 | | Mitochondria | |  |
| SELMODRAFT_70422 | | D8SFC1 | | P | | 534 | | 178 | | | GL377617:54191-54724 | | 0 | | _ | |  |
| SELMODRAFT_70490 | | D8QX01 | | P | | 528 | | 176 | | | GL377568:3842130-3842657 | | 0 | | _ | |  |
| SELMODRAFT_70595 | | D8QNZ9 | | P | | 954 | | 318 | | | GL377565:6566694-6568721 | | 1 | | _ | |  |
| SELMODRAFT_70599 | | D8RLD4 | | P | | 417 | | 139 | | | GL377583:2094512-2114099 | | 2 | | _ | |  |
| SELMODRAFT_71273 | | D8S3G6 | | P | | 372 | | 124 | | | GL377600:1336697-1337068 | | 0 | | _ | |  |
| SELMODRAFT_71320 | | D8REW9 | | P | | 402 | | 134 | | | GL377577:2842527-2842928 | | 0 | | _ | |  |
| SELMODRAFT_71675 | | D8SV31 | | P | | 504 | | 168 | | | GL377644:566949-569036 | | 1 | | Mitochondria | |  |
| SELMODRAFT_71694 | | D8S017 | | P | | 531 | | 177 | | | GL377596:1304658-1305188 | | 1 | | _ | |  |
| SELMODRAFT_71695 | | D8RQS6 | | P | | 531 | | 177 | | | GL377586:564992-565522 | | 0 | | _ | |  |
| SELMODRAFT_71699 | | D8QQF1 | | P | | 519 | | 173 | | | GL377565:2573797-2574315 | | 0 | | Mitochondria | |  |
| SELMODRAFT_71732 | | D8SQK1 | | P | | 558 | | 186 | | | GL377634:183734-184291 | | 0 | | Mitochondria | |  |
| SELMODRAFT_71738 | | D8RM84 | | P | | 378 | | 126 | | | GL377583:1430778-1431236 | | 1 | | _ | |  |
| SELMODRAFT_71753 | | D8QY15 | | P | | 510 | | 170 | | | GL377568:1927266-1929359 | | 2 | | Mitochondria | |  |
| SELMODRAFT_71754 | | D8SHN2 | | P | | 531 | | 177 | | | GL377620:517101-517940 | | 1 | | _ | |  |
| SELMODRAFT_71764 | | D8RCL8 | | P | | 420 | | 140 | | | GL377576:1334267-1335241 | | 1 | | _ | |  |
| SELMODRAFT_71785 | | D8R1A4 | | P | | 522 | | 174 | | | GL377570:4036748-4038205 | | 1 | | _ | |  |
| SELMODRAFT_71798 | | D8RYQ8 | | E | | 588 | | 196 | | | GL377595:162765-163655 | | 1 | | _ | |  |
| SELMODRAFT_71812 | | D8SUM7 | | P | | 543 | | 181 | | | GL377643:351410-351952 | | 0 | | _ | |  |
| SELMODRAFT_71827 | | D8QQK8 | | P | | 420 | | 140 | | | GL377565:2862534-2862953 | | 0 | | _ | |  |
| SELMODRAFT_71836 | | D8RLC7 | | P | | 441 | | 147 | | | GL377583:2057445-2058778 | | 1 | | _ | |  |
| SELMODRAFT_71839 | | D8QXX2 | | P | | 489 | | 163 | | | GL377568:1549395-1549883 | | 0 | | _ | |  |
| SELMODRAFT_71840 | | D8RUN3 | | P | | 489 | | 163 | | | GL377590:279469-279957 | | 0 | | Mitochondria | |  |
| SELMODRAFT_71859 | | D8SFT7 | | P | | 549 | | 183 | | | GL377617:1031202-1033034 | | 1 | | _ | |  |
| SELMODRAFT_71861 | | D8TE27 | | P | | 522 | | 174 | | | GL377738:61540-62061 | | 0 | | _ | |  |
| SELMODRAFT_71865 | | D8T9I1 | | P | | 525 | | 175 | | | GL377695:328118-329668 | | 1 | | _ | |  |
| SELMODRAFT_71910 | | D8SWH2 | | P | | 522 | | 174 | | | GL377648:528777-529298 | | 0 | | _ | |  |
| SELMODRAFT_71915 | | D8SY46 | | P | | 525 | | 175 | | | GL377652:525703-526542 | | 1 | | _ | |  |
| SELMODRAFT_71918 | | D8RQR4 | | P | | 402 | | 134 | | | GL377586:529861-530352 | | 1 | | _ | |  |
| SELMODRAFT_71935 | | D8RDL8 | | E | | 591 | | 197 | | | GL377576:195072-195662 | | 0 | | _ | |  |
| SELMODRAFT_71936 | | D8RII8 | | P | | 411 | | 137 | | | GL377580:2192905-2193498 | | 1 | | _ | |  |
| SELMODRAFT_71940 | | D8REF9 | | P | | 411 | | 137 | | | GL377577:2004805-2005391 | | 2 | | _ | |  |
| SELMODRAFT_71944 | | D8RWQ2 | | P | | 525 | | 175 | | | GL377593:218289-220493 | | 1 | | Mitochondria | |  |
| SELMODRAFT_71945 | | D8QS12 | | P | | 504 | | 168 | | | GL377566:3635702-3636205 | | 0 | | _ | |  |
| SELMODRAFT_71953 | | D8T7M3 | | P | | 411 | | 137 | | | GL377686:200743-201153 | | 0 | | _ | |  |
| SELMODRAFT_71962 | | D8R914 | | P | | 474 | | 158 | | | GL377574:2355961-2356434 | | 0 | | _ | |  |
| SELMODRAFT_71971 | | D8R7A0 | | P | | 513 | | 171 | | | GL377573:798276-799238 | | 1 | | _ | |  |
| SELMODRAFT_71989 | | D8T8C6 | | E | | 528 | | 176 | | | GL377689:224612-225139 | | 0 | | Mitochondria | |  |
| SELMODRAFT_72000 | | D8TBN4 | | P | | 528 | | 176 | | | GL377711:215392-215919 | | 0 | | Mitochondria | |  |
| SELMODRAFT_72001 | | D8SS50 | | E | | 537 | | 179 | | | GL377637:284410-284946 | | 0 | | _ | |  |
| SELMODRAFT_72012 | | D8R2H3 | | P | | 525 | | 175 | | | GL377570:2293672-2295408 | | 1 | | _ | |  |
| SELMODRAFT_72023 | | D8QT91 | | P | | 525 | | 175 | | | GL377566:6083451-6085187 | | 1 | | _ | |  |
| SELMODRAFT_72028 | | D8R300 | | P | | 525 | | 175 | | | GL377571:368868-369590 | | 1 | | _ | |  |
| SELMODRAFT_72031 | | D8R5C5 | | P | | 414 | | 138 | | | GL377572:3798566-3799351 | | 1 | | _ | |  |
| SELMODRAFT_72055 | | D8RKM7 | | P | | 447 | | 149 | | | GL377582:1603548-1603994 | | 0 | | _ | |  |
| SELMODRAFT_72059 | | D8T9H6 | | P | | 543 | | 181 | | | GL377695:308996-309538 | | 0 | | _ | |  |
| SELMODRAFT_72063 | | D8QTW5 | | P | | 453 | | 151 | | | GL377566:1748923-1749375 | | 0 | | _ | |  |
| SELMODRAFT_72072 | | D8T4N3 | | P | | 531 | | 177 | | | GL377674:380419-380949 | | 0 | | _ | |  |
| SELMODRAFT_72074 | | D8R5C8 | | E | | 528 | | 176 | | | GL377572:3835811-3837541 | | 1 | | _ | |  |
| SELMODRAFT_72097 | | D8R1F9 | | P | | 447 | | 149 | | | GL377570:256539-256985 | | 0 | | _ | |  |
| SELMODRAFT_72101 | | D8R254 | | P | | 456 | | 152 | | | GL377570:1658029-1658484 | | 0 | | _ | |  |
| SELMODRAFT_72112 | | D8R7F2 | | P | | 513 | | 171 | | | GL377573:983243-983755 | | 0 | | _ | |  |
| SELMODRAFT_72131 | | D8R274 | | P | | 531 | | 177 | | | GL377570:1736993-1738456 | | 1 | | SP | |  |
| SELMODRAFT_72132 | | D8S8Y9 | | P | | 522 | | 174 | | | GL377607:1197812-1198456 | | 1 | | _ | |  |
| SELMODRAFT_72136 | | D8QWZ1 | | P | | 528 | | 176 | | | GL377568:3789416-3791089 | | 1 | | Mitochondria | |  |
| SELMODRAFT_72146 | | D8RM14 | | P | | 513 | | 171 | | | GL377583:1109325-1109837 | | 0 | | _ | |  |
| SELMODRAFT_72148 | | D8SM25 | | P | | 534 | | 178 | | | GL377627:217402-217935 | | 0 | | _ | |  |
| SELMODRAFT_72165 | | D8S4K6 | | P | | 528 | | 176 | | | GL377601:1561497-1562024 | | 0 | | _ | |  |
| SELMODRAFT_72177 | | D8SFT2 | | P | | 543 | | 181 | | | GL377617:1012714-1013256 | | 0 | | _ | |  |
| SELMODRAFT_72179 | | D8RJ19 | | P | | 522 | | 174 | | | GL377581:798909-799430 | | 0 | | _ | |  |
| SELMODRAFT_72195 | | D8QMY1 | | P | | 513 | | 171 | | | GL377565:4613485-4614447 | | 1 | | _ | |  |
| SELMODRAFT_72200 | | D8R5V4 | | P | | 489 | | 163 | | | GL377572:1150816-1153167 | | 1 | | _ | |  |
| SELMODRAFT_72201 | | D8SG27 | | P | | 501 | | 167 | | | GL377618:516749-519112 | | 1 | | _ | |  |
| SELMODRAFT_72206 | | D8T8T0 | | P | | 318 | | 106 | | | GL377691:311951-312438 | | 1 | | _ | |  |
| SELMODRAFT_72211 | | D8RT80 | | P | | 525 | | 175 | | | GL377634:336374-337726 | | 1 | | _ | |  |
| SELMODRAFT_72234 | | D8R0K3 | | P | | 516 | | 172 | | | GL377569:3723183-3724646 | | 1 | | _ | |  |
| SELMODRAFT_72239 | | D8S514 | | P | | 528 | | 176 | | | GL377602:696496-697023 | | 0 | | _ | |  |
| SELMODRAFT_72250 | | D8SKV1 | | P | | 429 | | 143 | | | GL377625:619043-619644 | | 1 | | _ | |  |
| SELMODRAFT_72257 | | D8RHK0 | | P | | 531 | | 177 | | | GL377579:114293-114823 | | 0 | | _ | |  |
| SELMODRAFT_72269 | | D8RP29 | | P | | 519 | | 173 | | | GL377585:1007059-1007577 | | 0 | | _ | |  |
| SELMODRAFT_72271 | | D8T214 | | P | | 552 | | 184 | | | GL377664:479552-480103 | | 0 | | _ | |  |
| SELMODRAFT_72276 | | D8SL03 | | P | | 528 | | 176 | | | GL377625:881084-881611 | | 0 | | Mitochondria | |  |
| SELMODRAFT_72285 | | D8QUU5 | | P | | 336 | | 112 | | | GL377567:1627256-1627591 | | 0 | | _ | |  |
| SELMODRAFT_72286 | | D8RPE0 | | P | | 438 | | 146 | | | GL377627:379142-379579 | | 0 | | Mitochondria | |  |
| SELMODRAFT_72294 | | D8T2G0 | | P | | 525 | | 175 | | | GL377665:598462-598986 | | 0 | | _ | |  |
| SELMODRAFT_72295 | | D8SKR0 | | P | | 507 | | 169 | | | GL377625:449709-450770 | | 1 | | Mitochondria | |  |
| SELMODRAFT_72299 | | D8R423 | | P | | 345 | | 115 | | | GL377571:2489366-2489710 | | 0 | | _ | |  |
| SELMODRAFT_72303 | | D8QPR7 | | P | | 507 | | 169 | | | GL377565:1089227-1090288 | | 1 | | _ | |  |
| SELMODRAFT_72308 | | D8R7W1 | | P | | 543 | | 181 | | | GL377573:1952557-1953099 | | 0 | | Mitochondria | |  |
| SELMODRAFT_72314 | | D8S7C9 | | P | | 432 | | 144 | | | GL377605:730427-730858 | | 0 | | _ | |  |
| SELMODRAFT_72317 | | D8SZ55 | | P | | 432 | | 144 | | | GL377654:695367-695798 | | 0 | | _ | |  |
| SELMODRAFT_72322 | | D8QS93 | | P | | 447 | | 149 | | | GL377566:4254132-4254578 | | 0 | | _ | |  |
| SELMODRAFT_72328 | | D8R2I4 | | P | | 513 | | 171 | | | GL377570:2330886-2332427 | | 1 | | _ | |  |
| SELMODRAFT_72345 | | D8SFQ9 | | P | | 420 | | 140 | | | GL377617:891219-893120 | | 1 | | SP | |  |
| SELMODRAFT_72357 | | D8S4K9 | | P | | 411 | | 137 | | | GL377601:1574908-1575318 | | 0 | | _ | |  |
| SELMODRAFT_72358 | | D8QZ92 | | P | | 525 | | 175 | | | GL377569:1169057-1170688 | | 1 | | _ | |  |
| SELMODRAFT_72359 | | D8QMJ8 | | P | | 420 | | 140 | | | GL377573:1550350-1550769 | | 0 | | _ | |  |
| SELMODRAFT_72363 | | D8R6E5 | | P | | 411 | | 137 | | | GL377572:2117196-2117606 | | 0 | | _ | |  |
| SELMODRAFT_72364 | | D8T097 | | P | | 528 | | 176 | | | GL377658:20335-20862 | | 0 | | _ | |  |
| SELMODRAFT_72365 | | D8RXW9 | | P | | 519 | | 173 | | | GL377594:899103-899621 | | 0 | | Mitochondria | |  |
| SELMODRAFT_72369 | | D8SGV3 | | P | | 414 | | 138 | | | GL377619:553747-554705 | | 1 | | _ | |  |
| SELMODRAFT_72370 | | D8T7R6 | | P | | 420 | | 140 | | | GL377686:388053-388472 | | 0 | | Mitochondria | |  |
| SELMODRAFT_72424 | | D8SEJ7 | | P | | 396 | | 132 | | | GL377615:74501-75838 | | 1 | | _ | |  |
| SELMODRAFT_72427 | | D8RHZ7 | | P | | 369 | | 123 | | | GL377580:819189-819725 | | 1 | | _ | |  |
| SELMODRAFT_72439 | | D8T6H5 | | P | | 450 | | 150 | | | GL377681:188305-189831 | | 1 | | _ | |  |
| SELMODRAFT_72464 | | D8RE53 | | P | | 417 | | 139 | | | GL377577:978006-979721 | | 1 | | Mitochondria | |  |
| SELMODRAFT_72475 | | D8T9K6 | | P | | 225 | | 75 | | | GL377696:303976-304200 | | 0 | | _ | |  |
| SELMODRAFT_72476 | | D8RHD8 | | P | | 411 | | 137 | | | GL377579:2579367-2580050 | | 1 | | _ | |  |
| SELMODRAFT_72487 | | D8T880 | | P | | 414 | | 138 | | | GL377688:367634-368224 | | 1 | | _ | |  |
| SELMODRAFT_72489 | | D8T265 | | P | | 414 | | 138 | | | GL377665:139736-140326 | | 1 | | _ | |  |
| SELMODRAFT_72499 | | D8SCY1 | | P | | 423 | | 141 | | | GL377612:1190531-1191796 | | 1 | | _ | |  |
| SELMODRAFT_72536 | | D8SDT3 | | P | | 162 | | 54 | | | GL377614:484619-484780 | | 0 | | SP | |  |
| SELMODRAFT_72718 | | D8QSG8 | | P | | 297 | | 99 | | | GL377566:4665083-4665379 | | 0 | | _ | |  |
| SELMODRAFT_72770 | | D8SP28 | | P | | 360 | | 120 | | | GL377630:966730-967089 | | 0 | | _ | |  |
| SELMODRAFT_73325 | | D8QNX7 | | P | | 321 | | 107 | | | GL377565:6425783-6426103 | | 0 | | _ | |  |
| SELMODRAFT_73342 | | D8QNH1 | | P | | 1734 | | 578 | | | GL377565:5437230-5439521 | | 1 | | Mitochondria | |  |
| SELMODRAFT_73361 | | D8QPL3 | | P | | 1134 | | 378 | | | GL377565:711704-713032 | | 1 | | _ | |  |
| SELMODRAFT_73371 | | D8QN58 | | P | | 561 | | 187 | | | GL377565:4904652-4905863 | | 1 | | SP | |  |
| SELMODRAFT_73601 | | D8QR65 | | P | | 1731 | | 577 | | | GL377565:3740705-3746626 | | 1 | | _ | |  |
| SELMODRAFT_73641 | | D8QP85 | | P | | 1386 | | 462 | | | GL377565:6916345-6918945 | | 2 | | _ | |  |
| SELMODRAFT_73779 | | D8QNG0 | | P | | 750 | | 250 | | | GL377565:5367246-5367998 | | 0 | | Mitochondria | |  |
| SELMODRAFT_73817 | | D8QN33 | | P | | 1170 | | 390 | | | GL377565:4804184-4805794 | | 2 | | _ | |  |
| SELMODRAFT_73828 | | D8QQN8 | | P | | 1638 | | 546 | | | GL377565:3023602-3025242 | | 0 | | _ | |  |
| SELMODRAFT_73919 | | D8QN37 | | P | | 906 | | 302 | | | GL377565:4848951-4849859 | | 0 | | _ | |  |
| SELMODRAFT_74141 | | D8QNF8 | | P | | 735 | | 245 | | | GL377565:5364625-5365359 | | 0 | | _ | |  |
| SELMODRAFT_74281 | | D8QPN3 | | P | | 615 | | 205 | | | GL377565:878623-879240 | | 0 | | _ | |  |
| SELMODRAFT_74388 | | D8QNU1 | | DYW | | 1455 | | 485 | | | GL377565:6157646-6160027 | | 1 | | _ | |  |
| SELMODRAFT_74408 | | D8QMN4 | | P | | 963 | | 321 | | | GL377565:4154754-4155719 | | 0 | | SP | |  |
| SELMODRAFT_74454 | | D8QP66 | | P | | 1152 | | 384 | | | GL377565:6845598-6852352 | | 1 | | _ | |  |
| SELMODRAFT_74552 | | D8QPM7 | | P | | 270 | | 90 | | | GL377565:859560-859832 | | 0 | | Mitochondria | |  |
| SELMODRAFT_74577 | | D8QPK7 | | P | | 738 | | 246 | | | GL377565:688397-689134 | | 0 | | Mitochondria | |  |
| SELMODRAFT_74600 | | D8QPH9 | | P | | 759 | | 253 | | | GL377565:583649-584410 | | 0 | | _ | |  |
| SELMODRAFT_74627 | | D8QQ08 | | P | | 2628 | | 876 | | | GL377565:1606325-1608955 | | 0 | | Mitochondria | |  |
| SELMODRAFT_74706 | | D8QMI8 | | P | | 933 | | 311 | | | GL377565:3901883-3903016 | | 1 | | Mitochondria | |  |
| SELMODRAFT_74762 | | D8QQK3 | | P | | 1929 | | 643 | | | GL377565:2852049-2853980 | | 0 | | Mitochondria | |  |
| SELMODRAFT_74842 | | D8QQC7 | | DYW | | 2709 | | 903 | | | GL377565:2445121-2447832 | | 0 | | _ | |  |
| SELMODRAFT_75322 | | D8QPA4 | | E | | 1551 | | 517 | | | GL377565:239110-240936 | | 2 | | _ | |  |
| SELMODRAFT_75412 | | D8QQB8 | | P | | 690 | | 230 | | | GL377565:2409343-2410032 | | 0 | | _ | |  |
| SELMODRAFT_75478 | | D8QPS9 | | DYW | | 1443 | | 481 | | | GL377565:1125880-1127325 | | 0 | | _ | |  |
| SELMODRAFT_75566 | | D8QQV1 | | P | | 1773 | | 591 | | | GL377565:3281889-3284522 | | 1 | | _ | |  |
| SELMODRAFT_75584 | | D8QPG0 | | P | | 657 | | 219 | | | GL377565:516130-516786 | | 0 | | _ | |  |
| SELMODRAFT_75621 | | D8QMN5 | | PLS | | 1884 | | 628 | | | GL377565:4155716-4158882 | | 2 | | _ | |  |
| SELMODRAFT_75631 | | D8QQ44 | | PLS | | 2097 | | 699 | | | GL377565:1853225-1855324 | | 0 | | _ | |  |
| SELMODRAFT_75649 | | D8QMN0 | | P | | 1383 | | 461 | | | GL377565:4117483-4119276 | | 1 | | Mitochondria | |  |
| SELMODRAFT_75659 | | D8QPN1 | | P | | 552 | | 184 | | | GL377565:870123-870677 | | 0 | | _ | |  |
| SELMODRAFT_75909 | | D8QPA3 | | E | | 468 | | 156 | | | GL377565:228488-228958 | | 0 | | _ | |  |
| SELMODRAFT_75930 | | D8QSY9 | | P | | 294 | | 98 | | | GL377566:5495282-5496481 | | 1 | | _ | |  |
| SELMODRAFT_76034 | | D8QRS4 | | P | | 1395 | | 465 | | | GL377566:3219014-3220507 | | 1 | | _ | |  |
| SELMODRAFT_76039 | | D8QS53 | | E | | 1464 | | 488 | | | GL377566:3950362-3955108 | | 1 | | _ | |  |
| SELMODRAFT_76135 | | D8QSY0 | | P | | 573 | | 191 | | | GL377566:5437795-5438370 | | 0 | | Mitochondria | |  |
| SELMODRAFT_76175 | | D8QTC0 | | DYW | | 3363 | | 1121 | | | GL377566:181438-194024 | | 2 | | Mitochondria | |  |
| SELMODRAFT_76220 | | D8QSF0 | | P | | 1452 | | 484 | | | GL377566:4515323-4516855 | | 1 | | Mitochondria | |  |
| SELMODRAFT_76226 | | D8QRU8 | | P | | 2541 | | 847 | | | GL377566:3309069-3313115 | | 4 | | _ | |  |
| SELMODRAFT_76236 | | D8QT94 | | P | | 465 | | 155 | | | GL377566:6091968-6092435 | | 0 | | SP | |  |
| SELMODRAFT_76510 | | D8QS06 | | P | | 1809 | | 603 | | | GL377566:3609843-3611654 | | 0 | | _ | |  |
| SELMODRAFT_76597 | | D8QSJ1 | | P | | 3168 | | 1056 | | | GL377566:4764082-4769011 | | 2 | | Mitochondria | |  |
| SELMODRAFT_76772 | | D8QSE6 | | P | | 552 | | 184 | | | GL377566:4497489-4498043 | | 0 | | _ | |  |
| SELMODRAFT_76818 | | D8QTV0 | | P | | 672 | | 224 | | | GL377566:1667907-1669505 | | 1 | | _ | |  |
| SELMODRAFT_76916 | | D8QRX1 | | P | | 1479 | | 493 | | | GL377566:3437566-3439314 | | 1 | | _ | |  |
| SELMODRAFT_76934 | | D8QSJ4 | | P | | 2565 | | 855 | | | GL377566:4800965-4807845 | | 1 | | _ | |  |
| SELMODRAFT_76943 | | D8QRW0 | | P | | 1095 | | 365 | | | GL377566:3403200-3404822 | | 1 | | _ | |  |
| SELMODRAFT_76944 | | D8QRD6 | | P | | 774 | | 258 | | | GL377566:2481792-2483003 | | 2 | | _ | |  |
| SELMODRAFT_77006 | | D8QTY8 | | P | | 1500 | | 500 | | | GL377566:2073553-2075055 | | 0 | | _ | |  |
| SELMODRAFT_77068 | | D8QTF7 | | P | | 687 | | 229 | | | GL377566:548083-548769 | | 1 | | _ | |  |
| SELMODRAFT_77146 | | D8QT92 | | P | | 573 | | 191 | | | GL377566:6087260-6087931 | | 1 | | _ | |  |
| SELMODRAFT_77219 | | D8QRG4 | | P | | 801 | | 267 | | | GL377566:2604406-2605680 | | 1 | | Mitochondria | |  |
| SELMODRAFT_77254 | | D8QS50 | | P | | 1212 | | 404 | | | GL377566:3940043-3941731 | | 1 | | _ | |  |
| SELMODRAFT_77340 | | D8QRX4 | | P | | 960 | | 320 | | | GL377566:3472525-3474345 | | 1 | | Mitochondria | |  |
| SELMODRAFT_77345 | | D8QSJ3 | | P | | 1326 | | 442 | | | GL377566:4781996-4783324 | | 0 | | SP | |  |
| SELMODRAFT_77396 | | D8QRU5 | | P | | 567 | | 189 | | | GL377566:3297872-3299275 | | 1 | | _ | |  |
| SELMODRAFT_77426 | | D8QTZ8 | | E | | 1929 | | 643 | | | GL377566:2093959-2097051 | | 3 | | _ | |  |
| SELMODRAFT_77588 | | D8QRP1 | | P | | 2442 | | 814 | | | GL377566:3055427-3057868 | | 0 | | Mitochondria | |  |
| SELMODRAFT_77659 | | D8QSQ6 | | P | | 1437 | | 479 | | | GL377566:5120365-5121804 | | 0 | | _ | |  |
| SELMODRAFT_77736 | | D8QSG9 | | P | | 2193 | | 731 | | | GL377566:4665524-4667716 | | 0 | | Chloroplast | |  |
| SELMODRAFT_77859 | | D8QVZ6 | | E | | 984 | | 328 | | | GL377567:3705765-3707453 | | 2 | | _ | |  |
| SELMODRAFT_77892 | | D8QUF8 | | P | | 651 | | 217 | | | GL377567:905927-907842 | | 1 | | _ | |  |
| SELMODRAFT_78040 | | D8QUG8 | | P | | 660 | | 220 | | | GL377567:935925-936587 | | 0 | | Mitochondria | |  |
| SELMODRAFT_78092 | | D8QWC4 | | DYW | | 2805 | | 935 | | | GL377567:4317545-4320352 | | 0 | | Mitochondria | |  |
| SELMODRAFT_78126 | | D8QUH2 | | P | | 828 | | 276 | | | GL377567:954401-955399 | | 1 | | _ | |  |
| SELMODRAFT_78269 | | D8QVL1 | | P | | 795 | | 265 | | | GL377567:2952492-2953344 | | 1 | | _ | |  |
| SELMODRAFT_78295 | | D8QUS7 | | P | | 384 | | 128 | | | GL377567:1477509-1478102 | | 1 | | _ | |  |
| SELMODRAFT_78378 | | D8QW40 | | DYW | | 1626 | | 542 | | | GL377567:3883796-3885640 | | 1 | | _ | |  |
| SELMODRAFT_78491 | | D8QUR6 | | P | | 984 | | 328 | | | GL377567:1411964-1413688 | | 1 | | _ | |  |
| SELMODRAFT_78548 | | D8QW61 | | P | | 672 | | 224 | | | GL377567:3952378-3957665 | | 1 | | _ | |  |
| SELMODRAFT_78564 | | D8QW77 | | P | | 675 | | 225 | | | GL377567:4115368-4116042 | | 0 | | Mitochondria | |  |
| SELMODRAFT_78778 | | D8QUH3 | | P | | 657 | | 219 | | | GL377567:955394-956329 | | 1 | | SP | |  |
| SELMODRAFT_78994 | | D8QUW3 | | P | | 486 | | 162 | | | GL377567:1689226-1689714 | | 0 | | SP | |  |
| SELMODRAFT_79052 | | D8QUJ0 | | DYW | | 1956 | | 652 | | | GL377567:1075827-1077785 | | 0 | | _ | |  |
| SELMODRAFT_79165 | | D8QU21 | | P | | 579 | | 193 | | | GL377567:237168-238082 | | 3 | | SP | |  |
| SELMODRAFT_79341 | | D8QWK5 | | P | | 759 | | 253 | | | GL377568:3108269-3109213 | | 1 | | _ | |  |
| SELMODRAFT_79356 | | D8QWT8 | | E | | 1605 | | 535 | | | GL377568:3554915-3559577 | | 2 | | Mitochondria | |  |
| SELMODRAFT_79421 | | D8QWT9 | | P | | 1848 | | 616 | | | GL377568:3571662-3573512 | | 0 | | _ | |  |
| SELMODRAFT_79447 | | D8QY41 | | P | | 669 | | 223 | | | GL377568:2010180-2011328 | | 1 | | _ | |  |
| SELMODRAFT_79457 | | D8QWJ4 | | P | | 687 | | 229 | | | GL377568:3069295-3070284 | | 1 | | _ | |  |
| SELMODRAFT_79513 | | D8QXL6 | | P | | 1719 | | 573 | | | GL377568:864747-866672 | | 2 | | _ | |  |
| SELMODRAFT_79606 | | D8QXS0 | | P | | 726 | | 242 | | | GL377568:1310423-1311927 | | 2 | | SP | |  |
| SELMODRAFT_79648 | | D8QY04 | | P | | 1740 | | 580 | | | GL377568:1784126-1806503 | | 2 | | _ | |  |
| SELMODRAFT_79652 | | D8QYE0 | | P | | 1128 | | 376 | | | GL377568:2505818-2506948 | | 0 | | _ | |  |
| SELMODRAFT_79700 | | D8QWW2 | | P | | 1725 | | 575 | | | GL377568:3641262-3643067 | | 1 | | Mitochondria | |  |
| SELMODRAFT_79732 | | D8QX23 | | DYW | | 2487 | | 829 | | | GL377568:3924967-3927456 | | 0 | | Mitochondria | |  |
| SELMODRAFT_79750 | | D8QWS4 | | DYW | | 1983 | | 661 | | | GL377568:3441842-3444013 | | 1 | | _ | |  |
| SELMODRAFT_79771 | | D8QY17 | | P | | 1485 | | 495 | | | GL377568:1932011-1933498 | | 0 | | _ | |  |
| SELMODRAFT_79843 | | D8QWL4 | | P | | 1296 | | 432 | | | GL377568:3127241-3128539 | | 0 | | Mitochondria | |  |
| SELMODRAFT_79856 | | D8QXT2 | | P | | 570 | | 190 | | | GL377568:1392318-1392890 | | 0 | | _ | |  |
| SELMODRAFT_79889 | | D8QXK3 | | P | | 1923 | | 641 | | | GL377568:794389-796314 | | 0 | | _ | |  |
| SELMODRAFT_79925 | | D8QWK2 | | P | | 1311 | | 437 | | | GL377568:3100758-3102719 | | 1 | | Chloroplast | |  |
| SELMODRAFT_80086 | | D8QX80 | | DYW | | 2142 | | 714 | | | GL377568:4132670-4134814 | | 0 | | SP | |  |
| SELMODRAFT_80179 | | D8QWS0 | | P | | 819 | | 273 | | | GL377568:3430651-3431472 | | 0 | | _ | |  |
| SELMODRAFT_80280 | | D8QXP3 | | P | | 1050 | | 350 | | | GL377568:1142074-1146100 | | 2 | | _ | |  |
| SELMODRAFT_80295 | | D8QXD5 | | P | | 1188 | | 396 | | | GL377568:390844-392250 | | 1 | | SP | |  |
| SELMODRAFT_80298 | | D8QY09 | | P | | 1791 | | 597 | | | GL377568:1905050-1907188 | | 1 | | _ | |  |
| SELMODRAFT_80365 | | D8QYA8 | | P | | 975 | | 325 | | | GL377568:2391824-2392801 | | 0 | | Chloroplast | |  |
| SELMODRAFT_80412 | | D8QWJ6 | | P | | 2580 | | 860 | | | GL377568:3072621-3075494 | | 1 | | _ | |  |
| SELMODRAFT_80457 | | D8QXK1 | | P | | 1041 | | 347 | | | GL377568:791707-793017 | | 1 | | _ | |  |
| SELMODRAFT_80491 | | D8QXL9 | | P | | 1884 | | 628 | | | GL377568:931679-933868 | | 1 | | Mitochondria | |  |
| SELMODRAFT_80650 | | D8QYG4 | | P | | 642 | | 214 | | | GL377568:2635532-2636176 | | 0 | | _ | |  |
| SELMODRAFT_80662 | | D8QWX0 | | DYW | | 2343 | | 781 | | | GL377568:3710250-3712595 | | 0 | | _ | |  |
| SELMODRAFT_80847 | | D8QX00 | | E | | 1440 | | 480 | | | GL377568:3825863-3829238 | | 2 | | _ | |  |
| SELMODRAFT_80859 | | D8QXN7 | | P | | 717 | | 239 | | | GL377568:1033841-1076385 | | 2 | | _ | |  |
| SELMODRAFT_80903 | | D8QZX2 | | P | | 621 | | 207 | | | GL377569:2200498-2201121 | | 0 | | _ | |  |
| SELMODRAFT_80975 | | D8R0N3 | | P | | 993 | | 331 | | | GL377569:3854279-3855274 | | 0 | | _ | |  |
| SELMODRAFT_81012 | | D8QZX1 | | P | | 690 | | 230 | | | GL377569:2199505-2200284 | | 0 | | Mitochondria | |  |
| SELMODRAFT_81078 | | D8R0C0 | | P | | 1818 | | 606 | | | GL377569:3275895-3278165 | | 2 | | _ | |  |
| SELMODRAFT_81196 | | D8R0D3 | | DYW | | 2208 | | 736 | | | GL377569:3490502-3493309 | | 1 | | SP | |  |
| SELMODRAFT_81279 | | D8QYV8 | | E | | 1920 | | 640 | | | GL377569:470875-473532 | | 1 | | Mitochondria | |  |
| SELMODRAFT_81376 | | D8R040 | | P | | 792 | | 264 | | | GL377569:2671094-2671888 | | 0 | | _ | |  |
| SELMODRAFT_81428 | | D8R052 | | P | | 921 | | 307 | | | GL377569:2709766-2710689 | | 0 | | _ | |  |
| SELMODRAFT_81513 | | D8QZG1 | | P | | 963 | | 321 | | | GL377569:1497074-1498039 | | 0 | | _ | |  |
| SELMODRAFT_81625 | | D8QZE5 | | P | | 1422 | | 474 | | | GL377569:1397470-1399197 | | 1 | | _ | |  |
| SELMODRAFT_81698 | | D8QZ76 | | E | | 1512 | | 504 | | | GL377569:1016304-1017917 | | 0 | | SP | |  |
| SELMODRAFT_81711 | | D8QYY7 | | P | | 1074 | | 358 | | | GL377569:550672-552597 | | 1 | | Mitochondria | |  |
| SELMODRAFT_81759 | | D8R0C7 | | P | | 1740 | | 580 | | | GL377569:3335831-3337933 | | 2 | | Mitochondria | |  |
| SELMODRAFT_81828 | | D8R076 | | P | | 1560 | | 520 | | | GL377569:2884834-2886564 | | 1 | | _ | |  |
| SELMODRAFT_81964 | | D8QZ69 | | E | | 711 | | 237 | | | GL377569:993420-994133 | | 0 | | _ | |  |
| SELMODRAFT_82002 | | D8QYW4 | | PLS | | 2148 | | 716 | | | GL377569:486570-488978 | | 1 | | Mitochondria | |  |
| SELMODRAFT_82011 | | D8QZ54 | | P | | 1128 | | 376 | | | GL377569:914863-916599 | | 1 | | _ | |  |
| SELMODRAFT_82024 | | D8QZP0 | | DYW | | 2139 | | 713 | | | GL377569:1818125-1820266 | | 0 | | Mitochondria | |  |
| SELMODRAFT_82035 | | D8QZB2 | | P | | 1470 | | 490 | | | GL377569:1264427-1268071 | | 1 | | Mitochondria | |  |
| SELMODRAFT_82058 | | D8QYS5 | | P | | 762 | | 254 | | | GL377569:344057-344821 | | 0 | | _ | |  |
| SELMODRAFT_82072 | | D8R0C1 | | DYW | | 2385 | | 795 | | | GL377569:3278208-3280595 | | 0 | | Mitochondria | |  |
| SELMODRAFT_82101 | | D8QZ47 | | P | | 1413 | | 471 | | | GL377569:870064-871479 | | 0 | | Chloroplast | |  |
| SELMODRAFT_82221 | | D8QZY0 | | P | | 1584 | | 528 | | | GL377569:2235106-2236692 | | 0 | | Mitochondria | |  |
| SELMODRAFT_82255 | | D8QZ74 | | DYW | | 1296 | | 432 | | | GL377569:1009536-1010834 | | 0 | | _ | |  |
| SELMODRAFT_82268 | | D8R0H7 | | P | | 2220 | | 740 | | | GL377569:3634700-3637234 | | 1 | | Mitochondria | |  |
| SELMODRAFT_82351 | | D8QYP7 | | P | | 1128 | | 376 | | | GL377569:242198-243328 | | 0 | | _ | |  |
| SELMODRAFT_82637 | | D8R2H6 | | P | | 558 | | 186 | | | GL377570:2302186-2302746 | | 0 | | _ | |  |
| SELMODRAFT_82640 | | D8R2T5 | | P | | 1113 | | 371 | | | GL377570:2867339-2868787 | | 2 | | _ | |  |
| SELMODRAFT_82647 | | D8R186 | | P | | 1128 | | 376 | | | GL377570:3945759-3947960 | | 3 | | _ | |  |
| SELMODRAFT_82803 | | D8R1L6 | | P | | 855 | | 285 | | | GL377570:654521-666925 | | 2 | | _ | |  |
| SELMODRAFT_82857 | | D8R1Q7 | | P | | 1656 | | 552 | | | GL377570:929050-935201 | | 0 | | _ | |  |
| SELMODRAFT_82859 | | D8R0Y7 | | E | | 2418 | | 806 | | | GL377570:3376434-3378854 | | 0 | | Mitochondria | |  |
| SELMODRAFT_82937 | | D8R1R1 | | P | | 1620 | | 540 | | | GL377570:996105-997727 | | 1 | | SP | |  |
| SELMODRAFT_83034 | | D8R0U1 | | DYW | | 1800 | | 600 | | | GL377570:2982854-2985313 | | 0 | | _ | |  |
| SELMODRAFT_83058 | | D8R1L3 | | P | | 546 | | 182 | | | GL377570:642685-643233 | | 0 | | _ | |  |
| SELMODRAFT_83088 | | D8R1N5 | | P | | 2193 | | 731 | | | GL377570:850734-852926 | | 1 | | _ | |  |
| SELMODRAFT_83127 | | D8R1L8 | | PLS | | 1452 | | 484 | | | GL377570:684056-685588 | | 1 | | Mitochondria | |  |
| SELMODRAFT_83272 | | D8R2Q8 | | P | | 1335 | | 445 | | | GL377570:2750379-2751716 | | 1 | | _ | |  |
| SELMODRAFT_83321 | | D8R1R2 | | P | | 1800 | | 600 | | | GL377570:1014938-1021691 | | 1 | | _ | |  |
| SELMODRAFT_83339 | | D8R2S2 | | DYW | | 2163 | | 721 | | | GL377570:2800308-2803079 | | 0 | | Mitochondria | |  |
| SELMODRAFT_83491 | | D8R2H4 | | P | | 669 | | 223 | | | GL377570:2297481-2298152 | | 2 | | _ | |  |
| SELMODRAFT_83553 | | D8R0Z2 | | P | | 1032 | | 344 | | | GL377570:3391669-3393009 | | 0 | | SP | |  |
| SELMODRAFT_83579 | | D8R0Z6 | | P | | 414 | | 138 | | | GL377570:3405954-3406370 | | 0 | | Mitochondria | |  |
| SELMODRAFT_83698 | | D8R2S5 | | P | | 921 | | 307 | | | GL377570:2807477-2808793 | | 1 | | _ | |  |
| SELMODRAFT_83796 | | D8R1R7 | | PLS | | 1809 | | 603 | | | GL377570:1035001-1037007 | | 1 | | Mitochondria | |  |
| SELMODRAFT_83883 | | D8R4Q3 | | P | | 651 | | 217 | | | GL377571:3799498-3800441 | | 0 | | _ | |  |
| SELMODRAFT_83991 | | D8R315 | | P | | 1320 | | 440 | | | GL377571:437615-438937 | | 1 | | _ | |  |
| SELMODRAFT_84002 | | D8R4T4 | | P | | 1173 | | 391 | | | GL377571:3937146-3950004 | | 1 | | _ | |  |
| SELMODRAFT_84012 | | D8R3Z4 | | P | | 1599 | | 533 | | | GL377571:2366744-2368666 | | 6 | | Chloroplast | |  |
| SELMODRAFT_84108 | | D8R442 | | P | | 744 | | 248 | | | GL377571:2554685-2555827 | | 1 | | Mitochondria | |  |
| SELMODRAFT_84160 | | D8R4L3 | | P | | 1125 | | 375 | | | GL377571:3620898-3622118 | | 1 | | _ | |  |
| SELMODRAFT_84288 | | D8R4P8 | | P | | 1812 | | 604 | | | GL377571:3729620-3733589 | | 3 | | _ | |  |
| SELMODRAFT_84339 | | D8R2Y3 | | P | | 1485 | | 495 | | | GL377571:225600-227984 | | 1 | | _ | |  |
| SELMODRAFT_84442 | | D8R4Q5 | | P | | 651 | | 217 | | | GL377571:3817286-3818229 | | 1 | | _ | |  |
| SELMODRAFT_84506 | | D8R362 | | P | | 1056 | | 352 | | | GL377571:771217-772362 | | 1 | | Mitochondria | |  |
| SELMODRAFT_84571 | | D8R425 | | P | | 1482 | | 494 | | | GL377571:2495990-2497765 | | 1 | | _ | |  |
| SELMODRAFT_84620 | | D8R4R3 | | DYW | | 1794 | | 598 | | | GL377571:3877878-3880247 | | 1 | | _ | |  |
| SELMODRAFT_84628 | | D8R3H5 | | P | | 1596 | | 532 | | | GL377571:1314512-1316528 | | 6 | | _ | |  |
| SELMODRAFT_84657 | | D8R4Q7 | | DYW | | 1506 | | 502 | | | GL377571:3826775-3828783 | | 2 | | _ | |  |
| SELMODRAFT_84672 | | D8R4A5 | | P | | 1239 | | 413 | | | GL377571:2931416-2933587 | | 1 | | _ | |  |
| SELMODRAFT_84768 | | D8R4P5 | | DYW | | 1347 | | 449 | | | GL377571:3717191-3718540 | | 0 | | _ | |  |
| SELMODRAFT_85072 | | D8R3P2 | | P | | 954 | | 318 | | | GL377571:1744048-1745001 | | 0 | | _ | |  |
| SELMODRAFT_85078 | | D8R4Q6 | | DYW | | 774 | | 258 | | | GL377571:3818389-3819165 | | 0 | | _ | |  |
| SELMODRAFT_85091 | | D8R3N5 | | P | | 1089 | | 363 | | | GL377571:1724516-1725607 | | 0 | | _ | |  |
| SELMODRAFT_85102 | | D8R440 | | P | | 870 | | 290 | | | GL377571:2551219-2552091 | | 0 | | _ | |  |
| SELMODRAFT_85284 | | D8R6N3 | | E | | 1668 | | 556 | | | GL377572:2560180-2562537 | | 2 | | Mitochondria | |  |
| SELMODRAFT_85415 | | D8R5Z8 | | P | | 2907 | | 969 | | | GL377572:1410625-1423451 | | 2 | | _ | |  |
| SELMODRAFT_85839 | | D8R692 | | P | | 1074 | | 358 | | | GL377572:1810075-1811151 | | 0 | | _ | |  |
| SELMODRAFT_85926 | | D8R551 | | DYW | | 2433 | | 811 | | | GL377572:3408641-3411076 | | 0 | | Mitochondria | |  |
| SELMODRAFT_85930 | | D8R5E0 | | P | | 1146 | | 382 | | | GL377572:5175-8667 | | 3 | | _ | |  |
| SELMODRAFT_86032 | | D8R5M6 | | P | | 561 | | 187 | | | GL377572:641444-642007 | | 0 | | _ | |  |
| SELMODRAFT_86162 | | D8R6I6 | | E | | 1104 | | 368 | | | GL377572:2290642-2292258 | | 1 | | _ | |  |
| SELMODRAFT_86197 | | D8R6P3 | | P | | 933 | | 311 | | | GL377572:2620889-2630833 | | 0 | | Mitochondria | |  |
| SELMODRAFT_86236 | | D8R6R9 | | DYW | | 2022 | | 674 | | | GL377572:2783629-2785653 | | 1 | | Mitochondria | |  |
| SELMODRAFT_86341 | | D8R6N8 | | P | | 672 | | 224 | | | GL377572:2602256-2603479 | | 0 | | _ | |  |
| SELMODRAFT_86405 | | D8R5X6 | | DYW | | 2748 | | 916 | | | GL377572:1259732-1262482 | | 0 | | _ | |  |
| SELMODRAFT_86471 | | D8R8E1 | | P | | 588 | | 196 | | | GL377573:2753252-2753842 | | 1 | | _ | |  |
| SELMODRAFT_86789 | | D8R8E8 | | P | | 282 | | 94 | | | GL377573:2781891-2782172 | | 1 | | Mitochondria | |  |
| SELMODRAFT_87015 | | D8R7M8 | | P | | 1470 | | 490 | | | GL377573:1538623-1540761 | | 0 | | _ | |  |
| SELMODRAFT_87043 | | D8R7I8 | | PLS | | 1284 | | 428 | | | GL377573:1261735-1264404 | | 1 | | Mitochondria | |  |
| SELMODRAFT_87107 | | D8R7P4 | | P | | 933 | | 311 | | | GL377573:1582943-1584076 | | 1 | | Mitochondria | |  |
| SELMODRAFT_87167 | | D8R721 | | P | | 618 | | 206 | | | GL377573:465848-466468 | | 0 | | Mitochondria | |  |
| SELMODRAFT_87370 | | D8R8L1 | | DYW | | 2709 | | 903 | | | GL377573:3020031-3022742 | | 0 | | Chloroplast | |  |
| SELMODRAFT_87452 | | D8R8C7 | | P | | 1959 | | 653 | | | GL377573:2694225-2696186 | | 0 | | _ | |  |
| SELMODRAFT_87499 | | D8R749 | | P | | 1167 | | 389 | | | GL377573:574532-576142 | | 2 | | _ | |  |
| SELMODRAFT_87658 | | D8R8M0 | | P | | 690 | | 230 | | | GL377573:3057934-3058623 | | 0 | | _ | |  |
| SELMODRAFT_87828 | | D8R7I9 | | P | | 963 | | 321 | | | GL377573:1264995-1265960 | | 0 | | SP | |  |
| SELMODRAFT_87961 | | D8R9F7 | | P | | 2253 | | 751 | | | GL377574:27962-30259 | | 1 | | SP | |  |
| SELMODRAFT_88008 | | D8R947 | | P | | 2211 | | 737 | | | GL377574:2584670-2586883 | | 0 | | Chloroplast | |  |
| SELMODRAFT_88015 | | D8R9J5 | | P | | 630 | | 210 | | | GL377574:272718-273350 | | 0 | | _ | |  |
| SELMODRAFT_88078 | | D8R8Y9 | | P | | 1128 | | 376 | | | GL377574:2254254-2255384 | | 0 | | _ | |  |
| SELMODRAFT_88115 | | D8R9L7 | | P | | 867 | | 289 | | | GL377574:630216-631154 | | 1 | | _ | |  |
| SELMODRAFT_88255 | | D8R9B4 | | P | | 2235 | | 745 | | | GL377574:2884220-2886937 | | 2 | | _ | |  |
| SELMODRAFT_88274 | | D8RA10 | | P | | 1200 | | 400 | | | GL377574:1412169-1413590 | | 1 | | Chloroplast | |  |
| SELMODRAFT_88277 | | D8R9R4 | | P | | 567 | | 189 | | | GL377574:792929-793780 | | 1 | | _ | |  |
| SELMODRAFT_88400 | | D8RA77 | | PLS | | 2151 | | 717 | | | GL377574:1868627-1871038 | | 1 | | Mitochondria | |  |
| SELMODRAFT_88474 | | D8RA05 | | P | | 2241 | | 747 | | | GL377574:1360217-1362460 | | 0 | | Mitochondria | |  |
| SELMODRAFT_88546 | | D8R9J8 | | P | | 1017 | | 339 | | | GL377574:275729-276745 | | 0 | | _ | |  |
| SELMODRAFT_88629 | | D8RAD2 | | P | | 762 | | 254 | | | GL377574:2129765-2130529 | | 0 | | _ | |  |
| SELMODRAFT_88697 | | D8R9Q0 | | DYW | | 1860 | | 620 | | | GL377574:749141-751003 | | 0 | | SP | |  |
| SELMODRAFT_88791 | | D8R9H2 | | P | | 645 | | 215 | | | GL377574:102061-102705 | | 0 | | Chloroplast | |  |
| SELMODRAFT_88824 | | D8R9I9 | | DYW | | 2010 | | 670 | | | GL377574:243366-245977 | | 1 | | Chloroplast | |  |
| SELMODRAFT_88850 | | D8RAA1 | | E | | 654 | | 218 | | | GL377574:1944992-1945648 | | 0 | | _ | |  |
| SELMODRAFT_89004 | | D8R9Z2 | | E | | 1413 | | 471 | | | GL377574:1311479-1312894 | | 0 | | _ | |  |
| SELMODRAFT_89033 | | D8RBU3 | | P | | 1800 | | 600 | | | GL377575:2710541-2712529 | | 2 | | _ | |  |
| SELMODRAFT_89100 | | D8RAU2 | | P | | 756 | | 252 | | | GL377575:744865-746373 | | 1 | | _ | |  |
| SELMODRAFT_89112 | | D8RBS3 | | P | | 684 | | 228 | | | GL377575:2638226-2638912 | | 0 | | SP | |  |
| SELMODRAFT_89176 | | D8RBR3 | | P | | 1359 | | 453 | | | GL377575:2590479-2591840 | | 0 | | _ | |  |
| SELMODRAFT_89230 | | D8RBV5 | | DYW | | 1893 | | 631 | | | GL377575:2741378-2743876 | | 2 | | Chloroplast | |  |
| SELMODRAFT_89343 | | D8RBI7 | | P | | 1566 | | 522 | | | GL377575:2216217-2218379 | | 1 | | _ | |  |
| SELMODRAFT_89434 | | D8RBC6 | | P | | 969 | | 323 | | | GL377575:1854667-1855638 | | 0 | | _ | |  |
| SELMODRAFT_89588 | | D8RAP1 | | P | | 1758 | | 586 | | | GL377575:488933-491782 | | 1 | | _ | |  |
| SELMODRAFT_89916 | | D8RAT3 | | P | | 1611 | | 537 | | | GL377575:700768-702555 | | 1 | | _ | |  |
| SELMODRAFT_90070 | | D8RDC0 | | P | | 552 | | 184 | | | GL377576:2635853-2636725 | | 1 | | _ | |  |
| SELMODRAFT_90123 | | D8RC53 | | DYW | | 2454 | | 818 | | | GL377576:541497-544145 | | 1 | | _ | |  |
| SELMODRAFT_90172 | | D8RDD2 | | P | | 1197 | | 399 | | | GL377576:2705639-2706838 | | 0 | | _ | |  |
| SELMODRAFT_90222 | | D8RCX4 | | P | | 1203 | | 401 | | | GL377576:1888299-1890017 | | 1 | | Mitochondria | |  |
| SELMODRAFT_90260 | | D8RD59 | | DYW | | 1857 | | 619 | | | GL377576:2337100-2340795 | | 3 | | Chloroplast | |  |
| SELMODRAFT_90331 | | D8RD34 | | P | | 465 | | 155 | | | GL377576:2242567-2243034 | | 0 | | _ | |  |
| SELMODRAFT_90382 | | D8RD06 | | PLS | | 1710 | | 570 | | | GL377576:2014652-2016730 | | 1 | | Mitochondria | |  |
| SELMODRAFT_90409 | | D8RCV3 | | P | | 1080 | | 360 | | | GL377576:1803161-1805164 | | 1 | | _ | |  |
| SELMODRAFT_90480 | | D8RDA0 | | E | | 678 | | 226 | | | GL377576:2569194-2569874 | | 0 | | _ | |  |
| SELMODRAFT_90502 | | D8RDE7 | | P | | 699 | | 233 | | | GL377576:2773620-2774318 | | 0 | | _ | |  |
| SELMODRAFT_90563 | | D8RCP4 | | DYW | | 2088 | | 696 | | | GL377576:1465876-1467966 | | 0 | | _ | |  |
| SELMODRAFT_90575 | | D8RCE5 | | P | | 1188 | | 396 | | | GL377576:1045803-1047152 | | 1 | | _ | |  |
| SELMODRAFT_90583 | | D8RD56 | | P | | 1614 | | 538 | | | GL377576:2332382-2333998 | | 0 | | _ | |  |
| SELMODRAFT_90686 | | D8RCZ8 | | P | | 1290 | | 430 | | | GL377576:1970500-1972620 | | 2 | | Mitochondria | |  |
| SELMODRAFT_90967 | | D8RD65 | | P | | 1209 | | 403 | | | GL377576:2353283-2355640 | | 1 | | _ | |  |
| SELMODRAFT_90996 | | D8RCS7 | | P | | 2430 | | 810 | | | GL377576:1644953-1647592 | | 1 | | Mitochondria | |  |
| SELMODRAFT_91006 | | D8RCP9 | | P | | 1692 | | 564 | | | GL377576:1494530-1496224 | | 0 | | _ | |  |
| SELMODRAFT_91015 | | D8RCZ0 | | P | | 1635 | | 545 | | | GL377576:1944345-1947874 | | 2 | | _ | |  |
| SELMODRAFT_91104 | | D8RCQ0 | | P | | 1311 | | 437 | | | GL377576:1497235-1499427 | | 3 | | _ | |  |
| SELMODRAFT_91306 | | D8REP5 | | P | | 714 | | 238 | | | GL377577:2403823-2404743 | | 1 | | _ | |  |
| SELMODRAFT_91325 | | D8RDQ4 | | P | | 1734 | | 578 | | | GL377577:163468-165759 | | 1 | | Mitochondria | |  |
| SELMODRAFT_91339 | | D8REC0 | | P | | 786 | | 262 | | | GL377577:1776071-1777813 | | 1 | | _ | |  |
| SELMODRAFT_91372 | | D8RDN5 | | P | | 735 | | 245 | | | GL377577:68932-69666 | | 0 | | _ | |  |
| SELMODRAFT_91477 | | D8RDR2 | | P | | 1314 | | 438 | | | GL377577:237297-239252 | | 1 | | _ | |  |
| SELMODRAFT_91663 | | D8REX0 | | P | | 660 | | 220 | | | GL377577:2842977-2844032 | | 1 | | Mitochondria | |  |
| SELMODRAFT_91676 | | D8REN9 | | P | | 1224 | | 408 | | | GL377577:2395345-2396571 | | 0 | | _ | |  |
| SELMODRAFT_91690 | | D8RER8 | | P | | 1161 | | 387 | | | GL377577:2513782-2527139 | | 1 | | _ | |  |
| SELMODRAFT_91775 | | D8RDV5 | | P | | 639 | | 213 | | | GL377577:566184-566822 | | 0 | | Mitochondria | |  |
| SELMODRAFT_91949 | | D8RDT9 | | DYW | | 1335 | | 445 | | | GL377577:389048-390385 | | 0 | | SP | |  |
| SELMODRAFT_92008 | | D8RE15 | | P | | 2547 | | 849 | | | GL377577:812722-815271 | | 0 | | Mitochondria | |  |
| SELMODRAFT_92172 | | D8RFN1 | | P | | 1662 | | 554 | | | GL377578:1555018-1557183 | | 1 | | Mitochondria | |  |
| SELMODRAFT_92207 | | D8RFB3 | | P | | 1371 | | 457 | | | GL377578:874740-876113 | | 0 | | _ | |  |
| SELMODRAFT_92231 | | D8RF90 | | DYW | | 2367 | | 789 | | | GL377578:697433-699802 | | 0 | | _ | |  |
| SELMODRAFT_92328 | | D8RG95 | | P | | 1293 | | 431 | | | GL377578:2470503-2472224 | | 1 | | _ | |  |
| SELMODRAFT_92426 | | D8RFJ2 | | P | | 795 | | 265 | | | GL377578:1382723-1384357 | | 1 | | SP | |  |
| SELMODRAFT_92456 | | D8RFN6 | | P | | 1149 | | 383 | | | GL377578:1591306-1592457 | | 0 | | Mitochondria | |  |
| SELMODRAFT_92471 | | D8RF86 | | E | | 2067 | | 689 | | | GL377578:682044-684386 | | 1 | | _ | |  |
| SELMODRAFT_92492 | | D8RFE1 | | E | | 993 | | 331 | | | GL377578:918847-919842 | | 0 | | _ | |  |
| SELMODRAFT_92565 | | D8RFH2 | | P | | 921 | | 307 | | | GL377578:1194871-1196388 | | 1 | | SP | |  |
| SELMODRAFT_92605 | | D8RFC6 | | DYW | | 465 | | 155 | | | GL377578:888434-888901 | | 0 | | _ | |  |
| SELMODRAFT_92610 | | D8RFU1 | | P | | 771 | | 257 | | | GL377578:1841069-1843470 | | 1 | | Mitochondria | |  |
| SELMODRAFT_92825 | | D8RFF0 | | P | | 507 | | 169 | | | GL377578:947703-949028 | | 1 | | _ | |  |
| SELMODRAFT_92851 | | D8RG32 | | P | | 261 | | 87 | | | GL377578:2241564-2241824 | | 0 | | _ | |  |
| SELMODRAFT_93022 | | D8RFY8 | | P | | 852 | | 284 | | | GL377578:2032349-2033506 | | 1 | | _ | |  |
| SELMODRAFT_93286 | | D8RHK2 | | P | | 1743 | | 581 | | | GL377579:115710-117761 | | 1 | | Mitochondria | |  |
| SELMODRAFT_93292 | | D8RH68 | | P | | 1650 | | 550 | | | GL377579:2120181-2121986 | | 1 | | SP | |  |
| SELMODRAFT_93321 | | D8RGH5 | | DYW | | 2808 | | 936 | | | GL377579:917555-920365 | | 0 | | _ | |  |
| SELMODRAFT_93404 | | D8RHM6 | | P | | 993 | | 331 | | | GL377579:323909-325987 | | 1 | | Mitochondria | |  |
| SELMODRAFT_93439 | | D8RGU0 | | DYW | | 3288 | | 1096 | | | GL377579:1461300-1468990 | | 2 | | _ | |  |
| SELMODRAFT_93575 | | D8RH28 | | P | | 1836 | | 612 | | | GL377579:1792160-1795159 | | 2 | | Mitochondria | |  |
| SELMODRAFT_93612 | | D8RGQ1 | | P | | 2475 | | 825 | | | GL377579:1322335-1324812 | | 0 | | Mitochondria | |  |
| SELMODRAFT_93702 | | D8RGE9 | | P | | 579 | | 193 | | | GL377579:765319-765900 | | 0 | | _ | |  |
| SELMODRAFT_93962 | | D8RGM3 | | P | | 1473 | | 491 | | | GL377579:1163845-1166096 | | 2 | | _ | |  |
| SELMODRAFT_94034 | | D8RIM8 | | P | | 1731 | | 577 | | | GL377580:2631299-2637686 | | 1 | | _ | |  |
| SELMODRAFT_94128 | | D8RIJ9 | | DYW | | 2250 | | 750 | | | GL377580:2272749-2280249 | | 2 | | _ | |  |
| SELMODRAFT_94142 | | D8RIE9 | | P | | 750 | | 250 | | | GL377580:1825578-1826528 | | 1 | | _ | |  |
| SELMODRAFT_94312 | | D8RIJ4 | | P | | 1491 | | 497 | | | GL377580:2220078-2221688 | | 1 | | _ | |  |
| SELMODRAFT_94326 | | D8RIJ7 | | DYW | | 1896 | | 632 | | | GL377580:2244347-2246485 | | 1 | | _ | |  |
| SELMODRAFT_94343 | | D8RI87 | | P | | 714 | | 238 | | | GL377580:1382528-1383448 | | 1 | | _ | |  |
| SELMODRAFT_94570 | | D8RJD7 | | P | | 801 | | 267 | | | GL377581:1511808-1513082 | | 1 | | _ | |  |
| SELMODRAFT_94629 | | D8RIS8 | | DYW | | 1809 | | 603 | | | GL377581:313463-315892 | | 1 | | _ | |  |
| SELMODRAFT_94745 | | D8RIU5 | | P | | 1797 | | 599 | | | GL377581:399744-401543 | | 0 | | _ | |  |
| SELMODRAFT_94769 | | D8RJ38 | | P | | 1371 | | 457 | | | GL377581:894465-895934 | | 1 | | _ | |  |
| SELMODRAFT_94779 | | D8RIY8 | | P | | 1095 | | 365 | | | GL377581:571944-573566 | | 1 | | _ | |  |
| SELMODRAFT_94780 | | D8RJ98 | | P | | 1950 | | 650 | | | GL377581:1214175-1216883 | | 2 | | Mitochondria | |  |
| SELMODRAFT_94889 | | D8RJ16 | | P | | 2538 | | 846 | | | GL377581:785020-789066 | | 3 | | _ | |  |
| SELMODRAFT_94907 | | D8RJQ5 | | P | | 603 | | 201 | | | GL377581:2125547-2126152 | | 0 | | Mitochondria | |  |
| SELMODRAFT_94945 | | D8RJM3 | | E | | 1842 | | 614 | | | GL377581:2024992-2028084 | | 3 | | _ | |  |
| SELMODRAFT_94978 | | D8RJN9 | | P | | 1278 | | 426 | | | GL377581:2062753-2064123 | | 1 | | _ | |  |
| SELMODRAFT_95046 | | D8RIX3 | | P | | 960 | | 320 | | | GL377581:511976-513796 | | 1 | | Mitochondria | |  |
| SELMODRAFT_95056 | | D8RIP6 | | E | | 1071 | | 357 | | | GL377581:76459-81505 | | 1 | | _ | |  |
| SELMODRAFT_95125 | | D8RIX9 | | P | | 546 | | 182 | | | GL377581:544242-544790 | | 0 | | Chloroplast | |  |
| SELMODRAFT_95215 | | D8RIX6 | | P | | 1476 | | 492 | | | GL377581:538589-540337 | | 1 | | _ | |  |
| SELMODRAFT_95253 | | D8RJ74 | | P | | 2442 | | 814 | | | GL377581:1084689-1087130 | | 1 | | Mitochondria | |  |
| SELMODRAFT_95507 | | D8RJZ9 | | P | | 1062 | | 354 | | | GL377582:209893-212873 | | 2 | | _ | |  |
| SELMODRAFT_95540 | | D8RJY9 | | DYW | | 1794 | | 598 | | | GL377582:182752-185121 | | 1 | | _ | |  |
| SELMODRAFT_95554 | | D8RKW2 | | DYW | | 2124 | | 708 | | | GL377582:2006034-2008160 | | 0 | | _ | |  |
| SELMODRAFT_95641 | | D8RKS6 | | P | | 864 | | 288 | | | GL377582:1789663-1790797 | | 1 | | _ | |  |
| SELMODRAFT_95653 | | D8RL05 | | DYW | | 2346 | | 782 | | | GL377582:2323918-2326266 | | 0 | | _ | |  |
| SELMODRAFT_95698 | | D8RK02 | | DYW | | 1686 | | 562 | | | GL377582:223697-225385 | | 0 | | Mitochondria | |  |
| SELMODRAFT_95702 | | D8RKP9 | | P | | 573 | | 191 | | | GL377582:1672964-1673815 | | 1 | | _ | |  |
| SELMODRAFT_95714 | | D8RL31 | | P | | 693 | | 231 | | | GL377582:2424213-2424905 | | 0 | | _ | |  |
| SELMODRAFT_95815 | | D8RKP4 | | P | | 1068 | | 356 | | | GL377582:1658801-1659871 | | 0 | | Mitochondria | |  |
| SELMODRAFT_95851 | | D8RL39 | | E | | 1440 | | 480 | | | GL377582:2443730-2447102 | | 2 | | _ | |  |
| SELMODRAFT_95871 | | D8RKM2 | | P | | 1356 | | 452 | | | GL377582:1565065-1571185 | | 2 | | _ | |  |
| SELMODRAFT_95907 | | D8RKF4 | | P | | 1893 | | 631 | | | GL377582:846702-849215 | | 1 | | _ | |  |
| SELMODRAFT_95956 | | D8RK38 | | P | | 1248 | | 416 | | | GL377582:332606-334219 | | 2 | | _ | |  |
| SELMODRAFT_95989 | | D8RJW8 | | P | | 753 | | 251 | | | GL377582:103938-104693 | | 0 | | SP | |  |
| SELMODRAFT_96029 | | D8RKX7 | | P | | 1668 | | 556 | | | GL377582:2198523-2200373 | | 2 | | _ | |  |
| SELMODRAFT_96058 | | D8RKR9 | | P | | 1266 | | 422 | | | GL377582:1758406-1760595 | | 1 | | Mitochondria | |  |
| SELMODRAFT_96070 | | D8RKG8 | | P | | 846 | | 282 | | | GL377582:1085368-1117325 | | 1 | | _ | |  |
| SELMODRAFT_96075 | | D8RKV7 | | P | | 819 | | 273 | | | GL377582:1994845-1995666 | | 0 | | _ | |  |
| SELMODRAFT_96189 | | D8RKZ9 | | P | | 1626 | | 542 | | | GL377582:2289362-2291167 | | 1 | | Mitochondria | |  |
| SELMODRAFT_96227 | | D8RLW0 | | P | | 831 | | 277 | | | GL377583:887493-888326 | | 0 | | _ | |  |
| SELMODRAFT_96258 | | D8RL52 | | E | | 1200 | | 400 | | | GL377583:1503109-1505214 | | 1 | | SP | |  |
| SELMODRAFT_96294 | | D8RLT2 | | P | | 567 | | 189 | | | GL377583:644483-645052 | | 0 | | Mitochondria | |  |
| SELMODRAFT_96308 | | D8RLX5 | | P | | 537 | | 179 | | | GL377583:933306-933941 | | 1 | | _ | |  |
| SELMODRAFT_96329 | | D8RLV9 | | E | | 1842 | | 614 | | | GL377583:750893-772278 | | 3 | | _ | |  |
| SELMODRAFT_96362 | | D8RLD2 | | P | | 882 | | 294 | | | GL377583:2092134-2093142 | | 1 | | Mitochondria | |  |
| SELMODRAFT_96437 | | D8RL67 | | P | | 522 | | 174 | | | GL377583:1553854-1554378 | | 0 | | Mitochondria | |  |
| SELMODRAFT_96499 | | D8RLQ5 | | DYW | | 1149 | | 383 | | | GL377583:529500-530651 | | 0 | | _ | |  |
| SELMODRAFT_96515 | | D8RLB6 | | P | | 1074 | | 358 | | | GL377583:2022042-2024507 | | 2 | | _ | |  |
| SELMODRAFT_96526 | | D8RLP5 | | P | | 1134 | | 378 | | | GL377583:486623-488083 | | 1 | | Mitochondria | |  |
| SELMODRAFT_96595 | | D8RLE3 | | P | | 963 | | 321 | | | GL377583:2152038-2156496 | | 2 | | _ | |  |
| SELMODRAFT_96626 | | D8RLC5 | | P | | 2265 | | 755 | | | GL377583:2053392-2055659 | | 0 | | _ | |  |
| SELMODRAFT_96789 | | D8RLD9 | | P | | 894 | | 298 | | | GL377583:2136641-2137537 | | 0 | | Mitochondria | |  |
| SELMODRAFT_96794 | | D8RLK2 | | P | | 3348 | | 1116 | | | GL377583:164398-179571 | | 1 | | Chloroplast | |  |
| SELMODRAFT_96876 | | D8RLF5 | | DYW | | 1455 | | 485 | | | GL377583:2195981-2197645 | | 1 | | _ | |  |
| SELMODRAFT_96903 | | D8RM51 | | P | | 570 | | 190 | | | GL377583:1273503-1274261 | | 1 | | Mitochondria | |  |
| SELMODRAFT_96926 | | D8RLG3 | | P | | 1089 | | 363 | | | GL377583:2328436-2329527 | | 0 | | _ | |  |
| SELMODRAFT_96927 | | D8RLQ6 | | DYW | | 1818 | | 606 | | | GL377583:530668-532488 | | 0 | | _ | |  |
| SELMODRAFT_97132 | | D8RNF3 | | P | | 537 | | 179 | | | GL377584:2213240-2213875 | | 1 | | _ | |  |
| SELMODRAFT_97139 | | D8RMZ5 | | E | | 825 | | 275 | | | GL377584:1431164-1432585 | | 1 | | _ | |  |
| SELMODRAFT_97143 | | D8RND8 | | E | | 840 | | 280 | | | GL377584:2173587-2174429 | | 0 | | _ | |  |
| SELMODRAFT_97177 | | D8RN90 | | DYW | | 1455 | | 485 | | | GL377584:1929404-1930861 | | 0 | | _ | |  |
| SELMODRAFT_97233 | | D8RN89 | | P | | 1860 | | 620 | | | GL377584:1925590-1928451 | | 3 | | _ | |  |
| SELMODRAFT_97289 | | D8RMF6 | | P | | 837 | | 279 | | | GL377584:458973-460844 | | 2 | | Mitochondria | |  |
| SELMODRAFT_97343 | | D8RMC5 | | DYW | | 1461 | | 487 | | | GL377584:252034-253791 | | 1 | | SP | |  |
| SELMODRAFT_97435 | | D8RN21 | | P | | 1743 | | 581 | | | GL377584:1509651-1511585 | | 1 | | Chloroplast | |  |
| SELMODRAFT_97496 | | D8RN91 | | DYW | | 1818 | | 606 | | | GL377584:1930878-1932698 | | 0 | | _ | |  |
| SELMODRAFT_97527 | | D8RN24 | | P | | 1692 | | 564 | | | GL377584:1530321-1532306 | | 1 | | _ | |  |
| SELMODRAFT_97561 | | D8RMM6 | | P | | 1218 | | 406 | | | GL377584:794616-795836 | | 0 | | Mitochondria | |  |
| SELMODRAFT_97596 | | D8RMZ8 | | P | | 960 | | 320 | | | GL377584:1440591-1441553 | | 0 | | _ | |  |
| SELMODRAFT_97739 | | D8RND7 | | E | | 1185 | | 395 | | | GL377584:2172213-2173583 | | 1 | | _ | |  |
| SELMODRAFT_97859 | | D8RMJ4 | | DYW | | 1143 | | 381 | | | GL377584:656131-657276 | | 0 | | SP | |  |
| SELMODRAFT_97920 | | D8RNJ8 | | E | | 1323 | | 441 | | | GL377585:167256-169016 | | 2 | | _ | |  |
| SELMODRAFT_97942 | | D8RNS8 | | P | | 867 | | 289 | | | GL377585:618810-620096 | | 1 | | Chloroplast | |  |
| SELMODRAFT_98177 | | D8RPC5 | | P | | 675 | | 225 | | | GL377585:1472702-1474399 | | 1 | | Mitochondria | |  |
| SELMODRAFT_98207 | | D8RPB4 | | DYW | | 2094 | | 698 | | | GL377585:1388757-1410583 | | 2 | | _ | |  |
| SELMODRAFT_98388 | | D8RNX2 | | P | | 780 | | 260 | | | GL377585:773681-774769 | | 1 | | _ | |  |
| SELMODRAFT_98396 | | D8RNV3 | | P | | 1023 | | 341 | | | GL377585:703941-705884 | | 2 | | Chloroplast | |  |
| SELMODRAFT_98426 | | D8RP13 | | DYW | | 1803 | | 601 | | | GL377585:952719-954524 | | 0 | | _ | |  |
| SELMODRAFT_98472 | | D8RPM4 | | E | | 1593 | | 531 | | | GL377585:1977535-1979346 | | 1 | | _ | |  |
| SELMODRAFT_98546 | | D8RPC9 | | P | | 1773 | | 591 | | | GL377585:1493516-1499464 | | 4 | | _ | |  |
| SELMODRAFT_98550 | | D8RNS5 | | P | | 1131 | | 377 | | | GL377585:610179-611312 | | 0 | | _ | |  |
| SELMODRAFT_98565 | | D8RP49 | | P | | 561 | | 187 | | | GL377585:1090746-1091309 | | 0 | | Mitochondria | |  |
| SELMODRAFT_98704 | | D8RPQ0 | | P | | 1527 | | 509 | | | GL377585:2090815-2092965 | | 1 | | _ | |  |
| SELMODRAFT_98781 | | D8RQ23 | | E | | 798 | | 266 | | | GL377586:1392198-1392998 | | 0 | | _ | |  |
| SELMODRAFT_98793 | | D8RQD7 | | P | | 1167 | | 389 | | | GL377586:1927963-1931673 | | 1 | | _ | |  |
| SELMODRAFT_98955 | | D8RQ58 | | E | | 1638 | | 546 | | | GL377586:1484761-1486401 | | 0 | | _ | |  |
| SELMODRAFT_98963 | | D8RQM7 | | P | | 1281 | | 427 | | | GL377586:289322-291166 | | 1 | | _ | |  |
| SELMODRAFT_99426 | | D8RRE1 | | P | | 1743 | | 581 | | | GL377587:1093433-1095586 | | 1 | | _ | |  |
| SELMODRAFT_99647 | | D8RR22 | | E | | 2025 | | 675 | | | GL377587:419485-421512 | | 0 | | _ | |  |
| SELMODRAFT_99651 | | D8RRK4 | | P | | 1608 | | 536 | | | GL377587:1404600-1407173 | | 2 | | _ | |  |
| SELMODRAFT_99677 | | D8RR68 | | P | | 1641 | | 547 | | | GL377587:700146-702398 | | 2 | | _ | |  |
| SELMODRAFT_99745 | | D8RRQ5 | | P | | 933 | | 311 | | | GL377587:1747171-1748106 | | 0 | | _ | |  |
| SELMODRAFT_99756 | | D8RRG5 | | P | | 2508 | | 836 | | | GL377587:1268779-1271289 | | 0 | | _ | |  |
| SELMODRAFT_99957 | | D8RRC5 | | P | | 1719 | | 573 | | | GL377587:1042929-1045016 | | 1 | | Mitochondria | |  |
| SELMODRAFT_99975 | | D8RST9 | | P | | 711 | | 237 | | | GL377588:1858161-1859171 | | 1 | | _ | |  |
| SELMODRAFT_99993 | | D8RRV8 | | P | | 1137 | | 379 | | | GL377588:112692-114707 | | 1 | | _ | |  |
|  |  | |  | |  | |  | |  |  | |  | |  | |  | |
