# Supplementary figures and images for: Genome-wide investigation and expression analyses of the pentatricopeptide repeat protein gene family in foxtail millet
Source: BMC Genomics. 2016 Oct 28;17:840. doi: 10.1186/s12864-016-3184-2 (PMC5084403; doi:10.1186/s12864-016-3184-2)

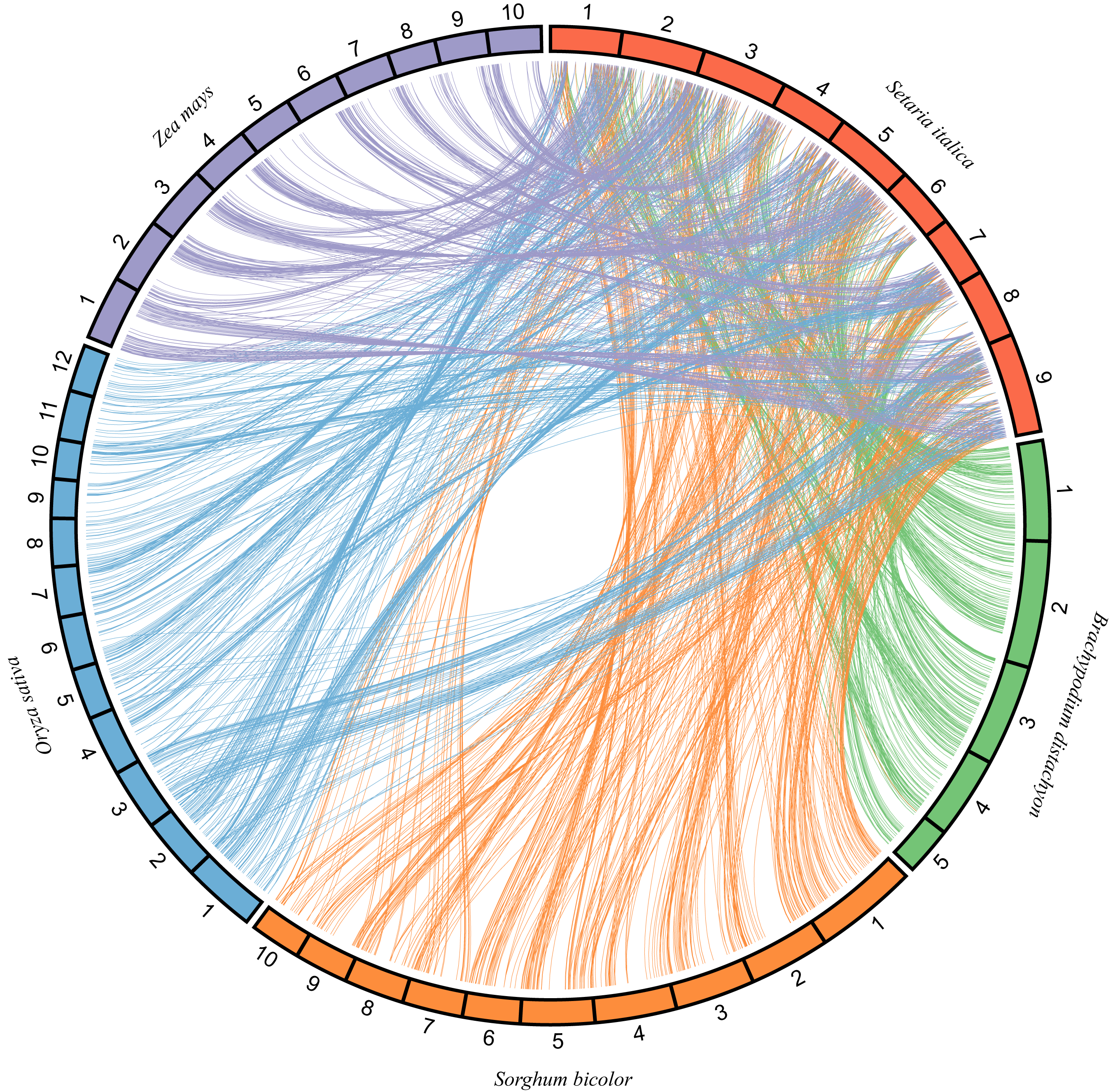

Supplement: Additional file 5: Figure S1. — Orthologous relationships of the PPR genes between foxtail millet and those of other grass species. (TIF 5719 kb) [file 12864_2016_3184_MOESM5_ESM.tif]

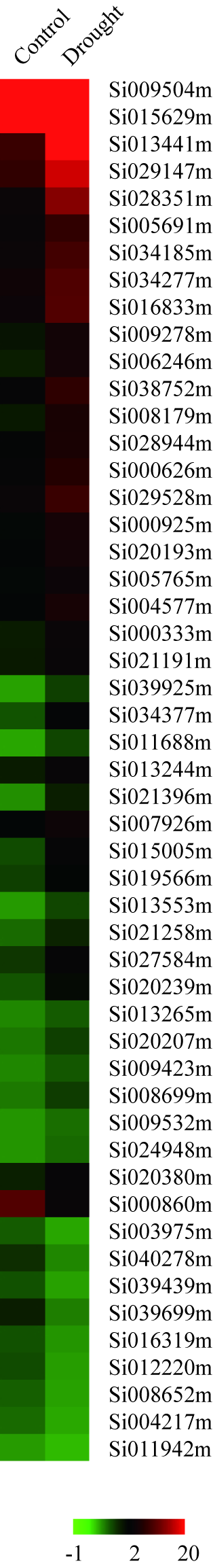

Supplement: Additional file 14: Figure S2. — The heat map of the expression of 51 PPR genes in foxtail millet under drought treatedment (20 % PEG 6000) based on RPKM values. (TIF 163 kb) [file 12864_2016_3184_MOESM14_ESM.tif]
