# Supplementary material for: Genome-wide investigation and expression analyses of the pentatricopeptide repeat protein gene family in foxtail millet
Source: BMC Genomics. 2016 Oct 28;17:840. doi: 10.1186/s12864-016-3184-2 (PMC5084403; doi:10.1186/s12864-016-3184-2)
Supplement: Additional file 6: Table S5. — The Ka/Ks ratios and the estimated divergence times for putatively tandemly duplicated SiPPR genes. (DOCX 20 kb) [file 12864_2016_3184_MOESM6_ESM.docx]

| **Table S5.** The dN/dS ratios and the estimated divergence times for putatively tandemly duplicated SiPPR genes. | | | | | | | |
| --- | --- | --- | --- | --- | --- | --- | --- |
| **Group** | **Gene IDs** | **Duplicate** | **Distance （kb）** | **dS** | **dN** | **dN/dS** | **Mya** |
| **1** | Si016264m | Si016291m | 41.454 | 45.14 | 1.52 | 0.033 | 3472.307692 |
| **2** | Si016257m | Si016610m | 29.492 | 50.8385 | 1.2231 | 0.0241 | 3910.653846 |
| **3** | Si019792m | Si017469m | 11.767 | 50.6016 | 1.4336 | 0.0283 | 3892.430769 |
| **4** | Si019714m | Si016535m | 46.224 | 49.3676 | 1.0936 | 0.0222 | 3797.507692 |
| **5** | Si020380m | Si020192m | 0.377 | 50.7756 | 0.9291 | 0.0183 | 3905.815385 |
| **6** | Si020221m | Si016997m | 5.354 | 79.2463 | 1.002 | 0.0126 | 6095.869231 |
| **7** | Si012630m | Si009450m | 7.243 | 0.1616 | 0.0634 | 0.3923 | 12.43076923 |
| **8** | Si009532m | Si012024m | 8.322 | 48.7448 | 0.9835 | 0.0202 | 3749.6 |
| **9** | Si012259m | Si012001m | 20.651 | 18.8098 | 0.8559 | 0.0455 | 1446.907692 |
| **10** | Si009638m | Si009423m | 27.641 | 47.3556 | 0.9937 | 0.021 | 3642.738462 |
| **11** | Si010939m | Si009770m | 22.433 | 53.5491 | 0.9696 | 0.0181 | 4119.161538 |
| **12** | Si009676m | Si011726m | 29.866 | 29.6047 | 1.1323 | 0.0382 | 2277.284615 |
| **13** | Si033103m | Si032416m | 36.459 | 2.7389 | 1.0328 | 0.3771 | 210.6846154 |
| **14** | Si028988m | Si032483m | 23.012 | 20.0169 | 1.235 | 0.0617 | 1539.761538 |
| **15** | Si029147m | Si033390m | 43.355 | 11.3814 | 0.9475 | 0.0833 | 875.4923077 |
| **16** | Si028089m | Si027770m | 7.734 | 50.8344 | 1.1021 | 0.0217 | 3910.338462 |
| **17** | Si028041m | Si027712m | 30.21 | 2.836 | 0.8747 | 0.3084 | 218.1538462 |
| **18** | Si027756m | Si027905m | 28.106 | 0.1149 | 0.1022 | 0.8898 | 8.838461538 |
| **19** | Si027905m | Si026059m | 20.337 | 2.4089 | 1.219 | 0.5061 | 185.3 |
| **20** | Si021546m | Si025098m | 9.449 | 18.7613 | 1.2462 | 0.0664 | 1443.176923 |
| **21** | Si025085m | Si024715m | 5.643 | 0.476 | 0.2005 | 0.4211 | 36.61538462 |
| **22** | Si025355m | Si025343m | 24.882 | 48.0096 | 1.1784 | 0.0245 | 3693.046154 |
| **23** | Si021338m | Si024194m | 44.004 | 1.7345 | 1.2824 | 0.7393 | 133.4230769 |
| **24** | Si008514m | Si007926m | 8.665 | 14.6672 | 0.7046 | 0.048 | 1128.246154 |
| **25** | Si006121m | Si006059m | 33.911 | 49.3995 | 1.4318 | 0.029 | 3799.961538 |
| **26** | Si005769m | Si008760m | 16.094 | 46.5475 | 1.0396 | 0.0223 | 3580.576923 |
| **27** | Si006322m | Si008760m | 9.376 | 47.2994 | 1.0928 | 0.0231 | 3638.415385 |
| **28** | Si005931m | Si008099m | 8.126 | 47.3919 | 1.0178 | 0.0215 | 3645.530769 |
| **29** | Si040278m | Si039680m | 5.885 | 21.318 | 1.0617 | 0.0498 | 1639.846154 |
| **30** | Si034454m | Si034766m | 17.365 | 5.8261 | 1.4831 | 0.2546 | 448.1615385 |
| **31** | Si035349m | Si035359m | 1.797 | 11.9626 | 0.79 | 0.066 | 920.2 |
| **32** | Si034189m | Si039892m | 29.137 | 0.8953 | 0.3386 | 0.3782 | 68.86923077 |
| **33** | Si034478m | Si034956m | 27.148 | 4.8208 | 1.3616 | 0.2824 | 370.8307692 |
| **34** | Si039489m | Si034734m | 25.879 | 3.5045 | 1.4217 | 0.4057 | 269.5769231 |
| **35** | Si034336m | Si034560m | 27.064 | 47.6635 | 1.4476 | 0.0304 | 3666.423077 |
| **36** | Si034307m | Si034006m | 47.323 | 52.1224 | 1.131 | 0.0217 | 4009.415385 |
| **37** | Si038545m | Si040060m | 3.041 | 49.5548 | 1.2276 | 0.0248 | 3811.907692 |
| **38** | Si040060m | Si034513m | 24.039 | 37.1841 | 1.1118 | 0.0299 | 2860.315385 |
| **39** | Si039802m | Si038646m | 48.953 | 16.6536 | 0.7737 | 0.0465 | 1281.046154 |
| **40** | Si039699m | Si035086m | 33.652 | 3.3745 | 0.6238 | 0.1849 | 259.5769231 |
| **41** | Si035086m | Si040248m | 7.373 | 47.2586 | 1.1282 | 0.0239 | 3635.276923 |
| **42** | Si005172m | Si001037m | 12.124 | 2.8296 | 0.5825 | 0.2059 | 217.6615385 |
| **43** | Si004356m | Si000425m | 34.7 | 19.3482 | 0.7069 | 0.0365 | 1488.323077 |
| **44** | Si002242m | Si000819m | 3.36 | 57.4955 | 0.9067 | 0.0158 | 4422.730769 |
| **45** | Si004569m | Si004167m | 22.536 | 6.2124 | 0.8429 | 0.1357 | 477.8769231 |
| **46** | Si004501m | Si004100m | 4.156 | 51.8582 | 0.6275 | 0.0121 | 3989.092308 |
| **47** | Si004766m | Si005072m | 3.629 | 0.2448 | 0.1173 | 0.4791 | 18.83076923 |
| **48** | Si003992m | Si000643m | 49.658 | 52.686 | 0.7771 | 0.0147 | 4052.769231 |
| **49** | Si005164m | Si001169m | 22.013 | 1.7666 | 1.2784 | 0.7237 | 135.8923077 |
| **50** | Si001169m | Si000780m | 27.099 | 2.8201 | 1.2511 | 0.4436 | 216.9307692 |
| **51** | Si015005m | Si013359m | 45.671 | 0.1427 | 0.0627 | 0.4395 | 10.97692308 |
| **52** | Si015417m | Si013553m | 3.971 | 49.1388 | 1.2753 | 0.026 | 3779.907692 |
| **Mean** | | | 21.68769231 | 27.60567692 | 0.966076923 | 0.166317308 | 2123.513609 |
