# Supplementary material for: Genome-wide investigation and expression analyses of the pentatricopeptide repeat protein gene family in foxtail millet
Source: BMC Genomics. 2016 Oct 28;17:840. doi: 10.1186/s12864-016-3184-2 (PMC5084403; doi:10.1186/s12864-016-3184-2)
Supplement: Additional file 7: Table S6. — The Ks/Ka ratios and the estimated divergence times for putatively segmentally duplicated SiPPR genes. Detailed segmental duplication information including domains present, chromosomal location, E-value, and % homology are provided for each putatively segmentally duplicated SiPPR gene. (DOCX 30 kb) [file 12864_2016_3184_MOESM7_ESM.docx]

**Table S6.** The dS/dN ratios and the estimated divergence times for putatively segmentally duplicated SiPPR genes. Detailed segmental duplication information including domains present, chromosomal location, E-value, and % homology are provided for each putatively segmentally duplicated SiPPR gene.

| **Gene 1** | **SMART/PfamA Domain/Class** | **locus** | **Chr.** | **Gene 2** | **SMART/PfamA Domain/Class** | **locus** | **Chr.** | **e-value** | **%Homology** | **dS** | **dN** | **dN/dS** | **Mya** |
| --- | --- | --- | --- | --- | --- | --- | --- | --- | --- | --- | --- | --- | --- |
| Si000252m.g | DYW | 30483839-30486638 | 5 | Si005933m.g | DYW | 537041-539254 | 4 | 0 | 39.27 | 13.7039 | 0.5373 | 0.0392 | 1054.1 |
| Si000252m.g | DYW | 30483839-30486638 | 5 | Si009326m.g | DYW | 19118936-19121834 | 7 | 0 | 98.42 | 0.0104 | 0.0072 | 0.6969 | 0.8 |
| Si000316m.g | DYW | 19714369-19716978 | 5 | Si025222m.g | DYW | 18483214-18485832 | 3 | 0 | 38.63 | 49.2942 | 0.6231 | 0.0126 | 3791.9 |
| Si000316m.g | DYW | 19714369-19716978 | 5 | Si034130m.g | DYW | 48928469-48932088 | 9 | 0 | 38 | 15.9625 | 0.6373 | 0.0399 | 1227.9 |
| Si000325m.g | E | 47128378-47130905 | 5 | Si034130m.g | DYW | 48928469-48932088 | 9 | 0 | 38.92 | 35.1819 | 0.6053 | 0.0172 | 2706.3 |
| Si000377m.g | DYW | 46754222-46756681 | 5 | Si034130m.g | DYW | 48928469-48932088 | 9 | 0 | 37.12 | 14.6396 | 0.6373 | 0.0435 | 1126.1 |
| Si000377m.g | DYW | 46754222-46756681 | 5 | Si040279m.g | DYW | 952102-955147 | 9 | 0 | 47.17 | 3.8171 | 0.6085 | 0.1594 | 293.62 |
| Si000565m.g | P | 38713970-38716978 | 5 | Si034731m.g | P | 12755603-12757522 | 9 | 0 | 49.66 | 1.3786 | 0.3762 | 0.2729 | 106.05 |
| Si001526m.g | P | 38848725-38850376 | 5 | Si004766m.g | P | 38853949-38855376 | 5 | 0 | 71.83 | 0.2611 | 0.1338 | 0.5125 | 20.085 |
| Si001716m.g | P | 4405325-4408308 | 5 | Si031917m.g | P | 38261317-38262474 | 2 | 0 | 96.11 | 0.0864 | 0.0193 | 0.2239 | 6.6462 |
| Si001716m.g | P | 4405325-4408308 | 5 | Si031929m.g | P | 7550538-7551683 | 2 | 0 | 96.33 | 0.0427 | 0.0189 | 0.4428 | 3.2846 |
| Si003790m.g | E | 47242452-47244805 | 5 | Si012752m.g | PLS | 35952992-35955372 | 7 | 0 | 97.11 | 0.0087 | 0.0123 | 1.4054 | 0.6692 |
| Si003790m.g | E | 47242452-47244805 | 5 | Si028360m.g | PLS PGAM | 1398082-1401749 | 8 | 0 | 70.66 | 0.7689 | 0.229 | 0.2978 | 59.146 |
| Si003790m.g | E | 47242452-47244805 | 5 | Si039392m.g | P | 58899438-58901785 | 9 | 0 | 91.88 | 0.0157 | 0.0167 | 1.0603 | 1.2077 |
| Si004230m.g | DYW | 4094435-4097284 | 5 | Si011688m.g | DYW | 22462397-22465258 | 7 | 0 | 88.67 | 0.0905 | 0.0379 | 0.4182 | 6.9615 |
| Si004766m.g | P | 38853949-38855376 | 5 | Si005072m.g | E | 38857578-38858975 | 5 | 0 | 0.69 | 0.2448 | 0.1173 | 0.4791 | 18.831 |
| Si005165m.g | P | 12535592-12537370 | 5 | Si032737m.g | P | 45558201-45560021 | 2 | 0 | 98.82 | 0.0107 | 0.0059 | 0.5509 | 0.8231 |
| Si005166m.g | P | 5363770-5366064 | 5 | Si012426m.g | P | 35255292-35257908 | 7 | 0 | 78.9 | 46.2649 | 0.1544 | 0.0033 | 3558.8 |
| Si005166m.g | P | 5363770-5366064 | 5 | Si026106m.g | PLS | 2040430-2042765 | 8 | 0 | 70.27 | 0.5739 | 0.3386 | 0.59 | 44.146 |
| Si005166m.g | P | 5363770-5366064 | 5 | Si027424m.g | P | 1897936-1899849 | 8 | 0 | 70.27 | 0.3894 | 0.3077 | 0.7903 | 29.954 |
| Si005891m.g | DYW | 3467239-3469605 | 4 | Si005933m.g | DYW | 537041-539254 | 4 | 0 | 44.18 | 18.8165 | 0.5256 | 0.0279 | 1447.4 |
| Si005891m.g | DYW | 3467239-3469605 | 4 | Si034333m.g | DYW | 43519077-43521436 | 9 | 0 | 42.39 | 2.4285 | 0.564 | 0.2323 | 186.81 |
| Si005933m.g | DYW | 537041-539254 | 4 | Si034313m.g | DYW | 9844384-9846834 | 9 | 0 | 41.78 | 4.0787 | 0.5431 | 0.1332 | 313.75 |
| Si005933m.g | DYW | 537041-539254 | 4 | Si034333m.g | DYW | 43519077-43521436 | 9 | 0 | 43.51 | 2.8332 | 0.5672 | 0.2002 | 217.94 |
| Si007926m.g | DYW | 8791450-8793255 | 4 | Si028351m.g | E | 40415776-40417329 | 8 | 0 | 96.14 | 0.1583 | 0.0143 | 0.09 | 12.177 |
| Si009450m.g | P | 15690397-15692828 | 7 | Si012630m.g | P | 15683154-15685289 | 7 | 0 | 69.95 | 0.1616 | 0.0634 | 0.3923 | 12.431 |
| Si009450m.g | P | 15690397-15692828 | 7 | Si027905m.g | PLS | 31107278-31110804 | 8 | 0 | 62.8 | 0.656 | 0.2882 | 0.4393 | 50.462 |
| Si009450m.g | P | 15690397-15692828 | 7 | Si028006m.g | P | 30333402-30335919 | 8 | 0 | 57.33 | 0.7583 | 0.2848 | 0.3755 | 58.331 |
| Si009450m.g | P | 15690397-15692828 | 7 | Si028348m.g | P | 31201831-31204264 | 8 | 0 | 56.98 | 0.7127 | 0.2829 | 0.397 | 54.823 |
| Si009592m.g | DYW | 35633431-35635386 | 7 | Si027770m.g | PLAC8 DUF2985 DYW | 980489-984211 | 8 | 0 | 92.96 | 0.129 | 0.0219 | 0.1697 | 9.9231 |
| Si011688m.g | DYW | 22462397-22465258 | 7 | Si013161m.g | DYW | 33093915-33097446 | 6 | 0 | 35.33 | 23.2441 | 0.6206 | 0.0267 | 1788 |
| Si011688m.g | DYW | 22462397-22465258 | 7 | Si025222m.g | DYW | 18483214-18485832 | 3 | 0 | 36.21 | 11.8206 | 0.6462 | 0.0547 | 909.28 |
| Si011688m.g | DYW | 22462397-22465258 | 7 | Si034130m.g | DYW | 48928469-48932088 | 9 | 0 | 34.3 | 8.5286 | 0.7129 | 0.0836 | 656.05 |
| Si011726m.g | P | 35444897-35447059 | 7 | Si028380m.g | P | 2230148-2232298 | 8 | 0 | 78.13 | 0.3345 | 0.1288 | 0.3851 | 25.731 |
| Si011942m.g | P | 33821002-33823140 | 7 | Si035003m.g | PLS | 47071665-47073354 | 9 | 0 | 86.46 | 0.2039 | 0.0738 | 0.362 | 15.685 |
| Si012220m.g | P | 34175516-34178074 | 7 | Si025982m.g | PLS | 750605-753279 | 8 | 0 | 89.22 | 0.077 | 0.0131 | 0.1697 | 5.9231 |
| Si012426m.g | P | 35255292-35257908 | 7 | Si027424m.g | P | 1897936-1899849 | 8 | 0 | 72.03 | 0.3885 | 0.1762 | 0.4535 | 29.885 |
| Si012426m.g | P | 35255292-35257908 | 7 | Si027905m.g | PLS | 31107278-31110804 | 8 | 0 | 45.95 | 1.2686 | 0.4254 | 0.3353 | 97.585 |
| Si012426m.g | P | 35255292-35257908 | 7 | Si028006m.g | P | 30333402-30335919 | 8 | 0 | 43.58 | 1.5234 | 0.4796 | 0.3148 | 117.18 |
| Si012426m.g | P | 35255292-35257908 | 7 | Si034189m.g | P | 14623370-14626112 | 9 | 0 | 50.97 | 0.7541 | 0.3363 | 0.446 | 58.008 |
| Si012507m.g | P | 15763106-15765434 | 7 | Si009450m.g | P | 15690397-15692828 | 7 | 0 | 52.36 | 0.723 | 0.3123 | 0.4319 | 55.615 |
| Si012507m.g | P | 15763106-15765434 | 7 | Si027905m.g | PLS | 31107278-31110804 | 8 | 0 | 54.49 | 0.7639 | 0.3744 | 0.4902 | 58.762 |
| Si012507m.g | P | 15763106-15765434 | 7 | Si028006m.g | P | 30333402-30335919 | 8 | 0 | 58.28 | 0.6109 | 0.2767 | 0.4529 | 46.992 |
| Si012711m.g | P | 34119609-34121333 | 7 | Si027872m.g | P | 811515-813155 | 8 | 0 | 85.71 | 0.1242 | 0.0713 | 0.574 | 9.5538 |
| Si012752m.g | PLS | 35952992-35955372 | 7 | Si028360m.g | PLS PGAM | 1398082-1401749 | 8 | 0 | 70.66 | 0.7451 | 0.2479 | 0.3327 | 57.315 |
| Si012752m.g | PLS | 35952992-35955372 | 7 | Si039392m.g | P | 58899438-58901785 | 9 | 0 | 91.98 | 0.0111 | 0.0176 | 1.5928 | 0.8538 |
| Si013265m.g | P | 2399796-2402691 | 6 | Si015406m.g | P | 32806976-32809363 | 6 | 0 | 85.42 | 0.1396 | 0.0634 | 0.4543 | 10.738 |
| Si013265m.g | P | 2399796-2402691 | 6 | Si021258m.g | P | 6334285-6336675 | 3 | 0 | 67.04 | 0.4488 | 0.205 | 0.4568 | 34.523 |
| Si013359m.g | E | 848981-852690 | 6 | Si015005m.g | P | 803310-804796 | 6 | 0 | 99.37 | 0.1472 | 0.0587 | 0.3989 | 11.323 |
| Si015328m.g | PLS | 34479834-34482470 | 6 | Si020192m.g | P | 41755814-41757189 | 1 | 0 | 87.28 | 0.0363 | 0.0245 | 0.6752 | 2.7923 |
| Si015406m.g | P | 32806976-32809363 | 6 | Si021258m.g | P | 6334285-6336675 | 3 | 0 | 63.1 | 0.4247 | 0.2179 | 0.513 | 32.669 |
| Si016590m.g | P | 22107709-22110780 | 1 | Si019135m.g | P | 22082060-22084350 | 1 | 0 | 46.67 | 0.9357 | 0.3381 | 0.3614 | 71.977 |
| Si016662m.g | PLS | 9386650-9388512 | 1 | Si028047m.g | DYW | 9975499-9977913 | 8 | 0 | 88.44 | 0.1348 | 0.0935 | 0.694 | 10.369 |
| Si017139m.g | P | 32196599-32198299 | 1 | Si020047m.g | P | 31585192-31586562 | 1 | 0 | 75.76 | 0.2281 | 0.131 | 0.5745 | 17.546 |
| Si019387m.g | P | 27884244-27886144 | 1 | Si034307m.g | E | 42004849-42007257 | 9 | 0 | 80.96 | 0.2352 | 0.1131 | 0.4809 | 18.092 |
| Si019494m.g | DYW | 4637358-4639542 | 1 | Si028041m.g | DYW | 30521648-30523845 | 8 | 0 | 94.13 | 0.1629 | 0.0319 | 0.1961 | 12.531 |
| Si020204m.g | DYW | 29526724-29529375 | 1 | Si000252m.g | DYW | 30483839-30486638 | 5 | 0 | 38.32 | 47.998 | 0.6146 | 0.0128 | 3692.2 |
| Si020204m.g | DYW | 29526724-29529375 | 1 | Si000316m.g | DYW | 19714369-19716978 | 5 | 0 | 38.64 | 15.9625 | 0.6373 | 0.0399 | 1227.9 |
| Si020204m.g | DYW | 29526724-29529375 | 1 | Si000377m.g | DYW | 46754222-46756681 | 5 | 0 | 44.56 | 13.1773 | 0.5991 | 0.0455 | 1013.6 |
| Si020204m.g | DYW | 29526724-29529375 | 1 | Si004230m.g | DYW | 4094435-4097284 | 5 | 0 | 43.74 | 4.1336 | 0.6282 | 0.152 | 317.97 |
| Si020204m.g | DYW | 29526724-29529375 | 1 | Si004364m.g | DYW | 5702298-5704712 | 5 | 0 | 43.54 | 8.9933 | 0.5836 | 0.0649 | 691.79 |
| Si020204m.g | DYW | 29526724-29529375 | 1 | Si005922m.g | DYW | 26581248-26583865 | 4 | 0 | 40.13 | 57.7751 | 0.5451 | 0.0094 | 4444.2 |
| Si020204m.g | DYW | 29526724-29529375 | 1 | Si005933m.g | DYW | 537041-539254 | 4 | 0 | 80.96 | 10.4026 | 0.5688 | 0.0547 | 800.2 |
| Si020204m.g | DYW | 29526724-29529375 | 1 | Si009326m.g | DYW | 19118936-19121834 | 7 | 0 | 43.09 | 47.9155 | 0.6137 | 0.0128 | 3685.8 |
| Si020204m.g | DYW | 29526724-29529375 | 1 | Si011688m.g | DYW | 22462397-22465258 | 7 | 0 | 39.67 | 10.0486 | 0.6234 | 0.062 | 772.97 |
| Si020204m.g | DYW | 29526724-29529375 | 1 | Si025222m.g | DYW | 18483214-18485832 | 3 | 0 | 38.32 | 2.5763 | 0.5912 | 0.2295 | 198.18 |
| Si020204m.g | DYW | 29526724-29529375 | 1 | Si034130m.g | DYW | 48928469-48932088 | 9 | 0 | 84.64 | 45.7331 | 0.6059 | 0.0132 | 3517.9 |
| Si021191m.g | DYW | 9539489-9542086 | 3 | Si013161m.g | DYW | 33093915-33097446 | 6 | 0 | 38.64 | 5.2397 | 0.6191 | 0.1181 | 403.05 |
| Si021453m.g | P | 12273424-12275334 | 3 | Si024301m.g | P | 22067758-22069746 | 3 | 0 | 58.85 | 1.5772 | 0.3632 | 0.2303 | 121.32 |
| Si021705m.g | E | 2897230-2899489 | 3 | Si032802m.g | E | 25213462-25215791 | 2 | 0 | 84.64 | 0.2653 | 0.0961 | 0.362 | 20.408 |
| Si024607m.g | DYW | 12724055-12726694 | 3 | Si034471m.g | DYW | 48021358-48023478 | 9 | 0 | 46.74 | 3.4449 | 0.4522 | 0.1313 | 264.99 |
| Si025318m.g | DYW | 4381945-4384001 | 3 | Si034527m.g | DYW | 14456423-14458942 | 9 | 0 | 92.23 | 0.0252 | 0.0215 | 0.8537 | 1.9385 |
| Si025355m.g | P | 25153908-25155785 | 3 | Si038942m.g | DYW | 35458481-35460391 | 9 | 0 | 56.11 | 0.4328 | 0.2242 | 0.518 | 33.292 |
| Si026106m.g | PLS | 2040430-2042765 | 8 | Si012507m.g | P | 15763106-15765434 | 7 | 0 | 42.8 | 1.3071 | 0.3981 | 0.3045 | 100.55 |
| Si026106m.g | PLS | 2040430-2042765 | 8 | Si027424m.g | P | 1897936-1899849 | 8 | 0 | 62.18 | 0.1966 | 0.1304 | 0.6633 | 15.123 |
| Si027756m.g | P | 31079172-31081289 | 8 | Si012507m.g | P | 15763106-15765434 | 7 | 0 | 57.7 | 0.639 | 0.2589 | 0.4052 | 49.154 |
| Si027756m.g | P | 31079172-31081289 | 8 | Si028006m.g | P | 30333402-30335919 | 8 | 0 | 89.94 | 0.0872 | 0.0598 | 0.6858 | 6.7077 |
| Si027760m.g | P | 29882484-29884928 | 8 | Si027756m.g | P | 31079172-31081289 | 8 | 0 | 85.79 | 0.0551 | 0.0557 | 1.0097 | 4.2385 |
| Si027760m.g | P | 29882484-29884928 | 8 | Si027905m.g | PLS | 31107278-31110804 | 8 | 0 | 68.12 | 0.1038 | 0.0555 | 0.5342 | 7.9846 |
| Si027760m.g | P | 29882484-29884928 | 8 | Si028006m.g | P | 30333402-30335919 | 8 | 0 | 84.79 | 0.0862 | 0.0562 | 0.6517 | 6.6308 |
| Si027760m.g | P | 29882484-29884928 | 8 | Si028348m.g | P | 31201831-31204264 | 8 | 0 | 83.2 | 0.1039 | 0.0669 | 0.6437 | 7.9923 |
| Si027905m.g | PLS | 31107278-31110804 | 8 | Si027756m.g | P | 31079172-31081289 | 8 | 0 | 85.79 | 0.1149 | 0.1022 | 0.8898 | 8.8385 |
| Si028006m.g | P | 30333402-30335919 | 8 | Si027905m.g | PLS | 31107278-31110804 | 8 | 0 | 68.12 | 0.093 | 0.0747 | 0.8029 | 7.1538 |
| Si028921m.g | TPR | 45886670-45889398 | 2 | Si029246m.g | P | 48552124-48554650 | 2 | 0 | 83.68 | 0.2324 | 0.1597 | 0.6872 | 17.877 |
| Si033236m.g | P | 31404170-31406058 | 2 | Si025228m.g | P | 21967236-21968773 | 3 | 0 | 78.92 | 0.1293 | 0.0711 | 0.55 | 9.9462 |
| Si034130m.g | DYW | 48928469-48932088 | 9 | Si009450m.g | P | 15690397-15692828 | 7 | 0 | 55.59 | 0.1616 | 0.0634 | 0.3923 | 12.431 |
| Si034130m.g | DYW | 48928469-48932088 | 9 | Si012507m.g | P | 15763106-15765434 | 7 | 0 | 37.12 | 0.6915 | 0.292 | 0.4223 | 53.192 |
| Si034130m.g | DYW | 48928469-48932088 | 9 | Si027905m.g | PLS | 31107278-31110804 | 8 | 0 | 38.16 | 0.7171 | 0.2975 | 0.4149 | 55.162 |
| Si034130m.g | DYW | 48928469-48932088 | 9 | Si028006m.g | P | 30333402-30335919 | 8 | 0 | 55.59 | 0.6539 | 0.2542 | 0.3887 | 50.3 |
| Si034130m.g | DYW | 48928469-48932088 | 9 | Si028348m.g | P | 31201831-31204264 | 8 | 0 | 68.12 | 0.599 | 0.2531 | 0.4225 | 46.077 |
| Si034189m.g | P | 14623370-14626112 | 9 | Si012507m.g | P | 15763106-15765434 | 7 | 0 | 40.13 | 1.2266 | 0.4428 | 0.361 | 94.354 |
| Si034189m.g | P | 14623370-14626112 | 9 | Si027756m.g | P | 31079172-31081289 | 8 | 0 | 43.54 | 1.2219 | 0.4723 | 0.3866 | 93.992 |
| Si034189m.g | P | 14623370-14626112 | 9 | Si028006m.g | P | 30333402-30335919 | 8 | 0 | 44.56 | 1.1278 | 0.4562 | 0.4045 | 86.754 |
| Si034189m.g | P | 14623370-14626112 | 9 | Si028348m.g | P | 31201831-31204264 | 8 | 0 | 43.74 | 1.1807 | 0.4702 | 0.3982 | 90.823 |
| Si034392m.g | DYW | 47137893-47140142 | 9 | Si039115m.g | DYW | 4239931-4242342 | 9 | 0 | 40.94 | 9.076 | 0.514 | 0.0566 | 698.15 |
| Si034471m.g | DYW | 48021358-48023478 | 9 | Si032252m.g | E | 26826210-26829263 | 2 | 0 | 41.32 | 14.7065 | 0.5207 | 0.0354 | 1131.3 |
| Si038542m.g | DYW | 15458660-15460753 | 9 | Si034392m.g | DYW | 47137893-47140142 | 9 | 0 | 38.5 | 2.5924 | 0.5614 | 0.2165 | 199.42 |
| Si038662m.g | E | 20681866-20683803 | 9 | Si039680m.g | E | 1296780-1298714 | 9 | 0 | 85.36 | 0.122 | 0.0455 | 0.3725 | 9.3846 |
| Si038752m.g | P | 34987589-34989349 | 9 | Si039389m.g | P | 34488758-34490074 | 9 | 0 | 93.12 | 0.0783 | 0.0337 | 0.4306 | 6.0231 |
| Si039892m.g | P | 14652507-14654795 | 9 | Si034189m.g | P | 14623370-14626112 | 9 | 0 | 52.76 | 0.8953 | 0.3386 | 0.3782 | 68.869 |
|  |  |  |  |  |  |  |  |  | **mean** | 5.81394 | 0.29662 | 0.37643 | 447.23 |
