# Supplementary material for: Genome-wide investigation and expression analyses of the pentatricopeptide repeat protein gene family in foxtail millet
Source: BMC Genomics. 2016 Oct 28;17:840. doi: 10.1186/s12864-016-3184-2 (PMC5084403; doi:10.1186/s12864-016-3184-2)
Supplement: Additional file 13: Table S12. — The PPRs with putative auxiliary motifs or domains in moss, Selaginella, Arabidopsis, foxtail millet, and rice. The domain/class, number of introns within the ORF, and the subcellular localization of the PPR proteins are provided for each PPR gene. (DOCX 29 kb) [file 12864_2016_3184_MOESM13_ESM.docx]

**Table S12.** The PPRs with putative auxiliary motifs or domains in moss, Selaginella, *Arabidopsis*, foxtail millet, and rice. The domain/class, number of introns within the ORF, and the subcellular localization of the PPR proteins are provided for each PPR gene.

| **Gene names** | **Protein ID** | **Alias/Symlbol** | **Auxiliary domain/motif** | **Class** | **Location** | **Introns NO** | **Organism** |
| --- | --- | --- | --- | --- | --- | --- | --- |
| PHYPADRAFT_112597 | A9REG1 |  | LAGLIDADG | P | Chloroplast | 1 | *Physcomitrella patens subsp. patens* (Moss) |
| PHYPADRAFT_41119 | A9T5I5 |  | LAGLIDADG | P | Chloroplast | 1 | *Physcomitrella patens subsp. patens* (Moss) |
| PHYPADRAFT_152956 | A9TZ74 |  | [RNase_Zc3h12a](http://smart.embl-heidelberg.de/smart/javascript:domWin(4)) | P | C/M | 7 | *Physcomitrella patens subsp. patens* (Moss) |
| PHYPADRAFT_174001 | A9RCG0 |  | [RNase_Zc3h12a](http://smart.embl-heidelberg.de/smart/javascript:domWin(3)) | P | Nuc | 7 | *Physcomitrella patens subsp. patens* (Moss) |
| PHYPADRAFT_190279 | A9SZZ6 |  | RRM | P | Chloroplast | 11 | *Physcomitrella patens subsp. patens* (Moss) |
| PHYPADRAFT_12664 | A9SC54 |  | RRM | P | Chloroplast | 9 | *Physcomitrella patens subsp. patens* (Moss) |
| PHYPADRAFT_131253 | A9SK65 |  | Smr | P | Mitochondria | 1 | *Physcomitrella patens subsp. patens* (Moss) |
| PHYPADRAFT_197840 | A9TSP1 |  | Smr | P | Chloroplast | 3 | *Physcomitrella patens subsp. patens* (Moss) |
| PHYPADRAFT_188228 | A9STA3 |  | Smr | P | Chloroplast | 3 | *Physcomitrella patens subsp. patens* (Moss) |
| SELMODRAFT_125731 | D8SH62 |  | TPR | P | C/M | 1 | *Selaginella moellendorffii* (Spikemoss) |
| SELMODRAFT_102729 | D8RS31 |  | TPR | P | C/M | 1 | *Selaginella moellendorffii* (Spikemoss) |
| SELMODRAFT_109608 | D8S5Q5 |  | RRM | P | Chloroplast | 9 | *Selaginella moellendorffii* (Spikemoss) |
| SELMODRAFT_118230 | D8SJB8 |  | TPR | P | C/M | 1 | *Selaginella moellendorffii* (Spikemoss) |
| SELMODRAFT_131102 | D8T3L0 |  | Smr | P | C | 4 | *Selaginella moellendorffii* (Spikemoss) |
| SELMODRAFT_135367 | D8TAA8 |  | Smr | P | C | 4 | *Selaginella moellendorffii* (Spikemoss) |
| SELMODRAFT_30598 | D8SMS2 |  | RRM | P | Chloroplast | 7 | *Selaginella moellendorffii* (Spikemoss) |
| SELMODRAFT_36105 | D8S083 |  | TPR | P | Mitochondria | 1 | *Selaginella moellendorffii* (Spikemoss) |
| SELMODRAFT_402057 | D8QPR3 |  | LRR | P | C/M | 9 | *Selaginella moellendorffii* (Spikemoss) |
| SELMODRAFT_403069 | [D8QNY5](http://www.uniprot.org/uniprot/D8QNY5) |  | UPF0016 | P | - | 26 | *Selaginella moellendorffii* (Spikemoss) |
| SELMODRAFT_412471 | D8RLK7 |  | WD40 SAD_SRA | P | Mitochondria | 20 | *Selaginella moellendorffii* (Spikemoss) |
| SELMODRAFT_412752 | D8RLC9 |  | [Terpene_synth_C](http://smart.embl-heidelberg.de/smart/javascript:domWin(14)) | P | - | 6 | *Selaginella moellendorffii* (Spikemoss) |
| SELMODRAFT_414470 | [D8RSW2](http://www.uniprot.org/uniprot/D8RSW2) |  | UPF0193 | P | - | 13 | *Selaginella moellendorffii* (Spikemoss) |
| SELMODRAFT_417741 | [D8S3G4](http://www.uniprot.org/uniprot/D8S3G4) |  | [RING](http://smart.embl-heidelberg.de/smart/javascript:domWin(3)) | P | C/M | 6 | *Selaginella moellendorffii* (Spikemoss) |
| SELMODRAFT_418683 | [D8S6T9](http://www.uniprot.org/uniprot/D8S6T9) |  | [Abhydrolase_6](http://smart.embl-heidelberg.de/smart/javascript:domWin(9)) | P | - | 8 | *Selaginella moellendorffii* (Spikemoss) |
| SELMODRAFT_420032 | [D8SAC1](http://www.uniprot.org/uniprot/D8SAC1) |  | [SNF](http://smart.embl-heidelberg.de/smart/javascript:domWin(2)) | P | - | 16 | *Selaginella moellendorffii* (Spikemoss) |
| SELMODRAFT_420919 | [D8SDJ3](http://www.uniprot.org/uniprot/D8SDJ3) |  | IGR | P | - | 2 | *Selaginella moellendorffii* (Spikemoss) |
| SELMODRAFT_426357 | [D8SW45](http://www.uniprot.org/uniprot/D8SW45) |  | [Stress-antifung](http://smart.embl-heidelberg.de/smart/javascript:domWin(10)) | P | Mitochondria | 5 | *Selaginella moellendorffii* (Spikemoss) |
| SELMODRAFT_431977 | [D8TEJ9](http://www.uniprot.org/uniprot/D8TEJ9) |  | DnaJ Proteasome | P | Mitochondria | 16 | *Selaginella moellendorffii* (Spikemoss) |
| SELMODRAFT_432224 | [D8TFC7](http://www.uniprot.org/uniprot/D8TFC7) |  | [SET](http://smart.embl-heidelberg.de/smart/javascript:domWin(2)) | P | - | 13 | *Selaginella moellendorffii* (Spikemoss) |
| SELMODRAFT_438108 | - |  | PBP | P | Mitochondria | 5 | *Selaginella moellendorffii* (Spikemoss) |
| SELMODRAFT_438275 | [D8QVL3](http://www.uniprot.org/uniprot/D8QVL3) |  | DEXDc PHD RING HELICc | P | Mitochondria | 20 | *Selaginella moellendorffii* (Spikemoss) |
| SELMODRAFT_438810 | [D8QZL2](http://www.uniprot.org/uniprot/D8QZL2) |  | [LAGLIDADG_2](http://smart.embl-heidelberg.de/smart/javascript:domWin(8)) | P | Chloroplast | 1 | *Selaginella moellendorffii* (Spikemoss) |
| SELMODRAFT_440917 | [D8RFE1](http://www.uniprot.org/uniprot/D8RFE1) |  | IGR | P | C/M | 5 | *Selaginella moellendorffii* (Spikemoss) |
| SELMODRAFT_441099 | [D8RGG6](http://www.uniprot.org/uniprot/D8RGG6) |  | WW DEXDc HELICc | P | C/M | 9 | *Selaginella moellendorffii* (Spikemoss) |
| SELMODRAFT_442782 | [D8RW16](http://www.uniprot.org/uniprot/D8RW16) |  | Plant_tran DDE | P | - | 7 | *Selaginella moellendorffii* (Spikemoss) |
| SELMODRAFT_443353 | [D8S0M9](http://www.uniprot.org/uniprot/D8S0M9) |  | RING EFP | P | Mitochondria | 17 | *Selaginella moellendorffii* (Spikemoss) |
| SELMODRAFT_78126 | [D8QUH2](http://www.uniprot.org/uniprot/D8QUH2) |  | TPR | P | - | 1 | *Selaginella moellendorffii* (Spikemoss) |
| SELMODRAFT_80247 | [D8QWT0](http://www.uniprot.org/uniprot/D8QWT0) |  | RNase_Zc3h12a NYN | P | C/M | 6 | *Selaginella moellendorffii* (Spikemoss) |
| SELMODRAFT_86032 | [D8R5M6](http://www.uniprot.org/uniprot/D8R5M6) |  | TPR | P | C/M | 1 | *Selaginella moellendorffii* (Spikemoss) |
| AT4G01400 | [Q8LDU5](http://www.uniprot.org/uniprot/Q8LDU5) |  | COG4 | P | Mitochondria | 3 | *Arabidopsis thaliana* |
| AT3G22690 | [Q9LUJ2](http://www.uniprot.org/uniprot/Q9LUJ2) |  | DUF1685 | P | Chloroplast | 3 | *Arabidopsis thaliana* |
| AT3G60040 | [Q8GWI2](http://www.uniprot.org/uniprot/Q8GWI2) |  | F-box LRR | P | C/M | 8 | *Arabidopsis thaliana* |
| AT1G12700 | [P0C7Q7](http://www.uniprot.org/uniprot/P0C7Q7) |  | [Helicase_C](http://smart.embl-heidelberg.de/smart/javascript:domWin(46)) | P | Mitochondria | 3 | *Arabidopsis thaliana* |
| AT2G15820 | [Q9XIL5](http://www.uniprot.org/uniprot/Q9XIL5) | OTP51 | [LAGLIDADG_2](http://smart.embl-heidelberg.de/smart/javascript:domWin(8)) | P | Chloroplast | 1 | *Arabidopsis thaliana* |
| AT5G04810 | [Q0WMY5](http://www.uniprot.org/uniprot/Q0WMY5) | ATPPR4 | RRM | P | Chloroplast | 9 | *Arabidopsis thaliana* |
| AT5G21222 | [Q8S9D1](http://www.uniprot.org/uniprot/Q8S9D1) |  | [S_TKc](http://smart.embl-heidelberg.de/smart/javascript:domWin(2)) | P | Mitochondria | 10 | *Arabidopsis thaliana* |
| AT1G18900 | [Q8GYP6](http://www.uniprot.org/uniprot/Q8GYP6) |  | Smr | P | Mitochondria | 3 | *Arabidopsis thaliana* |
| AT1G74750 | [Q9SSF9](http://www.uniprot.org/uniprot/Q9SSF9) |  | Smr | P | Mitochondria | 0 | *Arabidopsis thaliana* |
| AT1G74850 | [Q9S7Q2](http://www.uniprot.org/uniprot/Q9S7Q2) | pTAC2 | Smr | P | Chloroplast | 3 | *Arabidopsis thaliana* |
| AT1G79490 | [Q9SAK0](http://www.uniprot.org/uniprot/Q9SAK0) | EMB2217 | Smr | P | Mitochondria | 0 | *Arabidopsis thaliana* |
| AT2G17033 | [Q8GWA9](http://www.uniprot.org/uniprot/Q8GWA9) |  | Smr | P | Chloroplast | 2 | *Arabidopsis thaliana* |
| AT2G31400 | [Q9SIC9](http://www.uniprot.org/uniprot/Q9SIC9) | GUN1 | Smr | P | Chloroplast | 3 | *Arabidopsis thaliana* |
| AT4G16390 | [Q8GWE0](http://www.uniprot.org/uniprot/Q8GWE0) | SVR7 | Smr | P | Chloroplast | 0 | *Arabidopsis thaliana* |
| AT5G46580 | [Q9LS25](http://www.uniprot.org/uniprot/Q9LS25) |  | Smr | P | Chloroplast | 0 | *Arabidopsis thaliana* |
| Si028837m.g | [K3ZQF7](http://www.uniprot.org/uniprot/K3ZQF7) |  | Abhydrolase_5 | E | Chloroplast | 7 | Foxtail millet |
| Si034581m.g | [K4A6S6](http://www.uniprot.org/uniprot/K4A6S6) |  | EXOIII | E | C/M | 3 | Foxtail millet |
| Si028853m.g | [K3ZQH3](http://www.uniprot.org/uniprot/K3ZQH3) |  | HAT | PLS | Secretory pathway | 6 | Foxtail millet |
| Si016353m.g | [K3YQ11](http://www.uniprot.org/uniprot/K3YQ11) |  | LAGLIDADG_2 | P | Chloroplast | 1 | Foxtail millet |
| Si000105m.g | [K3XDY8](http://www.uniprot.org/uniprot/K3XDY8) |  | LysM Pkinase Pkinase_Tyr | P | C/M | 10 | Foxtail millet |
| Si028249m.g | [K3ZNR9](http://www.uniprot.org/uniprot/K3ZNR9) |  | PGAM | P | Chloroplast | 7 | Foxtail millet |
| Si028360m.g | [K3ZP30](http://www.uniprot.org/uniprot/K3ZP30) |  | PGAM | PLS | Chloroplast | 9 | Foxtail millet |
| Si034560m.g | [K4A6Q5](http://www.uniprot.org/uniprot/K4A6Q5) |  | Pkinase_Tyr Kdo RIO1 | P | Mitochondria | 6 | Foxtail millet |
| Si021162m.g | [K3Z3P4](http://www.uniprot.org/uniprot/K3Z3P4) |  | RRM | PLS | Chloroplast | 9 | Foxtail millet |
| Si028921m.g | [K3ZQP1](http://www.uniprot.org/uniprot/K3ZQP1) |  | TPR | P | Mitochondria | 2 | Foxtail millet |
| Si020193m.g | [K3Z0X6](http://www.uniprot.org/uniprot/K3Z0X6) |  | ZnF_C2H2 | PLS | Mitochondria | 6 | Foxtail millet |
| Si013172m.g | [K3YG04](http://www.uniprot.org/uniprot/K3YG04) |  | DYW DAGKc DAGKa | DYW | Chloroplast | 12 | Foxtail millet |
| Si027770m.g | [K3ZMF5](http://www.uniprot.org/uniprot/K3ZMF5) |  | PLAC8 DUF2985 | DYW | C/M | 1 | Foxtail millet |
| LOC_Os04g28300 | [Q7X919](http://www.genevestigator.com/gv/directlink.jsp?geneIDs=Q7X919&geneIDType=SwissProt) |  | Ankyrin repeat | P | C/M | 2 | *Oryza sativa* |
| LOC_Os03g59264 | [Q10BR5](http://www.uniprot.org/uniprot/Q10BR5) |  | [Calreticulin](http://smart.embl-heidelberg.de/smart/javascript:domWin(115)) | P | Chloroplast | 9 | *Oryza sativa* |
| LOC_Os08g15090 | [A2YSZ8](http://www.uniprot.org/uniprot/A2YSZ8) |  | DAGKc DAGKa | P | Chloroplast | 13 | *Oryza sativa* |
| LOC_Os04g58980 | [A2XZ66](http://www.uniprot.org/uniprot/A2XZ66) |  | DUF167 | DYW | Mitochondria | 3 | *Oryza sativa* |
| LOC_Os01g42990 | [Q7X919](http://www.genevestigator.com/gv/directlink.jsp?geneIDs=Q7X919&geneIDType=SwissProt) |  | FAD_binding_4 | P | Mitochondria | 4 | *Oryza sativa* |
| LOC_Os09g39970 | [Q5Z484](http://www.uniprot.org/uniprot/Q5Z484) |  | [IPPc](http://smart.embl-heidelberg.de/smart/javascript:domWin(83)) | DYW | Chloroplast | 10 | *Oryza sativa* |
| LOC_Os02g47360 | [Q6ZHJ5](http://www.uniprot.org/uniprot/Q6ZHJ5) | OSOTP 51 | [LAGLIDADG_2](http://smart.embl-heidelberg.de/smart/javascript:domWin(12)) | P | Chloroplast | 1 | *Oryza sativa* |
| LOC_Os03g28270 | [Q94H87](http://www.uniprot.org/uniprot/Q94H87) |  | LRR | P | C/M | 9 | *Oryza sativa* |
| LOC_Os02g58620 | [Q6K957](http://www.uniprot.org/uniprot/Q6K957) |  | MatE | P | Mitochondria | 10 | *Oryza sativa* |
| LOC_Os07g20510 | [Q6YS35](http://www.uniprot.org/uniprot/Q6YS35) |  | Mem_tans | P | Mitochondria | 7 | *Oryza sativa* |
| LOC_Os12g04110 | [Q2QY23](http://www.uniprot.org/uniprot/Q2QY23) |  | p450 PGAM | P | C/M | 7 | *Oryza sativa* |
| LOC_Os12g01910 | [Q2QYQ4](http://www.uniprot.org/uniprot/Q2QYQ4) |  | [Pkinase_Tyr](http://smart.embl-heidelberg.de/smart/javascript:domWin(16)) | P | C/M | 3 | *Oryza sativa* |
| LOC_Os06g20354 | [Q5Z4M3](http://www.uniprot.org/uniprot/Q5Z4M3) |  | [Ribosomal_L15e](http://smart.embl-heidelberg.de/smart/javascript:domWin(1)) | P | C/M | 2 | *Oryza sativa* |
| LOC_Os02g17360 | [B8AFN5](http://www.uniprot.org/uniprot/B8AFN5) |  | [RNase_Zc3h12a](http://smart.embl-heidelberg.de/smart/javascript:domWin(36)) | P | Chloroplast | 5 | *Oryza sativa* |
| LOC_Os04g58780 | [Q0J8W3](http://www.gramene.org/db/protein/protein_search?acc=Q0J8W3) |  | RRM | P | Chloroplast | 9 | *Oryza sativa* |
| LOC_Os06g12360 |  |  | [RWP-RK](http://smart.embl-heidelberg.de/smart/javascript:domWin(143)) | P | Mitochondria | 5 | *Oryza sativa* |
| LOC_Os07g10400 | [Q5JME5](http://www.uniprot.org/uniprot/Q5JME5) |  | [S_TKc](http://smart.embl-heidelberg.de/smart/javascript:domWin(81)) | P | Mitochondria | 6 | *Oryza sativa* |
| LOC_Os10g28640 | [Q338A3](http://www.uniprot.org/uniprot/Q338A3) |  | [STYKc](http://smart.embl-heidelberg.de/smart/javascript:domWin(63)) | P | Mitochondria | 6 | *Oryza sativa* |
