# Supplementary material for: Genome-wide investigation and expression analyses of the pentatricopeptide repeat protein gene family in foxtail millet
Source: BMC Genomics. 2016 Oct 28;17:840. doi: 10.1186/s12864-016-3184-2 (PMC5084403; doi:10.1186/s12864-016-3184-2)
Supplement: Additional file 16: Table S14. — PPR genes differentially expressed under drought treatment (20 % PEG 6000), salt treatment (250 mM NaCl), cold treatment (4 °C), and treatment with 100 μM ABA, at the 0, 6, and 24 h time points. (DOCX 19 kb) [file 12864_2016_3184_MOESM16_ESM.docx]

**Table S14.** PPR genes differentially expressed under drought treatment (20% PEG 6000), salt treatment (250 mM NaCl), cold treatment (4°C), and treatment with 100 μM ABA, at the 0, 6, and 24 h time points.

| **Phytozome ID** | **Control** | **Drought-6h** | **Drought-24h** | **NaCl-6h** | **NaCl-24h** | **Cold-6h** | **Cold-24h** | **ABA-6h** | **ABA-24h** |
| --- | --- | --- | --- | --- | --- | --- | --- | --- | --- |
| Si008179m | 1 | 1.042183146 | 0.949217289 | 2.6548521 | 1.9654 | 1.99877235 | 1.55635913 | 1.131841828 | 1.202328734 |
| Si006246m | 1 | 1.09895239 | 0.850815952 | 1.2354 | 1.023654 | 0.949219623 | 1.224488263 | 0.74958991 | 0.78958991 |
| Si000925m | 1 | 1.139013682 | 0.826077509 | 0.98778154 | 0.49874 | 0.849219623 | 1.124488263 | 1.233947284 | 0.989602126 |
| Si019498m | 1 | 0.425391478 | 0.771245466 | 0.98367025 | 0.698437612 | 0.804766107 | 1.060670906 | 1.321054 | 1.03203 |
| Si012220m | 1 | 1.15045164 | 1.358968233 | 1.118581328 | 0.912712588 | 1.318588354 | 1.057822684 | 0.912227278 | 0.502965284 |
| Si009504m | 1 | 1.350851582 | 3.124048314 | 9.460605365 | 4.949651684 | 1.081558075 | 2.575456124 | 0.912712588 | 3.837956587 |
| Si029147m | 1 | 1.496602589 | 0.31524366 | 4.519489882 | 3.092352916 | 11.37933228 | 2.405869348 | 2.16286007 | 0.96648059 |
| Si005765m | 1 | 1.463258232 | 0.833067674 | 10.50519165 | 0.432948134 | 0.559393157 | 1.400028066 | 1.415897857 | 2.574230654 |
| Si020193m | 1 | 1.35505153 | 1.179425982 | 35.79995017 | 18.3654789 | 3.182477895 | 1.219961158 | 0.464050516 | 1.247840685 |
| Si039439m | 1 | 2.16286007 | 0.96648059 | 1.357824253 | 0.812712588 | 1.62756515 | 0.602328734 | 1.481787257 | 0.767002743 |
| Si032871m | 1 | 15.3654 | 33.6472815 | 15.68421262 | 18.27689717 | 17.31147922 | 55.7200591 | 3.753961147 | 1.305638605 |
| Si006059m | 1 | 0.562623101 | 26.78102625 | 3.324767027 | 4.003298105 | 0.287748888 | 1.487493905 | 3.039508715 | 0.327676857 |
| Si008652m | 1 | 0.5505153 | 0.2425982 | 0.15782573 | 0.531841828 | 0.331841828 | 0.53812 | 0.6949291 | 0.65487 |
| Si000860m | 1 | 0.43263801 | 0.162268575 | 0.188886036 | 0.435829101 | 0.780559193 | 0.468492323 | 0.30165142 | 0.144986988 |
| Si034185m | 1 | 0.337821757 | 0.05373454 | 0.879013821 | 1.0390161 | 0.233636856 | 0.634341373 | 0.380659099 | 0.66231386 |
| Si029528m | 1 | 0.310365143 | 0.07663271 | 1.874391552 | 0.091261866 | 1.984192741 | 1.000650282 | 0.371857572 | 0.357689689 |
| Si005691m | 1 | 1.006165142 | 0.465024071 | 1.586193796 | 0.177875315 | 1.579715828 | 8.684462581 | 0.689584521 | 0.359095623 |
| Si013441m | 1 | 0.878258306 | 0.196218525 | 8.636054069 | 0.862464961 | 2.408081292 | 2.027637725 | 1.060910579 | 0.817907413 |
| Si028351m | 1 | 0.812135372 | 0.948847045 | 2.934214415 | 1.954602833 | 3.001095866 | 0.424337426 | 3.941828 | 1.357689689 |
| Si038752m | 1 | 0.565952021 | 0.593072761 | 6.899013682 | 1.037601368 | 4.08500523 | 3.957731296 | 1.918588354 | 0.626832393 |
| Si001059m | 1 | 1.15505153 | 1.269425982 | 11.41897667 | 2.578080653 | 2.532455884 | 0.843761329 | 1.654789 | 1.02564 |
| Si003975m | 1 | 1.15505153 | 1.269425982 | 4.502225298 | 3.87039537 | 29.68486923 | 2.443454552 | 0.949219623 | 0.74958991 |
| Si015629m | 1 | 1.154494638 | 0.954494638 | 3.559211845 | 0.088258559 | 3.102738131 | 10.21720672 | 0.17260799 | 0.095515148 |
| Si016319m | 1 | 0.470996241 | 0.599767743 | 2.292480015 | 1.73008642 | 9.941408239 | 1.564563738 | 0.7625491 | 0.9874512 |
| Si016833m | 1 | 0.446538245 | 0.041852977 | 1.19714804 | 1.108814804 | 0.929214415 | 1.412783403 | 0.192537172 | 0.034949807 |
| Si004217m | 1 | 2.222644504 | 1.174396542 | 0.380659099 | 1.131841828 | 3.82756515 | 0.760232354 | 0.364958991 | 0.9874512 |
| Si039699m | 1 | 1.015505153 | 0.956942598 | 0.879021096 | 1.003901 | 0.996456374 | 1.016456374 | 0.227234079 | 0.172210174 |
| Si034277m | 1 | 2.478625718 | 1.143150968 | 1.269840811 | 0.243590404 | 0.600691931 | 0.729586629 | 1.057822684 | 0.882344489 |
| Si028944m | 1 | 0.97520088 | 0.832743602 | 11.56523161 | 3.167284985 | 1.197520088 | 1.269425982 | 0.927113525 | 1.338253758 |
| Si000626m | 1 | 1.060912944 | 1.322167007 | 2.547771799 | 1.472512447 | 1.101722663 | 0.609978748 | 0.899797195 | 1.060912944 |
| Si034377m | 1 | 0.607645415 | 0.46800567 | 1.269838711 | 3.843590001 | 0.746216542 | 0.864483331 | 1.057822684 | 0.882344489 |
