# Supplementary material for: Genome-wide investigation and expression analyses of the pentatricopeptide repeat protein gene family in foxtail millet
Source: BMC Genomics. 2016 Oct 28;17:840. doi: 10.1186/s12864-016-3184-2 (PMC5084403; doi:10.1186/s12864-016-3184-2)
Supplement: Additional file 17: Table S15. — Primer sequences used for real-time PCR analysis. (DOCX 15 kb) [file 12864_2016_3184_MOESM17_ESM.docx]

**Table S15.** Primer sequences used for real-time PCR analysis.

| **Gene identifier** | **Primer sequences** |
| --- | --- |
| Si000626m | 5' CAAGACTGCACGATGAGGGT 3' |
|  | 5' TGGAACAGATTAGGACTATGGC 3' |
| Si000860m | 5' CCCGAGTTTTGGGACTTGAT 3' |
|  | 5' CGTGACATTGTCTGGCTGGT 3' |
| Si003975m | 5' GAAGGAAATGCTGGAATCTGA 3' |
|  | 5' TGGCATCAACAAGGTTAGGAC 3' |
| Si005691m | 5' GGATTCCCAGAAACAGCACA 3' |
|  | 5' ACCTCAAATACATCACCACCTT 3' |
| Si005765m | 5' ATGAACCAAGCGTTGTAGCC 3' |
|  | 5' CTGCAAAGACGAGATCATGTAA 3' |
| Si009504m | 5' CCAATTTCGCCTGCTAACG 3' |
|  | 5' CTCATCGGGCAGGTTCTTGG 3' |
| Si013441m | 5' TTTCCCTTGCCAAAACACCA 3' |
|  | 5' CCTCTTCCTCATAATCATCATCG 3' |
| Si016319m | 5' TCTATGCCAAACCATTTTACCT 3' |
|  | 5' TGTCACTGGGGTGTAGTCCTG 3' |
| Si020193m | 5' CAAAATCTTCCGCTGTTATGC 3' |
|  | 5' ATTTCCAACGACCACCTCAG 3' |
| Si028351m | 5' GCCGCTACTTGGACCACATT 3' |
|  | 5' GGCCTCCTTGCTCACATTCC 3' |
| Si028944m | 5' GCTGCTATACCTCCTACTCGAA 3' |
|  | 5' CCCATACTGCCCAACCTTTAC 3' |
| Si029147m | 5' AGAGCAAGAGCGTTGGGAGG 3' |
|  | 5' GCTCATAGCCTTGACCACATTT 3' |
| Si029528m | 5' GAGGAATGGGCGTCCAAAAG 3' |
|  | 5' CCGCAAAAGCATAGGTGTTA 3' |
| Si034185m | 5' CTGGGAACAGGGTAAAATCA 3' |
|  | 5' CAAACATATCACAACGTCCATAAA 3' |
| Si034377m | 5' GATGTCGGAGTCATGGAAATAT 3' |
|  | 5' GGTAAGGTTCATAGCCAAAGG 3' |
| Si038752m | 5' TTATGAAGGGTGGGTGGAGC 3' |
|  | 5' GTGTTTCTTCTTGGCATCTTATC 3' |
| Si039699m | 5' TCTTGGAACACGCTTATCTCA 3' |
|  | 5' AATCGGCATTTGCTCTATCA 3' |
| Si000925m | 5' GGGCAGTTGCTTGTGGCT 3' |
|  | 5' TTGGGGCGTGTTCTAGTCA 3' |
| Si006246m | 5' GGCATCGAACACTACGGC 3' |
|  | 5' TTCCTCATCTGCTTCCTCATC 3' |
| Si008179m | 5' TGAAATCTGAGCGAATAAAACC 3' |
|  | 5' ATCCCTTTGACAAGAAACCATT 3' |
| Si015629m | 5' TGTGGACGAGGTCAGGAAGG 3' |
|  | 5' TGAGGACGAACGGGAACG 3' |
| Si016833m | 5' AATGGGATTCCTCCAAGTGC 3' |
|  | 5' GAACCCTGCTCGGCTGTA 3' |
| Si028351m | 5' TATGGAAGAGCAGGGCAACT 3' |
|  | 5' TCGACCTCGATCACGGAC 3' |
| Si006059m | 5' TAGGCTGATGGAGCAGTTTG 3' |
|  | 5' TCCTGAGATTGGGATGGACG 3' |
| Si032871m | 5' AGCGTAACCTCGTCTCCTGG 3' |
|  | 5' TCCGACCATCGTTATTTCAG 3' |
| Si001059m | 5' TGCTTCTCAAAGACTGGGAGT 3' |
|  | 5' TAAGCAACGCATGAAAGGTC 3' |
| Si019498m | 5' CACAGGTTCCACCAGACAAT 3' |
|  | 5' CAAGCCAGGAGAATACGCTAT 3' |
| Si004217m | 5' GGTTTGACGCTATGAAGGAA 3' |
|  | 5' GCCTCTCACAACTATGCTGC 3' |
| Si008652m | 5' GGAAGTGCTCTAAAGGCTTG 3' |
|  | 5' GCATCACCCATAAGTTGCT 3' |
| Si012220m | 5' TGTTTGTTTGGGTGGTCG 3' |
|  | 5' CCAGCAGCAAGAATGTTGA 3' |
| Si019498m | 5' ACCATCATCGCCCTCTACT 3' |
|  | 5' TTCCTCTCATCCATCTCGTT 3' |
| Si039439m | 5' AAGGCTTCAGGCTCTTCGT 3' |
| actin | 5' CGACACCAAGTCTGGCTTT 3' |
| actin | 5′-CTGACGCCGAGGATATCCA-3′ |
