# Supplementary material for: Genome-wide investigation and expression analyses of the pentatricopeptide repeat protein gene family in foxtail millet
Source: BMC Genomics. 2016 Oct 28;17:840. doi: 10.1186/s12864-016-3184-2 (PMC5084403; doi:10.1186/s12864-016-3184-2)
Supplement: Additional file 18: Table S16. — Primer sequences used for the subcellular localization analysis of the PPR proteins in foxtail millet. (DOCX 14 kb) [file 12864_2016_3184_MOESM18_ESM.docx]

**Table S16.** Primer sequences used for the subcellular localization analysis of the PPR proteins in foxtail millet..

| **Gene identifier** | **Primer sequences** |
| --- | --- |
| Si009504m | 5' TCTGAATCCTGATACTCTCTGG 3' |
|  | 5' TAGGCAATCTGTCCAAGAATCC 3' |
| Si005765m | 5' TGTGTGTGCGTAGTTGATGG 3' |
|  | 5' TCTACTGCGTGTATGTATGGTG 3' |
| Si013441m | 5' TGTCCTGTTCCAATGGAAGC 3' |
|  | 5' TTGTATATGAAATGCCATCATG 3' |
| Si028351m | 5' GAGGAAGAAGAAGAAGCAG 3' |
|  | 5' AAGAACACGCTTACCTGGG 3' |
| Si038752m | 5' CTAACCAAATCCGCAGTTTC 3' |
|  | 5' GCACAATCAGACGAAGAAAC 3' |
| Si020193m | 5' TTGGTCGTAGTAGTCTCGG 3' |
|  | 5' CTCGGAGCAGAGAAACTT 3' |
| Si001059m | 5' AGAGGAGGGCGATTAGTCT 3' |
|  | 5' ATGGAAGCATTCAGGTGG 3' |
| Si039439m | 5' TTCGGTTTGAGTCCATACG 3' |
|  | 5' TAGCGGTTCGCAAAGAAG 3' |
| Si020193m | 5' CTCTCCACTCCATTCTCACTC 3' |
|  | 5' CCATACACACACACACACCAT 3' |
| Si032871m | 5' GCTGTTGGACTGTTGGTTGT 3' |
|  | 5' GATTAGAACAGCCAGATTCCAC 3' |
| Si006059m | 5' TACTGAGCTGGACTGGACTGC 3' |
|  | 5' TTGAAATGCTTGGCACTGC 3' |
| Si009504m | 5' TCTGAATCCTGATACTCTCTGG 3' |
|  | 5' TAGGCAATCTGTCCAAGAATCC 3' |
| Si005765m | 5' TGTGTGTGCGTAGTTGATGG 3' |
|  | 5' TCTACTGCGTGTATGTATGGTG 3' |
| Si013441m | 5' TGTCCTGTTCCAATGGAAGC 3' |
|  | 5' TTGTATATGAAATGCCATCATG 3' |
| Si028351m | 5' GAGGAAGAAGAAGAAGCAG 3' |
|  | 5' AAGAACACGCTTACCTGGG 3' |
| Si038752m | 5' CTAACCAAATCCGCAGTTTC 3' |
|  | 5' GCACAATCAGACGAAGAAAC 3' |
| Si020193m | 5' TTGGTCGTAGTAGTCTCGG 3' |
|  | 5' CTCGGAGCAGAGAAACTT 3' |
| Si001059m | 5' AGAGGAGGGCGATTAGTCT 3' |
|  | 5' ATGGAAGCATTCAGGTGG 3' |
| Si039439m | 5' TTCGGTTTGAGTCCATACG 3' |
|  | 5' TAGCGGTTCGCAAAGAAG 3' |
| Si020193m | 5' CTCTCCACTCCATTCTCACTC 3' |
|  | 5' CCATACACACACACACACCAT 3' |
| Si032871m | 5' GCTGTTGGACTGTTGGTTGT 3' |
|  | 5' GATTAGAACAGCCAGATTCCAC 3' |
| Si006059m | 5' TACTGAGCTGGACTGGACTGC 3' |
|  | 5' TTGAAATGCTTGGCACTGC 3' |
| Si009504m | 5' TCTGAATCCTGATACTCTCTGG 3' |
|  | 5' TAGGCAATCTGTCCAAGAATCC 3' |
| Si005765m | 5' TGTGTGTGCGTAGTTGATGG 3' |
|  | 5' TCTACTGCGTGTATGTATGGTG 3' |
| Si013441m | 5' TGTCCTGTTCCAATGGAAGC 3' |
|  | 5' TTGTATATGAAATGCCATCATG 3' |
